# Taxonomic review of *Copella* (Characiformes: Lebiasinidae) with an identification key for the species

**DOI:** 10.1371/journal.pone.0183069

**Published:** 2017-08-17

**Authors:** Manoela M. F. Marinho, Naércio A. Menezes

**Affiliations:** Museu de Zoologia da Universidade de São Paulo, Ipiranga, São Paulo, São Paulo, Brazil; BRAZIL

## Abstract

A taxonomic review of *Copella* is presented based on the analysis of the type material of all nominal species and extensive material from South American drainages. Six out of ten nominal species are recognized as valid: *Copella arnoldi*, *C*. *callolepis*, *C*. *compta*, *C*. *eigenmanni*, *C*. *nattereri*, and *C*. *vilmae*. *Copella carsevennensis* is a junior synonym of *C*. *arnoldi*, *C*. *nigrofasciata* and *‘Nannostomus’ stigmasemion* are junior synonyms of *C*. *callolepis*, *C*. *metae* is junior synonym of *C*. *eigenmanni*, and *C*. *meinkeni* is junior synonym of *C*. *nattereri*. Species of *Copella* occur in the rio Amazonas and Orinoco basins, and coastal drainages of Guyana, French Guiana, Surinam, and Venezuela. An identification key is provided.

## Introduction

Fishes of the neotropical family Lebiasinidae occur in Central America (Costa Rica and Panama) and all South American countries, except Chile [1]. The family includes 75 valid species [2]. distributed in two subfamilies, Lebiasininae and Pyrrhulininae, and seven genera: *Copeina* Fowler, *Copella* Myers, *Derhamia* Géry & Zarske, *Lebiasina* Valenciennes, *in* Cuvier & Valenciennes, *Nannostomus* Günther, *Piabucina* Valenciennes, and *Pyrrhulina* Valenciennes [1]. Members of the family can be recognized by having a rather elongate, cylindrical body, large scales, laterosensory canal of head and body reduced, anal fin short-based, no frontal or parietal fontanels [1], and the absence of the metapterygoid-quadrate fenestra [3].

The genus *Copella* includes small species, reaching a maximum size of 52.0 mm SL. They are popular among aquarists due to their peaceful behavior and colorful bodies. The genus was erected by Myers [4] based on the presence of a maxilla triply curved (“S” shaped), more pronounced in males. In the same paper, Myers included *Copeina compta* Myers, *Pyrrhulina nattereri* Steindachner, and *Copeina callolepis* Regan in *Copella* and designated *Copella compta* as the type species. Although Myers [4] stated that no other species of the “*Pyrrhulina*-*Copeina*” group could be identified as *Copella*, Géry [5] found that all the species previously recognized as *Copeina* (*C*. *arnoldi* Regan, *C*. *carsevennensis* Regan, *C*. *eigenmanni* Regan, *C*. *metae* Eigenmann, and *C*. *nigrofasciata* Meinken) should be transferred to *Copella*, except *Copeina guttata* (Steindachner) and *Copeina osgoodi* Eigenmann. Later on, Géry [6] and Zarske & Géry [7] described *Copella vilmae* and *C*. *meinkeni*, respectively.

The taxonomic problems related to the species of *Copella* range from unclear diagnoses [5], type material not represented by the same species [1] and literature misidentifications [7]. Currently, the genus includes ten nominal species: *Copella arnoldi*, *C*. *carsevennensis*, *C*. *callolepis*, *C*. *compta*, *C*. *eigenmanni*, *C*. *metae*, *C*. *nattereri*, *C*. *nigrofasciata*, *C*. *vilmae*, and *C*. *meinkeni*.

Examination of a large amount of material of *Copella* from the Amazon and Orinoco basins, and coastal drainages of Brazil, Guyana, French Guiana, Suriname, and Venezuela, as well as type material of all nominal species, made possible the most- comprehensive taxonomic revision of the genus. A dichotomous identification key and distribution maps are also presented.

## Material and methods

Counts and measurements follow Fink & Weitzman [8], with the addition of depth at dorsal-fin origin, pectoral to pelvic-fin origin, pelvic to anal-fin origin, and anal-fin base length, measured point to point. Additional meristic data are the first longitudinal scale row on body, the fourth longitudinal scale row, which is the mid-lateral scale series including the first three small scales posterior to opercle, the longitudinal scale rows between dorsal-fin origin and pelvic-fin origin, and longitudinal scale rows between dorsal-fin origin and anal-fin origin. Principal caudal-fin rays include all branched rays plus one unbranched ray in each lobe, following Hubbs & Lagler [9] and Lundberg & Baskin [10]. Teeth and anterior unbranched anal-fin ray counts were taken from clear and stained material (c&s), which was prepared according to Taylor & van Dyke [11]. Count of maxillary teeth of *C*. *vilmae* was made on specimens preserved in alcohol by transparency, because insufficient material was available for c&s preparations. Teeth were only counted in adult specimens, since juveniles have fewer teeth that are difficult to detect. Meristics of the holotype of ‘*Nannostomus’ stigmasemion* were not taken due to the poor condition of the specimen.

In species descriptions, counts are followed by their frequencies in parentheses. Asterisks indicate counts of holotype, lectotype, or syntypes. Measurements are given as percents of standard length (SL), except for subunits of the head given as percents of head length. Counts of vertebrae were made in c&s specimens and through x-rays. Vertebrae of the Weberian apparatus were counted as four elements and the fused PU1+U1 of the caudal region as a single element. Color in alcohol description does not follow the nomenclature proposed by Weitzman [12] for *Nannostomus* to avoid problems of homology (the longitudinal mid-lateral stripe on body, defined as “primary stripe”, does not seem to correspond to the same melanophore pattern within *Copella*). In the color in life section, description of colors are based on observation of freshly collected specimens of *C*. *arnoldi*, *C*. *eigenmanni*, and *C*. *nattereri* and on photographs of live specimens for the remaining species. Species citations or characterized in Aquarium magazines are not included in the synonym list, except when the articles contain important taxonomic considerations (*e*.*g*. description of *Pyrrhulina nigrofasciata*).

For sexual determination, the anal-fin inclinator muscle of the last pterygiophore was used. This structure is thicker and inserted more distally in the last anal-fin ray of males than of females of several lebiasinids, especially *Copella*. This was confirmed by examination of the gonads of one male and one female of *Copella arnoldi* (MZUSP 105776), *C*. *eigenmanni* (MZUSP 81443), *C*. *nattereri* (MZUSP 87426), and *C*. *stigmasemion* (MZUSP 101933). The anal-fin inclinator muscle is considerably thicker even in early developmental stages of males, before other external secondary sexual features appear, such as dimorphic coloration or elongate fins. Therefore, this structure was used to sex *Copella*. Due to the impossibility to unambiguously distinguish female and immature males (fem/imm), they were treated together.

Catalog numbers are followed by the number of specimens in alcohol, the number of c&s specimens, if any, and their SL range. Municipality originally referred as Tapurucuara was treated as Santa Isabel do Rio Negro, the correct name of the city. In the geographic distribution map, localities with no specific data were plotted over the respective city.

Most specimens analysed are from fish collections specified below. Few specimens captured in this study were collected under permit number 26281–1 issued by Instituto Brasileiro do Meio Ambiente e dos Recursos Naturais Renováveis (IBAMA), in areas not protected in any way, and the field studies did not involve endangered or protected species. They were killed with an overdose of anaesthetic MS-222 and then fixed in formalin. This study is part of the project number 226/2015 approved by brazilian ethics committee Comissão de Ética no Uso de Animais (CEUA) do Instituto de Biociências da Universidade de São Paulo under Credenciamento Institucional para Atividades com Animais em Ensino ou Pesquisa Científica (CIAEP), number 01.0165.2014, Conselho Nacional de Controle de Experimentação Animal (CONCEA) do Ministério da Ciência, Tecnologia e Inovação (MCTI). Institutional abbreviations are AMNH, American Museum of Natural History, New York, USA; ANSP, Academy of Natural Science of Philadelphia, USA; BMNH, Natural History Museum, London, UK; DZSJRP, Departamento de Zoologia e Botânica da Universidade Estadual Paulista, São José do Rio Preto, Brazil; CAS, California Academy of Science, San Francisco, USA; CM, Carnegie Museum, now at FMNH; CZUT-IC, Colleción Zoológica de la Universidad del Tolima, Ictiolgía, Ibagué, Colombia; FMNH, Field Museum of Natural History, Chicago, USA; IavH, Colección de peces dulceacuícolas del Instituto Alexander Von Humboldt, Villa de Leyva, Colombia; ICNMHN, Unidad de Ictiologia del Instituto de Ciências Naturales, Museo de Historia Natural, Universidad Nacional de Colombia, Bogotá, Colombia; IU, Indiana University (now distributed among several North American museums); INPA, Insituto Nacional de Pesquisa da Amazônia, Manaus, Brazil; MBUCV, Museo de Biologia, Universidad Central de Venezuela, Caracas, Venezuela; MCNG, Museo de Ciencias Naturales, Guanare, Venezuela; MCP, Museu de Ciências e Tecnologia da Pontifícia Universidade Católica do Rio Grande do Sul, Porto Alegre, Brasil; MCZ, Museum of Comparative Zoology, Cambridge, USA; MHNG, Museum d’Histoire naturelle, Geneve, Switzerland; MNHN, Museum National d'Histoire Naturelle, Paris, France; MHNLS, Museo de Historia Natural La Salle, Caracas, Venezuela; MLS, Museo de La Salle, Universidad de La Salle, Bogotá, Colombia; MPEG, Museu Paraense Emílio Goeldi, Belém, Brazil; MSNG, Museo Civico di Storia Naturale di Genova ‘Giacomo Doria’. Genova, Italy. MTD F, Museum für Tierkunde, Senckenberg Naturhistorische Sammlungen Dresden, Dresden, Germany; MZUSP, Museu de Zoologia da Universidade de São Paulo, São Paulo, Brazil; NMW, Naturhistorisches Museum, Vienna, Austria; NRM, Naturhistoriska riksmuseet, Stockholm, Sweden; SIU, Southern Illinois University at Carbondale, Carbondale, USA; SMF, Senckenberg-Museum, Frankfurt am Main, Germany; SU, Stanford University, now in CAS; UNIR Fundação Universidade Federal de Rondônia, Porto Velho, Brazil; USNM, National Museum of Natural History, Smithsonian Institution, Washington, D.C., USA; ZMA, Zoologisches Museum, Universiteit van Amsterdam, Amsterdam, The Netherlands; ZMB, Zoologisches Museum, Humboldt-Universitat, Berlin, Germany; ZMH, Zoologisches Museum und Zoologisches Institut, Universität Hamburg, Hamburg, Germany, ZMUC, Zoological Museum, University of Copenhagen, Copenhagen, Denmark.

## Results and discussion

Based on the analysis of type material of all the nominal species and of a large number of *Copella* specimens from South American drainages, six of the ten nominal species of the genus are recognized as valid: *Copella arnoldi*, *C*. *callolepis*, *C*. *compta*, *C*. *eigenmanni*, *C*. *nattereri*, and *C*. *vilmae*. *Copella carsevennensis* is a junior synonym of *C*. *arnoldi*, *C*. *nigrofasciata* and *‘Nannostomus’ stigmasemion* are junior synonyms of *C*. *callolepis*, *C*. *metae* is junior synonym of *C*. *eigenmanni*, and *C*. *meinkeni* is junior synonym of *C*. *nattereri*.

### *Copella* Myers, 1956

*Copella* Myers [4]: 12 [*C*. *compta*, type species by original designation; included *Copeina callolepis* and *Pyrrhulina nattereri* in *Copella*].—Weitzman [3]: 150 [osteological notes; included in subtribe Pyrrhulinina].—Weitzman & Cobb [13]: 2 [in tribe Pyrrhulinini].—Vari [14]: 5 [phylogenetic analysis of the Ctenoluciidae].—Weitzman & Weitzman [1]: 241 [literature compilation].—Oyakawa & Netto-Ferreira, [15]: 64 [literature compilation].

**Type species.** *Copeina compta* Myers. Type by original designation.

**Gender.** Feminine.

**Included species.** *Copella arnoldi*, *C*. *compta*, *C*. *eigenmanni*, *C*. *nattereri*, *C*. *callolepis*, and *C*. *vilmae*.

**Fig 1:**
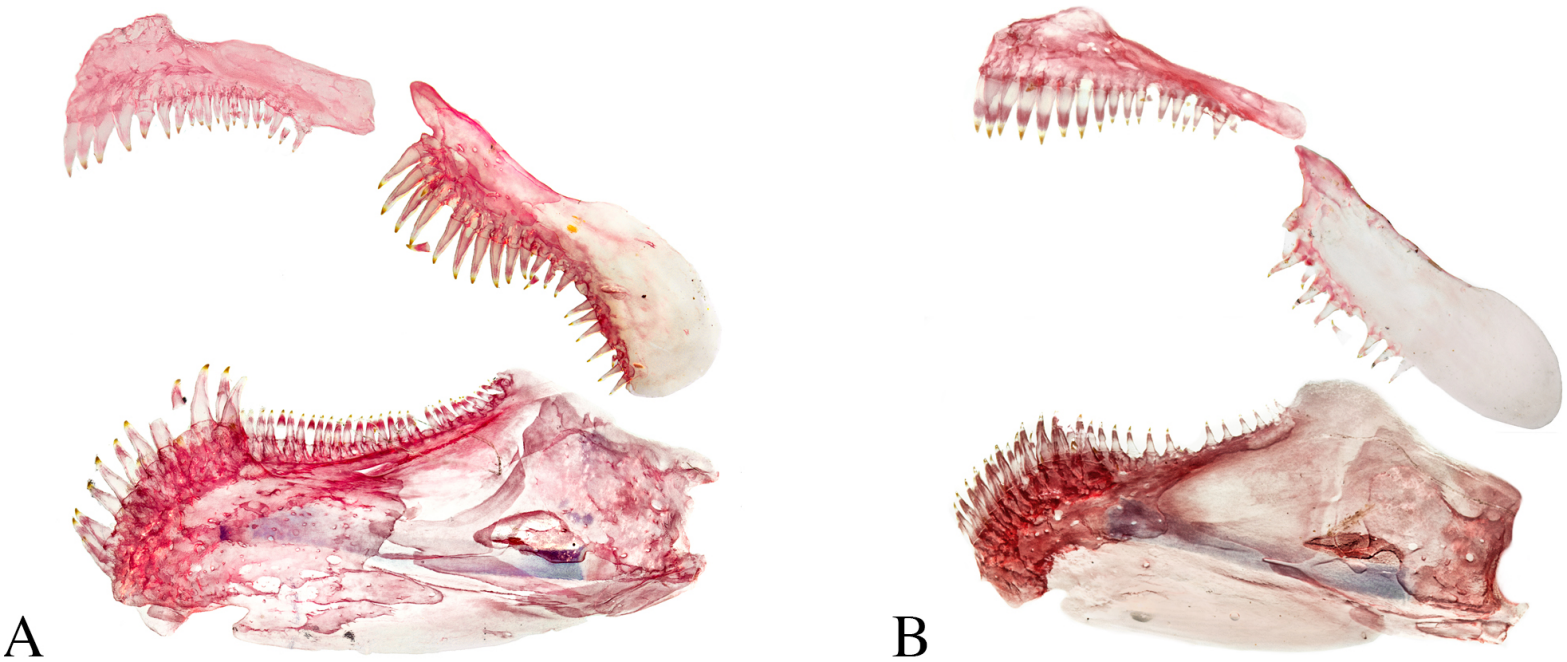
Jaws of *Copella eigenmanni*. (A) MZUSP 81143, male, 44.3 mm SL, lateral view of left side, and (B) MZUSP 81143, female, 29.2 mm SL, lateral view of right side, inverted.

**Fig 2:**
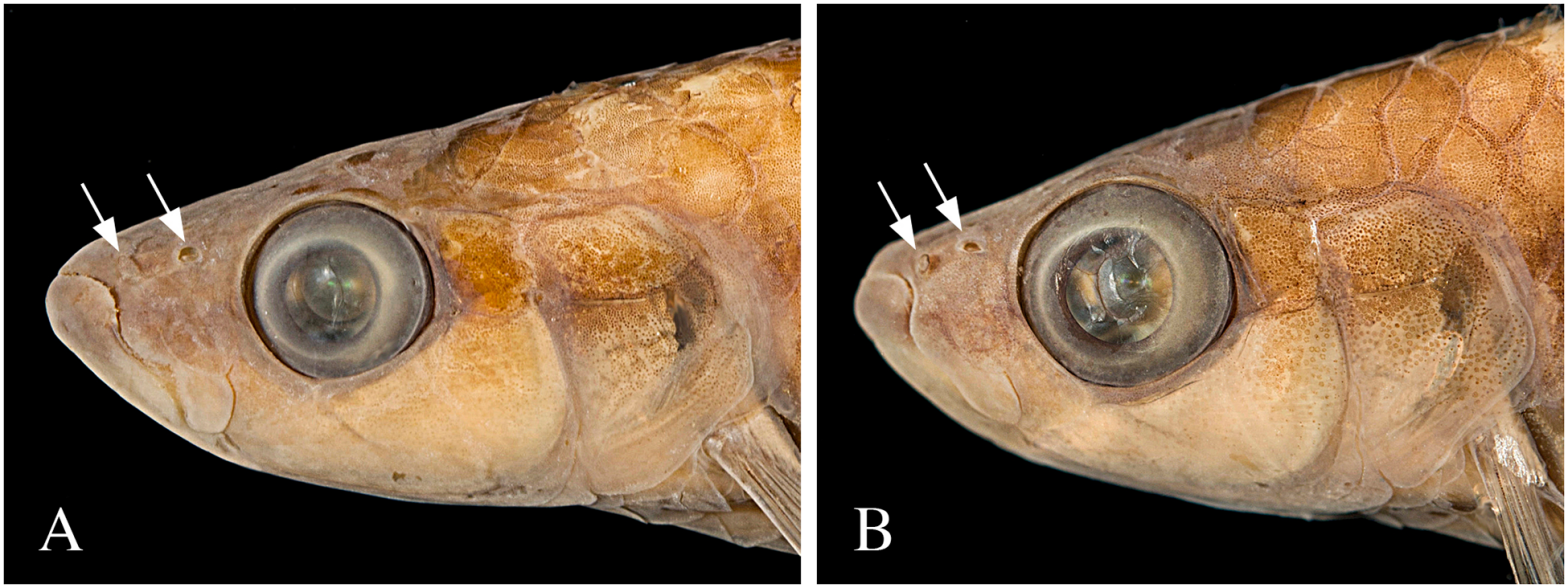
Lateral view of head of *Copella compta*. MZUSP 9162 (A) male, 68.9 mm SL, (B) female, 40.3 mm SL. Arrows show anterior and posterior nares. Also note differences in the curvature of the anterior border of maxilla between male and female.

**Diagnosis.** *Copella* can be easily distinguished from other genera of the Lebiasinidae by having the anterior portion of the maxilla triple curved in males (Figs 1A and 2A) (*vs*. approximately straight or convex, Fig 2A and 2B), and by males being distinctly longer than females (*vs*. males and females of about the same size). Additionally, *Copella* is distinguished from other genera of the family, except *Nannostomus*, by having anterior and posterior nares distant from each other (Fig 2) (*vs*. juxtaposed, close to each other). It is distinguished from *Nannostomus* by having a black spot on the dorsal fin (*vs*. dorsal fin hyaline), elongate fins, especially on males (*vs*. fins not elongate), and upturned mouth (*vs*. terminal).

**Fig 3:**
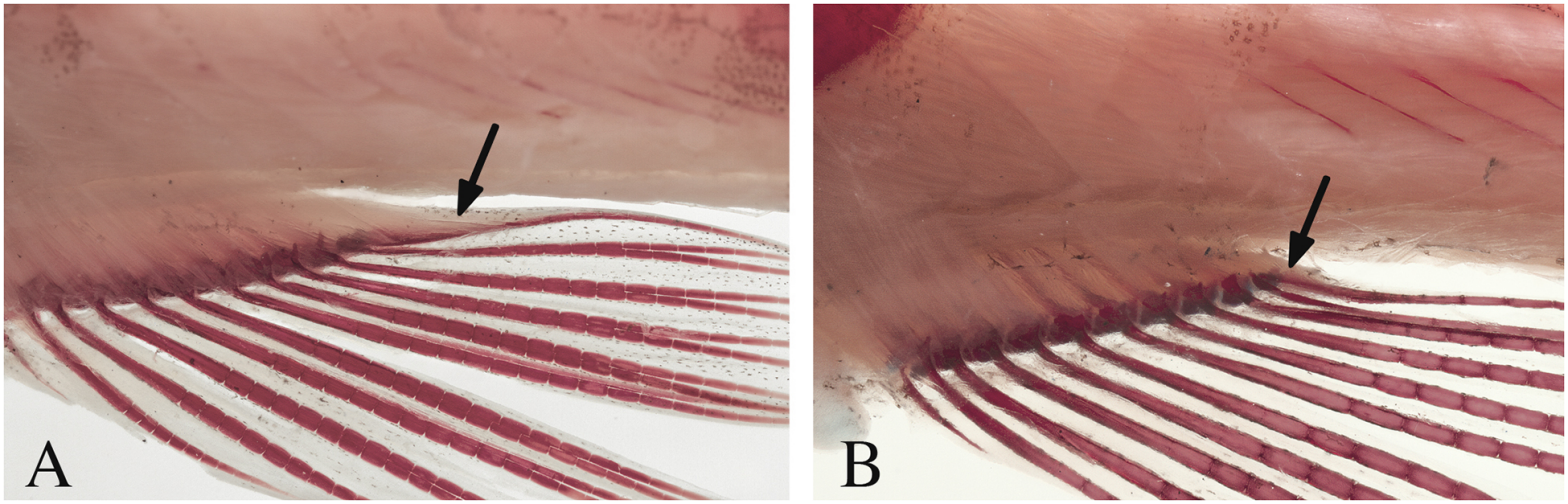
Anal fin of *Copella arnoldi*. Arrows show the anal-fin inclinator muscle of MZUSP 105770 (A) a male, 33.7 mm SL, and (B) a female, 21.7 mm SL.

**Remarks on sexual dimorphism in the genus.** Species of *Copella* are extremely sexually dimorphic and some of the dimorphic features are common to all of them. In all species, males are distinctly longer than females, with elongate fins. Males have the anterior border of the maxilla triple curved (“S” shaped”), bearing more teeth than females (Figs 1A and 2A), whereas in females it is approximately straight with fewer teeth (Figs 1B and 2B). These features are unique among lebiasinids and possibly represent synapomorphies to the genus. *Copella* also has similar pattern of external sexually dimorphic features found in other lebiasinids concerning modifications on the anal fin. The rays are anteroposteriorly thickened and longer in males than in females [14], having thickened membranes, and well-developed erector and depressor muscles [16] (Fig 3). Some species of *Copella* have sexually dimorphic coloration. Other sexual dimorphic features are described in the “Sexual dimorphism” section under species descriptions.

**Distribution.** Species of *Copella* occur in the rio Amazonas basin in Brazil, Colombia, Guyana, Peru, and Venezuela, the Orinoco basin in Colombia and Venezuela, and coastal drainages of Guyana, French Guiana, Surinam, and Venezuela.

**Ecological notes.** *Copella* mostly inhabits still to slow-flowing forest streams and minor tributaries or flooded forest in the wet season. We have also collected specimens in small marginal ponds, poorly connected to main streams, along with species belonging to the family Rivulidae. They are most often found in moderately acid black water, frequently associated with macrophytes and partially submerged plants, where they find food and protection. The species are usually seen in the water surface along the shoreline of small streams, eating allochthonous food and small insects with their upturned mouth [5]. *Copella callolepi*s and *C*. *nattereri* are known to deposit eggs on submerged vegetation, which are guarded by the male during incubation. *Copella arnoldi* presents a very uncommon reproductive behavior commented under Remarks on this species.

#### Key to the species of Copella

1a. Procurrent caudal-fin rays hyaline; darkly pigmented area extending from posteroventral portion of dentary to ventral portion of eye; brilliant white spots usually present on body scales, in live or preserved male specimens……………….***Copella arnoldi*** (lower rio Amazonas basin, coastal drainages of Guyana, French Guiana, Surinam, and mouth of rio Orinoco).

1b. Procurrent caudal-fin rays black (except in some populations of *C*. *nattereri* from the rio Negro); no dark pigmentation in the area extending from posteroventral portion of dentary to ventral portion of eye; white spots on body scales sometimes present in preserved species, spots red in life………………………………………2

2a. Middle caudal-fin rays dark……………………………………………***Copella eigenmanni*** (rio Orinoco basin, upper rio Negro and upper rio Putumayo, rio Amazonas basin).

2b. Middle caudal-fin rays hyaline………………………………….3

3a. 15–19 predorsal scales; first longitudinal scale row with 14–18 scales; fourth longitudinal scale row with 24–28 scales; clear spots (red in life) absent on posterior portion of body scales..………………………………………………….. 4

3b. 12–14 predorsal scales; first longitudinal scale row with 11–14 scales; fourth longitudinal scale row with 20–24 scales; clear/white spots (red in life) present on posterior portion of body scales..………….. ……………………………………………5

4a. Males with rows of conspicuous dark scales irregularly disposed on body, gradually lighter posteriorly; females and juveniles with brownish inconspicuous wide stripe on flank……………………………..***Copella vilmae*** (upper rio Amazonas, surroundings of Letícia, Colombia).

4b. Males lacking dark scales irregularly disposed on body, bearing a faint longitudinal dark stripe on flank; females with a plain coloration, without longitudinal dark stripe on body…………………………………………………….***Copella compta*** (upper rio Negro upstream São Gabriel da Cachoeira, Brazil and Venezuela).

5a. Clear/white spots (red in life) on posterior portion of body scales, limited dorsally, posteriorly and ventrally by dark pigmentation which is frequently horseshoe shaped; longitudinal dark stripe, when present, formed by subjacent pigmentation……………………………………***Copella nattereri*** (rio Amazonas from Letícia, Colombia, to mouth of rio Tapajós, rio Negro basin and upper and middle rio Orinoco basin).

5b. Clear/white spots (red in life) frequently restricted to fourth longitudinal scale row of body, not limited by dark pigmentation; longitudinal black stripe conspicuous, formed by superficial pigmentation, located below the row of clear spots…………………………***Copella callolepis*** (rio Amazonas, rio Madeira, and coastal drainages of Pará State at Brazil).

### *Copella arnoldi* (Regan, 1912)

Figs 3–10; Tables 1 and 2

**Fig 4:**
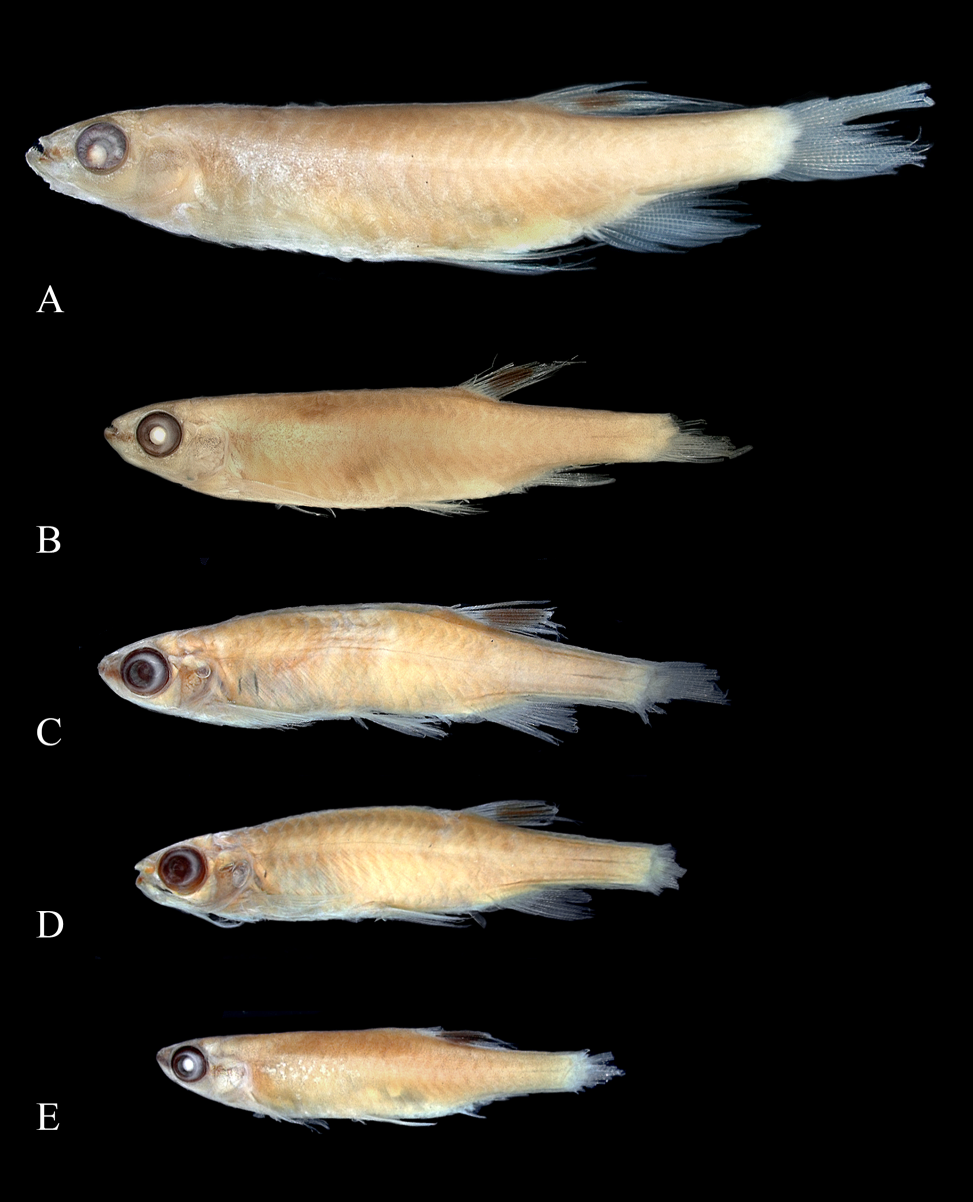
Syntypes of *Copeina arnoldi*, BMNH 1909.4.2.25–26. (A) male, 34.4 mm SL, (B) female, 25.4 mm SL, Amazon, Brazil and of *Copeina carsevennensis*, BMNH 1899.7.26.1–5, immatures, (C) 24.3 mm SL, (D) 22.9 mm SL, and (E) 18.5 mm SL, Carsevenne, French Guiana (= rio Calçoene, Amapá, Brazil).

**Fig 5:**
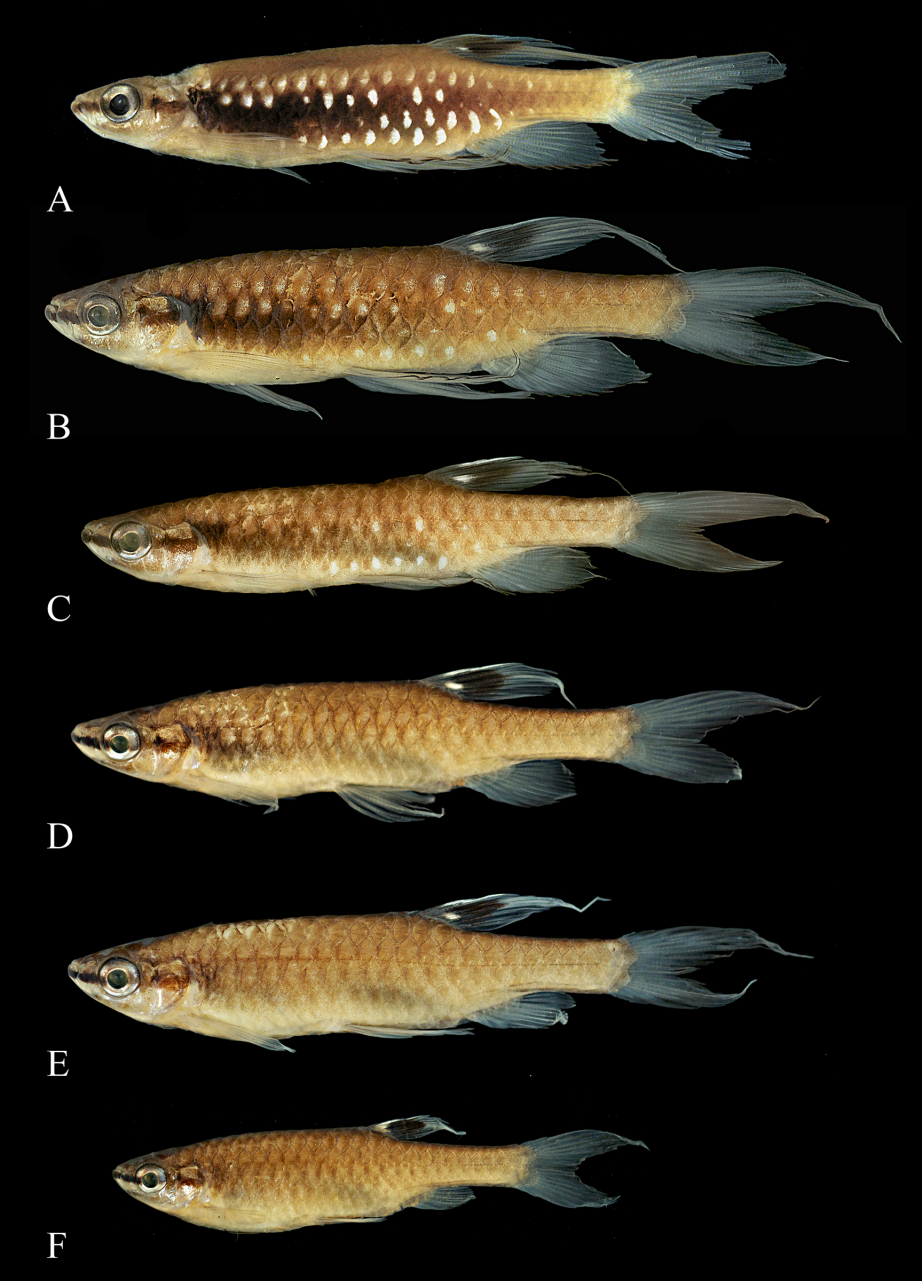
*Copella arnoldi*. (A) MPEG 23064, male, 33.2 mm SL, Marapanim, Pará, Brazil; MZUSP 105770, (B) male, 38.2 mm SL, flipped horizontally, (C) male, 33.6 mm SL, (D) male, 33.7 mm SL, male, (E) male, 33.5 mm SL, (F) female, 30.9 mm SL, Vigia, Pará, Brazil.

**Fig 6:**
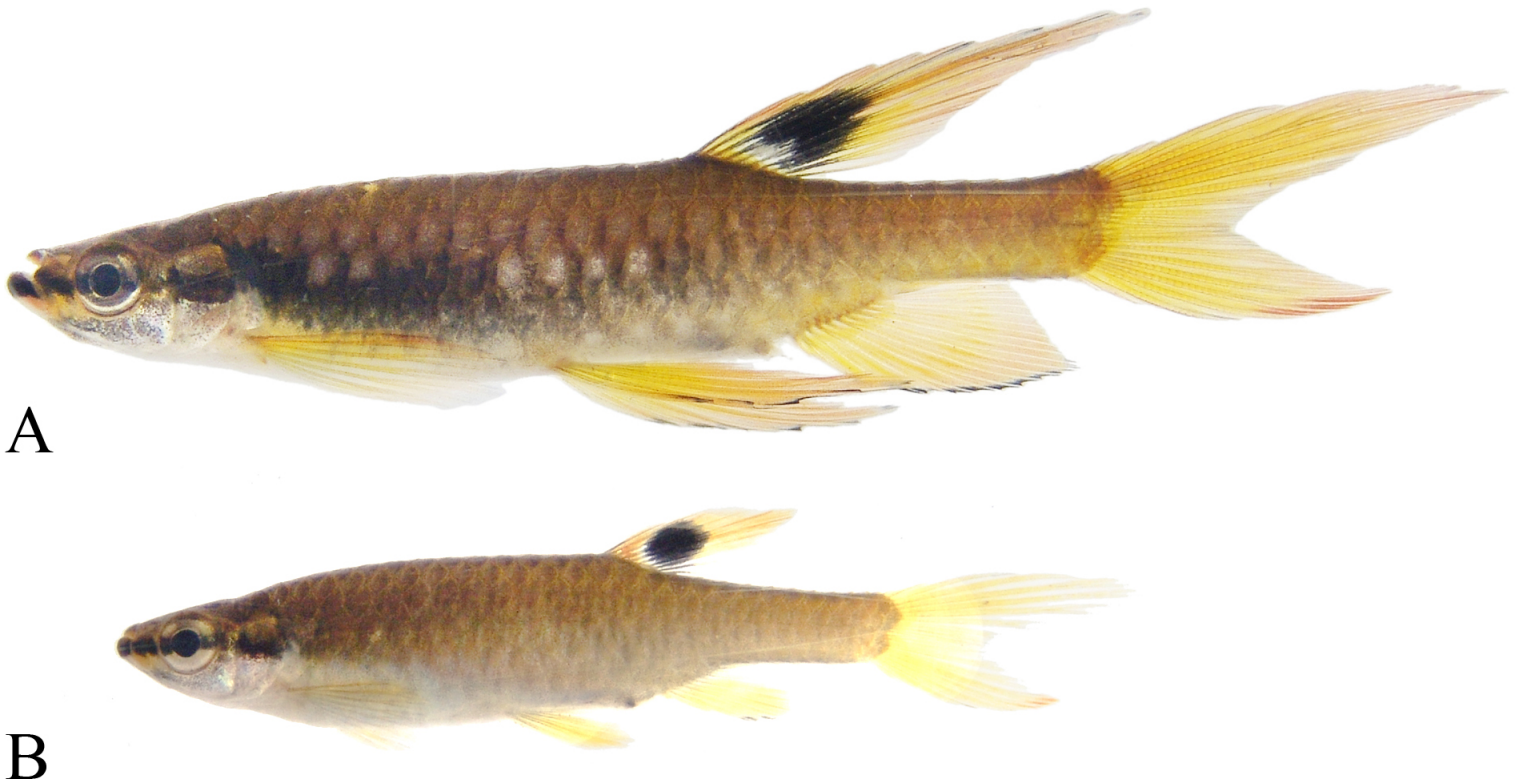
*Copella arnoldi*, live specimens. MZUSP 105770, (A) male, 39.9 mm SL, (B) female, 27.9 mm SL, Vigia, Pará, Brazil.

**Fig 7:**
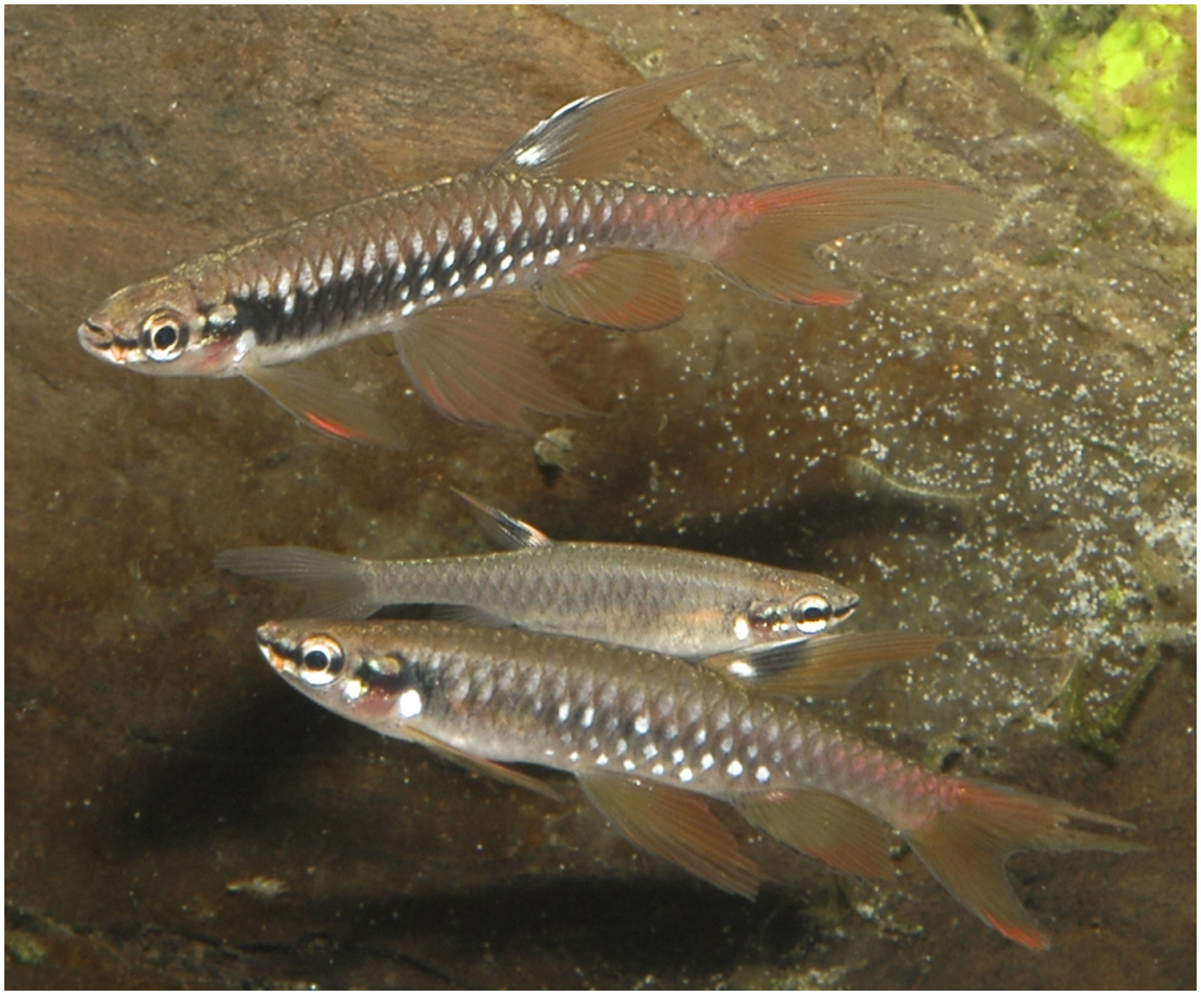
*Copella arnoldi* live specimen not preserved. Males above and below, female in the middle, Vitória do Xingú, Pará, Brazil. Reprinted under a CC BY license, with permission from Hans-George Evers, original copyright 2017.

**Fig 8:**
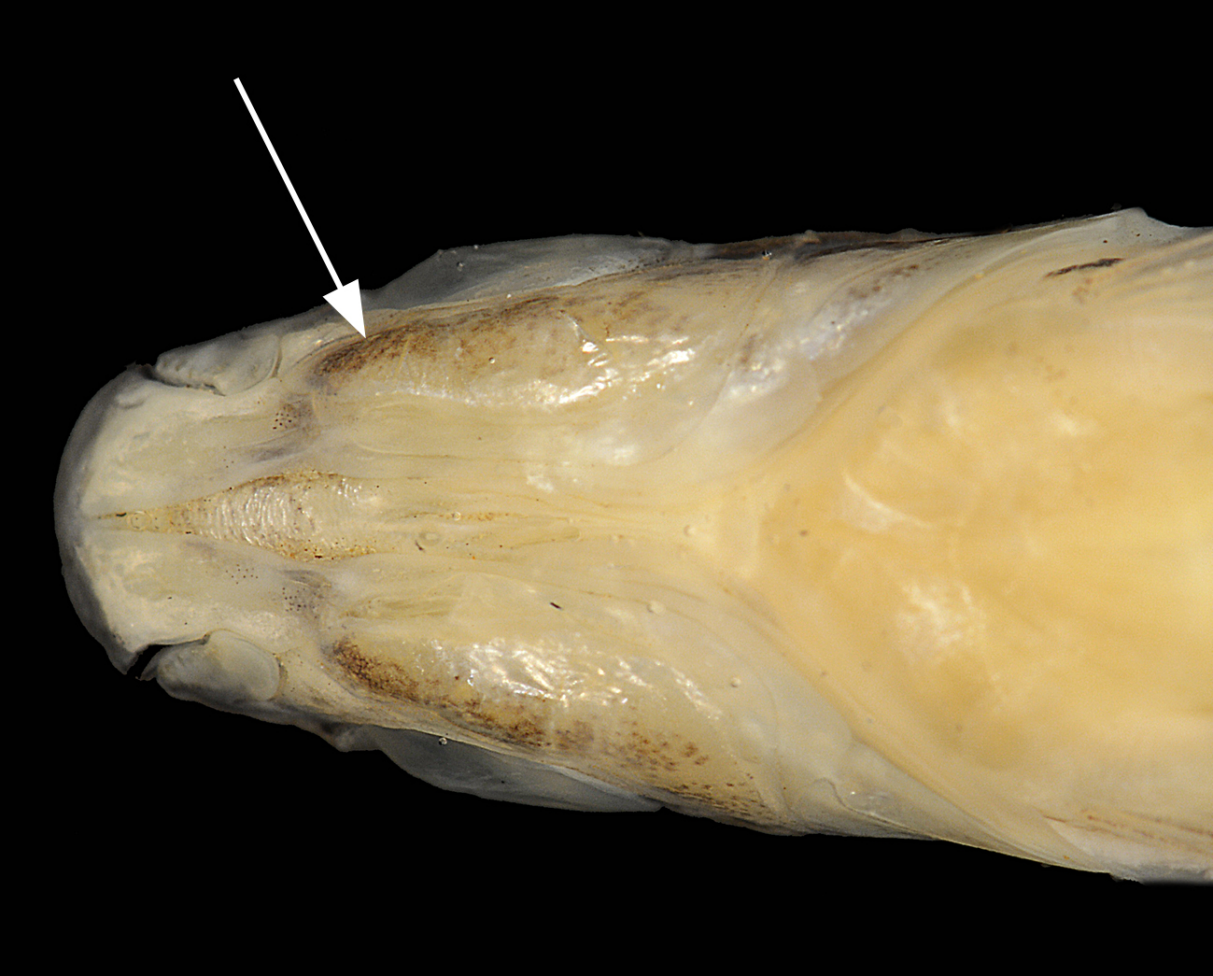
Ventral view of head of *Copella arnoldi*. MZUSP 105770, 35.3 mm SL showing characteristic pigmentation below eye.

**Fig 9:**
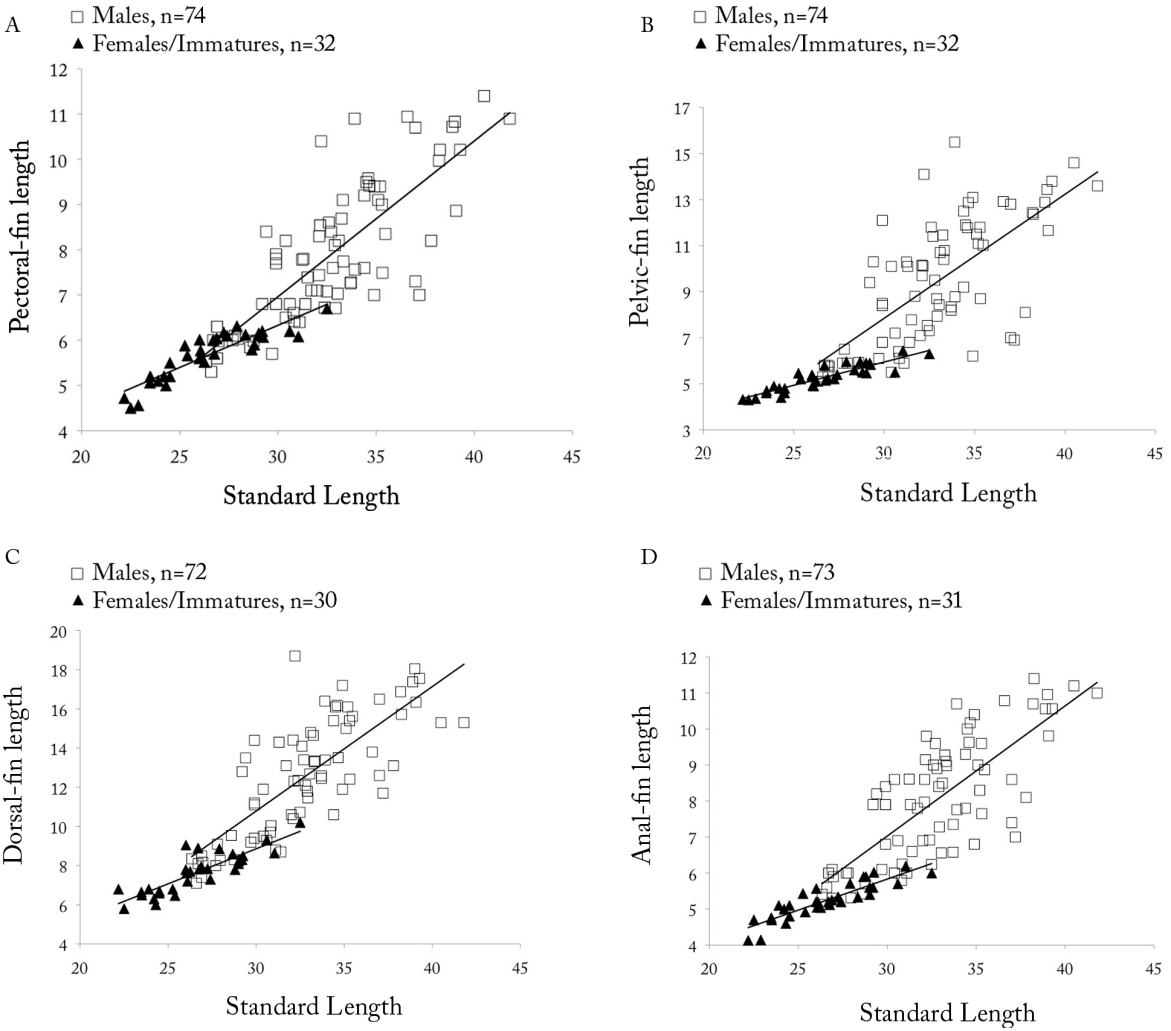
*Copella arnoldi*. (A) pectoral-, (B) pelvic-, (C) dorsal-, and (D) anal-fin lengths as a function of SL by sex.

**Fig 10:**
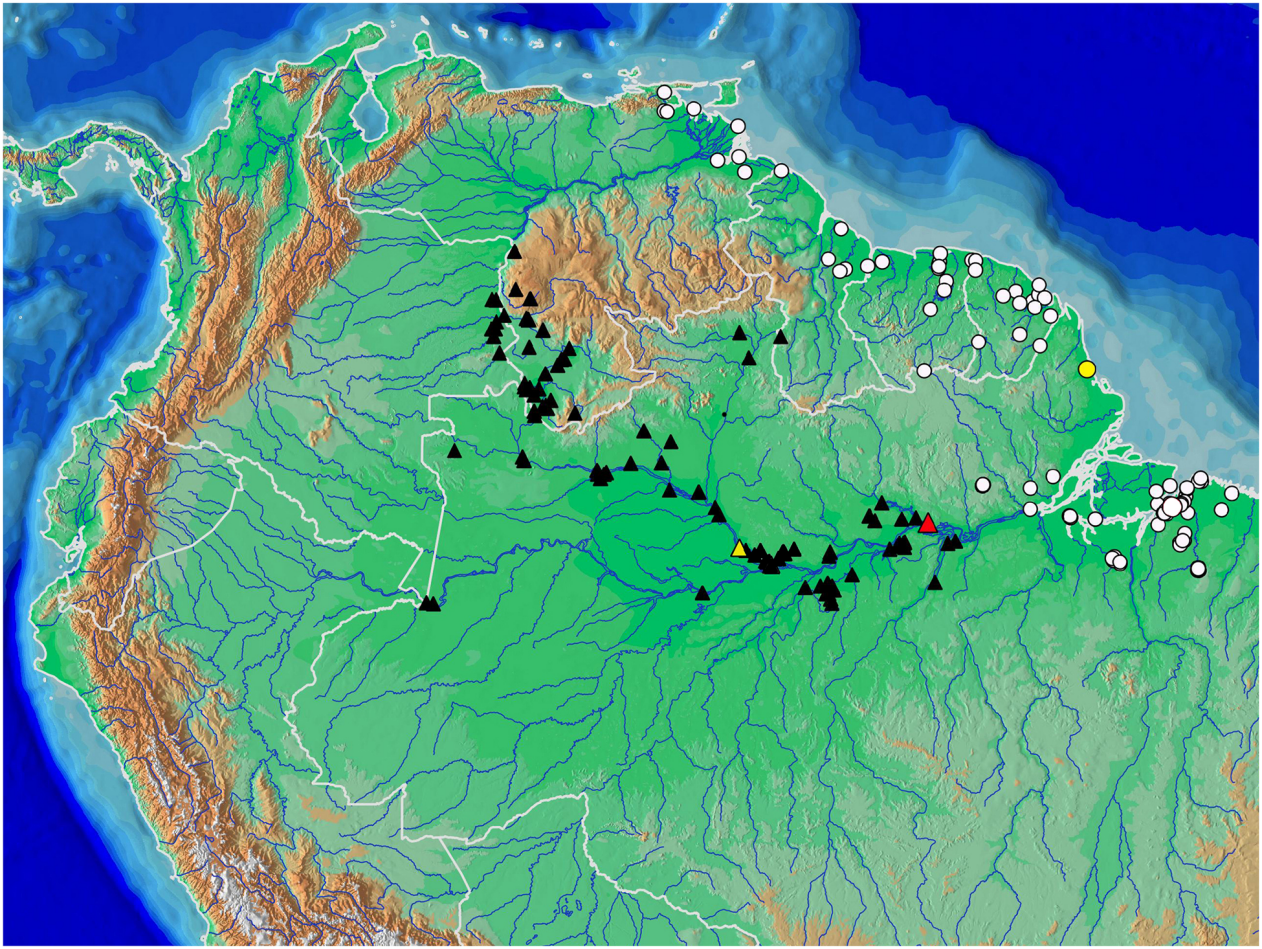
Distribution of *Copella arnoldi* (white circles) and of *C*. *nattereri* (black triangles); type localities of *Copeina carsevennensis* (yellow circle), *C*. *nattereri* (red, triangle), and *C*. *meinkeni* (yellow triangle). Some symbols may represent more than one locality or lot of specimens. Type localities of *C*. *arnoldi* and *C*. *callolepis* are “lower Amazon basin” and “Amazon”, respectively.

**Table 1:** Morphometrics of *Copella arnoldi*.

	*Copeina arnoldi*			*Copeina carsevennensis*			*Copella eigenmanni*			Non-type material					
	Syntypes			Syntypes			Paralectotypes			n	Range			Mean	SD
Standard length (mm)	25.4	and	34.4	22.9	and	24.3	19.5	and	20.9	104	22.2	-	41.8	31.1	
**Percents of standard length**															
Body depth	20.1	and	19.5	19.8	and	19.6	19.7	and	19.7	104	16.8	-	23.2	19.8	1.1
Dorsal- to caudal-fin origin	37.9	and	34.2	40.5	and	37.7	43.6	and	37.6	104	33.7	-	40.3	37.5	1.2
Snout to dorsal-fin origin	62.4	and	64.5	59.9	and	64.0	61.9	and	62.1	104	60.4	-	66.0	63.2	1.3
Snout to pectoral-fin origin	22.4	and	22.0	22.7	and	22.0	23.9	and	22.9	104	19.9	-	25.8	22.3	1.1
Snout to pelvic-fin origin	51.5	and	50.3	46.2	and	45.4	43.8	and	49.4	104	43.5	-	52.5	48.4	1.5
Snout to anal-fin origin	74.2	and	73.2	68.7	and	69.3	70.6	and	73.5	104	67.6	-	75.1	71.2	1.6
Pectoral- to pelvic-fin origin	29.9	and	30.3	25.3	and	23.8	22.7	and	26.8	104	23.0	-	30.4	27.3	1.5
Pelvic- to anal-fin origin	23.4	and	23.7	23.4	and	21.6	23.1	and	25.6	104	20.8	-	29.5	23.9	1.4
Pectoral-fin length males	-		22.2	-		-	-		20.6	74	18.9	-	32.3	24.1	3.1
Pectoral-fin length fem/imm	22.3		-	19.9	and	20.5	20.7		20.8	30	19.6	-	23.3	21.4	0.9
Pelvic-fin length males	-		26.7	-		-	-		-	74	17.8	-	32.3	24.1	3.1
Pelvic-fin length fem/imm	20.5		-	19.0	and	18.3	19.7		-	30	17.8	-	21.7	19.8	0.9
Dorsal-fin length males	-		30.8	-		-	-		-	72	26.5	-	58.0	38.1	6.6
Dorsal-fin length fem/imm	25.5		-	-		24.6	-		27.2	29	25.6	-	33.4	28.7	1.7
Anal-fin length males	-		22.7	-		-	-		-	73	18.7	-	31.6	24.1	3.1
Anal-fin length fem/imm	19.4		-	18.1	and	19.0	19.7		-	30	18.5	-	21.5	19.8	0.9
Anal-fin base length	9.2	and	9.2	8.0	and	9.4	10.1	and	8.6	104	7.7	-	12.6	10.0	1.0
Caudal peduncle depth	9.1	and	9.3	8.4	and	9.0	8.8	and	8.8	104	7.6	-	10.2	9.1	0.6
Caudal peduncle length	20.0	and	17.5	21.5	and	21.5	22.7	and	18.3	104	16.3	-	22.4	19.7	1.3
Head length	22.1	and	21.8	23.5	and	23.0	24.3	and	25.2	103	20.3	-	25.9	22.9	1.1
**Percents of head length**															
Eye diameter	38.8	and	31.8	39.2	and	39.1	39.7	and	33.1	102	29.0	-	40.3	35.3	2.5
Snout length	25.5	and	27.3	24.2	and	27.4	22.8	and	25.9	103	22.9	-	34.8	27.6	2.0
Interorbital distance	38.8	and	37.7	36.4	and	36.2	39.2	and	37.6	103	33.1	-	42.3	37.6	1.9
Upper jaw length	28.4	and	27.2	29.9	and	30.6	27.0	and	20.7	103	25.0	-	35.2	30.2	2.1

Syntypes of *Copeina arnoldi* BMNH 109.4.2.25–26 (2), syntypes of *Copeina carsevennensis* BMNH 1899.7.26.1–5 (2), paralectotypes of *Copella eigenmanni* BMNH 1911.10.31.140 (2) and non-type material DZSJRP 11120 (2), DZSJRP 11231 (8), MHNLS 12458 (3), MHNG 2200.34 (4), MHNG 2200.36 (6), MHNG 2647.005 (6), MNHN 1898.0053 (3), MPEG 8223 (2), MPEG 8305 (6), MPEG 10398 (5), MPEG 10716 (4), MZUSP 23064 (20), MZUSP 105770 (14), ZMA 101.937 (5), ZMA 104.197 (2), ZMA 104.288 (4), ZMA 105.694 (4), ZMA 106.105 (4), and ZMA 106.137 (2). n = number of specimens. SD = Standard deviation. Range does not include primary types.

**Table 2:** Meristics of syntypes of *Copeina arnoldi* BMNH 109.4.2.25–26 (2), syntypes of *Copeina carsevennensis* BMNH 1899.7.26.1–5 (2), and paralectotypes of *Copella eigenmanni* BMNH 1911.10.31.140 (2).

	*Copeina arnoldi*			*Copeina carsevennensis*			*Copella eigenmanni*		
	Syntypes			Syntypes			Paralectotypes		
Dorsal-fin rays	ii8	and	ii8	ii8	and	ii8	ii8	and	-
Pectoral-fin rays	i8	and	i8	i8	and	i9	i9	and	i8
Pelvic-fin rays	i6	and	i7	i7	and	i7	i7	and	i7
Anal-fin rays	iii9		-	iii9	and	iii9	iii9	and	iii9
Caudal-fin rays	i8,7i		-	-		-	-		-
Predorsal scales	13	and	14	15	and	15	14	and	14
First longitudinal scale row	13	and	14	15	and	13	13	and	14
Fourth longitudinal scale row	-		23	25	and	25	24	and	23
Longitudinal scale rows dorsal to pelvic	5	and	5	6	and	5	6	and	6
Longitudinal scale rows dorsal to anal	5	and	5	5	and	5	5	and	5
Circumpeduncular scale rows	10	and	10	10	and	10	10	and	10
Total vertebrae	37		-	37	and	37	-		-

*Pyrrhulina filamentosa*.—Eigenmann & Eigenmann [17]: 110 [possibly not *Pyrrhulina filamentosa*].—Eigenmann [18]: 104 [in part, from rio Demerara at Kumaka, rios Lama, and Aruka, Guyana].—Magalhães [19]: 179, fig 95 [misidentification; brief description; breeding behavior].

*Copeina arnoldi* Regan [20]: 393 [type locality: Amazon (= lower Amazon basin)].—Myers [21]: 111 [comparison with *Copeina* (= *Copella*) *compta*].—Fowler [22]: 344 [listed; *Copeina callolepis*, *C*. *eigenmanni*, and *C*. *carsevennensis* considered as synonyms].—Boeseman [23]: 184 [Maroni basin, Surinam; listed].—Meinken [24]: 116 [comparison with *Pyrrhulina nigrofasciata* (= *Copella callolepis*)].—Boeseman, [25]: 13 [rio Surinam basin; listed].—Boeseman, [26]: 18 [rio Paramaribo basin, Surinam; listed].—Boeseman, [27]: 186 [literature compilation].—Myers [4]: 13 [comparison with *Holotaxis melanostomus* (= *Pyrrhulina melanostoma*)].—Krekorian & Dunham, 1972 [28], [29] [breeding behavior].—Krekorian & Dunham [30] [breeding behavior].—Krekorian [31] [breeding behavior].

*Copeina eigenmanni*.—Regan [20]: 393 [in part, from Pará (Brazil), rios Aruka and Lama (Guyana)].—Fowler [22]: 344 [literature compilation; in part, from Pará and Guyana, considered as synonym of *Copeina* (= *Copella*) *arnoldi*].

*Copeina carsevennensis* Regan [20]: 394 [type locality: Carsevenne, French Guiana (= rio Calçoene, Amapá, Brazil)].—Myers [21]: 111 [comparison with *Copeina* (= *Copella*) *compta*].—Fowler [22]: 344 [literature compilation; synonymous with *Copeina* (= *Copella*) *arnoldi*].

*Copella arnoldi*.—Géry [5]: 143 [new combination; brief description; figure as “*Copella* of the arnoldi-group”].—Planquette *et al*. [32]: 178 [rio Maroni, French Guiana; brief description; unnumbered fourth figure pg. 179].—Weitzman & Weitzman [1]: 241 [literature compilation, introduced in Trinidad & Tobago].—Keith *et al*. [33]: 30 [Litany drainage, French Guiana; cited].—Zarske & Géry [7]: 44 [identification key].—Oyakawa & Netto-Ferreira [15]: 64 [literature compilation].—Montag *et al*., [34]: 245 [Ilha do Marajó; listed].—Zarske [35]: 14 figs 12–16, 18–19, 34–35 [rio Xingu; redescription; photo of syntype from BMNH 1909.4.2.23–26; taxonomic notes].—Mol *et al*. [36] [rios Corantijn, Saramacca, Commewijne, Marowijne; listed].

*Copella eigenmanni*.—Géry [5]: 147 [new combination; specimens from Guyana and Pará; possibly same species as *C*. *metae*].—Vari [14]: 5 [material used in phylogenetic analysis].—Weitzman & Weitzman [1]: 242 [mouth of rio Orinoco; literature compilation; comments on type locality].—Zarske & Géry [7]: 44 [identification key].—Oyakawa & Netto-Ferreira [15]: 64 [literature compilation; incomplete information about type locality].—Mol *et al*. [36] [synonym of *Copella arnoldi*].

*Copella carsevennensis*.—Géry [37]: 120 [new combination; Mooi Wana and foot of Albina-hills, Suriname; listed].—Géry [5]: 146 [literature compilation; unnumbered figure pg. 146, left].—Planquette *et al*. [32]: 178 [rio Maroni, rio Mana, rio Sinnamary, rio Kourou, rio Comté, rio Kaw, rio Aprouaggue, and rio Oiapoque, French Guiana; brief description; unnumbered first fig pg. 179].—Keith *et al*. [33]: 30 [Litany drainage, French Guiana; cited].—Mérigoux *et al*., [38]: 30 [rios Malmanouri and Karouabo, French Guiana; listed].—Weitzman & Weitzman [1]: 241 [literature compilation].—Zarske & Géry [7]: 44 [identification key].—Oyakawa & Netto-Ferreira [15]: 64 [literature compilation; comments on type locality].—Zarske [35]: 32, figs 25–30 [redescription; picture of 3 syntypes from BMNH 1911.10.31.140; syntypes of *Copeina eigenmanni* from Guyana possibly conspecific with *Copella carsevennensis*; taxonomic notes].—Mol *et al*. [36] [synonym of *Copella arnoldi*].

*Copella* spec. aff. *arnoldi*.—Zarske & Géry [7]: 44 [identification key].

*Copella* sp.—Montag *et al*. [39]: 18 [FLONA de Caxiuanã in igarapé; listed].

*Copella nattereri*.—Montag *et al*., [34]: 245 [misidentification; listed].

*Copella metae*.—Lasso & Sánchez-Duarte [40]: 64 [misidentification; listed].

**Diagnosis.** *Copella arnoldi* can be distinguished from all congeners, except some specimens of *C*. *nattereri*, by having the procurrent caudal-fin rays hyaline (*vs*. black). It can be distinguished from *C*. *nattereri* by the absence of a black mark on each body scale (*vs*. presence). Additionally, it is distinguished from all congeners by having a pigmented area extending anterodorsally from ventral tip of the dentary to ventral portion of the eye (*vs*. absent). Some males of *Copella arnoldi* are unique among congeners in having brilliant white spots on scales of the third, fourth, fifth, and sixth longitudinal scale rows.

**Description.** Morphometrics in Table 1, meristics of types in Table 2. Largest examined male 42.3 mm SL, female 32.5 mm SL. Greatest body depth slightly anterior to vertical through pelvic-fin origin. Body cylindrical, slightly compressed laterally. Dorsal profile of body straight or slightly convex from tip of snout to end of supraoccipital, straight or slightly convex from that point to dorsal-fin origin, posteroventrally inclined along dorsal-fin base and straight along caudal peduncle. Ventral profile of body convex from anterior tip of dentary to vertical through anterior margin of orbit, straight from that point to vertical through pectoral-fin origin, slightly convex from that point to pelvic-fin origin, straight from pelvic-fin origin to anal-fin origin, posterodorsally inclined along anal-fin base and straight along caudal peduncle.

Mouth upturned. Premaxillary teeth in one row, with 15 (2), 18 (2), or 19 (1) teeth, decreasing in size laterally. Number of maxillary teeth sexually dimorphic, 9 (1), 10 (1), or 12 (1) in males, 3 (1) or 5 (1) in females, decreasing in size posteriorly, especially in males. Dentary teeth in two rows, outer with 8 (2), 10 (1), 11(1), or 12 (1) teeth, increasing in size laterally, inner with 24 (2), 25 (1), 28 (1), or 30 (1) teeth, decreasing in size laterally.

Dorsal fin with ii, 8* (109) rays, second and third branched rays longer. Pectoral fin with i (101), 8* (10), 9 (65), 10 (24), or 11 (2) rays, first three branched rays longer. Pelvic fin with i (102), 6 (1), 7* (99), or 8 (2) rays, third branched ray longest. Anal fin with iii (6), 7,i (2) or 8,i* (99), rays, fourth and fifth branched rays longer. Adipose fin absent. Caudal fin with i (94), 7 (4), 8* (88), or 9 (2) rays in upper lobe, first and second branched rays longer, and 5 (1), 6 (3), or 7* (58), i (62) rays in lower lobe, first and second branched rays longer. Upper caudal-fin lobe longer than lower. Relative fin lengths variable among males, specimens of same size with distinct fin lengths (Fig 9), but among males from same lot, longer males tend to have longer fins and be more intensively colored than other males. This may be related to hierarchical position within shoal.

Predorsal scales 13* (23), 14* (54), or 15 (20), in one series. First longitudinal scale row with 12 (8), 13* (30), 14* (52), or 15 (7) scales. Fourth longitudinal scale row with 23* (42), 24 (38), 25 (14), or 26 (2) scales. Lateral line not pored, first lateral line scale with small canal medially on its anterior portion, without lateral opening. Longitudinal scale rows between dorsal-fin origin and pelvic-fin origin 5* (21) or 6 (75). Longitudinal scale row between dorsal-fin origin and anal-fin origin 5* (106). Circumpeduncular scale rows 10* (106). Total number of vertebrae 34 (1), 35 (19), 36 (17), or 37* (5).

**Color in alcohol.** Overall ground coloration of body beige. Dark stripe extending from anterior tip of dentary to posterior tip of opercle (Figs 5 to 7). Dark pigmentation extending anterodorsally from posteroventral portion of dentary to ventral portion of eye (Fig 8). Thin predorsal dark stripe, frequently wider over second and third scales. Faint dark pigmentation at base and at posterior border of body scales (Fig 5). Ventral region clear. Small dark blotch behind opercle on males and females. Males with blur dark stripe of variable extension and intensity, extending from opercle to, at most, vertical through end of anal-fin base (Figs 5A–5E, 6A, 7). Dorsal fin with black round spot above smaller white one. Remaining fins hyaline. Pelvic and anal fins usually with dark edge, more intense in males. Some specimens from Surinam and mouth of rio Orinoco with distal portion of first pelvic-fin ray conspicuously black. Some males with brilliant white spots on scales of third, fourth, fifth, and sixth longitudinal scale rows, mainly restricted to median portion of body (Figs 5A to 5C, 6A and 7). Males with very long dorsal fin bearing elongated black spot extending to fin tip.

**Color in life.** Dark stripe extending from anterior tip of dentary to posterior tip of opercle. Upper and lower jaws yellow to pale red. Overall body coloration light brown or beige, ventral portion clear. Fins yellow to orange. Dorsal fin with black spot dorsal to small, white round spot. Some males with brilliant white spots on scales of third, fourth, fifth, and sixth longitudinal scale rows, mainly restricted to median portion of body. Some males with dark stripe extending from opercle to, at most, vertical through end of anal-fin base, of variable intensity. Males with base of dorsalmost rays of upper caudal-fin lobe, and tip of basalmost rays of lower caudal-fin intense red (Figs 6 and 7).

**Sexual dimorphism.** Males with more numerous maxillary teeth than females (see description above). Pectoral, pelvic, dorsal, and anal fins distinctly longer in males than in females (Fig 9). Tip of pectoral fin sometimes extending beyond pelvic-fin origin in males, but never to that point in females. Tip of adpressed pelvic fin reaching to two-thirds length of caudal peduncle in males, but only to level of anus in females. Tip of adpressed dorsal fin reaching to one-half length of median caudal-fin rays in males, and approximately to one-half length of caudal peduncle in females. Tip of adpressed anal-fin reaching to level of first ventral procurrent rays in males, and to two-thirds length of caudal peduncle in females. Upper caudal-fin lobe longer than lower, especially in males. Sexually dimorphic color pattern described in “Color in alcohol” section.

**Distribution.** Lower rio Amazonas basin, coastal drainages of Pará and Amapá, Brazil, Guyana, French Guiana, Surinam, mouth of rio Orinoco, and coastal drainages of Sucre and Monagas, Venezuela (Fig 10).

**Behavioral notes.** *Copella arnoldi* is widely known among aquarists by its unique breeding behavior and parental care (see Krekorian & Dunham [28], [29], [30], Krekorian [31]). The male and the female line up side by side at the surface of the water and jump together out of the water, to spawn. Fertilized eggs are laid on the underside of an emergent leaf and the male then splashes them with its tail for about three days until they hatch, hence the popular name “Splash tetra”.

Eight individuals of *Copella arnoldi*, five males and three females (MZUSP 105770), were kept and observed in aquarium. Territoriality was performed by two males, that usually chased other males and displayed parallel to each other, opening their fins and mouths widely. These two males were slightly larger than the others and had extremely long fins. They were more colorful, with more brilliant white spots, and a darker black stripe on body, compared to the other males. The remaining males were smaller, without exuberant coloration, some of them with color similar to the females. Only one of the largest males had access to the mature female, whose abdomen was orange and full of ovocytes. These observations bring the question of whether the differences in coloration among males of *Copella arnoldi* in the same shoal are somehow related to the hierarchical position in the group. However, this deserves further studies.

Just before lining up to jump out of the water toward the upper glass of the aquarium, the female follows the male, touching her abdomen at the anterodorsal portion of the male several times, swimming agitatedly. This seems to be the same behavior described by Zarske ([35]; fig 30), but interpreted as an attempt of the female to push the male down to the bottom. He proposed such breeding behavior as one of the differences between *Copella arnoldi* and *C*. *carsevennensis*. However, as he pointed out, the couple never spawned to confirm his hypothesis.

**Remarks.** Regan [20] described *Copella arnoldi* (rio Amazonas) and *C*. *carsevennensis* (Carsevenne, French Guiana = rio Calçoene, coastal drainage of Amapá, Brazil) based on the position of the dorsal fin that would distinguish *C*. *arnoldi* by having “the dorsal-fin origin nearer to base of caudal than to head” (*vs*. “origin of dorsal fin equidistant from head and base of caudal, or a little nearer head”). Géry [5] diagnosed both species also by the number of longitudinal scales (23–24 *vs*. 26) and Planquette *et al*. [32] by the presence of a black stripe in a low position on body reaching the level of the anal fin in *C*. *arnoldi* contrasting with an uniform coloration, except for the presence of a small stripe that does not surpass the level of the pectoral fin, in *C*. *carsevennensis*. Zarske [35] also distinguished *Copella arnoldi* from *C*. *carsevennensis* (Zarske [35]: tab. 8) by occasionally having a black stripe on the anterior half of body (*vs*. absence), presence of series of brilliant white spots at the posterior half of the body (*vs*. absence), and having the distal edge of dorsal, pelvic, and anal fins black (*vs*. fins never with a conspicuous black border). Zarske [35] observed that males of *C*. *arnoldi* have two color patterns that could be related to sexual activity (Zarske [35]: figs 15 and 16, 18 and 19). Interestingly, the “sexual inactive” males (Zarske [35]: figs 15 and 19) have the same color pattern of the supposed males of *C*. *carsevennensis* (Zarske [35]: figs 28 to 30). Furthermore, some diagnostic features of *C*. *arnoldi* (white spots on the posterior half of the body and distal edge of fins black) can be seen in the figure of the supposed *C*. *carsevennensis* (see Zarske [35]: fig 30].

In the present analysis, data taken from type and non-type material from several localities in Brazil, French Guiana, Surinam, Guyana, and Venezuela (Fig 36) did not reveal any morphological feature that could effectively separate the two species. Morphometric and meristic data (including those related to dorsal-fin position and longitudinal scale counts) largely overlap among the populations examined. Likewise, no color differences were observed that could justify the maintenance of two names. Indeed, there is color pattern variation in males (see Color in alcohol). However, males with exuberant coloration, presenting a longitudinal dark stripe, having brilliant white spots were syntopically collected with males that have similar coloration of females, and lack dark stripe and white spots on the body. Furthermore, males with intermediate coloration are always present (Fig 5B to 5D). If color variation among males is related to hierarchical position, sexual activity, or environmental influences, this should be further investigated through studies using different approaches. The features presented in the literature to distinguish the aforementioned *Copella arnoldi* from *C*. *carsevennensis* are herein interpreted as variation within a single species. Thus, *Copella carsevennensis* is considered a junior synonym of *C*. *arnoldi*, as previously proposed by Fowler [22].

Zarske [35] stated that the type locality of *Copella arnoldi* is Ilha do Arapiranga, near Belém, Pará, which according to him is a collecting site area explored by German aquarists in the past. However, this is highly speculative. Based on the distribution of *C*. *arnoldi* given as the “Amazon”, the only possible inference is that the types might have come from anywhere in the lower Amazon basin.

Eigenmann [18] listed *Pyrrhulina filamentosa* from several localities in Guyana. One of us (MM) had the opportunity to analyze only part of this material: the paralectotypes of *Copeina eigenmanni* from the rios Aruka and Lama, and one specimen from rio Demerara, Kumaka (CAS 227312), and were identified as *Copella arnoldi*, but the identification of the remaining material could not be confirmed.

Kenny [41], Weitzman & Weitzman [1] and Phillip et al. [42] listed *Copella arnoldi* for Trinidad and Tobago but this could not be confirmed in the present study.

**Material examined of *Copella arnoldi* in** S1 Appendix.

### *Copella callolepis* (Regan, 1912)

**Fig 11:**
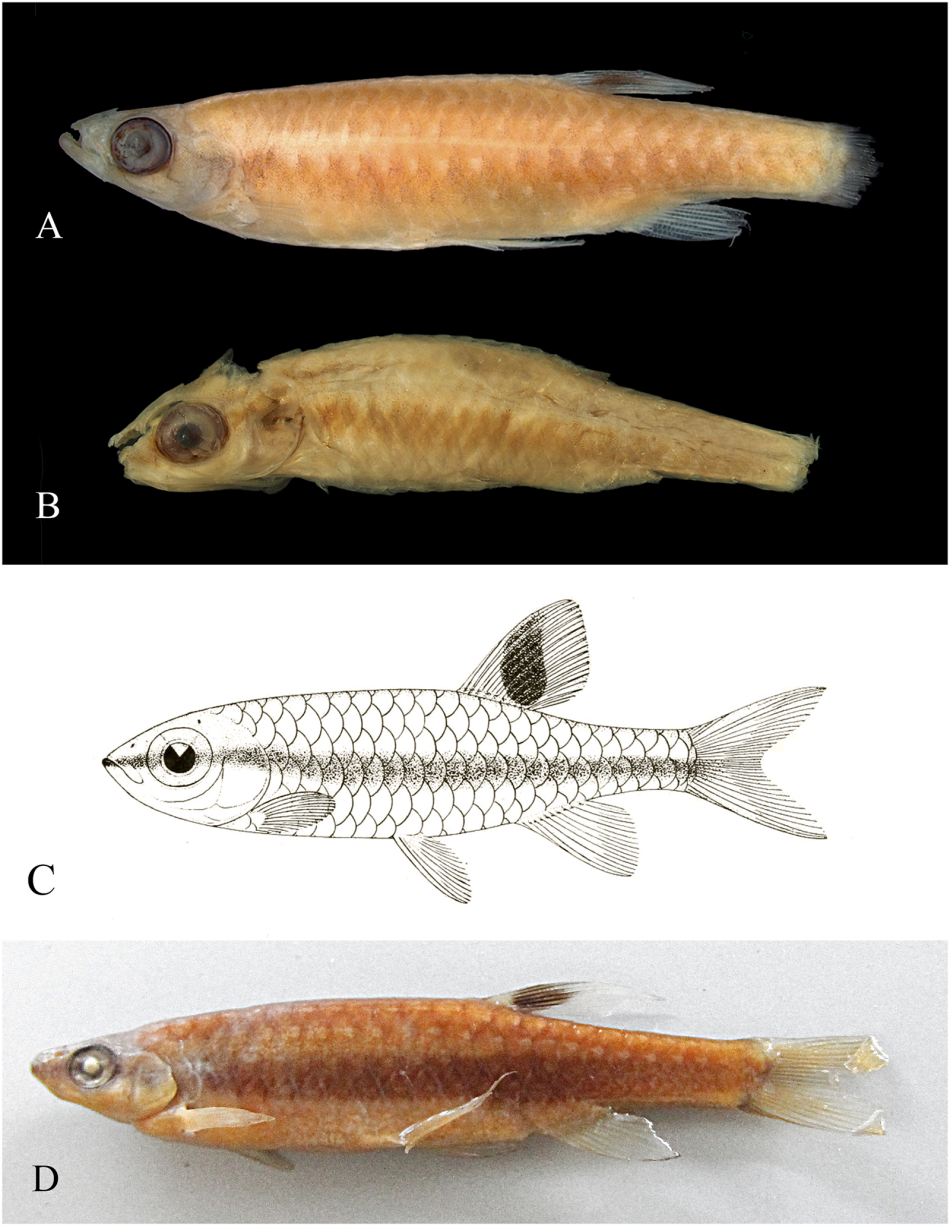
*Copella callolepis*. (A) lectotype of *Copeina callolepis*, BMNH 1909.4.2.27, male, 31.9 mm SL, Amazon, (B) holotype of *Nannostomus stigmasemion*, ANSP 39188, immature, 10.8 mm SL, tributary of the rio Madeira near Porto Velho, Brazil, flipped horizontally (photo by K. Luckenbill & M. Sabaj-Perez), (C) drawing of holotype of *Nannostomus stigmasemion* (Fowler, 1913: fig 4), (D) largest syntype of *Pyrrhulina nigrofasciata* ZMH 1211, male, 35.8 mm SL, upper rio Amazonas.

**Fig 12:**
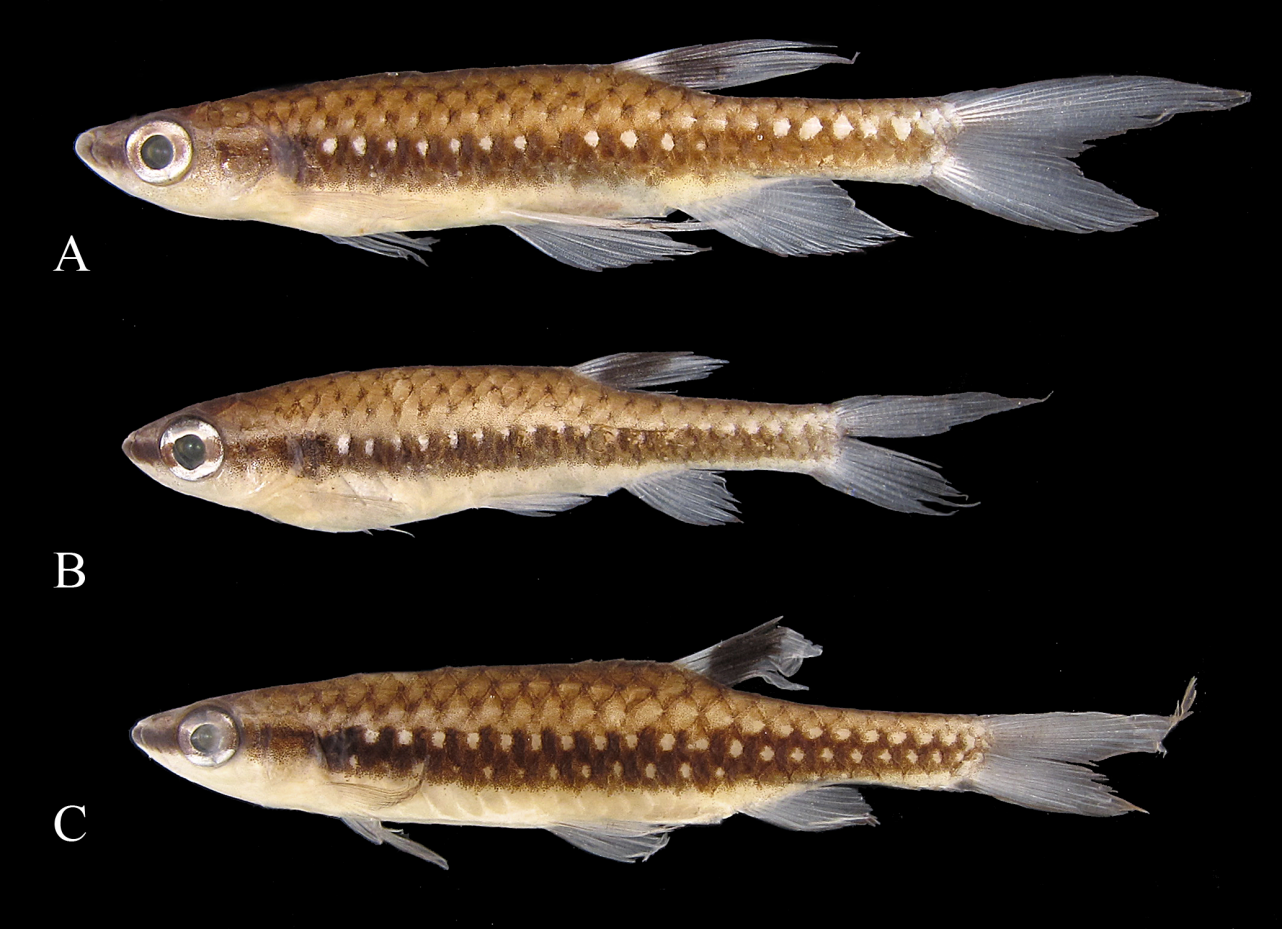
*Copella callolepis*. (A) MZUSP 101930, female, 29.9 mm SL, rio Jari, Amapá, Brazil; MPEG 16249: (B) male, 32.3 mm SL, (C) female, 26.0 mm SL, rio Solimões, Coari, Amazonas, Brazil.

**Fig 13:**
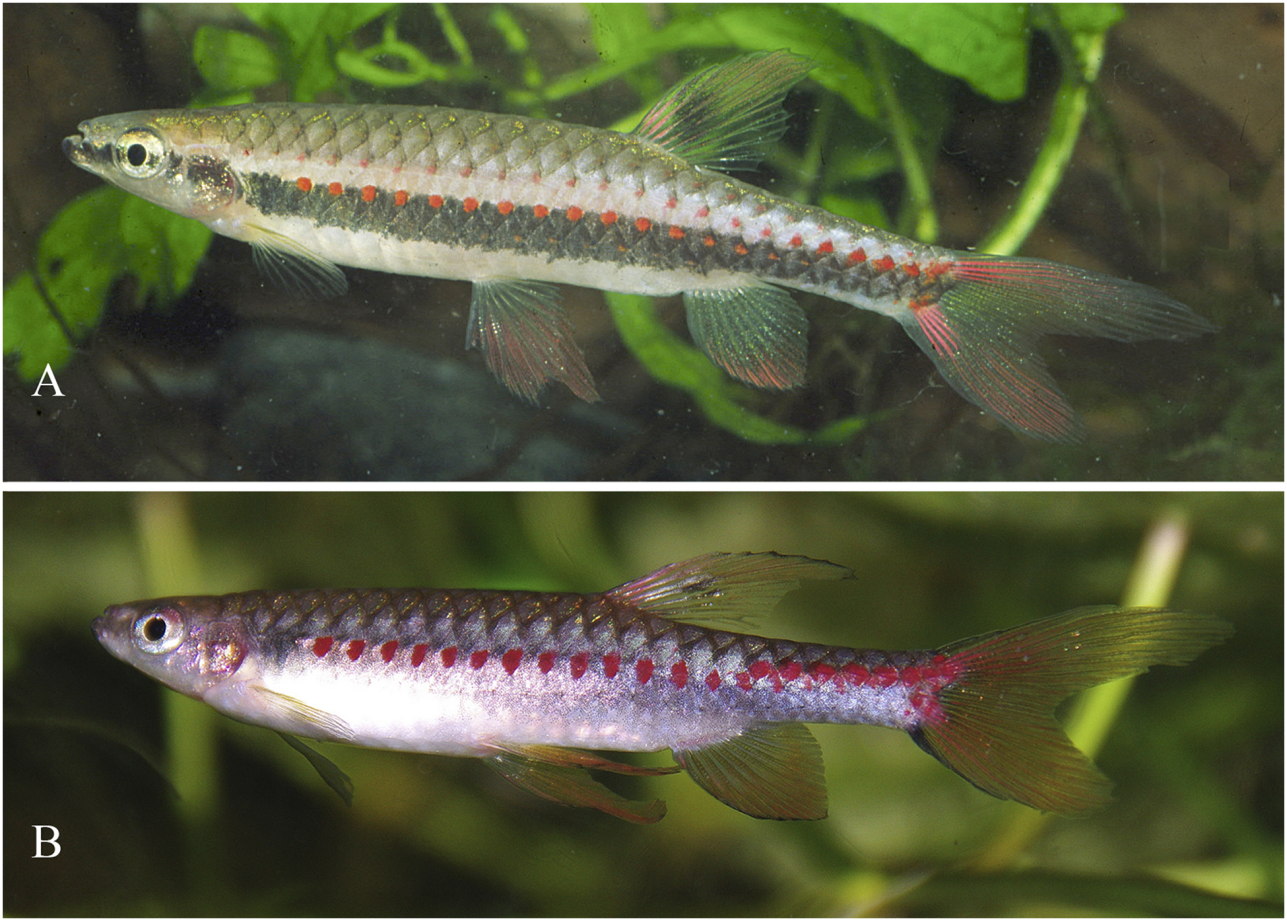
*Copella callolepis*, live specimens. (A) male, aquarium specimen not preserved. Reprinted under a CC BY license, with permission from Hans-George Evers, original copyright 2017. (B) male, aquarium specimen not preserved, reprinted from apisto.sites.no under a CC BY license, with permission of Tom Christoffersen, original copyright 2017.

**Fig 14:**
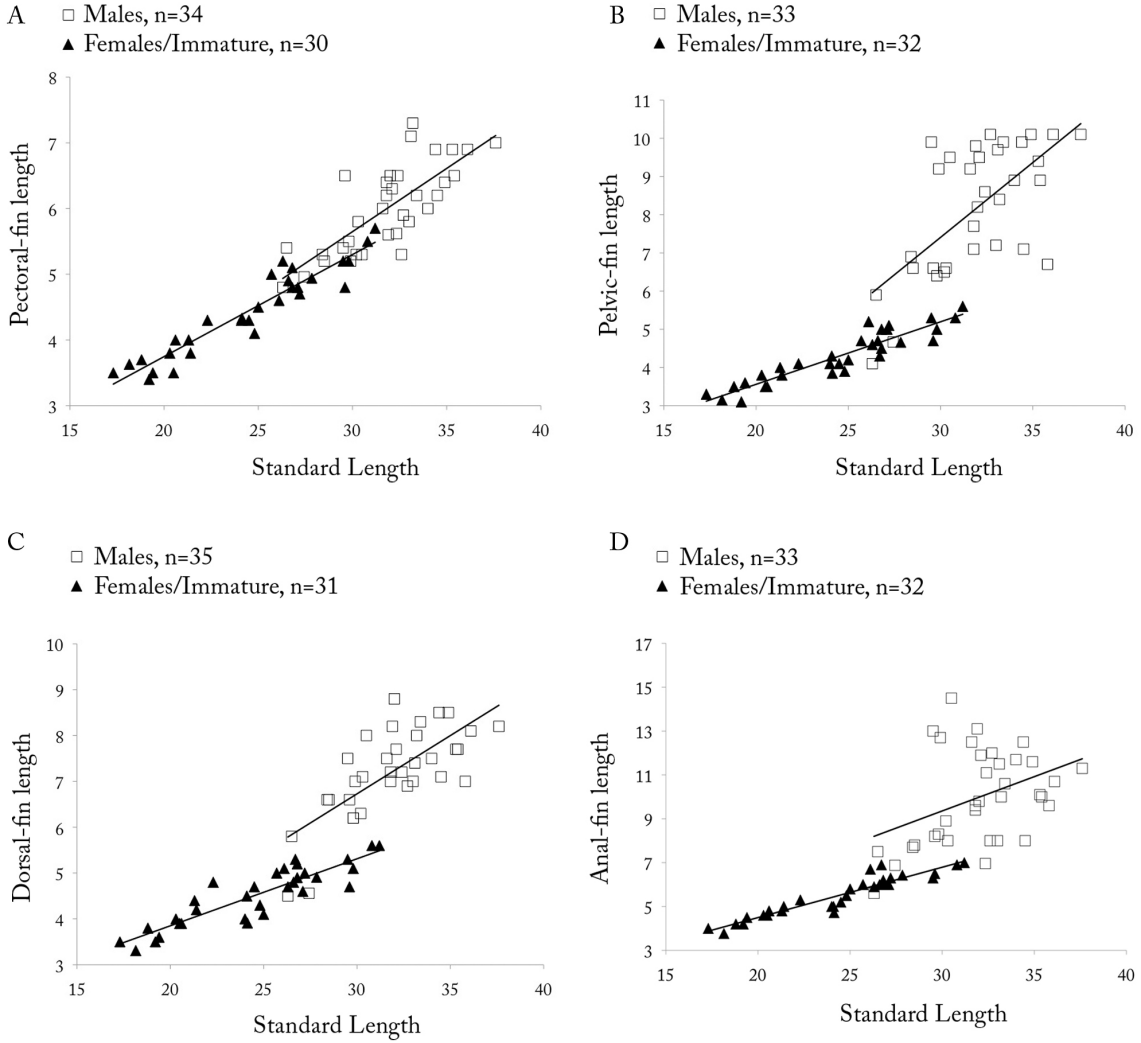
*Copella callolepis*. (A) pectoral-, (B) pelvic-, (C) dorsal-, and (D) anal-fin lengths as a function of SL by sex.

**Fig 15:**
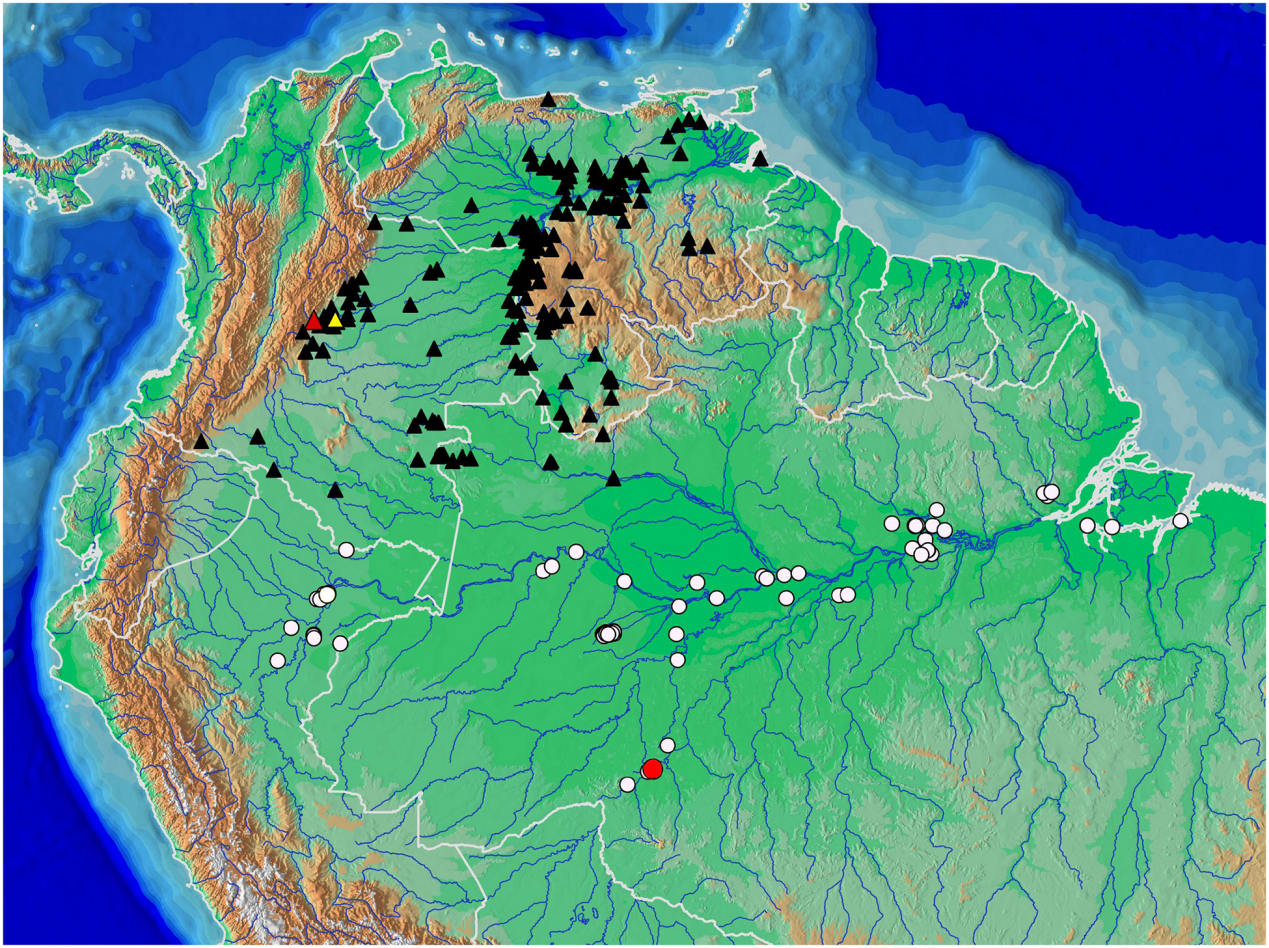
Distribution of *Copella eigenmanni* (black triangles) and *C*. *callolepis* (white circles) with type localities of ‘*Nannostomus’ stigmasemion* (red circle), *C*. *eigenmanni* (red triangle plotted at Villavicencio, see text), and *Copeina metae* (yellow triangle). Some symbols may represent more than one locality or lot of specimens. Type localities of *Copella callolepis* and ‘*Pyrrhulina’ nigrofasciata* are “Amazon” and “upper rio Amazonas at Peru” respectively.

Figs 11–15; Tables 3 and 4

**Table 3:** Morphometrics of *Copella callolepis*.

	*Copeina callolepis*	*Nannostomus stigmasemion*	*Pyrrhulina nigrofasciata*	n	Range			Mean	SD
	Lectotype	Holotype	Largest syntype						
Standard length (mm)	31.9	10.8	35.8	64	17.3	-	37.6	28.4	
**Percents of standard length**									
Body depth	19.8	19.4	20.4	64	14.1	-	21	18.3	1.6
Dorsal- to caudal-fin origin	38.9	40.7	40.2	64	35.4	-	41.4	38.5	1.3
Snout to dorsal-fin origin	62.2	63.9	63.1	64	56.9	-	65.1	61.8	1.5
Snout to pectoral-fin origin	23.2	26.9	21.5	64	20.9	-	26.3	23.7	1.6
Snout to pelvic-fin origin	47.2	48.1	51.1	64	45.4	-	51.7	48.4	1.5
Snout to anal-fin origin	70.9	68.5	73.2	64	67.1	-	73.9	70.2	1.5
Pectoral- to pelvic-fin origin	27.4	22.2	29.9	62	21.8	-	29.7	25.3	2.5
Pelvic- to anal-fin origin	23.7	19.4	21.5	64	19.7	-	25.9	22.5	0.7
Pectoral-fin length males	-	-	15.4	34	16.3	-	22.0	18.8	0.9
Pectoral-fin length fem/imm	-	-	-	31	16.2	-	22.4	18.3	1.2
Pelvic-fin length males	20.5	-	18.7	34	15.6	-	33.7	25.1	0.7
Pelvic-fin length fem/imm	-	-	-	32	15.8	-	20.0	17.6	1.2
Dorsal-fin length males	22.6	-	26.8	34	21.3	-	47.7	31.3	2.0
Dorsal-fin length fem/imm	-	-	-	31	19.6	-	25.7	22.5	1.1
Anal-fin length males	16.7	-	19.6	34	16.6	-	27.6	22.4	0.6
Anal-fin length fem/imm	-	-	-	32	15.9	-	21.3	18.5	1.5
Anal-fin base length	9.2	10.2	8.9	64	6.9	-	10.4	8.7	0.8
Caudal peduncle depth	10.9	8.3	9.5	64	7.8	-	10.7	9.0	1.0
Caudal peduncle length	19.2	18.5	21.8	64	16.7	-	26.2	20.8	2.2
Head length	23.3	27.8	22.1	64	21.4	-	26.8	23.8	1.4
**Percents of head length**									
Eye diameter	34.4	36.7	29.1	62	31.6	-	43.0	36.2	2.8
Snout length	26.7	30.0	31.6	64	25.4	-	32.8	28.6	1.6
Interorbital distance	37.9	36.7	38.0	64	32.9	-	39.7	36.3	1.6
Upper jaw length	32.5	26.7	27.8	64	22.7	-	33.9	28.3	1.8

Lectotype of *Copeina callolepis* BMNH 1909.4.2.27, holotype of *Nannostomus stigmasemion* ANSP 39188, largest syntype of *Pyrrhulina nigrofasciata* ZMH 1211, remaining syntypes of *Pyrrhulina nigrofasciata* ZMH 1212 (3) and non-type material BMNH 1952.7.31.3–5 (3), MPEG 9927 (1), MPEG 13775 (7), MPEG 15917 (6), MPEG 16249 (6), MPEG 17409 (5), MZUSP 23510 (7), MZUSP 85603 (8), MZUSP 85597 (1), MZUSP 101930 (3), MZUSP 101933 (3), MZUSP 103302 (1), UFRO-I 6386 (10), n = number of specimens, SD = Standard deviation. Ranges do not include primary types.

**Table 4:** Meristics of the lectotype of *Copeina callolepis* BMNH 1909.4.2.27 and of largest probable syntype of *Pyrrhulina nigrofasciata* ZMH 1211.

	*Copeina callolepis*	*Pyrrhulina nigrofasciata*
	Lectotype	Largest syntype
Dorsal-fin rays	ii8	ii8
Pectoral-fin rays	i8	i9
Pelvic-fin rays	i7	i7
Anal-fin rays	iii9	iii9
Predorsal scales	13	12
First longitudinal scale row	12	12
Fourth longitudinal scale row	22	24
Longitudinal scale rows dorsal to pelvic	5	6
Longitudinal scale rows dorsal to anal	5	5
Circumpeduncular scale rows	10	10
Total vertebrae	34	-

Meristic data of *Nannostomus stigmasemion* was not taken due to the poor condition of the material.

*Copeina callolepis* Regan [20]: 393 [in part, BMNH 1909.4.2.27, type locality: Amazon].—Myers [21]: 111 [comparison with *C*. *compta*].—Fowler [22]: 344 [literature compilation; placed as synonym of *Copeina arnoldi*].—Weitzman & Weitzman [1]: 242 [considered as synonym of *Copella nattereri*]

*Nannostomus stigmasemion* Fowler [43]: 523–524, fig 4 [type locality: tributary of the rio Madeira near Porto Velho, Brazil]. Fowler [22]: 259 [listed].

*Pyrrhulina nigrofasciata* Meinken [24]: 115–117, figs 1 to 3 [type locality: upper rio Amazonas at Peru].—Géry [5]: 147 [possible synonym of *Copella metae*].—Wilkens, [44]: 156 [type catalog].

*Copella callolepis*.—Myers [4] [new combination; possible synonym of *C*. *nattereri*].—Géry [6]: 28 [considered *Copella callolepis* conspecific with *C*. *nattereri*].—Zarske & Géry [7]: 21, figs 4 and 5 [lectotype designation; photo lectotype BMNH 1909.4.2.27; included as synonym of *Copella nattereri*].

*Copella nigrofasciata*.—Ortega & Vari [45]: 10 [rio Amazonas, Peru; listed, new combination].—Zarske & Géry [46]: 14, fig 4 [photo of possible syntype ZMH 1211; comparison with *Pyrrhulina zigzag*].—Weitzman & Weitzman [1]: 242 [literature compilation; comparison with *C*. *metae*].—Zarske & Géry [7]: 26, figs 11, 22 [identification key].—Oyakawa & Netto-Ferreira [15]: 64 [literature compilation].—Montag *et al*. [39]: 18 [FLONA de Caxiuanã in igarapé; listed].

*Copella eigenmanni*.—Géry [5]: 141 [misidentification of unnumbered figure pg. 141 labeled “possibly *C*. *eigemmanni*”].

*Copella nattereri*.—Zarske & Géry [7]: 24, figs 7–9 and 20 [misidentification; redescription; taxonomic notes; identification key].

**Diagnosis.** *Copella callolepis* can be distinguished from all congeners by having a series of conspicuous clear spots on each scale along the fourth longitudinal scale row, not followed by a black spot (*vs*. absence of clear spots in *Copella compta*, *C*. *eigenmanni*, and *C*. *vilmae*; clear spots not present on the fourth longitudinal scale row in some males of *C*. *arnoldi*; clear spots followed by dark spots on *C*. *nattereri*). Additionally, *C*. *callolepis* can be distinguished from all congeners, except some specimens of *C*. *eigenmanni*, by having a black longitudinal band on body from the dentary to the caudal peduncle (*vs*. absence or longitudinal dark band extending up to the anal fin).

**Description.** Morphometrics in Table 3 and meristics of types in Table 4. Largest examined male 37.6 mm SL, female 30.4 mm SL. Greatest body depth slightly anterior to vertical through pelvic-fin origin. Body cylindrical, slightly compressed laterally. Dorsal profile of body straight to slightly convex from tip of snout to end of supraoccipital, slightly convex from that point to dorsal-fin origin, posteroventrally inclined along dorsal-fin base and straight along caudal peduncle. Ventral profile of body convex to posteroventrally inclined from anterior tip of dentary to vertical through anterior margin of orbit, straight from that point to vertical through pectoral-fin origin, slightly convex from that point to pelvic-fin origin, straight from pelvic-fin origin to anal-fin origin, posterodorsally inclined along anal-fin base and straight along caudal peduncle.

Mouth upturned. Premaxillary teeth in one row, with 14 (3), 15 (1), 16 (3), or 17 (1) teeth, decreasing in size laterally. Number of maxillary teeth sexually dimorphic, 11 (2), 15 (1), or 16 (1) in males, 5(2), 6 (1), or 9 (1) in females, decreasing in size posteriorly, especially in males. Dentary teeth in two rows, outer with 7 (1), 8 (2), 9 (4), or 10 (1) increasing in size laterally, inner with 21 (2), 22 (1), 23 (1), 25 (2), 26 (1), or 34 (1) teeth, decreasing in size laterally.

Dorsal fin with ii (64), 8* (62) or 9 (2) rays, second and third branched rays longer. Pectoral fin with i (64), 8* (8), 9 (44), or 10 (12) rays, second branched ray longest, not reaching pelvic-fin origin in males and females. Pelvic fin with i (64), 7* (59) or 8 (5) rays, third branched ray longest. Anal fin with iii (11), 7,i (2), or 8,i* (62) rays, fourth and fifth branched rays longer. Adipose fin absent. Caudal fin with i (59), 7 (3), 8 (52), or 9 (4) rays in upper lobe, first and second branched rays longer, and 7 (59) or 8 (1), i (60) rays in lower lobe, first and second branched rays longer. Upper caudal-fin lobe longer than lower.

Predorsal scales 12 (4), 13* (35), or 14 (23), in one series. First longitudinal scale row with 12* (4), 13 (39), or 14 (11) scales. Fourth longitudinal scale row with 21 (1), 22* (10), 23 (32), or 24 (19) scales. Lateral line not pored, first lateral line scale with small canal medially on its anterior portion, without lateral opening. Longitudinal scale rows between dorsal-fin origin and pelvic-fin origin 5* (24) or 6 (40). Longitudinal scale rows between dorsal-fin origin and anal-fin origin 5* (57) or 6 (8). Circumpeduncular scale rows 10* (65). Total number of vertebrae 32 (11), 33 (30), 34* (16), or 35 (3).

**Color in alcohol.** Overall ground coloration of body beige to brown. Dorsal portion of body dark. Thin predorsal dark stripe, frequently wider over second and third scales. Scales on middorsal portion of body with posterior border dark, forming slight reticulate pattern; some specimens with small dark spot at posterior border of scale on that region (Fig 12). Black stripe extending from anterior tip of dentary to end of caudal peduncle, along fourth and fifth longitudinal scale rows. Clear longitudinal stripe over third longitudinal scale row. Conspicuous clear spot on each scale of fourth longitudinal scale row, from behind opercle to vertical through end of dorsal-fin base and on each scale of third longitudinal scale row of caudal peduncle (Fig 12A and 12B). Rarely, specimens with clear spot also on each scale of third to fifth longitudinal scale rows (Fig 12C). Ventral region of body clear. Dorsal fin with black round spot. Remaining fins hyaline. Pelvic and anal fins usually with dark edge, more intense in males. Some males with distal tip of dorsal fin dark. Some specimens with base of middle caudal-fin rays faint dark. Dorsal and ventral procurrent caudal-fin rays dark, forming black triangle at base of lower caudal-fin lobe. Dorsal procurrent caudal-fin rays hyaline and ventral procurrent caudal-fin rays faint dark in juveniles.

**Color in life.** Dorsal portion of body beige to gray. Black stripe extending from anterior tip of dentary to end of caudal peduncle, along fourth and fifth longitudinal scale rows (Fig 13A). Black stripe rarely faded (Fig 13B), only on courtship or when frightened (T. Christoffersen, personal observation). Clear longitudinal stripe over third longitudinal scale row. Red spot on each scale of fourth longitudinal scale row, from behind opercle to vertical through end of dorsal-fin base, and on each scale of third longitudinal scale row of caudal peduncle (Fig 13). Rarely, specimens with red spot on each scale of second to fifth longitudinal scale rows. Ventral portion of body white. Fins yellow to orange. Basal portion of dorsal fin red. Some specimens with base of longest upper and lower caudal-fin rays red.

**Sexual dimorphism.** Males longer than females. Males with more numerous maxillary teeth than females (see description above). Pelvic, dorsal, and anal fins longer in males than females (Fig 14). Pectoral fin apparently not sexually dimorphic. Tip of adpressed pelvic fin reaching to base of third branched anal-fin ray in males, and to anterior margin of anus in females. Tip of adpressed dorsal fin reaching caudal-fin base in males and approximately to one-half length of caudal peduncle in females. Tip of adpressed anal-fin reaching level of first ventral procurrent rays in males, and to two-thirds length of caudal peduncle in females. Upper caudal-fin lobe longer than lower, more evident in males.

**Distribution.** *Copella callolepis* is known in the Amazon basin from the rio Ucayali and Putumayo drainages in Peru, rio Amazonas and rio Madeira in Brazil, to the Amazon estuary in Pará State, Brazil (Fig 15).

**Remarks.** The analysis of type material of *Copella callolepis* revealed that Regan [20] used two distinct species to describe it. One of them (BMNH 1909.4.2.27), although faded colored, has a longitudinal dark band and three longitudinal rows of clear spots on the body. The abdominal region, as well as other remaining portions of body, is devoid of series of black spots characteristic of the spotted tetra *C*. *nattereri* (Fig 11A). The other specimen (BMNH 1909.4.2.28) clearly corresponds to the spotted tetra *C*. *nattereri*, with clear marks close to the posterior edge of the body scales, posteriorly bordered with dark spots. Hence, *Copella callolepis* has long been considered synonym of *C*. *nattereri* (see synonym list). However, Zarske & Géry [7] erected as lectotype of *C*. *callolepis* the specimen from BMNH 1909.4.2.27, linking the name *C*. *callolepis* to the species with a black longitudinal band on body, long known as the black-banded *C*. *nigrofasciata*.

*Nannostomus* (= *Copella*) *stigmasemion* was described by Fowler [43] from the rio Madeira basin near Porto Velho, based on a single juvenile specimen 10.8 mm SL. This specimen was not cited by Weitzman & Weitzman [1], but inside the jar with the holotype there is a label written by S. Weitzman: “This specimen described by Fowler as *Nannostomus stigmasemion* is a juvenile of *Copella nattereri*”. Although poorly preserved, it was possible to notice that the specimen is indeed a *Copella*, not a *Nannostomus* species. However, it lacks the characteristic pigmentation of *Copella nattereri*, that is one black spot on each scale; instead it has a black longitudinal band on body (Fig 11B), similar to that present in *C*. *callolepis*. This could be confirmed by the drawing of the holotype in the original description (Fig 11C). Additionally, according to our study, *Copella nattereri* only occurs near the mouth of the rio Madeira, far from the surroundings of Porto Velho. The only species occurring in the rio Madeira basin near Porto Velho is *Copella callolepis*. Therefore, *C*. *stigmasemion* is herein considered synonym of *C*. *callolepis*.

Meinken [24] originally described *Pyrrhulina nigrofasciata* based on three aquarium specimens, two males and one female, said to be imported from the Peruvian Amazon, sent by J. Franke. Possible syntypes are deposited as BMNH 1952.7.31.3–5 (1 male, 32.3 mm SL, 1 female 24.1 mm SL, and 1 unsexed 27.4 mm SL) and as ZMH 1211 (1 male 35.8 mm SL) and ZMH 1212 (1 male 32.6 mm SL, 1 female 29.6 mm SL and 1 unsexed 26.3 mm SL), sent by Meinken in 1952 (O. Crimmen and R. Thiel personal communication). All specimens correspond to the same species referred here as *Copella callolepis*. By comparing the measurements of a fully grown male and a female (37.2 mm SL and 31.0 mm SL respectively) given by Meinken [24] to the measurements taken from the probable syntypes, most of the data match with those of the male ZMH 1211 (35.8 mm SL) (Fig 11D) and the female ZMH 1212 (29.6 mm SL), which are the largest male and the largest female of the possible syntypes. However, the lot ZMH 1212 is represented by three specimens, more than what Meinken had in his hands. It is not possible to know whether one specimen was incorporated to ZMH 1212 after Meinken’s description, but based on the data available, it is most likely that the original syntypes of *Pyrrhulina nigrofasciata* are those belonging to the ZMH collection.

Thus, according to the analysis of the lectotype of *C*. *callolepis*, of possible types of ‘*Pyrrhulina’ nigrofasciata*, and of the type of ‘*Nannostomus’ stigmasemion*, along with considerable amount of freshly collected material listed herein, we consider them to be conspecific. Following the principle of priority, and that the senior synonym was described before 1899, ‘*Nannostomus*’ *stigmasemion* and *‘Pyrrhulina’ nigrofasciata* are considered junior synonyms of *Copella callolepis*.

One of the paralectotypes of *Pyrrhulina nattereri* (NMW 56974), is probably *Copella callolepis*, but this could not be confirmed due to the poor condition of the material.

*Copella nigrofasciata* (= *C*. *callolepis*) cited by Bogotá-Gregory & Maldonado-Ocampo [47] and Maldonado *et al*. [48] from the Amazon basin of Colômbia are probably misidentifications of *Copella eigenmanni* (see Fig 15) but this could not be confirmed.

**Material examined of *Copella callolepis* in** S1 Appendix.

### *Copella compta* (Myers, 1927)

**Fig 16:**
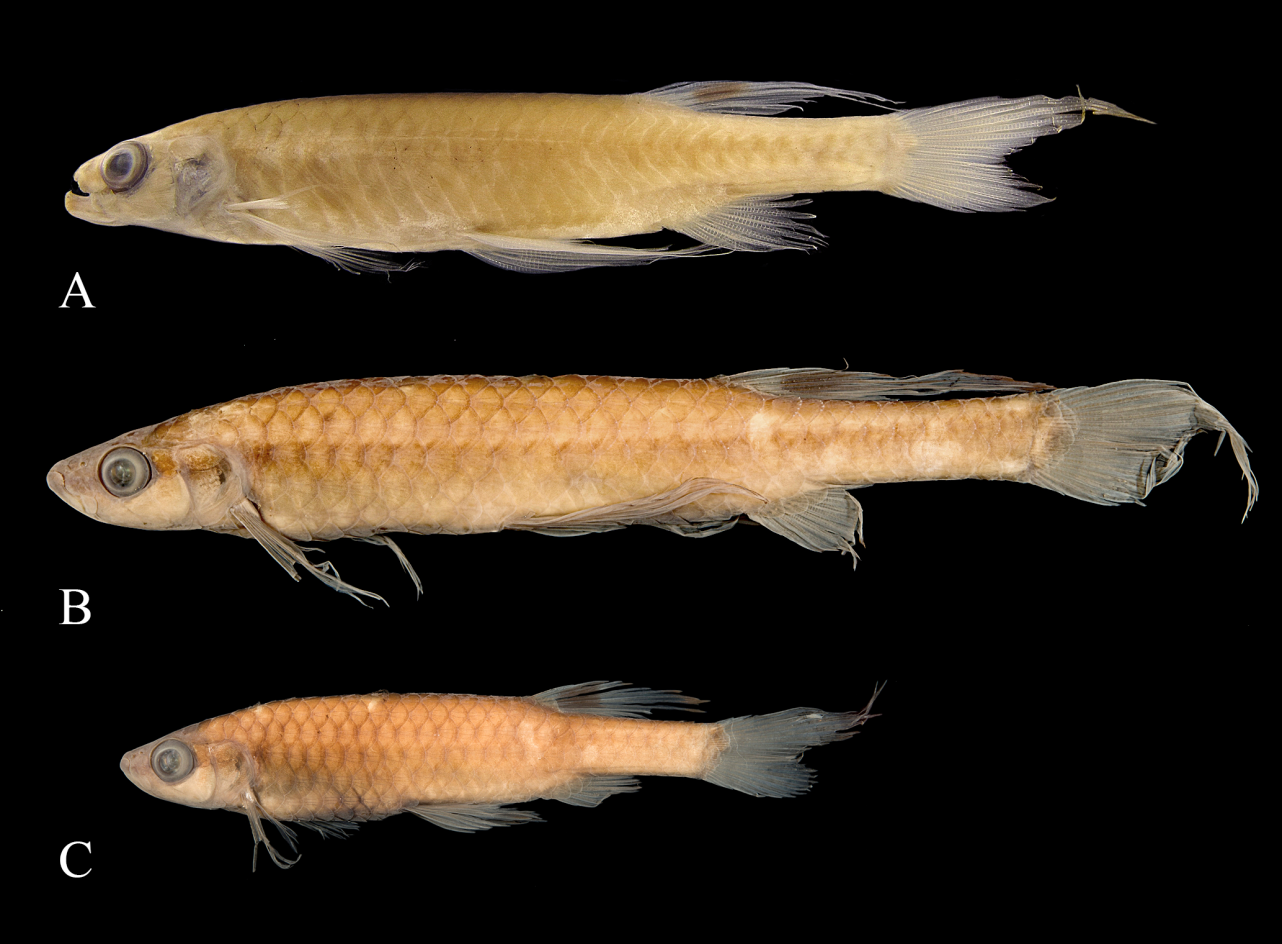
Copella compta. (A) holotype, male, CAS 60496, 52.0 mm SL, rio Negro at São Gabriel da Cachoeira, Amazonas, Brazil; MZUSP 9162 (B) male, 68.9 mm SL, (C) female, 40.3 mm SL, São Gabriel da Cachoeira, Amazonas, Brazil.

**Fig 17:**
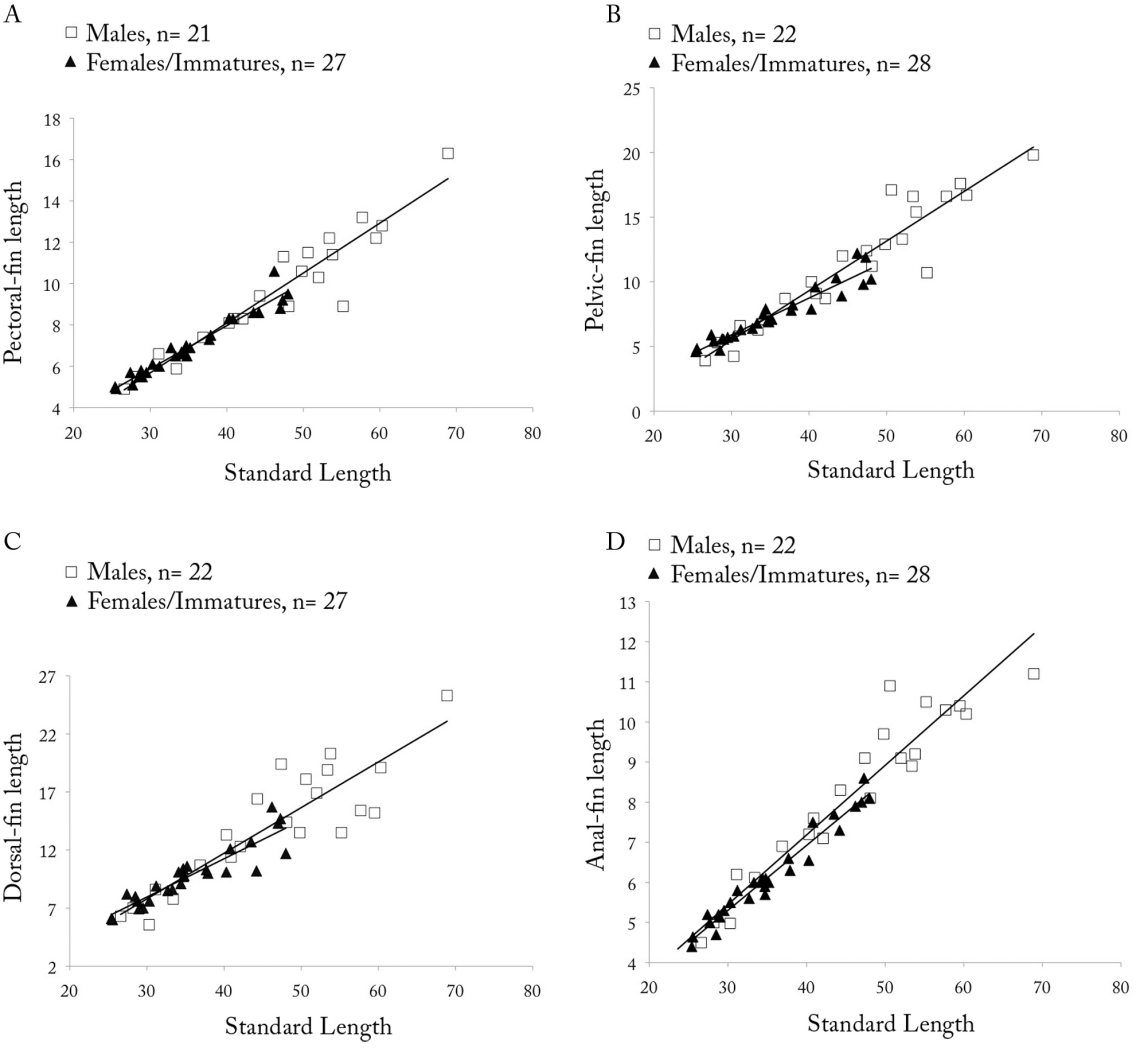
*Copella compta*. (A) pectoral-, (B) pelvic-, (C) dorsal-, and (D) anal-fin length as a function of SL by sex.

**Fig 18:**
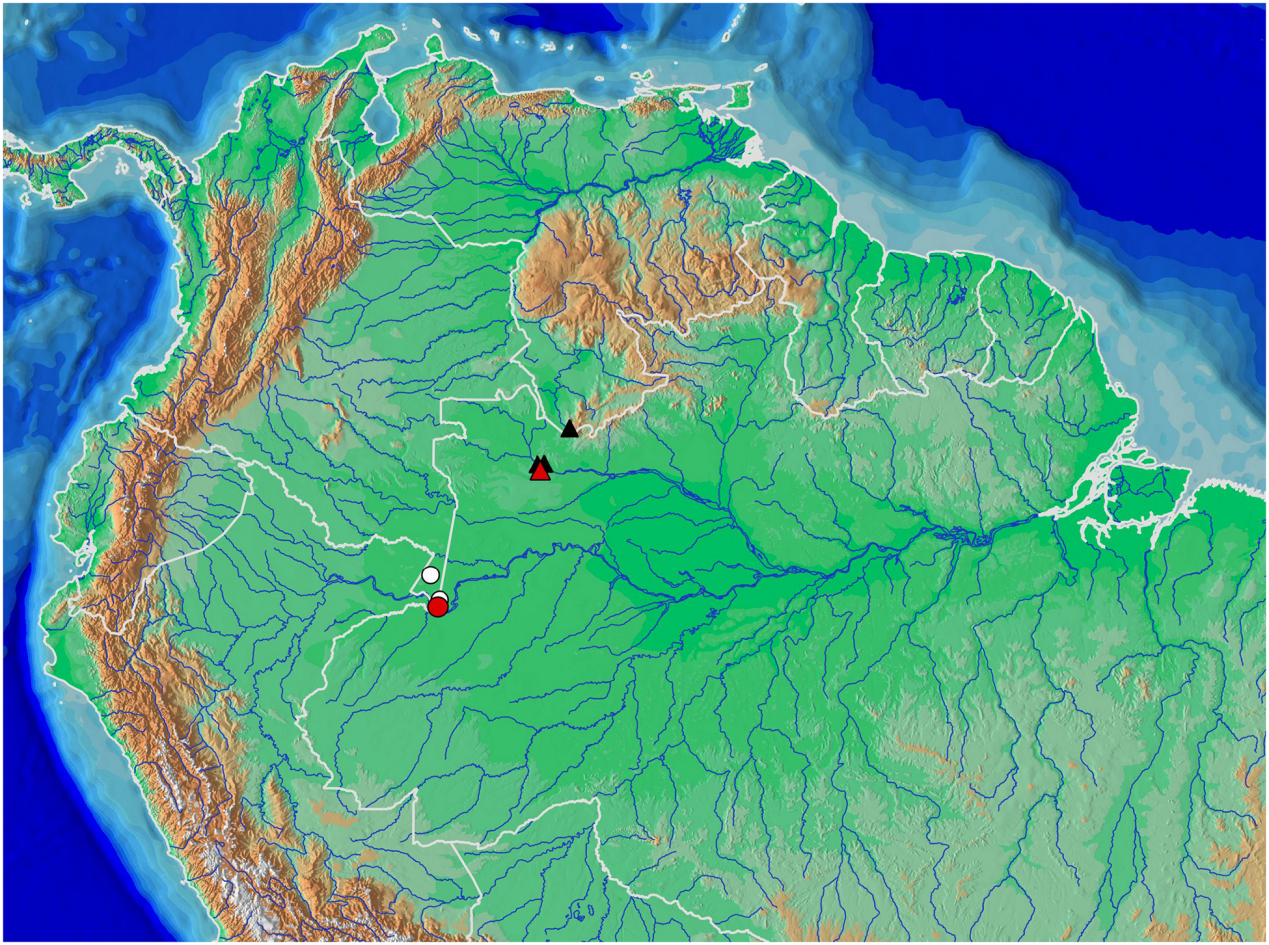
Distribution of *Copella compta* represented by triangles (type locality in red) and *C*. *vilmae* represented by circles (type locality in red). Some symbols may represent more than one locality or lot of specimens.

Figs 2, 16–18; Tables 5 and 6

**Table 5:** Morphometrics of *Copella compta*.

	*Copeina compta*	n	Range			Mean	SD
	Holotype						
Standard length (mm)	50.6	50	23.6	-	68.9	39.6	
**Percents of standard length**							
Body depth	16.9	50	13.8	-	19.3	16.5	1.2
Dorsal- to caudal-fin origin	31.9	50	30.5	-	35.5	32.7	1.3
Snout to dorsal-fin origin	68.4	50	64.4	-	69.9	67.8	1.2
Snout to pectoral-fin origin	21.7	50	19.3	-	24.4	22.3	1.2
Snout to pelvic-fin origin	47.2	50	45.7	-	50.9	48.4	1.1
Snout to anal-fin origin	71.2	50	67.3	-	73.1	70.7	1.1
Pectoral- to pelvic-fin origin	25	50	24.5	-	28.8	27.0	1.1
Pelvic- to anal-fin origin	24.1	50	20.5	-	25.3	23.0	1.1
Pectoral-fin length males	22.7	21	16.1	-	23.9	20.6	2.0
Pectoral-fin length fem/imm	-	27	18.4	-	23	19.7	0.9
Pelvic-fin length males	33.8	23	14.0	-	33.8	23.9	5.5
Pelvic-fin length fem/imm	-	28	16.6	-	26.3	20.7	2.1
Dorsal-fin length males	35.7	22	18.4	-	40.9	29.9	5.7
Dorsal-fin length fem/imm	-	27	23.1	-	33.9	27.3	2.8
Anal-fin length males	21.6	23	15.2	-	21.6	17.9	1.4
Anal-fin length fem/imm	-	28	16.3	-	19.1	17.5	0.7
Anal-fin base length	9.2	50	6.8	-	10.0	8.3	0.6
Caudal peduncle depth	9.2	50	7.4	-	9.7	8.8	0.5
Caudal peduncle length	19.7	50	18.4	-	24.0	20.9	1.3
Head length	22.5	50	19.9	-	24.9	23.2	1.1
**Percents of head length**							
Eye diameter	30.0	49	27.3	-	40.3	33.5	3.1
Snout length	27.1	50	25	-	33.6	28.5	2.0
Interorbital distance	34.9	49	28.6	-	37.8	34.1	1.8
Upper jaw length	-	50	26.8	-	39.1	33.7	2.0

Holotype of *Copeina compta* CAS 60496, paratypes of *Copeina compta* CAS 60497 (7), MCZ 31568 (3) MHNG 2200.018 (1), SU 18070 (5) and non-type material AMNH 231274 (9), INPA 9162 (23), and MZUSP 27457 (2), n = number of specimens, SD = Standard deviation. Range does not include the holotype.

**Table 6:** Meristics of the holotype of *Copeina compta* CAS 60496.

	Holotype
Dorsal-fin rays	ii8
Pectoral-fin rays	i9
Pelvic-fin rays	i7
Anal-fin rays	iii9
Caudal-fin rays	-
Predorsal scales	18
First longitudinal scale row	17
Fourth longitudinal scale row	27
Longitudinal scale row dorsal to pelvic	6
Longitudinal scale row dorsal to anal	5
Circumpeduncular scale row	10
Total vertebrae	36

*Copeina compta* Myers [21]: 111 [Type locality: Brazil, creek above São Gabriel rapids, rio Negro].—Fowler [22]: 344 [literature compilation].—Böhlke [49]: 23 [type catalog].—Zarske [35]: 14 [cited].

*Copella compta*.—Myers [4]: 12 [new combination].—Géry [6]: 25 [comparison with *C*. *vilmae*].—Géry [5]: 147 [comparison with *Copella vilmae*; unnumbered figure of pg. 147].—Weitzman & Weitzman [1]: 242 [literature compilation].—Oyakawa & Netto-Ferreira [15]: 64 [literature compilation].—Wallace [50]: 134, fig 97 [listed].

**Diagnosis.** *Copella compta* can be distinguished from all congeners, except *C*. *vilmae*, by having 16–18 scales in the first longitudinal scale row (*vs*. 13–15), and additionally by having 26–27 scales in the fourth longitudinal scale row (*vs*. 23–25). Males of *C*. *compta* can be distinguished from the males of *C*. *vilmae* by the absence of rows of conspicuous dark scales irregularly arranged on body (*vs*. presence); females of *C*. *compta* can be distinguished from the females of *C*. *vilmae* by the absence of a dark longitudinal band on body (*vs*. presence). *Copella compta* is the largest species of the genus, the males reaching 68.9 mm SL and the females 48.1 mm SL (*vs*. less than 55.5 mm SL and 44.2 mm SL, respectively, in the congeners).

**Description.** Morphometrics in Table 5 and meristic of holotype in Table 6. Largest examined male 68.9 mm SL, female 48.0 mm SL. Greatest body depth at vertical through pelvic-fin origin. Body cylindrical, slightly compressed laterally. Dorsal profile of body straight or slightly convex from tip of snout to end of supraoccipital, straight or slightly convex from that point to dorsal-fin origin, posterioventrally inclined along dorsal-fin base and straight along caudal peduncle. Ventral profile of body convex from anterior tip of dentary to vertical through anterior margin of orbit, straight from that point to vertical through pectoral-fin origin, straigth to slightly convex from that point to pelvic-fin origin, straight from pelvic-fin origin to anal-fin origin, posterodorsally inclined along anal-fin base and straight along caudal peduncle.

Mouth upturned. Premaxillary teeth in one row, with 20 (2) or 21 (1) teeth, decreasing in size laterally. Number of maxillary teeth sexually dimorphic, 14 (1) in males, and 5 (1) or 8 (1) in females, decreasing in size posteriorly, especially in males. Dentary teeth in two rows, outer with 10 (1), 11 (1), or 12 (1), increasing in size laterally, inner with 36 (2) or 37 (1) teeth, decreasing in size laterally.

Dorsal fin with ii, 8* (49) rays, second and third branched rays longer. Pectoral fin with i (46), 8 (2), 9* (20), or 10 (24) rays, first three branched rays longer. Pelvic fin with i, 7* (49) rays, third branched ray longest. Anal fin with iii (4), 7,i (1), or 8,i* (49) rays, fourth and fifth branched rays longer. Adipose fin absent. Caudal fin with i (48), 7 (1) or 8 (47) rays in upper lobe, first and second branched rays longer, and 6 (1) or 7* (47), i (48) rays in lower lobe, second and third branched rays longer. Upper caudal-fin lobe longer than lower one.

Predorsal scales 17 (16), 18* (32), or 19 (1), in one series. First longitudinal scale row with 16 (3), 17* (32), or 18 (12) scales. Fourth longitudinal scale row with 26 (16), 27* (33), or 28 (1) scales. Lateral line not pored, first lateral line scale with small canal medially on its anterior portion, without lateral opening. Longitudinal scale rows between dorsal-fin origin and pelvic-fin origin 6* (50). Longitudinal scale rows between dorsal-fin origin and anal-fin origin 5* (50). Circumpeduncular scale rows 10* (50). Total number of vertebrae 36* (2) and 37 (11).

**Color in alcohol.** Overall ground coloration of body beige to brown. Dark stripe extending from anterior tip of dentary to posterior tip of opercle. Dorsal portion of body dark. Thin dark stripe at predorsal region. Dorsolateral body scales with dark posterior border, forming reticulate pattern. Males with dark longitudinal band on fourth and fifth longitudinal scale rows, extending from opercle to end of caudal peduncle or only conspicuous behind opercle, fading posteriorly, with anterior portion of scales of posterior portion of body dark. Females without dark band on body, anterior portion of third to sixth longitudinal scale series faint dark. Ventral region of body clear. Dorsal procurrent caudal-fin rays hyaline in males and females (probably as result of poor preserved material), ventral procurrent caudal-fin rays dark in males and females. Females and juveniles with inconspicuous dark spot at base of upper caudal-fin lobe. Dorsal fin with black round spot. Pectoral, pelvic, anal, and caudal fins hyaline; distal profile of pelvic and anal fins and tip of largest dorsal-fin rays frequently dark (Fig 16).

**Color in life.** Unknown.

**Sexual dimorphism.** Males longer than females. Males with more numerous maxillary teeth than females (see description above). Pectoral, pelvic, dorsal, and anal fins only slightly longer in males than in females (Fig 17). Tip of pectoral fin reaching to vertical through 12^th^ scale of first longitudinal row in males, and through nineth scale in females. Tip of adpressed pelvic fin reaching to vertical through bases between first and fourth branched anal-fin rays in males, and to anal-fin origin in females. Tip of adpressed dorsal fin reaching to caudal-fin base in males, and approximately to two-thirds length of caudal peduncle in females. Upper caudal-fin lobe longer than lower, especially in males. Color pattern differences described in “Color in alcohol” section. Breeding tubercles on head and body of some male specimens.

**Distribution.** Upper rio Negro upstream from São Gabriel da Cachoeira, Brazil and Venezuela (Fig 18).

**Remarks.** Myers originally described *Copeina compta* based on the holotype (“type”) IU 17693 (now CAS 60496) (Fig 16A) and two lots of paratypes IU 17694 (now CAS 60497) and MCZ 31568, with no specification of the number of specimens in each lot. The lot SU 18070 has the same data as those cited by Myers and the corresponding specimens are considered paratypes, probably splitted from the original lots after the description. SU 18070 had originally 11 specimens [49] but now there are nine specimens in alcohol (five were analyzed), one c&s, and one transferred to MHNG 2200.038.

Citations of *Copella compta* by Zarske & Géry [7] from rio Aracá, near Barcelos (Zaske & Géry [7]: fig 24) and by Bogotá-Gregory & Maldonado-Ocampo [47] and Maldonado-Ocampo *et al*. [48] for rio Amazonas in Colombia could not be confirmed herein.

**Material examined of *Copella compta* in** S1 Appendix.

### *Copella eigenmanni* (Regan, 1912)

**Fig 19:**
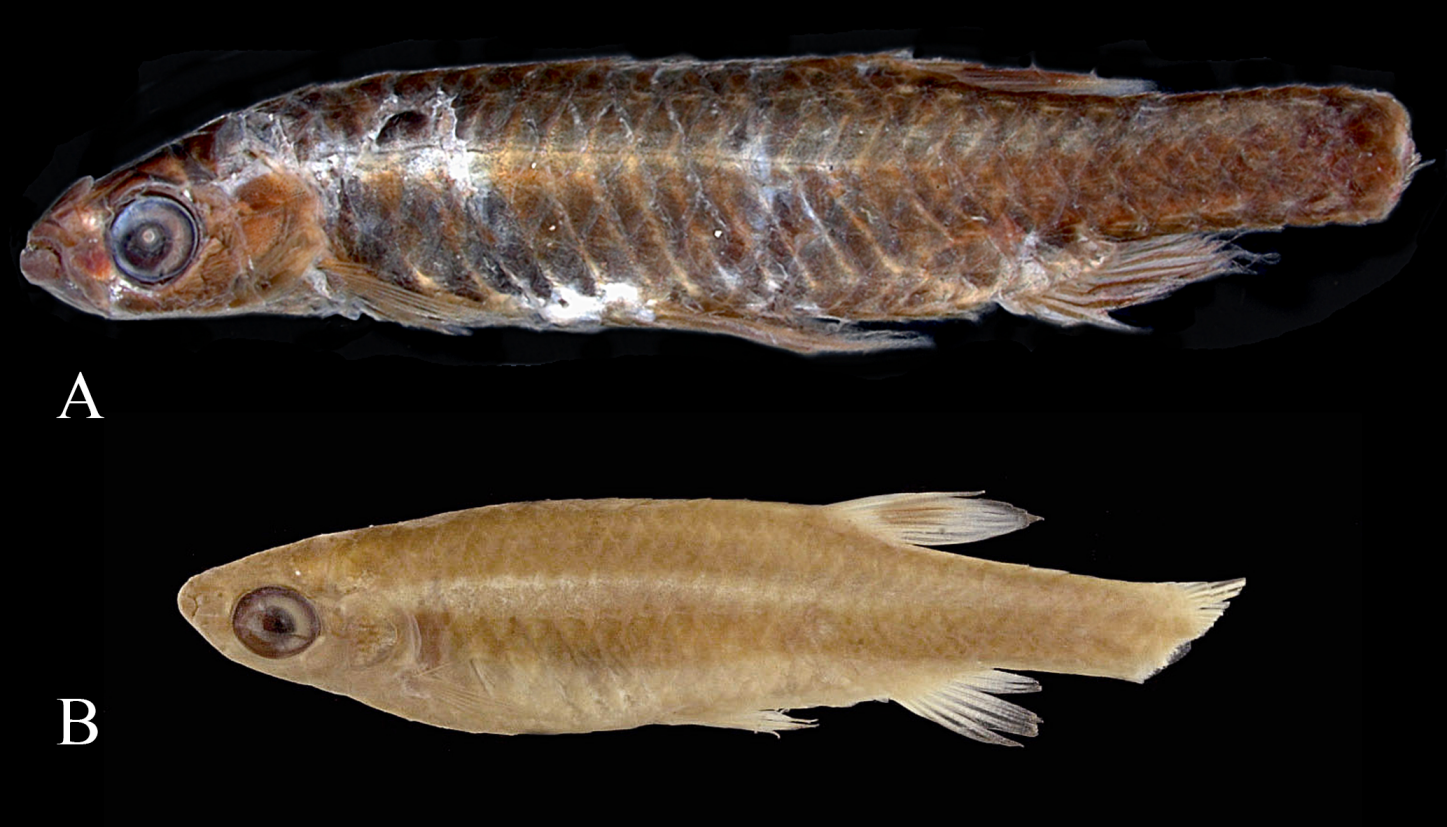
*Copella eigenmanni*. (A) Lectotype of *Copeina eigenmanni*, BMNH 1869.7.25.6 male, 37.7 mm SL, Bogotá (= probably rio Meta), Colombia, (B) holotype of *Copeina metae*, CAS 60494, male, 28.8 mm SL, Barrigona [= Puerto Barrigón], rio Meta, Colombia.

**Fig 20:**
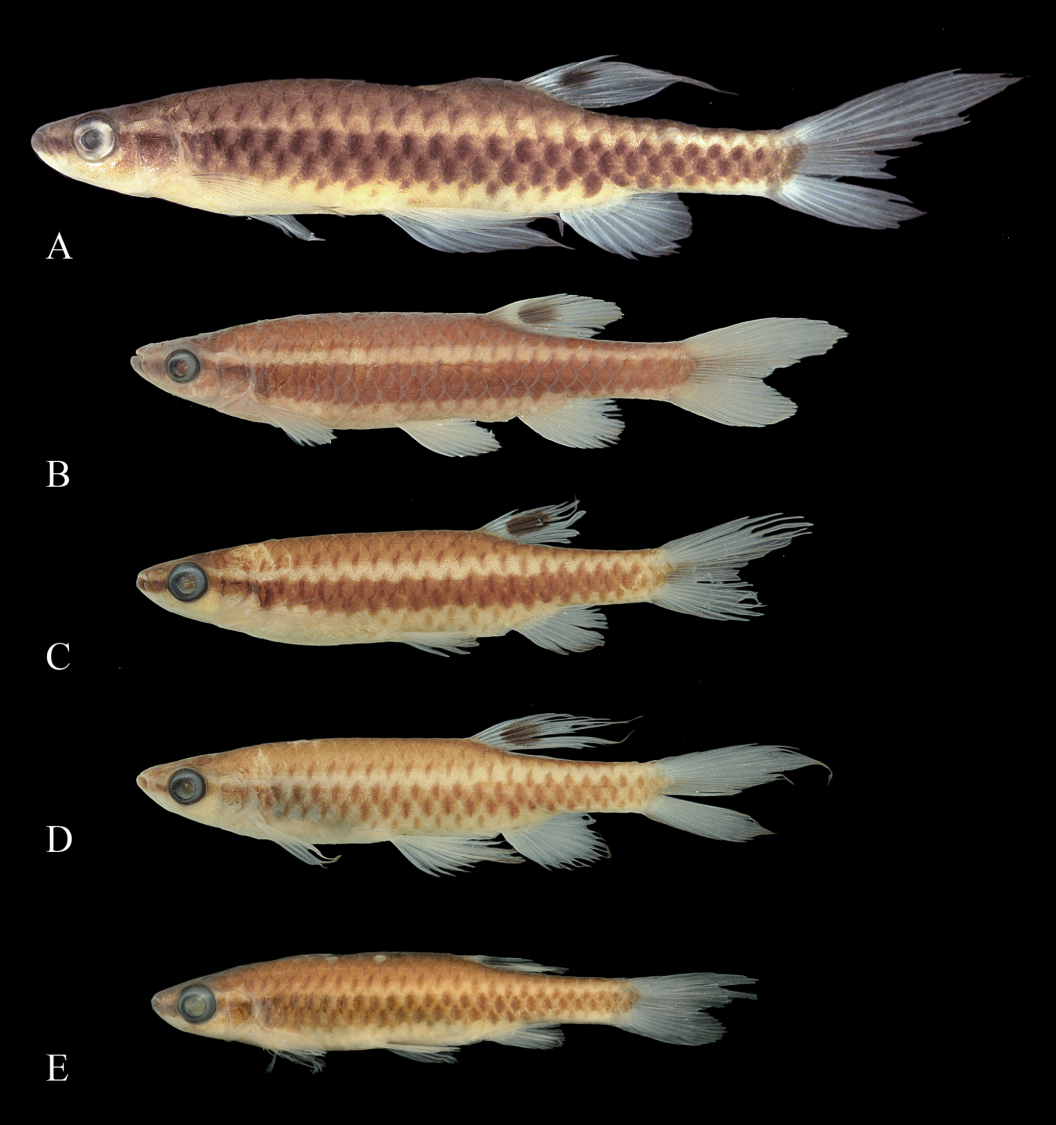
*Copella eigenmanni*. (A) male, MZUSP 81443, 50.5 mm SL, rio Tiquié, Amazonas, Brazil, (B) male, ICNMHN 1386, 37.5 mm SL, Maya, rio Meta, Colombia, (C) male ICNMHN 942, 32.3 mm SL, (D) male, ICNMHN 942, 36.3 mm SL, Cumaral, rio Meta, Colombia, (E), female, MZUSP 81143, 34.3 mm SL, rio Tiquié, Amazonas, Brazil.

**Fig 21:**
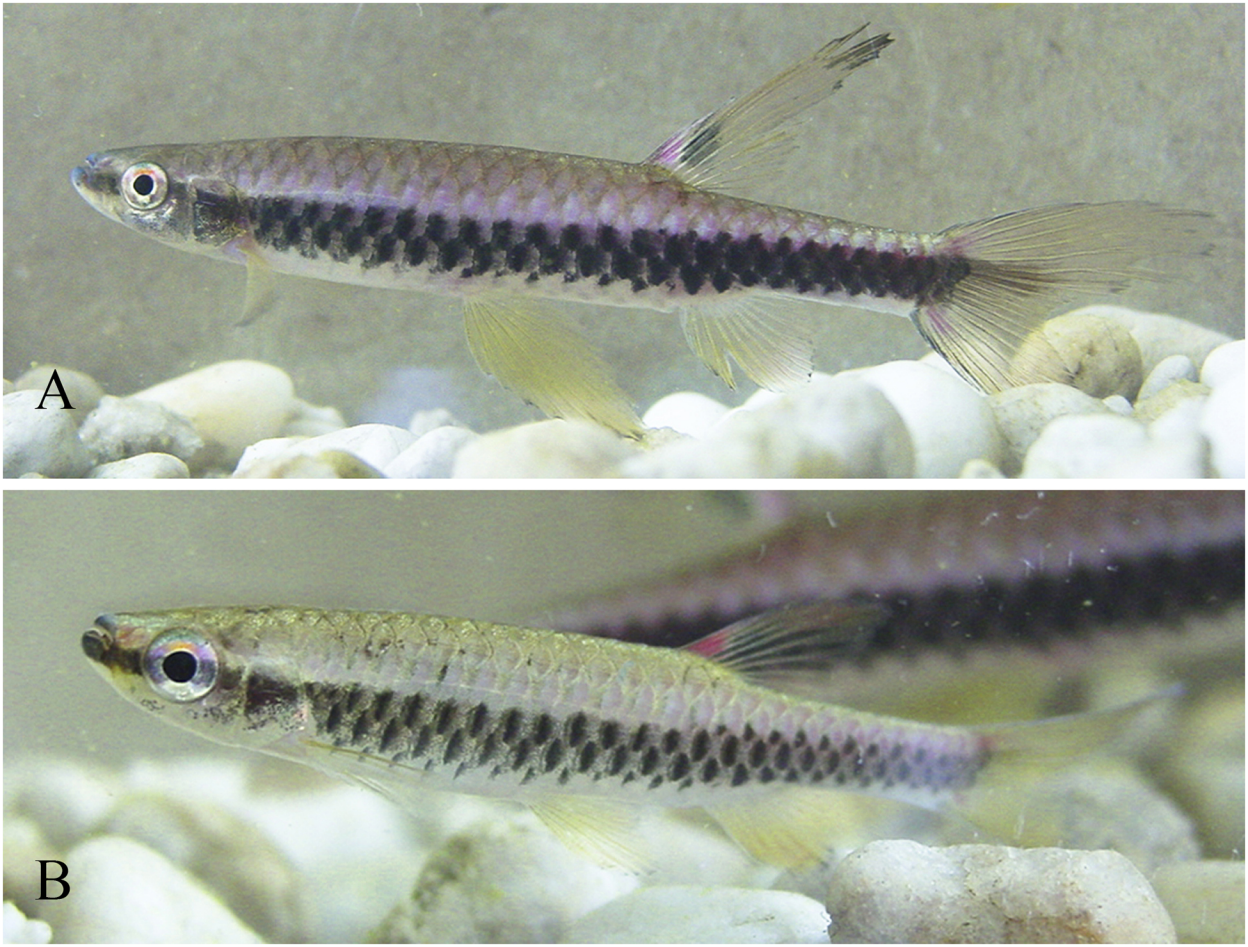
*Copella eigenmanni*, live specimens. MZUSP 81443, rio Tiquié, Amazonas, Brazil. (A) male, approximately 50 mm SL, (B) juvenile. Photo: A. Cabalzar & F. Lima.

**Fig 22:**
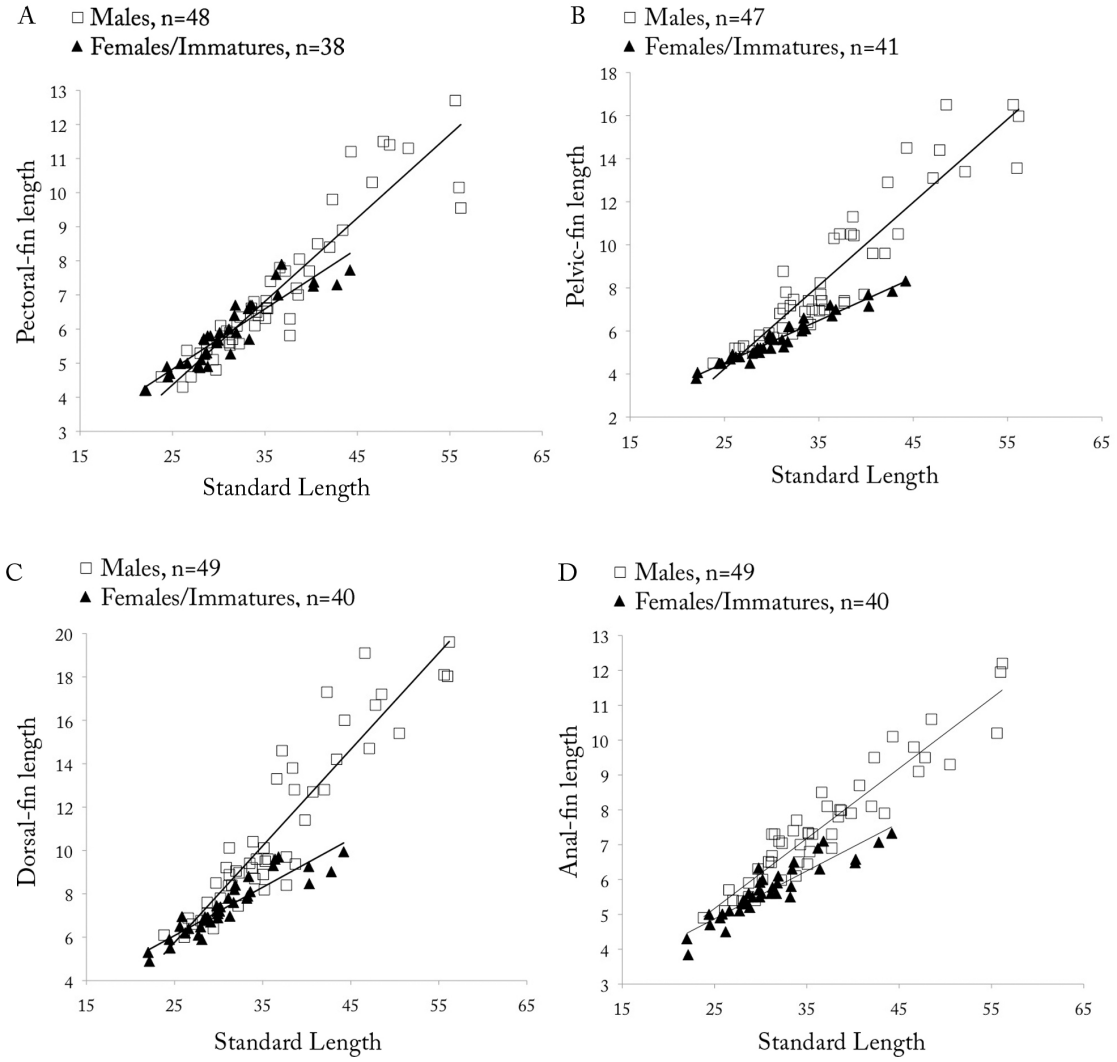
*Copella eigenmanni*. (A) pectoral-, (B) pelvic-, (D) dorsal-, and (A) anal-fin length as a function of SL by sex.

Figs 1, 15, 19–22; Tables 7 and 8

**Table 7:** Morphometrics of *Copella eigenmanni*.

	*Copeina eigenmanni*	*Copeina metae*	n	Range			Mean	SD
	Lectotype	Holotype						
Standard length (mm)	37.7	28.8	88	22.0	-	56.2	33.1	
**Percents of standard length**								
Body depth	18.5	20.1	88	14.3	-	21.7	18.6	1.2
Dorsal- to caudal-fin origin	34.8	35.4	88	32.9	-	39.1	36.2	1.2
Snout to dorsal-fin origin	65.7	64.6	88	61.1	-	67.3	64.6	1.5
Snout to pectoral-fin origin	23.0	23.3	88	19.7	-	26	22.8	1.1
Snout to pelvic-fin origin	46.6	46.9	87	45.7	-	52.6	48.5	1.3
Snout to anal-fin origin	73.2	70.8	88	68.1	-	74.5	71.2	1.4
Pectoral- to pelvic-fin origin	27.4	25.3	87	16.2	-	32.3	26.4	1.7
Pelvic- to anal-fin origin	27.1	23.3	87	17.6	-	26.1	23.3	1.4
Pectoral-fin length males	15.4	-	47	16.3	-	25.2	19.6	2.0
Pectoral-fin length fem/imm	-	17.0	37	16.8	-	21.5	19.1	1.1
Pelvic-fin length males	19.4	-	46	18.1	-	34.1	23.4	4.3
Pelvic-fin length fem/imm	-	18.1	40	16.3	-	19.9	18.4	0.8
Dorsal-fin length males	22.2	-	48	21.6	-	41.1	29.4	4.8
Dorsal-fin length fem/imm	-	23.6	39	20.9	-	26.9	24	1.5
Anal-fin length males	18.4	-	48	18.1	-	23.4	20.5	1.5
Anal-fin length fem/imm	-	18.1	39	16.1	-	21.3	18.5	1.2
Anal-fin base length	10.8	8.3	88	6.2	-	11.0	9.0	0.8
Caudal peduncle depth	9.6	10.4	88	8.2	-	11.7	9.6	0.6
Caudal peduncle length	23.6	20.8	88	17	-	22.8	19.7	1.4
Head length	22.0	24.3	88	18.4	-	26.7	23.4	1.2
**Percents of head length**								
Eye diameter	29.3	34.3	88	25.6	-	39	32.2	3.2
Snout length	32.2	28.6	88	24.6	-	45.6	31.6	5.3
Interorbital distance	35.5	-	88	28.8	-	50.6	36.1	3.5
Upper jaw length	31.9	25.7	88	25.1	-	37.9	31.9	2.6

Lectotype of *Copeina eigenmanni* BMNH 1869.7.25.6, holotype of *Copeina metae* CAS 60494, paratypes of *C*. *metae* CAS 60495 (10) and non-type material FMNH 55166 (2), FMNH 103819 (10), FMNH 159182 (8), ICN 1385 (4), ICN 12192 (2), MBUCV 11336 (10), MHNG 2200.028 (7), MZUSP 81443 (10), MZUSP 85149 (10), USNM 269898 (2), USNM 269899 (7), USNM 272409 (4), and USNM 272412 (2), n = number of specimens, SD = Standard deviation. Range does not include primary types.

**Table 8:** Meristics of lectotype of *Copeina eigenmanni* BMNH 1869.7.25.6 and holotype of *Copeina metae* CAS 60494.

	*Copeina eigenmanni*	*Copeina metae*
	Lectotype	Holotype
Dorsal-fin rays	ii8	ii8
Pectoral-fin rays	i9	-
Pelvic-fin rays	i7	i7
Anal-fin rays	iii9	iii9
Caudal-fin rays	-	i8,7i
Predorsal scales	15	15
First longitudinal scale row	15	-
Fourth longitudinal scale row	25	23
Longitudinal scale rows dorsal to pelvic	6	6
Longitudinal scale rows dorsal to anal	5	5
Circumpeduncular scale rows	10	-
Total vertebrae	-	34

*Copeina eigenmanni* Regan [20]: 393 [in part, from Bogotá; type locality: Bogotá (Colombia)].—Eigenmann [51]: 233 [specimens from Bogotá placed as synonym of *Copeina* (= *Copella*) *metae*].

*Copeina metae* Eigenmann [52]: 229 [type locality: Barrigona, rio Meta, Colombia].—Eigenmann [51]: 233: pl. XX, fig 3 [*Copeina* (= *Copella*) *eigenmanni* from Bogotá placed as synonym].—Myers [21]: 111 [comparison with *C*. *compta*].—Fowler [53]: 2 [rio Meta at Villavicencio; listed].—Böhlke [49]: 23 [type catalog].

*Copella metae*.—Géry [5]: 143 [new combination].—Taphorn [54]: 465, figs 295 and 296 [rio Aguaro; brief description].—Weitzman & Weitzman [1]: 242 [literature compilation; comments on misidentifications regarding *C*. *nigrofasciata* (= *Copella callolepis*)].—Zarske & Géry [46]: 13, fig 5 [photo of one paratype SU 24656, wrongly labeled as “Syntypus. CAS 124 656”; comparison with *Pyrrhulina zigzag*].—Zarske & Géry [7]: 44 [identification key].—Galvis *et al*. [55]: 166, fig 194 a e b [streams near Villavicencio and Puerto Gaitán; brief description].—Maldonado *et al*. [56]: 122 [rio Tomo, Colombia; listed].—Maldonado *et al*. [48]: 183 [literature compilation].—Urbano-Bonilla *et al*. [57]: 158 [Casanare basin, Colombia; listed].

*Copella compta*.—Galvis *et al*., [58]: 180, fig 214 [rio Vaupés and caño Mitúceño surrounding Mitú; misidentification; brief description].

*Copella* cf. *compta*.—Galvis *et al*., [55]: 165, fig 193 [near Puerto Inírida; brief description].

*Copella eigenmanni*.—Zarske [35]: 29, figs 21–24 [lectotype designation from Bogotá; redescription; taxonomic notes].

**Diagnosis.** *Copella eigenmanni* can be distinguished from all congeners by having the middle caudal-fin rays dark (*vs*. hyaline or with a small dark patch at base of middle caudal-fin rays on *Copella callolepis*). Additionally, it can be distinguished from *C*. *arnoldi* by having the procurrent caudal-fin rays black (*vs*. hyaline), from *C*. *compta* by having 14–15 scales on the first longitudinal scale row (*vs*. 16–17), from *C*. *nattereri* by the absence of a black spot on the posterior portion of each body scale (*vs*. presence), from *C*. *callolepis* by the absence of a series of conspicuous clear spot on each scale of the fourth longitudinal scale row (*vs*. presence), from males of *C*. *vilmae* by the absence of discontinuous longitudinal series of dark scales on body (*vs*. continuous), and from females of *C*. *vilmae* by the absence of a small dark spot at the base of the upper caudal-fin lobe (*vs*. presence).

**Description.** Morphometrics in Table 7 and meristics of types in Table 8. Largest examined male 56.4 mm SL, female 44.2 mm SL. Greatest body depth slightly anterior to vertical through pelvic-fin origin. Body cylindrical, slightly compressed laterally. Dorsal profile of body straight to slightly convex from tip of snout to the end of supraoccipital, straight to slightly convex from that point to dorsal-fin origin, posteroventrally inclined along dorsal-fin base, and straight along caudal peduncle. Ventral profile of body convex from anterior tip of dentary to vertical through anterior margin of orbit, straight from that point to vertical through pectoral-fin origin, slightly convex from that point to pelvic-fin origin, straight from pelvic-fin origin to anal-fin origin, posterodorsally inclined along anal-fin base and straight along caudal peduncle.

Mouth upturned. Premaxillary teeth in one row, with 14 (1), 15 (1), 17 (2), 19 (1), 20 (2), or 21 (1) teeth, decreasing in size laterally. Maxilary teeth sexually dimorphic, 13 (3), 14 (1), or 20 (1) teeth in males, 7 (2), or 9 (1) teeth in females, decreasing in size laterally, especially in males. Dentary teeth in two rows, outer with 9 (2), 10 (2), 11 (1) 12 (2), or 13 (1) teeth, increasing in size laterally, inner with 24 (1), 25 (1), 26 (1), 27 (1), 28 (1), 30 (1), 33 (1), or 42 (1) teeth, decreasing in size laterally (Fig 1).

Dorsal fin with ii (90), 7 (1) or 8* (89) rays, second and third branched rays longer. Pectoral fin with i (89), 8 (6), 9* (57), 10 (25), or 11 (1) rays, second and third branched rays longer, not reaching pelvic-fin origin. Pelvic fin with i (91), 7* (89) or 8 (2) rays, third or fourth branched rays longer. Anal fin with iii (3), 8,i* (91) rays, third and fourth branched rays longer. Adipose fin absent. Caudal fin with i (86), 7 (15), 8 (70), or 9 (1) rays in upper lobe, first three branched rays longer, and 6 (4), 7 (81), or 8 (1), i (86), rays in lower lobe, second and third branched rays longer. Upper caudal-fin lobe longer than lower. Relative fin lengths variable among males, specimens of same size with distinct fin lengths (Fig 22).

Predorsal scales 14 (19), 15* (68), or 16 (4), in one series. First longitudinal scale row with 14 (40) or 15* (49) scales. Fourth longitudinal scale row with 22 (2), 23 (18), 24 (45), or 25* (25) scales. Lateral line not pored, first lateral line scale with small canal medially on its anterior portion, without lateral opening. Longitudinal scale rows between dorsal-fin origin and pelvic-fin origin 5 (28) or 6* (66). Longitudinal scale rows between dorsal-fin origin and anal-fin origin 5* (91). Circumpeduncular scale rows 10* (91). Total number of vertebrae 33 (2), 34 (9), or 35 (1).

**Color in alcohol.** Overall ground coloration of body beige to brown. Dark stripe from anterior tip of dentary to posterior tip of opercle. Dorsal portion of body dark. Thin dark stripe at predorsal region. Anterior portion of scales of third to sixth longitudinal scale rows dark, with apparently subjacent dark coloration on posterior portion of scales on fourth and fifth longitudinal scale rows of variable intensity, frequently forming conspicuous longitudinal dark band (Fig 20). Some specimens with longitudinal conspicuous clear stripe at third longitudinal scale row, contrasting with conspicuous dark band on fourth and fifth longitudinal scale rows, from opercle to caudal peduncle, (Fig 20B); some specimens with clear stripe on third longitudinal scale row and dark spots on anterior portion of scales of third to sixth longitudinal scale rows (Fig 20D and 20E); and some individuals with intermediate color pattern, with clear stripe on third longitudinal scale row, dark spots on anterior portion of third to sixth longitudinal scale rows, and subjacent, not conspicuous, dark band on fourth and fifth longitudinal scale rows (Fig 20A and 20C). Ventral region of body clear. Dorsal and ventral procurrent caudal fin rays black. Dorsal fin with black round spot; some males with tip of dorsal fin dark. Pectoral, pelvic, anal, and caudal fins hyaline; distal profile of pelvic and anal fins usually dark. Middle caudal fin rays dark, fading toward tips of rays.

**Color in life.** Dorsal portion of body gray. Upper portion of eye orange. Body with variable color pattern (see Color in Alcohol). Specimens with longitudinal black band on fourth to fifth longitudinal scale rows with conspicuous metallic gray to purple band above it, on third row, and metallic green coloration mid-dorsally on caudal peduncle (Fig 21A); specimens without longitudinal black band with lighter metallic grey to purple band on third row, and red pigmentation on fourth and fifth longitudinal scale rows on caudal peduncle (Fig 21B). Fins yellow to orange. Pink spot at dorsal-fin base and upper and lower portions of caudal fin lobes.

**Sexual dimorphism.** Males longer than females. Males with more numerous maxillary teeth than females (see description above). Pectoral, pelvic, dorsal, and anal fins only slightly longer in males than in females. Tip of adpressed pelvic fin reaching to base of last branched anal-fin ray in males, and up to anus in females. Tip of adpressed dorsal fin reaching to caudal-fin base in males, and approximately up to one-half length of caudal peduncle in females. Upper caudal-fin lobe longer than lower one, especially in males. No sexual dimorphism in color pattern in *Copella eigenmanni*.

**Distribution.** Rio Orinoco basin, Colombia and Venezuela; upper rio Negro, Brazil and Venezuela and upper rio Putumayo drainage, Colombia, rio Amazonas basin (Fig 15).

**Remarks.** The original description of *Copella eigenmanni* was based on specimens designated as syntypes from several localities: Pará, Brazil, Guyana, and Bogotá, Colombia (probably collected near Villavicencio on the rio Meta, according to Weitzman & Weitzman [1]). Regan [20] mentioned color differences among specimens from Brazil, Guyana, compared to those from Colombia: “In the smaller examples there is sometimes an indistinct dusky band on the anterior part of the body and an indication of a pale stripe above the dark one on the head. In the larger ones, from Bogotá, a silvery stripe from eye to caudal fin separates a broad dark band below from the dark colour of the back”, but considered all the same species. Eigenmann [52] described *Copeina metae* from the rio Meta and stated: “Regan records presumably this species from Bogotá [in reference part of the syntypes of *C*. *eigenmanni*]. His specimens were probably collected in the Meta, and some at least of those reported from Bogotá represent the present species [= *C*. *metae*]”. Our findings agree with Eigenmann’s conclusion that *Copeina metae* is conspecific with the type specimens of *C*. *eigenmanni* from Bogotá.

Zarske [35] designated as lectotype for *Copella eigenmanni* a specimen from Bogotá (Fig 19A), and by doing so synonymyzed *Copella metae* with *C*. *eigenmanni* and kept the old catalog number (BMNH 1869.7.25.6–7) for the lectotype, but apparently did not pay attention to the fact that the original lot was represented by two specimens which should be considered respectively lectotype and paralectotype. This situation is now resolved with designation of BMNH 1869.7.25.6 for the lectotype and consequently BMNH 1869.7.25.7 for paralectotype. Zarske (2011; pags. 24 and 34) tentatively identified paralectotypes from Brazil and Guyana as *C*. *arnoldi* and *C*. *carsevennensis*, respectively. These are herein recognized as belonging to *C*. *arnoldi*.

Eigenmann [52] described *Copeina metae* based on the holotype (IU 13521 a) (Fig 19B) and 34 paratypes (IU 13521). Presently, the holotype is under catalog number CAS 60494 and only 26 paratypes are under CAS 60495 and two under SU 246526. The lots CAS 69238 (6 specimens) and FMNH 55166 (4 specimens) have the same data of the type material, however, according to D. Catania and M. Rogers, there is no further information that could indicate they are type material. Therefore, eight paratypes of *Copeina metae* remain missing.

Ortega and Vari [45] cited *Copella metae* for Peru but its occurence in Peru could be not confirmed. Only *C*. *callolepis* is recorded in that country.

**Material examined of *Copella eigenmanni* in** S1 Appendix.

### *Copella nattereri* (Steindachner, 1876)

**Fig 23:**
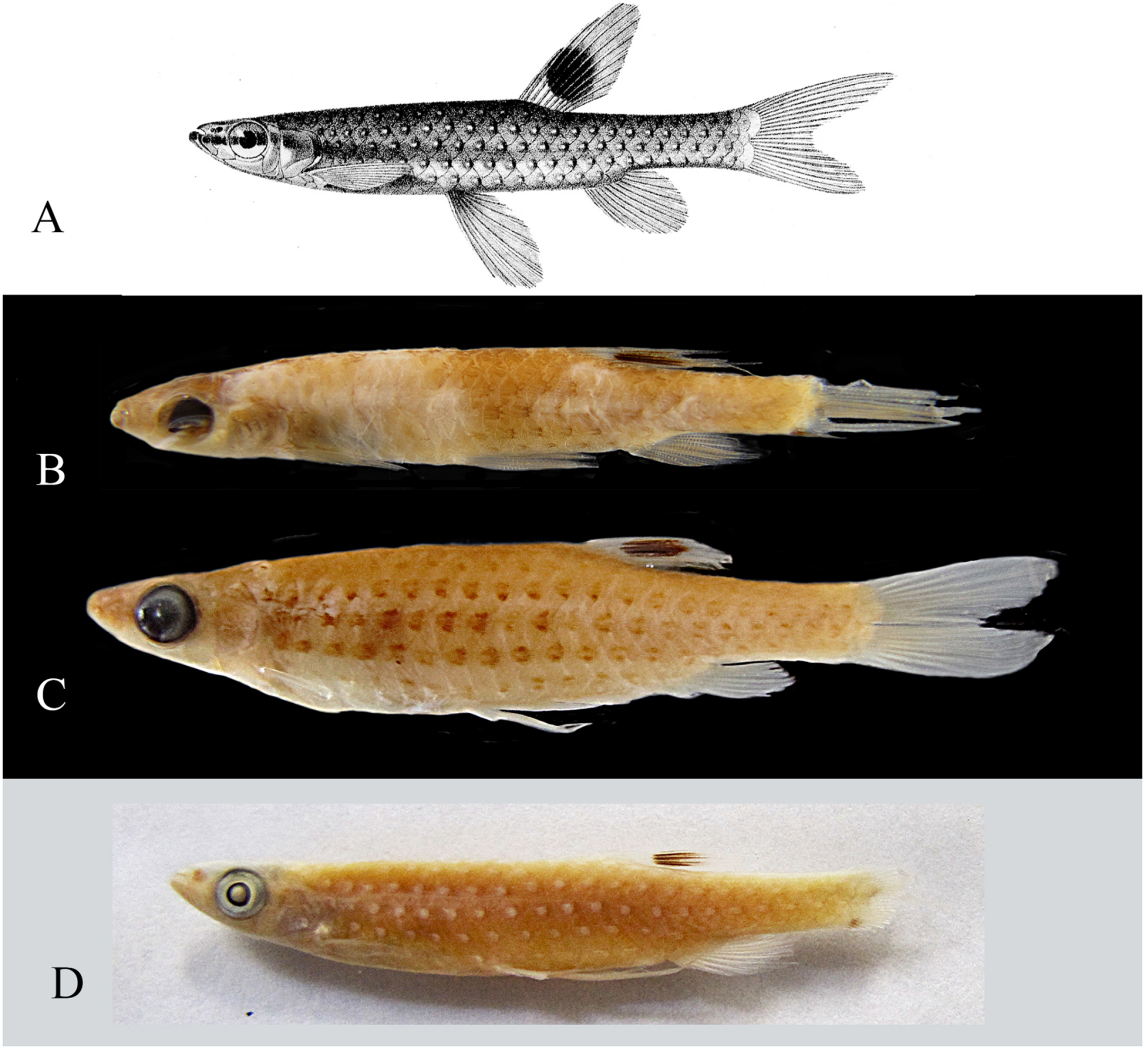
*Copella nattereri*. (A) plate II, fig 5 of Steindachner (1876), (B) Lectotype of *Pyrrhulina nattereri*, NMW 95055, male, 33.7 mm SL, Óbidos, Pará, Brazil, (C) holotype of *Copella meinkeni*, MTD F 30587, probably female, 40.6 mm SL, clear water stream on Southern Western bank of rio Negro about 5 km downstream Novo Airão, Amazonas, Brazil, (D) paratype of *Copella meinkeni*, NMW 56973 (33.0 mm SL), Codajás and Tabatinga, Brazil.

**Fig 24:**
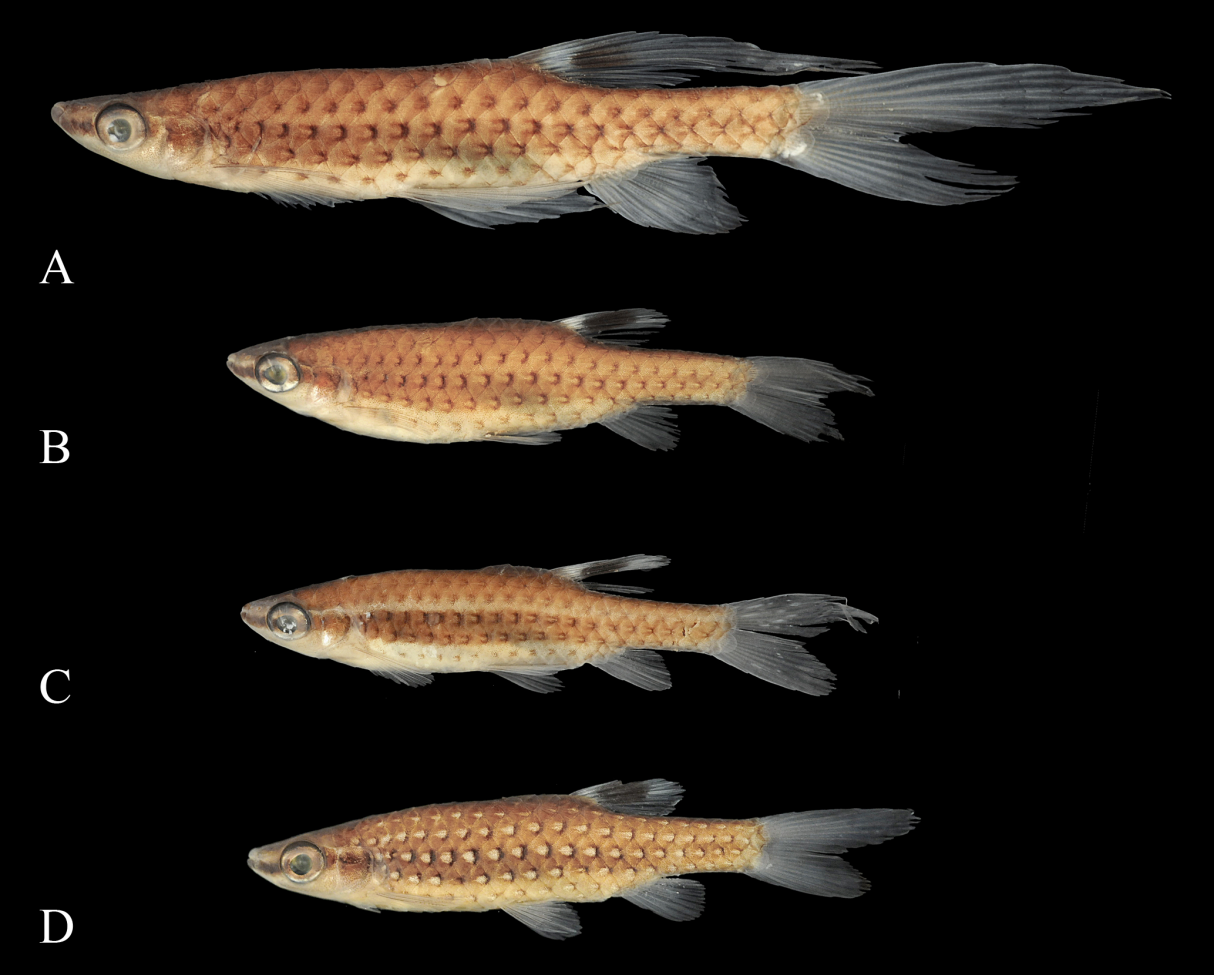
*Copella nattereri*. MZUSP 87426, (A) male, 39.3 mm SL, (B) female, 27.3 mm SL, (C) female, 25.7 mm SL, Rio Preto da Eva, Amazonas, Brazil, (D) MPEG 15913, female, 27.3 mm SL, rio Madeira, Maués, Amazonas, Brazil.

**Fig 25:**
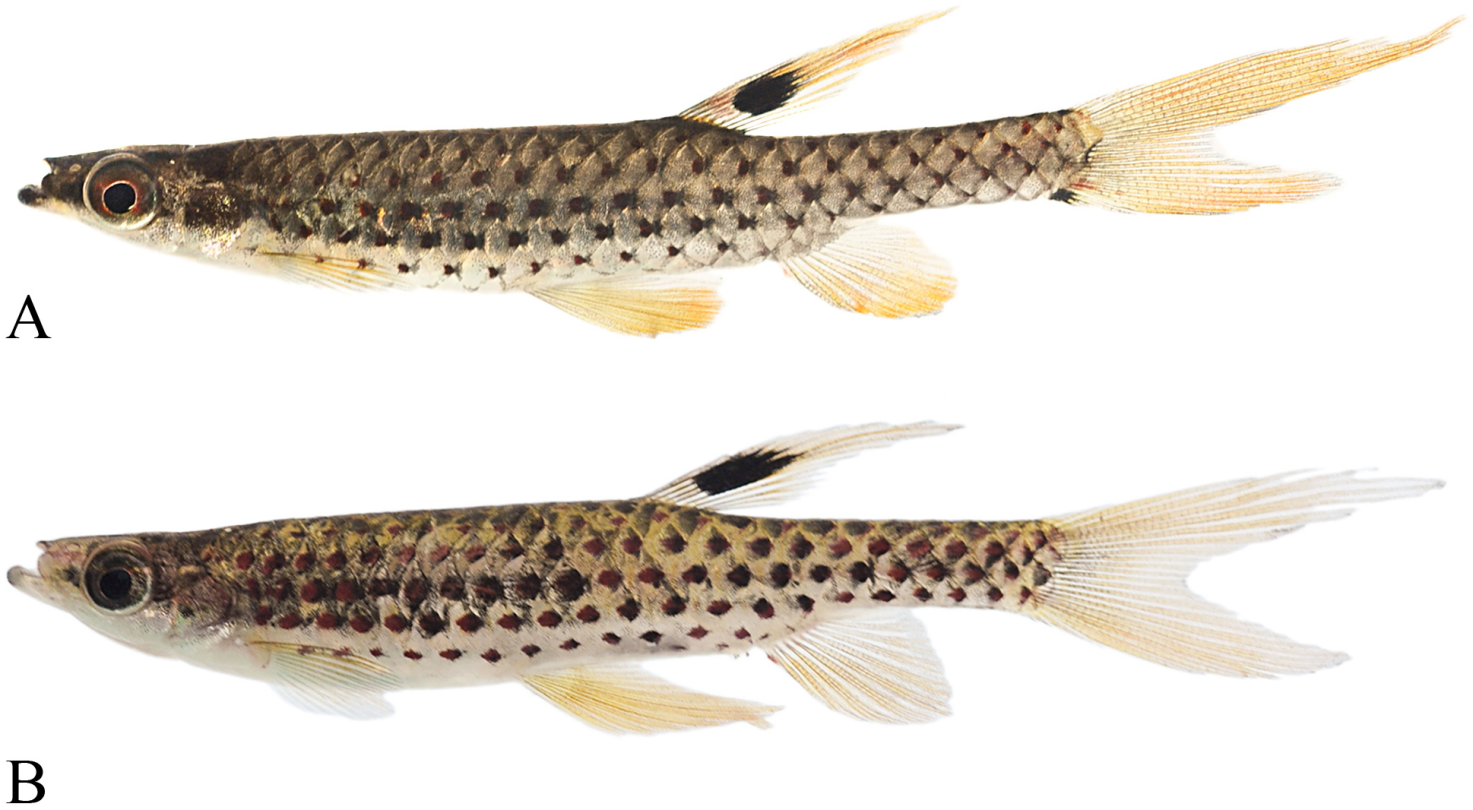
*Copella nattereri*, freshly-collected specimens. MZUSP 109514 a) male not preserved b) male, 35.8 mm, rio Negro, Santa Isabel do Rio Negro, Amazonas, Brazil.

**Fig 26:**
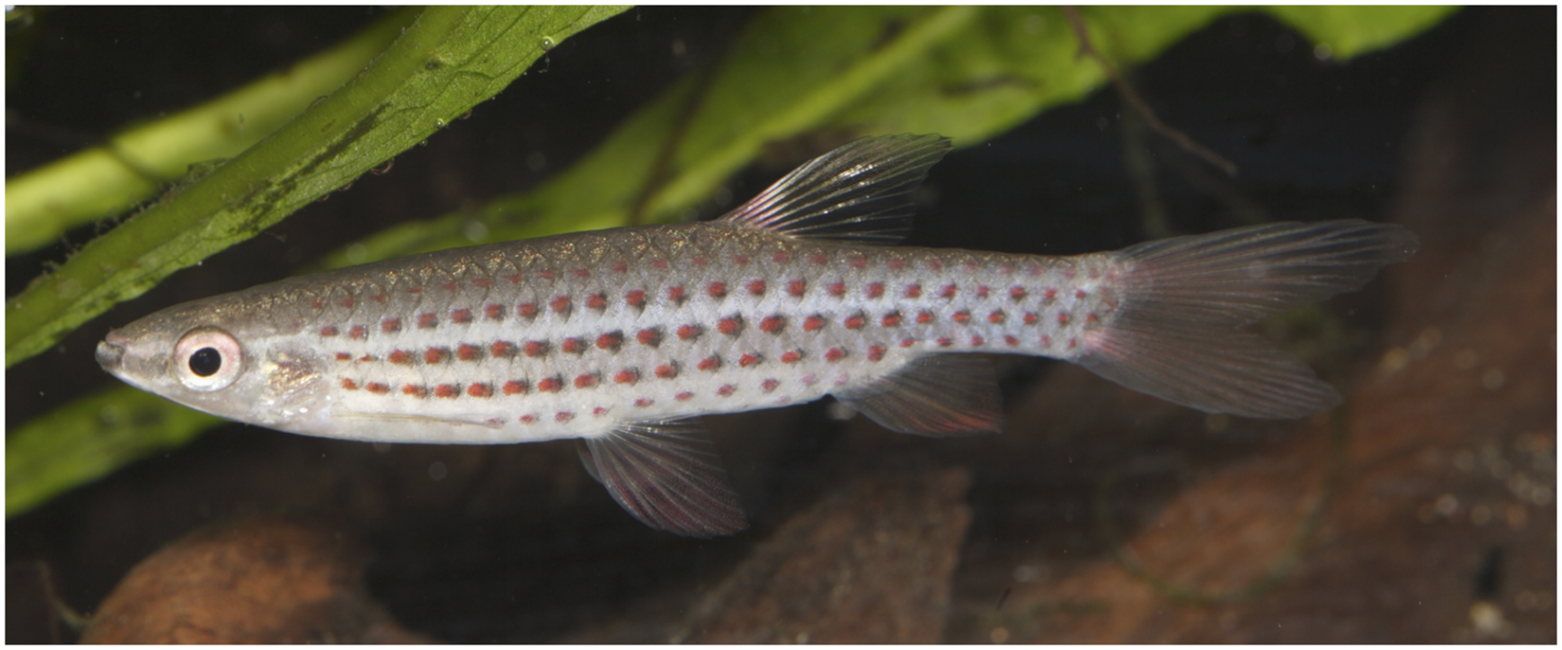
*Copella nattereri*, live male specimen not preserved. Reprinted from belowwater.com under a CC BY license, with permission from Oliver Lucanus, original copyright 2017.

**Fig 27:**
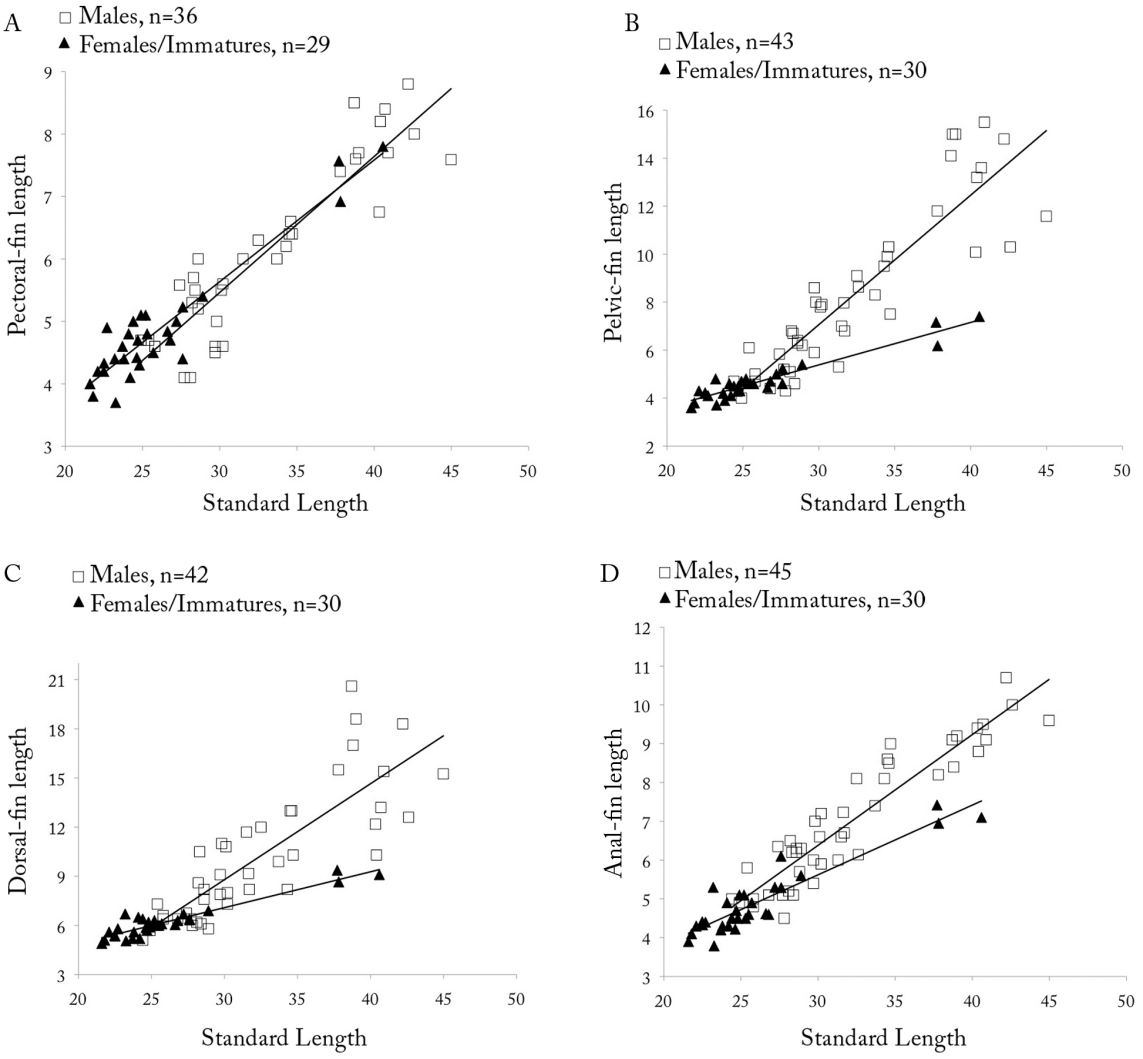
*Copella nattereri*. (A) pectoral-, (B) pelvic-, (C) dorsal-, and (D) anal-fin length as a function of SL by sex.

Figs 10, 23–27; Tables 9 and 10

**Table 9:** Morphometrics of *Copella nattereri*.

	*Pyrrhulina nattereri*	*Copella meinkeni*	n	Range		Mean	SD
	Lectotype	Holotype					
Standard length (mm)	33.7	40.6	73	21.6	45.0	30.2	
**Percents of standard length**							
Body depth	15.6	20.6	73	14.0	22.9	18.7	1.7
Dorsal- to caudal-fin origin	33.8	38.1	73	35.1	63.3	38.4	3.3
Snout to dorsal-fin origin	63.7	62.1	73	36.6	66.3	62.1	3.4
Snout to pectoral-fin origin	24.7	24.6	73	21.6	26.7	24.1	1.1
Snout to pelvic-fin origin	48.6	50.6	73	24.4	51.9	48.8	3.3
Snout to anal-fin origin	72.9	74.8	73	23.0	76.4	71.4	6.0
Pectoral- to pelvic-fin origin	22.9	25.1	73	21.5	28.5	25.6	1.6
Pelvic- to anal-fin origin	23.1	25.9	73	21.6	26.7	24.2	1.2
Pectoral-fin length males	17.8	-	35	14.6	22.1	18.5	1.8
Pectoral-fin length fem/imm	-	17.5	28	15.8	21.8	18.7	1.3
Pelvic-fin length males	24.7	-	41	15.5	38.6	25.1	6.5
Pelvic-fin length fem/imm	-	18.2	29	16.0	20.8	18.0	1.1
Dorsal-fin length males	29.4	-	40	20.2	53.3	30.8	8.0
Dorsal-fin length fem/imm	-	22.4	29	21.4	28.9	24.0	1.6
Anal-fin length males	21.9	-	44	16.3	25.9	21.6	2.3
Anal-fin length fem/imm	-	17.5	29	16.3	22.8	18.9	1.4
Anal-fin base length	9.4	8.0	73	6.7	10.3	8.8	0.7
Caudal peduncle depth	8.1	9.9	73	7.1	10.8	9.3	0.7
Caudal peduncle length	20.0	17.7	73	17.1	21.6	19.5	0.9
Head length	24.0	25.4	73	21.2	26.0	23.9	1.2
**Percents of head length**							
Eye diameter	33.5	36.8	72	30.1	42.6	35.6	2.5
Snout length	31.5	33.2	72	25.0	33.3	29.7	2.0
Interorbital distance	34.6	36.4	72	33.7	41.9	38.0	2.0
Upper jaw length	27.5	29.7	70	21.8	35.2	28.0	2.4

Lectotype of *Pyrrhulina nattereri* NMW 95055, holotype of *Copella meinkeni* MTD F 30587, paralectotypes of *P*. *nattereri* NMW 57148 (1), NMW 56974 (3), paralectotype of *C*. *callolepis* BMNH 1909.4.2.28 (1), paratypes of *C*. *meinkeni* MHNG 2577.32 (1), MTD F 30588–30592 (5), and non-type material ANSP 191389 (3), ICN 16375 (3), ICN 16891 (2), MCZ 6259 (10), MHNG 2577.048 (5), MZUSP 63522 (3), MZUSP 63526 (3), MZUSP 63527 (2), MZUSP 66733 (4), MZUSP 66766 (8) MZUSP 87426 (10), MZUSP 109514 (6), and USNM 311005 (3), n = number of specimens, SD = Standard deviation. Range does not include primary types.

**Table 10:** Meristics of lectotype of *Pyrrhulina nattereri* NMW 95055 and holotype of *Copella meinkeni* MTD F 30587.

	*Pyrrhulina nattereri*	*Copella meinkeni*
	Lectotype	Holotype
Dorsal-fin rays	ii8	ii8
Pectoral-fin rays	i9	i9
Pelvic-fin rays	i7	i7
Anal-fin rays	iii9	iii9
Caudal-fin rays	-	i87i
Predorsal scales	14	13
First longitudinal scale row	-	13
Fourth longitudinal scale row	-	21
Longitudinal scale rows dorsal to pelvic	5	6
Longitudinal scale rows dorsal to anal	5	5
Circumpeduncular scale rows	-	10
Total vertebrae	32	-

*Pyrrhulina nattereri* Steindachner [59]: 8, pl. 2, fig 5 and 6a [in part, see Remarks; type locality: Óbidos, in a tributary of the rio Amazonas].—Eigenmann & Eigenmann [17]: 112 [in part, rio Trombetas and several localities in the rio Amazonas from Codajás to Óbidos, see discussion; brief description].—Regan [20] [Amazonas, brief description].—Fowler [60]: 263 [literature compilation].—Meinken [24]: 116 [comparison with *Pyrrhulina nigrofasciata* (= *Copella callolepis*)].

*Copeina nattereri*.—Eigenmann [61]: 428 [new combination; listed].

*Copeina callolepis* Regan [20]: 393 [in part, paralectotype BMNH 1909.4.2.28, type locality: Amazon].

*Copella nattereri*.—Myers [4] [new combination, *Copella callolepis* as possible synonym].—Géry [6]: 28 [comparison with *C*. *vilmae*; considers *Copella callolepis* conspecific with *C*. *nattereri*].—Géry [5]: 147 [brief description; unnumbered figs of pg. 144 (third) and 145].—Weitzman & Weitzman [1]: 242 [*Copeina callolepis* placed as synonym; literature compilation].—Arbeláez *et al*. [62]: 103 [rio Amazonas at Letícia; listed].—Bogotá-Gregory & Maldonado-Ocampo [47]: 47 [literature compilation].—Zarske & Géry [7]: 19, figs 2–3, 18 (lower specimen) [lectotype designation; photo of lectotype NMW 95055; redescription; taxonomic notes].—Galvis *et al*. [55]: 167, fig 195 [Puerto Inírida; brief description].

*Copella callolepis*.—Zarske & Géry [7]: 21, figs 6 [fig of paralectotype BMNH 1909.4.2.28].—Zarske [35]: 37–39, fig 31, 36 [revalidation].

*Copella* cf. *arnoldi*.—Bogotá-Gregory & Maldonado-Ocampo [63]: 64, Bogotá-Gregory & Maldonado-Ocampo [47]: 74, Maldonado-Ocampo *et al*. [48]: 183 [misidentification; listed].

*Copella vilmae*.—Mojica *et al*. [64]: 200, Bogotá-Gregory & Maldonado-Ocampo [47]: 74, Maldonado *et al*. [48] [rio Amazonas at Letícia; misidentification; listed].—Galvis *et al*., [58]: 180, figs 215 [misidentification; brief description].

*Copella meinkeni* Zarske & Géry [7]: 27, figs 12–15, 18 (upper specimen) [type locality: rio Negro at Novo Airão; identification key].—Zarske [35]: 38, fig 32 [taxonomic comment].

*Copella* spec. aff. *meinkeni*.—Zarske & Géry [35]: 31, fig 1, 16–17, 21, 26 [rio Tapajós, rio Amazonas at Santarém and Rio Preto da Eva, rio Negro, description; identification key].

**Diagnosis.** *Copella nattereri* can be distinguished from all congeners by the presence of a dark spot on each scale of the flank (*vs*. absence). It can be further distinguished from all the species, except *Copella callolepis*, by having clear spots (red to purple in life) on the scales of the flank, (*vs*. absence). It is further distinguished from *C*. *callolepis* by the absence of a conspicuous black longitudinal band (*vs*. when present, longitudinal band dusky and not conspicuous).

**Description.** Morphometrics in Table 9 and meristics of types in Table 10. Largest examined male 45.0 mm SL, female 40.5 mm SL. Greatest body depth slightly anterior to vertical through dorsal-fin origin. Body cylindrical, slightly compressed laterally. Dorsal profile of body straight to slightly concave from tip of snout to end of supraoccipital, straight to slightly convex from that point to dorsal-fin origin, posteroventrally inclined along dorsal-fin base and straight along caudal peduncle. Ventral profile of body convex to posteroventrally inclined from anterior tip of dentary to vertical through anterior margin of orbit, straight from that point to vertical through pectoral-fin origin, slightly convex from that point to pelvic-fin origin, straight from pelvic-fin origin to anal-fin origin, posterodorsally inclined along anal-fin base and straight along caudal peduncle.

Mouth upturned. Premaxillary teeth in one row, with 15 (1), 16 (1), 18 (1), 19 (1), 20 (1), 21 (1), 22 (1), or 24 (1) teeth, decreasing in size laterally. Number of maxillary teeth sexually dimorphic, 11 (1), 12 (1), 13 (1), 14 (1), or 20 (1) in males, 6 (1), 7 (1), or 8 (1) in females, decreasing in size posteriorly, especially in males. Dentary teeth in two rows, outer with 8 (1), 10 (3), 11 (3), or 12 (1), increasing in size laterally, inner with 24 (3), 27 (1), 30 (1), 31 (2), or 34 (1) teeth, decreasing in size laterally.

Dorsal fin with ii (75), 7 (1) or 8* (74) rays, second and third branched rays longer. Pectoral fin with i (56), 8 (3), 9* (22), or 10 (26) rays, first three branched rays longer, their tips never reaching pelvic fin. Pelvic fin with i (76), 7* (73) or 8 (3) rays, third branched ray longest. Anal fin with iii (5), 8,i* (74), or 9,i (1) rays, third and fourth branched rays longer. Adipose fin absent. Caudal fin with i (74), 7 (7), 8 (58), or 9 (1) in upper lobe, first and second branched rays longer, and 6 (2), 7 (67), or 8 (1), i (70) rays in lower lobe, second and third branched rays longer. Upper caudal-fin lobe longer than lower.

Predorsal scales 12 (6), 13 (42), or 14* (23). First longitudinal scale row with 11 (2), 12 (7), 13 (37), or 14 (24) scales. Fourth longitudinal scale row with 20 (2), 21 (14), 22 (35), or 23 (16) scales. Lateral line not pored, first lateral line scale with small canal medially on its anterior portion, without lateral opening. Longitudinal scale rows between dorsal-fin origin and pelvic-fin origin 5* (29) or 6 (43). Longitudinal scale rows between dorsal-fin origin and anal-fin origin 5* (71). Circumpeduncular scale rows 10* (70). Total number of vertebrae 32* (13), 33 (20), or 34 (5).

**Color in alcohol.** Overall ground coloration of body beige to brown. Dorsal portion of body dark, ventral clear. Dark stripe extending from anterior tip of dentary to posterior tip of opercle. Thin predorsal dark stripe, sometimes wider, with guanine deposition over second and third scales. Clear spot on posterior portion of scales of second or third to sixth longitudinal scale rows, limited dorsally, posteriorly and ventrally by dark pigmentation, usually horseshoe shaped. Scales with posterior border dark in some individuals (Figs 21A and 22A). Subjacent coloration of flank homogeneous (Fig 24A, 24B and 24D) or with clear stripe on third longitudinal scale row, contrasting with band of subjacent pigmentation, on fourth to fifth longitudinal scale rows of variable intensity, more conspicuous anteriorly (Fig 24C). Ventral region of body clear. Black round spot in dorsal fin, dorsal to small white spot. Remaining fins hyaline. Pelvic and anal fins often with dark edge, usually more intense in males. Dorsal and ventral procurrent caudal-fin rays hyaline or dark (Fig 25). Pigmentation of procurrent caudal-fin rays more conspicuous in males than females and usually absent in juveniles. Adult specimens with hyaline procurrent caudal-fin rays only found in rio Negro basin (Fig 25B).

**Color in life.** Dorsolateral portion of body gray, ventral portion beige. Distal portion of scales on second or third to sixth longitudinal scale rows with red to purple spots, limited dorsally, posteriorly, and ventrally by dark pigmentation. Dark longitudinal band on fourth and fifth longitudinal scale rows. Fins yellow to pale red or hyaline (Figs 25 and 26).

**Sexual dimorphism.** Males longer than females. Males with more numerous maxillary teeth than females (see description above). Pelvic, dorsal, and anal fin of males longer than in females (Fig 27). Length of pectoral fin apparently not dimorphic. Tip of adpressed pelvic fin reaching to base of last branched anal-fin ray in males, and to anterior border of anus in females. Tip of adpressed dorsal fin reaching to one-half length of middle caudal-fin rays in males, and approximately to one-half length of caudal peduncle in females. Tip of adpressed anal-fin reaching to level of first ventral procurrent rays in males, and up to two-thirds length of caudal peduncle in females (Fig 27). Upper caudal-fin lobe longer than lower, especially in males. No evidence of sexual dimorphism related to color pattern in this species.

**Distribution.** Rio Amazonas at Letícia, Colombia; rio Amazonas from mouth of rio Negro to mouth of rio Tapajós, Brazil; rio Negro basin, Brazil, Venezuela, and Guyana; upper and middle rio Orinoco basin, Venezuela (Fig 10).

**Remarks.** The description of *Pyrrhulina nattereri* by Steindachner [59] was based on syntypes from two localities: “Joh. Natterer collected the here-described species in many samples at the mouth of the Rio Negro and by Prof. Agassiz [= Thayer Expedition] at Óbidos in a tributary of the Amazon”. Possible types of *Pyrrhulina nattereri* are known to be deposited in the NMW, MSNG, ZMUC, and MCZ museums. Specimens deposited at NMW from the Thayer Expedition collected in Óbidos are undoubtedly type material (NMW 56974, 57148 and 95055). However, the lot NMW 56973 from Codajás and Tabatinga, that are questionably marked as type material of *Pyrrhulina nattereri*, cannot be considered type since the locality does not match with those mentioned in the original description. Zarske & Géry [7] listed this same lot (NMW 56973) as paratypes of *Copella meinkeni* (= *Copella nattereri*) (Fig 23D).

Samples deposited in the Genova and Copenhagen museums are said to be from the rio Amazonas with no further information. The material from Genova (MSNG 9239, 1 specimen), and Copenhagen (ZMUC P241264 and P241265, 2 specimens) were donated by Steindachner in 30 Oct 1880 (G. Doria personal communication) and in 1876 (J. Nielsen personal communication), respectively, and therefore they should be considered paralectotypes of *Pyrrhulina nattereri*. Regarding the 57 specimens (MCZ 6259) collected during the Thayer Expedition in Óbidos and deposited at MCZ, it is not clear whether Steindachner analyzed this material or not during his stay at MCZ from 1870–1871 (before he left to take part in Hassler’s expedition from 1871 to 1872, according to Borodin [65]). Since this material has not been specifically designated we find no reason to consider it as types.

Despite Zarske’s effort in searching the material from the mouth of the rio Negro, it was never found is considered as probably lost [7]. The lack of details on the origin of the samples deposited at MSNG and ZMUC raises the possibility that it could have come from that locality, but this was not confirmed.

In the present study, only types of *Copella nattereri* from the NMW museum could be examined. The lot NMW 56974 has five numbered paralectotypes. Numbers 1, 2, and 5 (28.8, 28.8, and 32.0 mm SL, respectively) represent, indeed, *C*. *nattereri*. Number 3, however, probably belongs to *C*. *callolepis* (28.9 mm SL) and number 4 (24.0 mm SL) is a specimen of *Copella* not precisely identified due to its poor condition. The lot NMW 57148 has four paralectotypes in poor conditions, the smallest (16.4 mm SL) is a *Nannostomus* sp. with a dark band on body and the remaining are *Copella nattereri*.

The topotypes under MCZ 6259 mentioned above as not representing type material is actually part of a mixture including other lots bearing MCZ numbers “6263+6300+6835+6836+6837”. Examination of the 78 specimens in this mixture revealed the presence of *Copella nattereri* (57 specimens, 18.3–30.7 mm SL), *C*. *callolepis* (9, 28.9–32.9 mm SL), *Copella* sp. (10, 17.3–28.1 mm SL), and *Pyrrhulina* sp. (2, 25.9–29.2 mm SL, in poor condition). The 57 specimens of *Copella nattereri* kept MCZ catalog number 6259 and the others received the numbers MCZ 170504, MCZ 170505, and 170506, respectively.

Steindachner [59] originally described *Pyrrhulina* (= *Copella*) *nattereri* based on the presence of a bright spot close to the posterior edge of the body scales, bordered with dark brown pigmentation, except for its anterior edge. Steindachner also mentioned that a weak dark longitudinal band is not rare between the third and the fourth longitudinal scale rows (Fig 23A). Zarske & Géry [7] chose a lectotype for *Copella nattereri* discussing its identity, stating that it does not correspond to the same species that has been traditionally referred to by aquarists as *Copella nattereri* (the “spotted tetra”), but to what has been named *Copella nigrofasciata* (= *Copella callolepis*), species that has a black longitudinal band and one to three conspicuous longitudinal rows of clear spots on body (Fig 12), and made a new available name for the spotted tetra, *Copella meinkeni* (Fig 23C). The analysis of the lectotype of *Pyrrhulina nattereri* revealed that it clearly has the same color pattern described by Steindachner, not the characteristic coloration of *Copella callolepis* (see the presence of rows of dark spots on body in Fig 23B). Therefore, *C*. *meinkeni* is considered junior synonym of *Copella nattereri*, the valid name for the spotted tetra.

According to Zarske [35], there are two species with a series of black spots on body scales, *C*. *meinkeni* (= *C*. *nattereri*) and *C*. *callolepis*, the former being larger and with hyaline procurrent caudal-fin rays, and the later being smaller, with a black mark on the ventral procurrent caudal-fin rays. According to the present study, there are only one species of *Copella* with series of black spots on body scales, which is *Copella nattereri* (with *C*. *meinkeni* as its junior synonym). The lectotype proposed by Zarske & Géry [7] for *C*. *callolepis* has a black longitudinal band on body and clear spots, not corresponding to the same species (see Remarks under *C*. *callolepis*). The presence of a black mark on the ventral procurrent caudal-fin rays, mentioned by Zarske [35] is herein considered variable within *Copella nattereri* (see Color in alcohol section). Although both conditions (presence and absence of a dark mark on the procurrent caudal-fin rays) are only found in the adult specimens from the rio Negro basin (Fig 25) (adults of the remaining localities where this species occurs have dark procurrent caudal-fin rays and juveniles from all the localities usually lack a dark mark on procurrent caudal-fin rays). Therefore, this feature was not found to be unambiguous to recognize a different species. Furthermore, dark procurrent rays can be even observed in some paratypes of *C*. *meinkeni* (NMW 56973: Fig 23D). No morphometric, meristic, osteological, or color features were found to justify considering more than one species in the populations of *C*. *nattereri* analyzed.

Zarske & Géry [7] listed “15 out of 30” specimens from the lot MHNG 2205.096 as paratypes of *Copella meinkeni*. Examination of this lot revealed there are actually a total of 41 specimens, a mixture of the 15 paratypes and other 26 non-types. Remaining type material of *Copella nattereri* and its synonym species that were not examined are: MSNG 9239 (1, Paralectotype of *Pyrrhulina nattereri*), rio Amazonas. ZMUC P241264 and P241265 (2, *Pyrrhulina nattereri*), rio Amazonas. MTD F 29454–29456, 3 paralectotypes of *C*. *meinkeni*. MTD F 17133–17136, 4 paralectotypes of *C*. *meinkeni*. Priv. Coll. Géry: 1076: 1–2.2006, 2 Ex paralectotypes of *C*. *meinkeni*, rio Trombetas.

Eigenmann & Eigenman [17] reported *Pyrrhulina* (= *Copella*) *nattereri* for several localities in the rio Amazonas from Codajás to Óbidos. The specific localities are: rio Trombetas, rio Amazonas at Villa Bella (= Parintins), Manaus, Silves, Lago Saracá, Lago Hyanauari (= Janauari), Codajás, and Jatuarana [probably near Parintins (Lima *et al*., 2003)]. Ulrey [66] listed *Pyrrhulina* (= *Copella*) *nattereri* for the lower Amazon, Brazil, but we had no opportunity to confirm his identification. Therefore, this locality is not included in the range of distribution.

**Material examined of *Copella nattereri* in** S1 Appendix.

### *Copella vilmae* Géry, 1963

**Fig 28:**
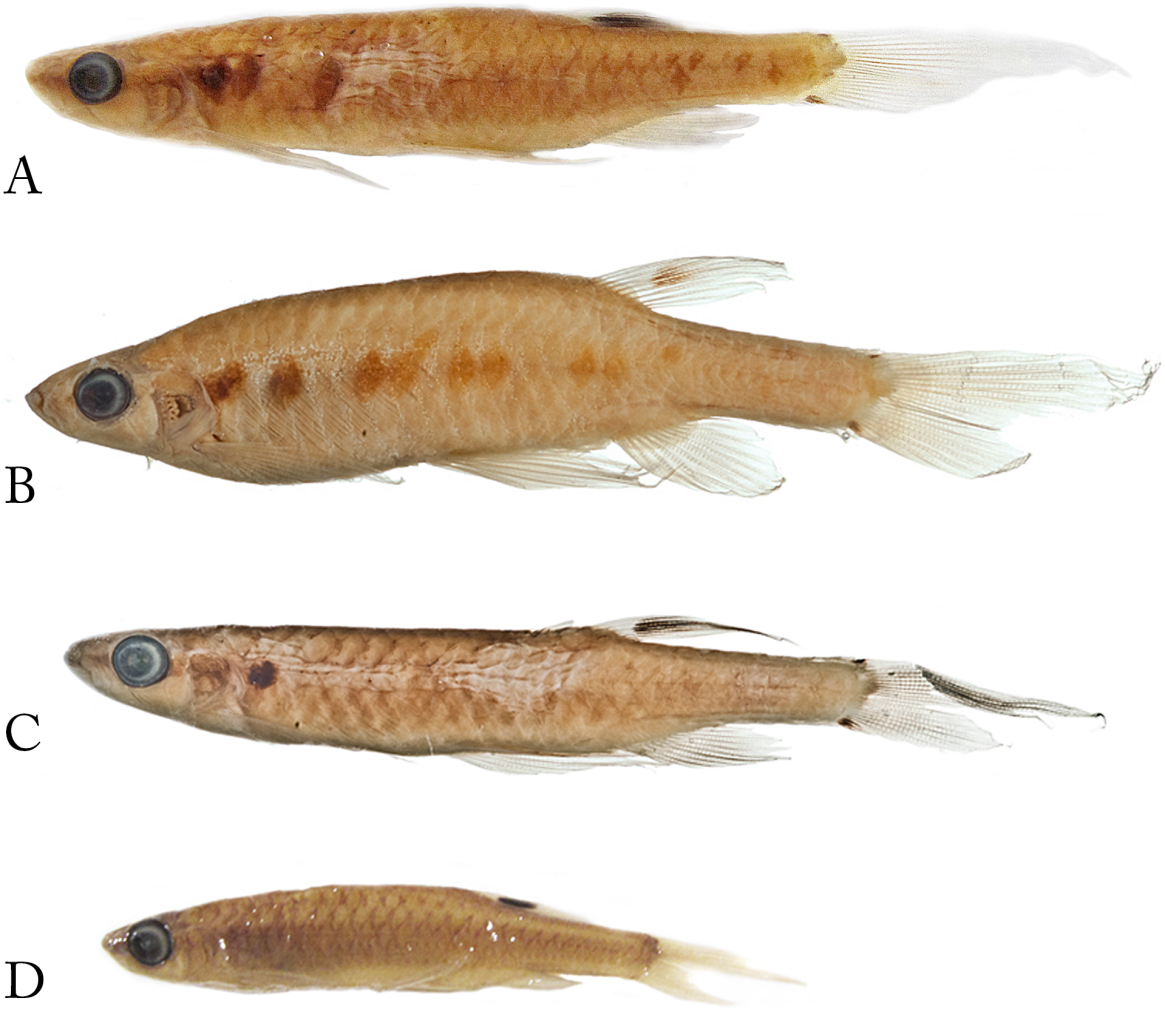
*Copella vilmae*. (A) holotype, male, SMF 5931, 43.9 mm SL, Igarapé Preto near Belém, 60 km downstream Letícia, Colombia, (B) paratype, male, USNM 198135, 46.4 mm SL, (C) USNM paratype, male, 45.4 mm SL, Igarapé Preto near Belém, 60 Km downstream Letícia, Colombia, (D) AMNH 218052, female, 30.5 mm SL, rio Amazonas at Letícia, Colombia. Photos A and B by Sandra Raredon.

**Fig 29:**
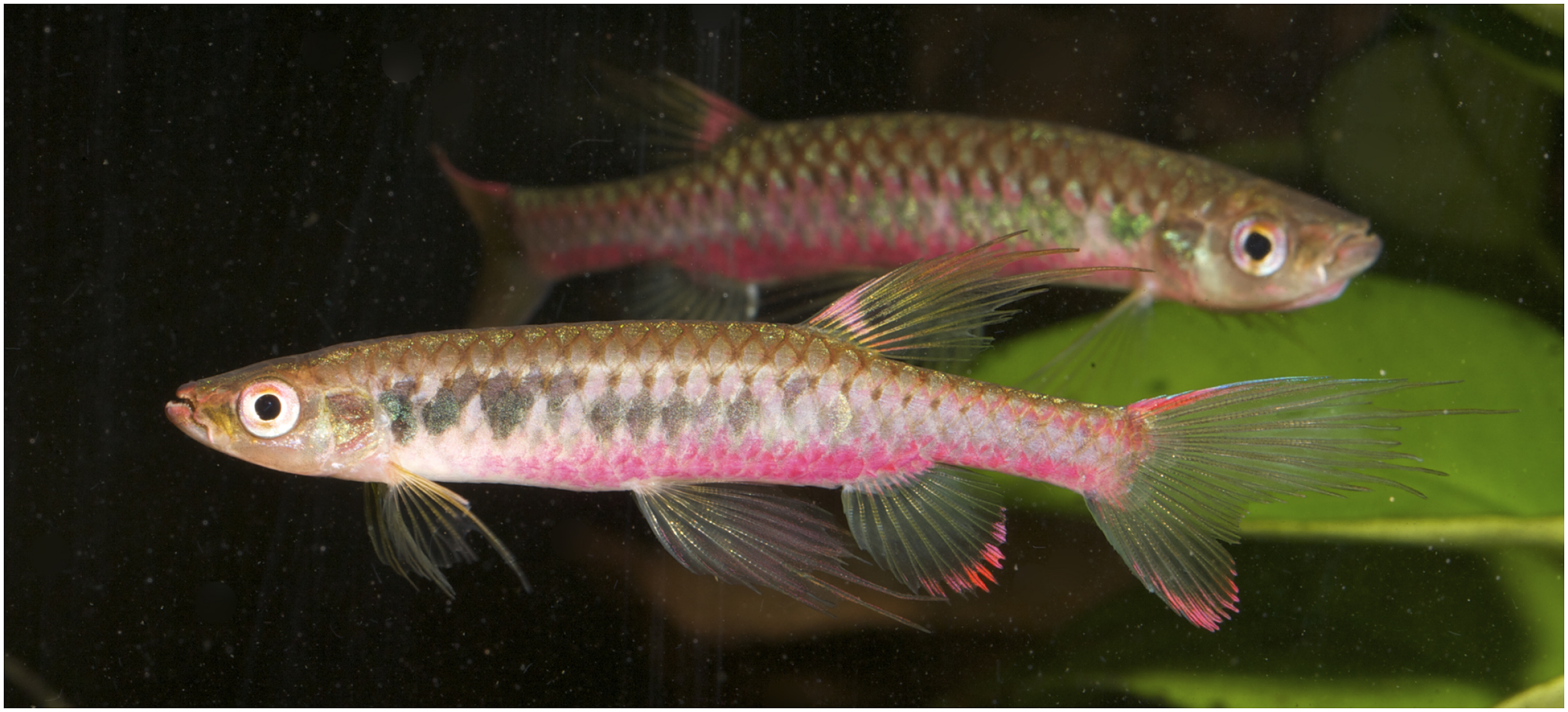
*Copella vilmae*, live males, aquarium specimens not preserved. Reprinted from belowwater.com under a CC BY license, with permission from Oliver Lucanus, original copyright 2017.

**Fig 30:**
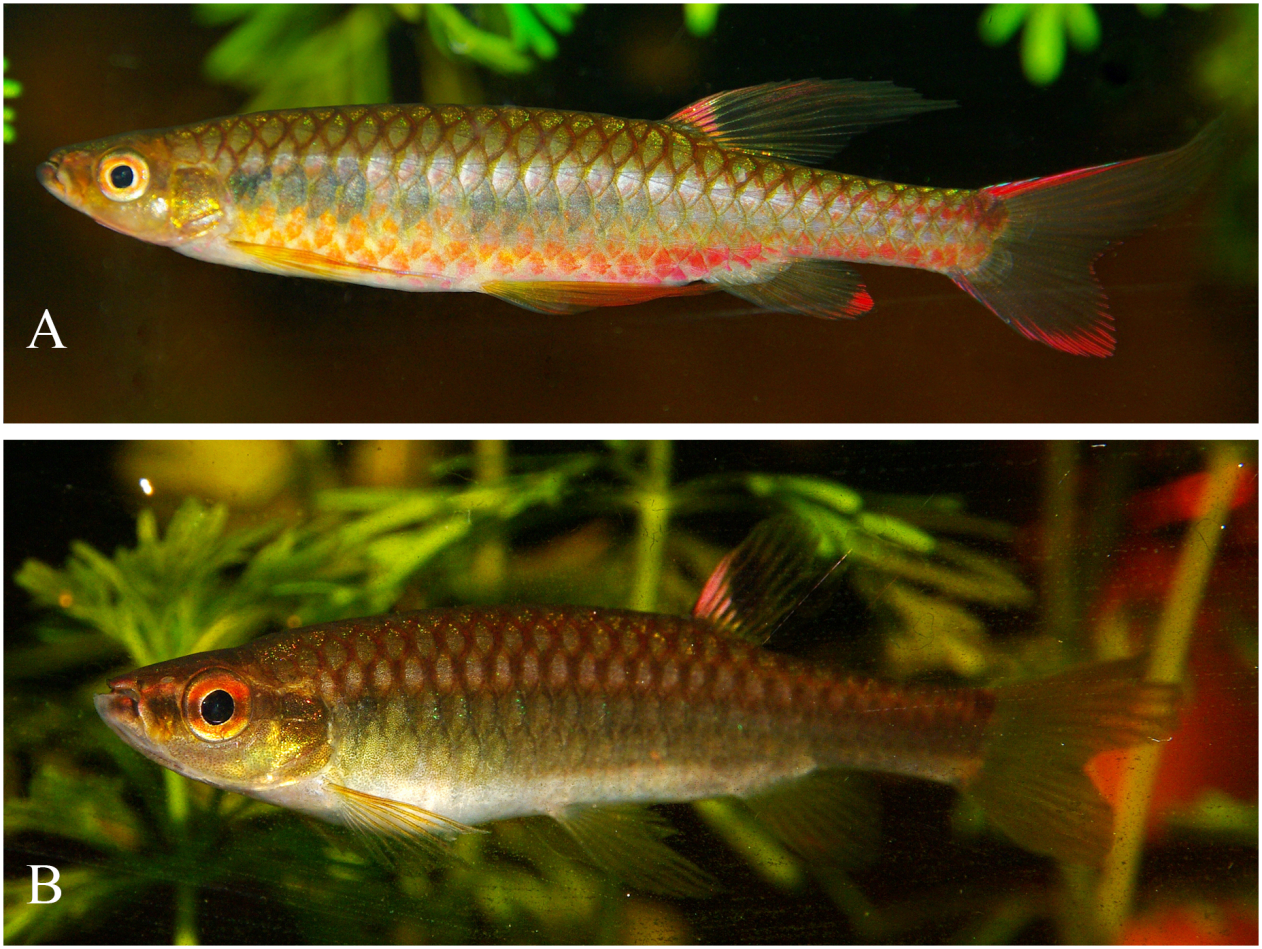
*Copella vilmae*, live aquarium specimens not preserved. (A) male, (B) female. Both pictures reprinted from apisto.sites.no under a CC BY license, with permission of Tom Christoffersen, original copyright 2017.

**Fig 31:**
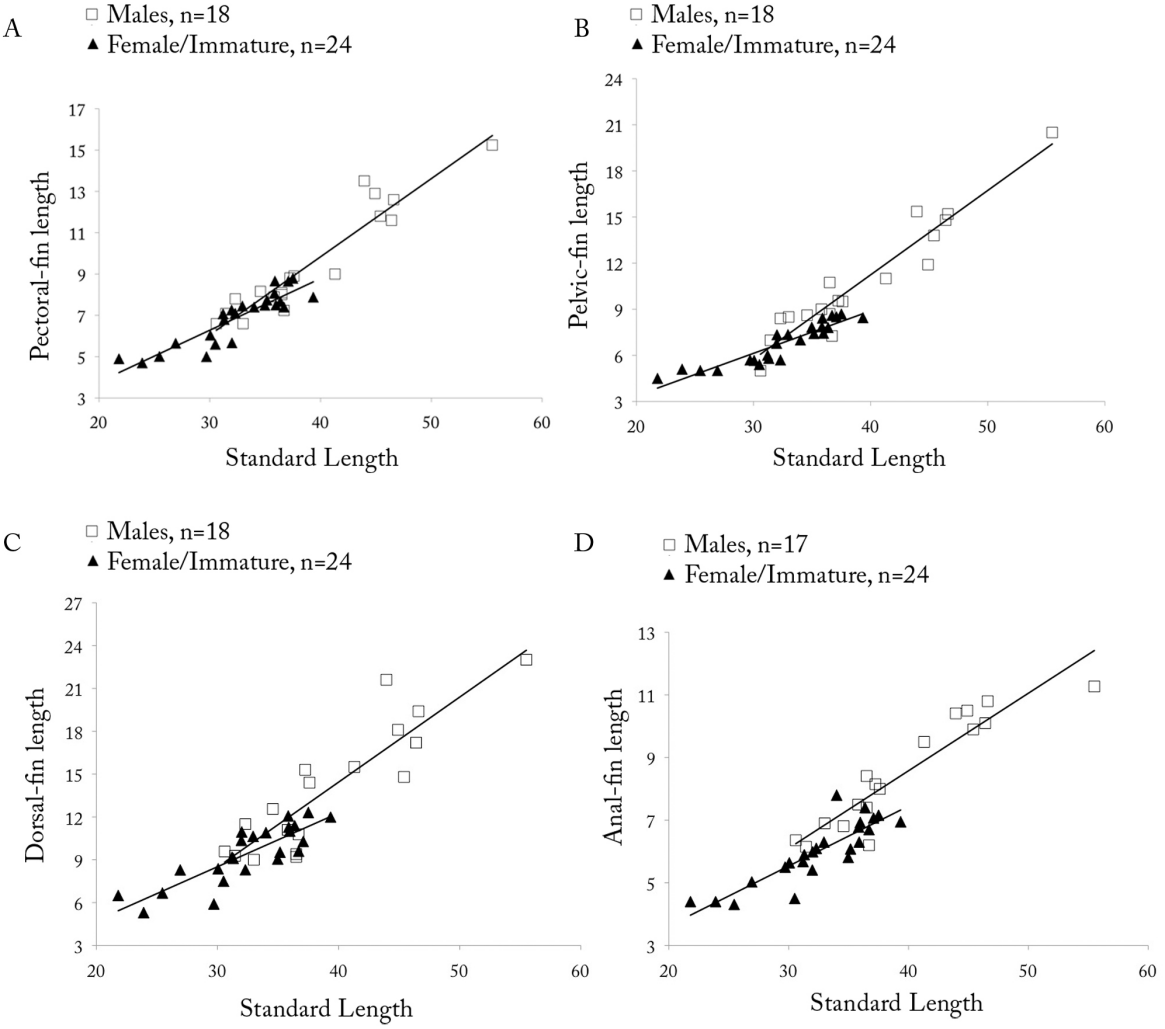
*Copella vilmae*. (A) pectoral-, (B) pelvic-, (C) dorsal-, and (D) anal-fin length as a function of SL by sex.

Figs 18, 28–31; Tables 11 and 12

**Table 11:** Morphometrics of *Copella vilmae*.

	*Copella vilmae*	n	Range		Mean	SD
	Holotype					
Standard length (mm)	43.9	41	21.8	55.5	35.1	
**Percents of standard length**						
Body depth	18.6	41	15.3	21.9	18.2	1.5
Dorsal- to caudal-fin origin	35.8	41	32.9	37.6	35.1	1.0
Snout to dorsal-fin origin	65.9	40	62.7	68.7	65.7	1.4
Snout to pectoral-fin origin	22.3	40	21.3	25.9	22.7	0.9
Snout to pelvic-fin origin	49.7	40	42.6	51.3	48.5	1.4
Snout to anal-fin origin	73.1	39	67.2	74.2	71.0	1.4
Pectoral- to pelvic-fin origin	27.9	41	22.7	29.2	26.7	1.5
Pelvic- to anal-fin origin	24.7	40	20.0	27.4	23.2	1.3
Pectoral-fin length males	30.7	17	19.7	28.7	23.6	2.6
Pectoral-fin length fem/imm	-	24	16.8	24.1	21.2	1.8
Pelvic-fin length males	35.0	17	16.3	36.9	26.4	4.9
Pelvic-fin length fem/imm	-	24	17.7	23.5	20.9	1.8
Dorsal-fin length males	49.2	17	25.2	41.6	34.2	5.6
Dorsal-fin length fem/imm	-	24	19.9	34.2	28.9	3.6
Anal-fin length males	23.7	16	16.9	23.4	21.2	1.7
Anal-fin length fem/imm	-	24	14.8	22.9	18.5	1.5
Anal-fin base length	10.3	41	7.1	10.9	8.8	0.9
Caudal peduncle depth	9.3	41	6.9	10.5	8.9	0.7
Caudal peduncle length	19.4	41	17.0	23.6	20.2	1.4
Head length	22.3	40	20.0	24.5	22.7	0.9
**Percents of head length**						
Eye diameter	33.9	39	29.2	40.3	34.8	2.8
Snout length	28.2	38	25.4	34.8	28.7	1.8
Interorbital distance	35.6	40	32.1	40.2	35.4	1.9
Upper jaw length	35.6	39	28.5	38.2	33.1	2.5

Holotype of *Copella vilmae* SMF 5931, paratypes of *C*. *vilmae* MHNG 2200.018 (2), SMF 5967–5978 (4), USNM 198135 (4) and non-type material AMNH 218052 (3), AMNH 218053 (5), IavH 8385 (2), IavH 11192 (6), IavH 11193 (13), n = number of specimens, SD = Standard deviation. Ranges do not include the holotype.

**Table 12:** Meristics of the holotype of *Copella vilmae*.

	Holotype
Dorsal-fin rays	ii8
Pectoral-fin rays	i9
Pelvic-fin rays	i7
Anal-fin rays	iii9
Caudal-fin rays	i8,7i
Predorsal scales	15
First longitudinal scale row	15
Fourth longitudinal scale row	25
Longitudinal scale rows dorsal to pelvic	5
Longitudinal scale rows dorsal to anal	5
Circumpeduncular scale rows	10
Total vertebrae	-

*Copella vilmae* Géry [6]: 25, figs 1–2 [type locality: igarapé Preto, affluent of upper rio Amazonas near Belém (= a small city in the upper rio Amazonas), about 60 km below Letícia, Amazonas, Peru-Colombia-Brazil border].—Géry [67]: 11 [notes on habitat].—Géry [5]: 147 [comparison with *Copella compta*; unnumbered figures pg. 149].—Vari & Howe [68]: 15 [listed].—Weitzman & Weitzman [1]: 242 [literature compilation].—Zarske & Géry [7]: 41 e 44 [identification key].—Oyakawa & Netto-Ferreira [15]: 64 [literature compilation].—Zarske [35]: 32 [taxonomic notes].

**Diagnosis.** *Copella vilmae* is easily distinguished from the remaining congeners by the presence of an interrupted longitudinal series of dark scales on the body of males (*vs*. presence of a continuous longitudinal series of dark scales forming a longitudinal band or absence of any longitudinal color pattern). Females of *C*. *vilmae* can be diagnosed from the remaining congeners, except females of *C*. *compta*, by the presence of a small dark spot at the base of the upper caudal-fin lobe (*vs*. absence). Females of *C*. *vilmae* can be distinguished from females of *C*. *compta* by the presence of a brownish longitudinal band on body (*vs*. absence).

**Description.** Morphometrics in Tables 11 and 12. Largest examined male 55.5 mm SL, female 36.4 mm SL. Greatest body depth at vertical through pelvic-fin origin. Body cylindrical, slightly compressed laterally. Dorsal profile of body straight to slightly convex from tip of snout to end of supraoccipital, slightly convex from that point to dorsal-fin origin, posteroventrally inclined along dorsal-fin base and straight along caudal peduncle. Ventral profile of body convex from anterior tip of dentary to vertical through anterior margin of orbit, straight from that point to vertical through pectoral-fin origin, straight to slightly convex from that point to pelvic-fin origin, straight from pelvic-fin origin to anal-fin origin, posterodorsally inclined along anal-fin base and straight along caudal peduncle.

Mouth upturned. Premaxillary teeth in one row, with 19 (1) or 20 (2) teeth, decreasing in size laterally. Number of maxillary teeth sexually dimorphic, 15 (1) in males and 5 (1) or 7 (2) in females, decreasing in size posteriorly, especially in males. Dentary teeth in two rows, outer with 12 (1) teeth increasing in size laterally, inner with 29 (1) teeth, decreasing in size laterally.

Dorsal fin with ii, 8* (42) rays, second and third branched rays longer. Pectoral fin with i (41), 8 (2), 9* (28), or 10 (11) rays, second and third branched rays longer. Pelvic fin with i, 7* (42) rays, third and fourth branched ray longer. Anal fin with iii (6), 8,i* (38) rays, third and fourth branched rays longer. Adipose fin absent. Caudal fin with i (40), 7 (2), 8* (38) rays in upper lobe, second and third branched rays longer, and 6 (1), 7* (38), or 8 (1), i (40) rays in lower lobe, second and third branched rays longer. Upper caudal-fin lobe longer than lower. Males with variable relative fin length. Males of same size may present distinct fin lengths (Fig 31).

Predorsal scales 15* (9), 16 (32), or 17 (1), in one series. First longitudinal scale row with 14 (4), 15* (24), 16 (12), or 17 (1) scales. Fourth longitudinal scale row with 24 (8), 25* (19), or 26 (14) scales. Lateral line not pored, first lateral line scale with small canal medially on its anterior portion, without lateral opening. Longitudinal scale rows between dorsal-fin origin and pelvic-fin origin 5* (16) or 6 (25). Longitudinal scale rows between dorsal-fin origin and anal-fin origin 5* (42). Circumpeduncular scale rows 10* (42). Total number of vertebrae 34 (2) or 35 (3).

**Color in alcohol.** Overall ground coloration of body beige. Dark stripe from anterior tip of dentary to posterior tip of opercle. Dorsal portion of body dark. Inconspicuous dark pigmentation at base of scales. Largest males with series of dark brown scales interposed with clear ones often on fourth longitudinal scale row, but also on third and fifth rows, without fixed arrangement. Brown scales on anterior portion of body deep dark, gradually lighter posteriorly (Fig 28A to 28C). Females and juveniles with distinct colorations of males, consisting of inconspicuous wide brownish stripe on flank, on fourth and fifth longitudinal scale rows, extending from opercle to caudal peduncle, with clear longitudinal stripe above it (Fig 28D). Juvenile males with color pattern intermediate between those of adult males and females, with longitudinal wide stripe on flank and clear stripe above it, and series of dark brown scales on fourth longitudinal scale row. Dorsal fin with black round spot. Remaining fins hyaline. Pelvic and anal fins usually with dark edge, more intense in males. Dorsal and ventral procurrent caudal-fin rays dark, darker in males. Females and juveniles with inconspicuous dark spot at base of upper caudal-fin lobe.

**Color in life.** Dorsal portion of body olivaceous, ventral portion pale yellow anteriorly and deep pink posteriorly. Males with series of brilliant green scales irregularly arranged, mainly on fourth longitudinal scale row. Base of dorsal fin, base of dorsalmost rays of upper caudal-fin lobe, and tip of basalmost rays of lower caudal-fin deep pink in males (Figs 29 and 30A). Females with metallic-green longitudinal band on body, extending from opercle to end of caudal peduncle (Fig 30B).

**Sexual dimorphism.** Males longer than females. Males with more numerous maxillary teeth than females (see description above). Pelvic, dorsal, and anal fins longer in males than in females. Pectoral-fin length apparently not sexually dimorphic. Tip of adpressed dorsal fin reaching to one-half length of upper caudal-fin lobe rays in males, and approximately to two-thirds length of caudal peduncle length in females. Tip of pectoral fin sometimes extending beyond pelvic-fin origin in males, never in females. Tip of adpressed pelvic fin reaching base of last anal-fin rays in males, and to anus in females. Upper caudal-fin lobe longer than lower one, especially in males. Differences in color pattern between sexes described in “Color in alcohol” section.

**Distribution.** *Copella vilmae* is known upper rio Amazonas, surroundings of Letícia, Colombia (Fig 18).

**Remarks.** Géry [6] described *Copella vilmae* based on the holotype (Fig 28A) and 20 paratypes. Four paratypes were kept in aquarium and then deposited at SU. These four specimens are now under USNM 198135. Among the remaining 16 paratypes, 12 were said to be deposited at SMF 5967–78 and nothing was said about the other four paratypes. At SMF, we only analyzed 11 paratypes stored in a single jar under SMF 5967–77, separated by small glass tubes. The paratype SMF 5978 was not available and it was not possible to confirm whether it is missing. The remaining four paratypes cited in the description are deposited at MHNG 2200.018.

**Material examined of *Copella vilmae* in** S1 Appendix.

## Supporting information

S1 Appendix Material examined of *Copella*. (DOCX)

## References

[1] Weitzman M, Weitzman SH. Family Lebiasinidae In: Reis RE, Kullander SO, Ferraris CJ Jr., editors. Check list of the freshwater fishes of South and Central America. Porto Alegre: Edipucrs; 2003 pp. 241–251.

[2] Eschmeyer WN, Fricke R. Catalog of Fishes electronic version. 8 2016 http://research.calacademy.org/research/ichthyology/catalog/fishcatmain.asp

[3] Weitzman SH. Osteology and relationships of South American characid fishes of subfamilies Lebiasinidae and Erythrinidae with special references to subtribe Nannostomina. Proc U S Nat Mus. 1964;116: 127–170.

[4] Myers GS. *Copella*, a new genus of pyrrhulinin characid fishes from the Amazon. Stanford Ichthyological Bulletin. 1956;7: 12–13.

[5] Géry J. Characoids of the World. Neptune City: T. F. H. Publications; 1977.

[6] Géry J. *Copella* vilmae n. sp. (Pisces: Characoidei). Senckenb Biol. 1963;44: 25–31.

[7] Zarske A, Géry J. Zur Identität von *Copella nattereri* (Steindachner, 1876) einschließlich der Beschreibung einer neuen Art (Teleostei: Characiformes: Lebiasinidae). Zool Abh. 2006;56: 15–46.

[8] Fink WL, Weitzman SH. The so-called Cheirodontin fishes of Central America with description of two new species (Pisces, Characidae). Smithson Contribu Zool. 1974;172: 1–46.

[9] Hubbs CL, Lagler KF. Fishes of the Great Lakes region. Bloomfield Hills: Cranbrook Institute of Science; 1947.

[10] Lundberg JG, Baskin JN. The caudal skeleton of the catfishes, Order Siluriformes. Am Mus Novit. 1979;2398: 1–49.

[11] Taylor WR, van Dyke GC. Revised procedures for staining and clearing smallfishes and other vertebrates for bone and cartilage study. Cybium. 1985;9: 107–119.

[12] Weitzman SH. Review of South American characid fishes of the subtribe Nannostomina. Proc U S Nat Mus. 1966;119: 1–56.

[13] Weitzman SH, Cobb JS. A revision of the South American fishes of the genus *Nannostomus* Günther (Family Lebiasinidae). Smithson Contrib Zool. 1975; 186: 1–36.

[14] Vari RP. The neotropical fish family Ctenoluciidae (Teleostei: Ostariophysi: Characiformes): supra and intrafamilial phylogenetic relationships, with a revisionary study. Smithson Contrib Zool. 1995;564: 1–97.

[15] Oyakawa OT, Netto-Ferreira AL. Família Lebiasinidae In: Buckup PA, Menezes NA, Ghazzi MA, editors. Catálogo das espécies de peixes de água doce do Brasil. Rio de Janeiro: Museu Nacional; 2007 pp. 64–65.

[16] Netto-Ferreira A. L. Three new species of Lebiasina (Characiformes: Lebiasinidae) from the Brazilian Shield border at Serra do Cachimbo, Pará, Brazil. Neotrop Ichthyol. 2012;10: 487–498.

[17] Eigenmann CH, Eigenmann RS. A review of the Erythrininae. Proc Calif Acad Sci. 1889;2: 100–116,pl. 1.

[18] Eigenmann CH. The freshwater fishes of British Guiana, including a study of the ecological grouping of species, and the relation of the fauna of the plateau to that of the lowlands. Mem Carnegie Mus. 1912;5: i–xxii, 1–578, pls 1–103.

[19] Magalhães AC. Monographia Brazileira de Peixes Fluviaes. São Paulo: Graphicars 1931.

[20] Regan CT. A revision of the South American characid fishes of the genera *Chalceus*, *Pyrrhulina*, *Copeina and Pogonocharax*. Ann Mag Nat Hist. 1912;10: 387–395.

[21] Myers GS. Descriptions of new South American fresh-water fishes collected by Dr. Carl Ternetz. Bull Mus Comp Zool. 1927;68: 107–135.

[22] Fowler HW. Os peixes de água doce do Brasil. Arquivos de Zoologia do Estado de São Paulo. 1948;6: 1–204.

[23] Boeseman M. A preliminary list of Surinam fishes not included in Eigenmann’s enumeration of 1912. Zool Meded. 1952;31: 179–200.

[24] Meinken H. Mitteilungen der Fischbestimmungsstelle im WB. des VDA. X. *Pyrrhulina nigrofasciata* spec. nov. Aquarien-Terrarien-Z. 1952;5: 115–117.

[25] Boeseman M. Scientific results of the Surinam expedition 1948–1949. Part II. Zoology. N° 2. The Fishes (I). Zool Meded. 1953;32: 1–24.

[26] Boeseman M. On a small collection of Surinam fishes Zool Meded. 1954;34: 183–199.

[27] Boeseman M. On Recent Accessions of Surinam Fishes. Zool Meded. 1956;34: 183–199.

[28] Krekorian C O'Neil, Dunham DW. Preliminary observations on the reproductive and parental behaviour of the spraying characid *Copeina arnoldi* Regan. Z Tierpsychologie. 1972;31: 419–437. 4650799

[29] Krekorian C O’Neil, Dunham DW. Parental egg care in the spraying characid, *Copeina arnoldi* Regan: Role of the spawning surface. Anim Behav. 1972;20: 356–360.

[30] Krekorian C O’Neil, Dunham DW. Visual discrimination by the spraying characid *Copeina* arnoldi* Regan. Anim Behav. 1973;21: 741–748. 4777203 10.1016/s0003-3472(73)80100-9

[31] Krekorian C O’Neil. Field observations in Guyana on the reproductive biology of the spraying characid *Copeina arnoldi* Regan. Am Midl Nat. 1976;96: 88–97.

[32] Planquette P, Keith P, Le-Bail PY. Atlas des Poissons Déau Douce de Guyane (tome I). Paris: Muséum National d'Histoire Naturelle; 1996.

[33] Keith P, Nandrin L, Le Bail P. *Rivulus gaucheri*, a new species of rivuline (Cypronodontiformes: Rivulidae) from French Guiana. Cybium. 2006;30: 133–137.

[34] Montag LFA, Albuquerque AA, Freitas TMS, Barthem RB. Ictiofauna de campos alagados da Ilha do Marajó, Estado do Pará, Brasil. Biota Neotropica. 2009;9: 241–253.

[35] Zarske A. Beiträge zur Kenntnis der Vertreter der Gattungen *Pyrrhulina* Valenciennes, 1846 und *Copella* Myers, 1956 des nordöstlichen Südamerika (Teleostei: Characiformes: Lebiasinidae). Vertebr Zool. 2011;61: 13–45.

[36] Mol JH, Vari RP, Covain R, Willink PW, Fisch-Muller S. Annotated checklist of the freshwater fishes of Suriname. Cybium. 2012;36: 263–292.

[37] Géry J. Notes on characoid fishes collected in Surinam by Mr. H. P. Pijpers, with descriptions of new forms. Contrib. Zool., Amsterdam. 1965;35: 101–126.

[38] Mérigoux S, Ponton D, Mérona B. 1998. Fish richness and species-habitat relationships in two coastal streams of French Guiana, South America. Environ Biol Fishes. 1998;51: 25–39.

[39] Montag LFA., Freitas TMS, Wosiacki WB, Barthem RB. Os peixes da Floresta Nacional de Caxiuanã (municípios de Melgaço e Portel, Pará–Brasil). Bol Mus Para Emílio Goeldi, sér Ciências Naturais, 2008; 3: 11–34.

[40] Lasso CA, Sánchez-Duarte P. Los peces del delta del Orinoco. Diversidad, bioecología, uso y conservación. Caracas: Fundación La Salle de Ciencias Naturales y Chevron C. A; 2011.

[41] Kenny JS. Views from the Bridge: a memoir on the freshwater fishes of Trinidad. Narataria: Trinprint Ltd; 1995.

[42] Phillip DAT, Taphorn DC, Holm E, Gilliam BAL, López-Fernández H. Annoted list and key to the stream fishes of Trinidad & Tobago. Zootaxa. 2013;3711: 001–064. 10.11646/zootaxa.3711.1.1 25320768

[43] Fowler HW. Fishes from the Madeira River, Brazil. Proc Acad Nat Sci Philadelphia. 1913;65: 517–579.

[44] Wilkens H. Die Typen der Ichthyologischen Sammlung des Zoologischen Instituts und Zoologischen Museums der Universität Hamburg (ZMH), Teil III. Mitt Hamb Zool Mus Inst. 1977;74: 155–163.

[45] Ortega H, Vari RP. Annotated checklist of the freshwater fishes of Peru. Smithson Contribu Zool. 1986;437: iii + 25 p.

[46] Zarske A, Géry J. Ein neuer Salmler aus Peru, *Pyrrhulina zigzag* sp. n. (Pisces: Teleostei: Lebiasinidae). Das Aquarium.1997;31: 12–17.

[47] Bogotá-Gregory JD, Maldonado-Ocampo JA. Peces de la zona hidrogeográfica de la Amazonia, Colombia. Biota Colombiana. 2006;7: 55–94.

[48] Maldonado-Ocampo JA, Vari RP, Usma JS. Checklist of the Freshwater Fishes of Colombia. Biota Colombiana. 2008;9: 143–237.

[49] Böhlke JE. A catalogue of the type specimens of Recent fishes in the Natural History Museum of Stanford University. Stanford Ichthyological Bulletin. 1953;5: 1–168.

[50] Wallace AR. Peixes do Rio Negro [Fishes of the Rio Negro] Organization, introductory text and translation by Mônica de Toledo-Piza Ragazzo. São Paulo: Editora da Universidade de São Paulo; 2002.

[51] Eigenmann CH. The Fishes of Western South America, Part I. The Fresh-water Fishes of Northwestern South America, Including Colombia, Panama, and the Pacific slopes of Ecuador and Peru, Together with an Appendix Upon the Fishes of the Rio Meta in Colombia. Mem Carnegie Mus.1922;9: 1–349. Pls 1–38.

[52] Eigenmann CH. On new species of fishes from the Rio Meta basin of weastern Colombia and on albino or blind fishes from near Bogotá. Indiana University studies. 1914;23: 229–230.

[53] Fowler HW. Descriptions of two new fresh-water fishes from Colombia. *Not Nat* Acad Nat Sci Philadelphia. 1945;158: 1–111.

[54] Taphorn DC. The characiform fishes of the Apure River drainage, Venezuela. Biollania Edición Especial, 4. Guanara: Monografias Cientificas del Museo de Ciencias Naturales, UNELLEZ; 1992.

[55] Galvis G, Mojica JI, Provenzano F, Lasso C, Taphorn D, Royero R, et al Peces de la Orinoquía colombiana con énfasis en especies de interés ornamental. Bogotá: Ministério de Agricultura y Desarrollo Rural, INCODER, Universidad Nacional de Colombia; 2007.

[56] Maldonado-Ocampo JA, Lugo M, Bogotá-Gregory JD, Lasso CA, Vássquez L, Usma JS, et al Pexes del río Tomo, cuenca del Orinoco, Colombia. Biota Colombiana. 2006;7: 113–128.

[57] Urbano-Bonilla A, Zamudio J, Maldonado-Ocampo JA, Bogotá-Grégory JD, Cortes-Millán GA, López Y. Peces del piedemonte del departamento de Casanare, Colombia. Biota Colombiana. 2009;10: 149–162.

[58] Galvis G, Sánchez P, Mesa L, López Y, Gutiérrez MA, Gutiérrez A, et al Peces de la Amazonia colombiana con énfasis en especies de interés ornamental. Bogotá: Ministério de Agricultura y Desarrollo Rural, INCODER, Universidad Nacional de Colombia—Departamento de Biología-Instituto de Ciencias Naturales 2007.

[59] Steindachner F. Beiträge zuer Kenntniss der Characinen des Amazonenstromes. Sitzungsber. Kaiserl. Akad. Wiss., Wien. 1876;72: 6–24, pls. 1–2.

[60] Fowler HW. A collection of Fishes Obteined by Mr. William C. Morrow in the Ucayali River Basin, Peru. Proc Acad Nat Sci Philadelphia. 1940;91: 219–1940.

[61] Eigenmann CH. Catalogue of the fresh-water fishes of tropical and south temperate America. In: Reports of the Princeton University expeditions to Patagonia 1896–1899. Zoology. 1910;3: 375–511.

[62] Arbeláez F, Gálvis G, Mojica JI, Duque S. Composition and richnes of the ichthyofauna in a *terra firme* forest stream of the Colombian Amazonia. Amazoniana. 2004;18: 95–107.

[63] Bogotá-Gregory JD, Maldonado-Ocampo JA. La colección de peces del Instituto Alexander von Humboldt (IAvH) nuevos registros y representatividad parte II: Amazonia. Dahlia. 2005;8: 61–69.

[64] Mojica IJ, Gálvis G, Arbeláez F, Santos M, Vejarano S, Prieto-Piraquive E, et al Peces de la Cuenca del río Amazonas en Colombia: Región de Leticia. Biota Colombiana. 2005;6: 191–210.

[65] Borodin NA. Notes on some species and subspecies of the genus *Leporinus* Spix. Mem Mus Comp Zool. 1929;50: 268–191.

[66] Ulrey AB. The South American Characinidae collected by Chas. Fred. Hartt. Ann N Y Acad Sci. 1895;8: 257–300.

[67] Géry J. Characidae et Crenuchidae de I’Igarapé Préto (Haute Amazonie). Senckenb biol. 1965;46: 11–45.

[68] Vari RP, Howe JC. Catalog of Type Specimens of Recent Fishes in the National Museum of Natural History, Smithsonian Institution, 1: Characiformes (Teleostei: Ostariophysi). Smithson Contrib Zool. 1991;517: 1–52.

