# Supplementary material for: Taxonomic review of Copella (Characiformes: Lebiasinidae) with an identification key for the species
Source: PLoS One. 2017 Aug 17;12(8):e0183069. doi: 10.1371/journal.pone.0183069 (PMC5560855; doi:10.1371/journal.pone.0183069)
Supplement: S1 Appendix — (DOCX) [file pone.0183069.s001.docx]

**S1 Appendix. Material examined of *Copella.***

**Material examined of *Copella arnoldi***

*Type material.* BMNH 1909.4.2.25-26 (2 syntypes of *Copeina arnoldi*, 25.4 and 34.4 mm SL), Amazon (=lower rio Amazonas basin), Arnoldi. BMNH 1899.7.26.1-5 [ex MNHN] (5 syntypes of *Copeina carsevennensis*, 17.0-24.3 mm SL), Carsevenne (=rio Calçoene, Amapá, Brazil, approximately 2º30’N 50º57’W). BMNH 1911.10.31.140 (7 paralectotypes of *Copeina eigenmanni*, 14.8-20.9 mm SL), rio Aruka, Guyana [=approximately 8º12’N 59º44’W], 1908, C. Eigenmann. BMNH 1894.5.18.40-41 (2 paralectotypes of *Copeina eigenmanni*, Pará [=Brazil]. BMNH 1911.10.31.146 (1 paralectotype of *Copeina eigenmanni*, 21.6 mm SL), rio Lama, Guyana [=approximately 6º32’N 58º1’W], 1908, C. Eigenmann.

*Non-type material. Rio Amazonas basin, Brazil.* DZSJRP 11106 (7, 15.5-27.6 mm SL), stream without name, at main road of Jutaituba farm, rio Pacajá, rio Pará, Portel, Pará, 2°59’34”S 50°11’18”W, 23 Apr 2008, J. Serra *et al.* DZSJRP 11120 (4, 16.1- 33.1 mm SL), igarapé Santo Antônio at Jutaituba farm, rio Jacundá, Baião, Pará, 3°2’10”S 50°0’W, 24 Apr 2008, J. Serra *et al.* DZSJRP 11231 (22, 21.2-35.5 mm SL), stream without name, rio Pacajá, rio Pará, Baião, Pará, 3°4’57”S 49°59’59”W, 21 Apr 2008, J. Serra *et al.* DZSJRP 11237 (2, 24.9 and 25.0 mm SL), stream without name at Jutaituba farm, rio Pacajá , rio Pará, Baião, Pará, 2°53’32”S 50°9’37”W, 26 Apr 2008, J. Serra *et al.* DZSJRP 11239 (2, 16.5 and 22.7 mm SL), igarapé Moconho at Jutaituba farm, rio Pacajá, rio Pará, Baião, Pará, 2°55’57”S 50°13’3”W, 26 Apr 2008, J. Serra *et al.* INPA 39737, igarapé da Carol, Lago Ajuruxi, RESEX Cajari, Mazagão Ajuruxi, Amapá, 0º34’27.5”S 51º54’57.7”W, N. Junior. MCZ 46128 (1, 28.7 mm SL), stream at km 18, Castanhal-Belém road, Amazonas, Santa Isabel, Pará, Jul 1965, N. Menezes. MCP 41586 (15, not measured), affluent of rio Barcarena, Barcarena, Pará, 21 Oct 2006, 1º36’10”S 48º40’32”W, F. Vieira, L. Figueiredo & J. Corrêa. MCP 48312 (4, not measured), Córrego do Bispo, Mazagão, Amapá, 3º52’32”N 55º24’26”W, 15 Jul 2008, J. Pezzi & L. Cotrim. MNHN 1898.0053 (6, 22.3-31.0 mm SL), Carsevenne (= rio Calçoene, Amapá), Geay. MPEG 2940 (41, 15.1-22.4 mm SL), mouth of rio Goiapi, Cachoeira do Arari, Ilha do Marajó, Cachoeira do Arari, Pará, 8 Sep 1990, M. Assunção. MPEG 2941 (22, 14.7-27.4 mm SL), mouth of rio Goiapi, Cachoeira do Arari, Ilha do Marajó, Cachoeira do Arari, Pará, 08 Sep 1990, M. Assunção. MPEG 4778 (1, 21.9 mm SL), mouth of rio Goiapi, Cachoeira do Arari, Ilha do Marajó, Cachoeira do Arari, Pará, 1 Nov 1991, M. Assunção. MPEG 4988 (4, 17.2-20.4 mm SL), rio Guamá, Mocambo reservoir, Belém, Pará, 16 Sep 1997, N. Bittencourt. MPEG 5710 (8, 14.9-20.9 mm SL), rio Guamá, Mocambo reservoir, Belém, Pará, 19 Mar 1999, F. Pimentel. MPEG 6559 (36, 14.0-31.7 mm SL), igarapé Curuá, Caxiuanã (ECFPn), Melgaço, Pará, 1°42.6’S 51°26.9’W, 6 Oct 2000, L. Montag. MPEG 6563 (3, 17.7-23.8 mm SL), igarapé Curuá, Caxiuanã (ECFPn), Amazonas, Melgaço, Pará, 1°42.6’S 51°26.9’W, 6 Oct 2000, L. Montag. MPEG 6946 (4, 23.5-29.0 mm SL), tributary of rio Cachoeirinha, headquarter, Paragominas, Pará, 3°12’7”S 47°45’6”W, 14 Apr 2003, A. Bezerra & V. Sena. MPEG 6950 (1, 33.9 mm SL), tributary of rio Cachoeirinha, headquarter, Capim, Paragominas, Pará, 3°12’7”S 47°45’6”W, 17 Apr 2003, V. Sena. MPEG 7138 (1, 23.1 mm SL), rio Arienga, São Sebastião, Barcarena, Pará, 1°38’S 48°43’W, 26 Mar 2003, A. Sousa & V. Sena. MPEG 7142 (3, 11.0-16.4 mm SL), stream under transmission line of PA 481, Barcarena, Pará, 1°38’S 48°45’W, 27 Mar 2002, A. Sousa & V. Sena. MPEG 7162 (1, 22.8 mm SL), Vila Arienga, Barcarena, Pará, 26 Mar 2002. MPEG 7172 (1, 22.7 mm SL), igarapé Tauá, near PA 483, Barcarena, Pará, 1°34’S 48°42’W, 25 Mar 2002, V. Sena. MPEG 7181 (9, 14.5-22.0 mm SL), steam at PA 483, near Castanhalzinho, Barcarena, Pará, 1°38’S 48°39’W, 27 Mar 2002, A. Bezerra & V. Sena. MPEG 7182 (1, 38.9 mm SL), igapó Torre, Estação Científica Ferreira Penna, Pará, Melgaço, Pará, 1 Jun 2002, L. Montag. MPEG 7182 (6, 13.3-35.7 mm SL), igarapé Caripiaçu, at road of beach Caripi at Vila dos Cabanos, 1.5 km of beach, Barcarena, Pará, 1°29’S 48°42’W, 22 Mar 2002, V. Sena. MPEG 7183 (1, 23.8 mm SL), igarapé Tauá, tributary of rio Barcarena, under transmission line at PA 481, Barcarena, Pará, 1°39’05”S 48°39’13”W, 23 Mar 2002, A. Bezerra & V. Sena. MPEG 7334 (8, 16.6-27.7 mm SL), igarapé Paraquequara, headquarter of Mineração farm, Paragominas, Pará, 3°16’40”S 47°43’53”W, 14 Dec 2002, A. Bezerra & V. Sena. MPEG 7403 (1, 20.6 mm SL), igarapé Paraquequara, near Monte Santo farm, Paragominas, Pará, 3°16’18”S 47°46’5”W, 13 Dec 2002, A. Sousa & V. Sena. MPEG 7446 (1, 17.4 mm SL), stream tributary of rio Paraquequara, headquarter of Monte Santo farm, near Trincheira II, Paragominas, Pará, 3°15’18”S 47°45’13”W, 14 Dec 2002, A. Sousa & V. Sena. MPEG 7484 (7, 15.2-26.8 mm SL), igarapé Paraquequara, headquarters of Monte Santo farm, Paragominas, Pará, 3°13’30”S 47°45’09”W, 15 Dec 2002, A. Sousa & V. Sena. MPEG 7490 (1, 23.1 mm SL), Pesqueiro São Raimundo, quadra 32, Almeirim, Pará, 26 Jul 1999, J. Junior. MPEG 7495 (1, 30.4 mm SL), igarapé Paraquequara, headquarters of Mineração farm, Paragominas, Pará, 3°16’40”S 47°43’53”W, 14 Dec 2002, V. Sena. MPEG 8217 (4, 14.9-25.3 mm SL), stream headwater of rio Galego, Mário Célio farm, rio Caete, Bragança, Pará, 29 Apr 2005, R. Silva. MPEG 8223 (2, 32.7 and 34.5 mm SL), stream headwater of rio Galego, Mário Célio farm, rio Caete, Bragança, Pará, 14 Apr 2005, R. Silva. MPEG 8305 (197, 15.0-32.5 mm SL, 2 c&s, 29.7-30.5 mm SL), rio Caripiaçu at road Vila dos Cabanos, near Caripi beach, Barcarena, Pará, 20 Sep 2001, W. Wosiacki & W. Bezerra. MPEG 8366 (7, 15.3-28.3 mm SL), headwater rio Galego, Bairro do Samaumapara, Bragança, Pará, 20 May 2005, R. Silva. MPEG 9413 (9, 11.8-23.5 mm SL), igarapé Anuerazinho, Tomé-Açu, Pará, 2°32’39.2”S 48°16’10.5”W, 1 Jul 2005, A. Sousa. MPEG 9416 (9, 20.4-32.0 mm SL), rio Paranã, Castanhal farm, Ponta de Pedras, Pará, 1°22’9.3”S 48°55’24.7”W, 18 Dec 2003, A. Sousa. MPEG 9418 (3, 12.8-18.4 mm SL), igarapé Arrainha, Tomé-Açu, Pará, 2°25’11.1”S 48°12’13.1”W, 1 Jul 2005, A. Sousa. MPEG 9419 (1, 23.4 mm SL), igarapé Marupaúba, Tomé-Açu, Pará, 2°13’14”S 48°8’14.3”W, 1 Jul 2005, A. Sousa. MPEG 9421 (17, 17.9-32.1 mm SL), rio Puraquequara, Caxiuanã (ECFPn), Melgaço, Pará, 1 Nov 1999, R. Barthem. MPEG 9422 (125, 11.6-28.5 mm SL), rio Caxiuanã, FLONA de Caxiuanã, Melgaço, Pará, 1 Dec 2004, L. Montag. MPEG 9425 (2, 25.8 and 26.5 mm SL), stream headwater of rio Galego, Bragança, Pará, 15 Apr 2005, R. Silva. MPEG 9428 (86, 13.5-24.9 mm SL), rio Caxiuanã, FLONA de Caxiuanã, Amazonas, Melgaço, Pará, 1°45.7’29”S 51°23.9’19”W, 29 Nov 2004, L. Montag. MPEG 9554 (2, 15.4 and 21.4 mm SL), igarapé Anuera-Grande, Tomé-Açu, Pará, 2°30’2.9”S 48°16’52.6” W, 30 Jun 2005, A. Sousa. MPEG 10231 (2, 17.9 and 22.5 mm SL), stream of Balneário Taiassuí, Benevides, Pará, 5 Jun 2006, L. Montag. MPEG 10385 (1, 14.5 mm SL), igarapé Curuazinho, Melgaço, Pará, 1°43.9’18.1”S 51°26’W, 14 Nov 2003, L. Montag. MPEG 10387 (18, 13.7-30.7 mm SL), igarapé Curuá, rio Pará, Melgaço, Pará, 1°42’51”S 51°27’36”W, 13 Nov 2003, L. Montag. MPEG 10398 (28, 14.2-33.4 mm SL, 2 c&s, 27.4-31.5 mm SL), igarapé Curuazinho, rio Pará, Melgaço, Pará, 1°44’12.9”S 51°25’54”W, 17 Nov 2003, L. Montag. MPEG 10402 (1, 17.1 mm SL), igarapé Moju, Melgaço, Pará, 23 Nov 2003, L. Montag. MPEG 10411 (3, 16.6-21.7 mm SL), stream, Melgaço, Pará, 22 Nov 2003, L. Montag. MPEG 10419 (1, 17.4 mm SL), stream, Melgaço, Pará, 1 Dec 2004, L. Montag. MPEG 10715 (423, 15.6-29.9 mm SL), igarapé FLONA de Caxiuanã, rio Pará, Melgaço, Pará, 19 Nov 2004, L. Montag. MPEG 10716 (238, 12.9-29.8 mm SL), igarapé FLONA de Caxiuanã, rio Pará, Melgaço, Pará, 1°45.6’S 51°25.46’W, 30 Nov 2004, L. Montag. MPEG 11261 (22, 13.9-29.5 mm SL), rio Puraquequara, FLONA de Caxiuanã, Melgaço, Pará, 1°43,9’38”S 51°28,2’9”W, 14 Nov 2003, L. Montag. MPEG 11271 (1, 38.7 mm SL), igapó Torre, Estação Científica Ferreira Penna, rio Pará, Melgaço, Pará, 1 Jun 2002, L. Montag. MPEG 11469 (37, 14.0-30.1 mm SL), igarapé Grande, rio Pará, Melgaço, Pará, 8 Jun 2008, L. Montag. MPEG 11491 (12, 13.2-22.4 mm SL), igarapé Curuá, Melgaço, Pará, 1 Jun 2002, L. Montag. MPEG 12124 (27, 10.1-26.5 mm SL), stream of balneário Taiassuí, Benevides, Pará, 19 Apr 2003, W. Wosiacki. MPEG 12478 (9, 14.4-21.6 mm SL), stream of balneário Taiassuí, Benevides, Pará, 1°23’43.7”S 48°14’57.3”W, 13 Nov 2006, A. Hercos. MPEG 12577 (12, 10.1-22.9 mm SL), igarapé do Gelo, Benevides, Pará, 1°21’41”S 48°14’41”W, 6 May 2006, M. Medonça. MPEG 15557 (1, 25.1 mm SL), rio Ipitinga, Almeirim, Pará, 0°51’13.8”S 53°57’5.8”W, 23 Oct 2008, T. Freitas. MPEG 15586 (11, 14.6-28.0 mm SL), rio Ipitinga, Almeirim, Pará, 0°49’59.2”S 53°56’7.7”W, 23 Oct 2008, T. Freitas. MPEG 15619 (10, 17.8-22.1 mm SL), rio Ipitinga, Almeirim, Pará, 0°49’2”S 53°56’27”W, 25 Oct 2008, T. Freitas. MPEG 15640 (7, 16.0-26.2 mm SL), rio Ipitinga, Almeirim, Pará, 0°49’51.9”S 53°57’19.6”W, 26 Oct 2008, T. Freitas. MPEG 15651 (1, 18.3 mm SL), rio Ipitinga, Almeirim, Pará, 0°49’43.8”S 53°56’53.4”W, 26 Oct 2008, T. Freitas. MPEG 15652 (4, 17.8-24.7 mm SL), rio Ipitinga, Almeirim, Pará, 0°49’43.8”S 53°56’53.4”W, 26 Oct 2008, T. Freitas. MPEG 15673 (7, 17.4-25.1 mm SL), rio Ipitinga, Almeirim, Pará, 0°48’33.7”S 53°55’51.7”W, 27 Oct 2008, T. Freitas. MPEG 15778 (1, 21.7 mm SL), rio Ipitinga, Almeirim, Pará, 0°49’24.2”S 53°55’43.4”W, 30 Oct 2008, T. Freitas. MPEG 17822 (10, 14.1-20.2 mm SL), igarapé Taiassuí, Benevides, Pará, 1°23’46”S 48°14’59.4”W, 1 Sep 2009, W. Wosiacki. MPEG 18254 (2, 21.4 and 23.3 mm SL), igarapé Ponte Quebrada, rio Guamá, Ourém, Pará, 1°32’17.5”S 47°05’2.9”W, 15 Nov 2009, B. Pamplona. MZUSP 23064 (129, 15.3-38.8 mm SL), alegre, 15 km NE of Marapanim, black water stream, Marapanim, Pará, 0°43’S 47°40’W, 12 Sep 1965, Expedição do Departamento de Zoologia. MZUSP 101948 (7, 19.7-24.6 mm SL), rio Caracuru and stream without name, affluent of rio Caruru, near Vila Santa Maria, rio Jari, Monte Dourado, Pará, 0°54’55”S 52°34’39”W, 10 Oct 2007, M. Carvalho *et al.* MZUSP 105746 (18, 10.0-22.2 mm SL), igarapé Mocajuteua, affluent of rio Moju, rio Tocantins, Igarapé Miri, Pará, 1°57’51”S 48°54’18”W, 9 Apr 2010, M. Marinho & D. Bastos. MZUSP 105756 (22, 15.5-31.9 mm SL), igarapé Lagoa Azul, affluent of rio Tauá, Santo Antônio do Tauá, Pará, 1°9’6”S 48°6’35”W, 2 Apr 2010, M. Marinho & D. Bastos. MZUSP 105763 (2, 21.9 and 24.4 mm SL), clear water igarapé at Bacuriteua, affluent of rio Marapanim, Pará, 0°37’36”S 47°40’45”W, 3 Apr 2010, M. Marinho & D. Bastos. MZUSP 105770 (130, 16.6-40.1 mm SL, 6 c&s, 12.8-31.4 mm SL, 2 mus, 21.7-33.7 mm SL), stream and lagoon, headquarters of Lagoa Azul farm, Vigia, Pará, 0°56’8”S 48°4’38”W, 27 Mar 2010, M. Marinho *et al.* MZUSP 105776 (14, 19.9-34.9 mm SL), rio igarapé-Açú, affluent of rio Guamá, Bujaru, Pará, 1°37’12”S 48°3’10”W, 4 Apr 2010, M. Marinho & D. Bastos. MZUSP 105780 (4, 27.5-29.8 mm SL), stream in reservoir at road near Caripi, rio Tocantins, Barcarena, Pará, 1°29’58”S 48°42’08”W, 9 Apr 2010, M. Marinho & D. Bastos. MZUSP 105799 (7, 12.7-29.8 mm SL), stream affluent of rio Marapanim, Marapanim, Pará, 0°42’46”S 47°43’13”W, 4 Apr 2010, M. Marinho & D. Bastos. MZUSP 105810 (1, 25.4 mm SL), igarapé do Tubo, affluent of rio Mojuim, Vigia, Pará, 1°4’49”S 48°1’55”W, 2 Apr 2010, M. Marinho & D. Bastos. MZUSP 106100 (6, 11.4-37.6 mm SL), stream of balneário Olho no Olho, Ilha do Marajó, Salvaterra, Pará, 0°50’51”S 48°34’1”W, 10 Apr 2010, M. Marinho & D. Bastos. MZUSP 106153 (8, 23.2-31.3 mm SL), stream at PA 318, affluent of rio Marapanim, Marapanim, Pará, 0°42’30”S 47°43’42”W, 3 Apr 2010, M. Marinho & D. Bastos. USNM 88272 (4, 20,2-34,6 mm SL), Maranhão, 1926, G. Ormaechea. USNM 203574 (3, 20,6-26,0 mm SL), small stream in Santa Isabel do Pará, near Belém, Pará, 18 Jul 1935, H. Cerron. ZMB non-catalogued (5, 31.9-39.4 mm SL), Ilha do Arapiranga, Pará, Broderson, 1928.

*Coastal drainages, Guyana.* CAS 227312 (1, 31,8 mm SL), rio Demerara in Kumaka, 24 Sep 1908, C. Eigenmann. FMNH 85373 (20, 22.8-30.6 mm SL), igarapé Dakara, upstream Luis Chung’s Compound, rio Demerara, 6 Aug 1975, Thomerson, Hicks & Taphorn. MHNH 2002-3517 (1, 42.6 mm SL), “collection de Guyana”. MHNH 2002-3558 (4, 37.7-42.1 mm SL), “collection de Guyana”. USNM 345977 (1, 34.9 mm SL), rio Berbiche drainage, near Dubulai Ranch, KT-2, 30 Jun 1996, T. Bergquist. USNM 345978 (7, 13.1 mm SL), Kaikotin creek, rio Berbice drainage near Dubulai Ranch, 21 Jun 1996, T. Bergquist.

*Coastal drainages, French Guiana.* MCZ 54406 (5, 14.3-24.7 mm SL), Kaw, 12 Jul 1979, R. Mittermier *et al.* MHNG 2200.013 (10 of 15, 15.1-27.9 mm SL), Cabassan, Cayenne. MHNG 2200.036 (6, 23.9-42.3 mm SL), Crique “Magnan”, Moy. Comté, rio Comté, 30 Aug 1978, P. Lamarque. MHNG 2200.037 (2, 30 and 37.6 mm SL), stream affluent of rio Approuague, 1925, C. Ternetz. MHNG 2615.099 (3, 13.1-17.6 mm SL), rio Sinnamary, crique Alaparoubo, 31 Aug 1995, D. Ponton & S. Mérigoux. MHNG 2616.002 (3, 27.3-44.5 mm SL), rio Synnamary, right affluent upstream Saut Dalles, Ponton & Mérigoux, 27 Oct 1995. MHNG 2647.005 (16, 30.7-39.8 mm SL), Crique Grillon, affluent of rio Orapu, rio Mahury drainage, Roura, 4°16’48.1”N 52°27’4.4”W, 07 Nov 2003, C. Weber *et al.* MNHN 2681.094 (1, 37.3 mm SL), stream at forest, opposite to Roche-Mon-Père, about 1 hour downstream Camopi by boat and 15 min dowsntream crique Sikini, rio Oiapok, St Georges-Oyapok, 3º16’56.3”N 52º12’36.6”W, 6 Nov 2006, Fisch-Muller *et al.* MHNG 2724.014 (1, 43.6 mm SL), stream at left bank of before crique Balanfois, rio Arrataye, rio Approuague basin, 21-22 Nov 2010, Montoya-Burgos *et al.* MNHN 2001.0267 (7, 17.0-71.9 mm SL), rio Synnamary, Cayenne, Ponton & Merigoux, 1996. MNHN 2001.0268 (3, 27.0-34.2 mm SL), Synnamary, Cayenne, Ponton & Merigoux, 1995. MNHN 2001.1534 (8, 20.1-36.5 mm SL), rio Oiapoque, Cayenne, Dec 1986, Planquette *et al.* MNHN 2001.1879 (7, 24.8-40.1 mm SL), Crique Japigny, rio Approuague, Cayenne, 17 Nov 1988, Boujard *et al.* MNHN 2002-0863 (1, 37.5 mm SL), crique Litany, “nivraie de Boum-boum au niveau de son abates”, Antécume Pata, St. Laurent du Maroni, 20 Dec 2001, Fermon *et al.* MHNH 2003.0093 (8, 18.4-28.4 mm SL), Crique downstream Antécume Pata, rio Litany, rio Maroni drainage, Commergnat *et al.*, 25 Oct 2002. MHNH 2003.2520 (11, 22.3-40.5 mm SL), rio Approuague, Decouverte, Cayenne, 10 Nov 2003, Weber. MNHN 2004.0355 (1, 34.4 mm SL), rio Serpent, rio Maroni drainage, Nov 2003, Ecobios. MNHN 2004.3030 (5, 18.6-24.5 mm SL), rio Leblond, rio Sinnamary drainage, Trinite, 14 Nov 2001. MNHN 2004.3110 (11, 18.7-32.3 mm SL), rio Leblond, rio Synnamari drainage, Trinite, 13 Nov 2001, Brenm. MNHN 2004.3146 (14, 14.7-29.6 mm SL), stream, rio Ouanary, rio Oiapoque basin, Cayenne, 4º5’49”N 51º57’54”W, 21 Oct 2004, Vigouroux, Roland & Nandrin. MNHN 2011.0305 (2, 32.8 and 37.0 mm SL), Aya, rio Sinnamary drainage, Trinité massif, 4º36’36”N 53º21’32”W, Aug 2009, Melki. ZMA 106.137 (2, 34.6 and 37.1 mm SL), side of Marowijne, 10 Apr 1969, H. Nijssen.

*Coastal drainages, Surinam*. ANSP 189192 (1, 31.6 mm SL), Coropinae Creek, rio Suriname drainage, Republiek, 5°29’57”S 55°12’52”W, 28 Apr 2007, M. Sabaj & P. Willink. MHNG 1554.021 (7, not measured), small stream at forest, Brownsberg National Park, Oct 1976, P. De Rham. MHNG 2200.034 (10 of 15, 12.7-40.4 mm SL), Mooi Wana, crique near bridge at road Albina-Moengo, Maroni, Mar 1962, H. Pijpers. USNM 226869 (8, 17.6-21.8 mm SL), Lana Creek, near 4 Km upstream from intersection West of rio Corantijn, Nickerie, 5°28’N 57°15’W, 7 Sep 1980, R. Vari *et al.* USNM 226873 (1, 27.6 mm SL), creek without name, Corantijn, Nickerie, 5°32’N 57°10’W, 15 May 1980, H. Madarie. USNM 226878 (1, 21.2-31.8 mm SL), Koekwie Kreek, Corantijn, Nickerie, 5°31’N 57°10’W, 15 May 1980, H. Madarie. USNM 311229 (4, 20.5-34.0 mm SL), edge of bay in blackwater creek at savanna, ¾ miles West of Islands Bosbeheer, headquarter at Zanderij, 43 Km South of Paramaribo, Pará, 14 Apr 1969, D. Dunham. USNM 311233 (4, 21.8-27.9 mm SL), black water creek, Islands Bosbeheeri, headquater at Zanderij, 43 km, sul de Paramaribo, 10 Apr 1969, D. Dunham. USNM 332108 (1, 30,7 mm SL), Berlyn, Pará creek, Pará, 14 Sep 1969, H. Pypers. USNM 409768 (4, 27.1-34.1 mm SL), forest stream right tributary of upper rio Paloemeu, upstream of 50 m high waterfall, 2º27’22”N 55º37’35”W, 16 Mar 2012, J. Mol & W. You. ZMA 101.937 (28, 13.5-35.3 mm SL), Onverwarcht, Paran-Zandery road, Oct 1956, J. Yanderkamp. ZMA 101.943 (13, 12.5-36.7 mm SL), Zandery, Bosbivak, 22 Apr 1956, J. Komp. ZMA 104.197 (15, 18.8-36.0 mm SL), small creek at woodzuter camp, foot of Albina Hill, Mar 1962, H. Pijpers. ZMA 104.288 (34, 15.1-35.6 mm SL), Mooi Wana, crique near bridge of Albina-Moengo road, Maroni, Mar 1962, H. Pijpers. ZMA 105.511 (10, 13.4-35.5 mm SL), “Nordelijke tak v/d Tapoeripa-kreek, ca 3 km ten N van Brokopondo”, 13 Nov 1966, H. Nijssen. ZMA 105.694 (16, 21.3-36.4 mm SL), “Jenjeekreek a/d rechteroever u/d rio Suriname, 7.5 km ten North van Botopasi”, 21 Mar 1967, H. Nijssen. ZMA 105.829 (1, 25.7 mm SL), “Jenjeekreek n/d rechteroever v/d rio Nickerie, 12 km (geografisch) te W2W v/d stondansi $ val distr Nickerie”, 5 Apr 1967, H. Nijssen. ZMA 106.105 (46, 16.3-39.7 mm SL), “Marowijne (=Gran) creek gestuwd, geografisch ca 53.5 km ten 2. van Afobaka’s stuwdam, ca 80 cm. Diep, visqif. Brokopondo, rotend blad”, 25 May 1966, H. Nijssen. ZMA 106.107 (13 of 20, 27.1-39.5 mm SL), “Marowijne (=Gran) kreek ca. 58 km ten 2 van Afobaka’s stuwdam (distr. Brokopondo)”, 9 Jun 1966, H. Nijssen. ZMA 106.113 (6, 26.5-39.5 mm SL), “Marowijne (=Gran) kreek ca. 60 km (geografisch) ten 2 van Afobaka’s stuwdam (gestuwd)”, Brokopondo, 8 Jun 1966, H. Nijssen.

*Rio Orinoco basin. Venezuela.* MBUCV 12451 (1, 21.6 mm SL), caño Ajies, affluent of rio Turuepano, at comunidade de Ajies, Sucre, 8 Dec 1980, J. Baskin & O. Castillo. MHNLS 14272 (96, 16.6-25.0 mm SL), caño Ibaruma, caño Guaramo, near headwaters, Serrania de Imataca, Antonio Dias, Delta Amacuro, 8°10’N 60°47’W, 22 Feb 1995, V. Ponte. MHNLS 14278 (46, 12.1-25.1 mm SL), caño Dorina, affluent of rio Araguao, Laguna Morichal Ataibo, comunidade Dorina, Antonio Dias, Delta Amacuro, 8°10’N 60°47’W, 22 Aug 1991, V. Ponte, W. Wilbert & Aquilino. MHNLS 14279 (32, 16.9-24.7 mm SL), caño Capure, caño Jotajana, Pedernales, Delta Amacuro, 9°59’N 62°15’W, Apr 1997, V. Ponte. MHNLS 14285 (49, 16.4-25.5 mm SL), caño Ibaruma, caño Guaramo near cabeceira, mouth of rio Orinoco, Serrania de Imataca, Antonio Dias, Delta Amacuro, 8°10’N 60°47’W, 2 Mar 1995, V. Ponte. MHNLS 14286 (3, 14.5-17.4 mm SL), caño Pagayo, mouth of rio Grande, near Isla Pagayo, Antonio Dias, Delta Amacuro, 8°36’N 60°57’W, 8 Mar 1995, V. Ponte. MHNLS ex 9558 (23, 18.0-28.7 mm SL), caño Guiniquina, Tucupita, Delta Amacuro, 9°29’N 60°59’W, 28 Apr 1992, V. Ponte. MHNLS ex 14284 (10, 16.8-24.8 mm SL), caños El Toro and Acoima, near base camp, first raudal, Serrania de Imataca, Antonio Dias, Delta Amacuro, 8°30’N 61°34’W, 22 Nov 1993, V. Ponte & O. Lasso. SMF 21381 (2, 36.4-37.1 mm SL), “Zuchttier”, South America, Tropicarium ded, 1961.

*Coastal drainages*, *Venezuela*. MBUCV 12440 (69, 14.0-22.3 mm SL), small caño at caño Ajies, approximately 3 km South of comunidade de Ajies, Sucre, 8 Dec 1980, J. Baskin & O. Castillo. MHNLS 12458 (12, 18.3-30.4 mm SL), quebrada la Guacharaca, affluent of rio Cachipo, Punceres, Monagas, 9°55’11”N 63°6’20”W, 7 May 1997, L. Lasso, V. Ponte & D. Figueira. MHNLS ex 12459 (2, 21.5 and 28.2 mm SL), rio Cachipo, setor Sabana II, Alcantarilla N 3, Punceres, Monagas, 9°55’11”N 63°6’20”W, 7 May 1997, L. Lasso, V. Ponte & D. Figueira. MHNLS 12460 (1, 21.3 mm SL), rio Guarapiche, affluent of rio San Juan, South of Santo Antonio de Maturin, Maturin, Monagas, 9°53’53”N 63°1’10”W, 8 May 1997, L. Lasso, V. Ponte & D. Figueira. MHNLS 12461 (16 of 17, 16.2-21.6 mm SL), floodplain at road Zamora camp, near Cachipo, Maturin, Monagas, 9°56’1”N 63°1’42”W, May 1997, L. Lasso, V. Ponte & D. Figueira. MHNLS 12463 (2, 21.0 and 22.6 mm SL), rio Cachipo, Reserva Floresal Guarapiche, Maturin, Monagas, 9°57’8”N 63°0’59”W, 8 May 1997, L. Lasso, V. Ponte & D. Figueira.

**Material examined of *Copella callolepis***

*Type material.* BMNH 1909.4.2.27 (lectotype of *Copeina callolepis*), Amazon, J. Arnoldi. ANSP 39188 (holotype of *Nannostomus stigmasemion*, 10.8 mm SL), tributary of the rio Madeira near Porto Velho, Brazil. BMNH 1952.7.31.3-5 (3 possible syntypes of *Pyrrhulina nigrofasciata*, 24.1, 27.4 and 32.3 mm SL), imported from the upper rio Amazonas, Meinken, 1951. ZMH 1211 (1 possible syntype of *Pyrrhulina nigrofasciata*, 35.8 mm SL, male), imported from the rio Amazonas, Meinken, 1951. ZMH 1212 (3 possible syntypes of *Pyrrhulina nigrofasciata*, 26.3, 29.6 and 32.6 mm SL) imported from the rio Amazonas, Meinken, 1951.

*Non-type material. Rio Amazonas basin, Brazil.* MCZ 6334 (17, 17.1-25.3 mm SL), lago Codajás (lago Badajós), Amazonas [= approximately 3°24’S 62°38’W], 27 Nov-6 Dec 1865, Thayer Expedition. MCZ 6354 (2, 23.3 and 27.6 mm SL), Lago Curupira, Amazonas [=approximately 3°46’S 58°33’W], Nov-Dec 1865, Thayer Expedition. MCZ 170504 (9, 18.3-30.7 mm SL), rio Amazonas at Óbidos [approximately 1º52’S 55º30”W], Nov-Dec 1865, Thayer Expedition. MHNG 2200.022 (14, not measured), rio Purus, Boca de Tapauá, 5º37’27”S 63º11’18”W, 21 Nov 1976, H. Axelrodi, M. Brittan & B. Frank. MHNG 2200.025 (2, not measured), stream 35 km Northwest of Óbidos, 12 Dec 1967, R. Geisler. 2200.026 (1, not measured), Rio Curuçamba, North of Obidos, 9 Dec 1967, R. Geisler. MPEG 129 (1, 17.1 mm SL), rio Tefé drainage, Mucura, Tefé, Amazonas, 5 Aug 1979, M. Golding. MPEG 5795 (3, 24.3-33.2 mm SL), São Raimundo, Almeirim, Pará, 18 Aug 2006, J. Junior. MPEG 9267 (3, 25.0-35.7 mm SL), igarapé Mutum, Juruti, Pará, 2°36’44.8”S 56°11’37.3”W, 2 Aug 2004, W. Wosiacki & A. Bezerra. MPEG 9811 (20, 19.0-38.5 mm SL), headwaters of rio Ribeiro, Juruti, Pará, 2°25’6.8”S 56°26’37.7”W, 18 Sep 2005, W. Wosiacki. MPEG 9817 (59, 19.5-36.3 mm SL), headwaters of rio Ribeiro, Juruti, Pará, 2°25’6.8”S 56°26’37.7”W, 18 Sep 2005, B. Prudente. MPEG 9927 (2, 19.1 and 38.0 mm SL), stream under transmission line at PA 481, Barcarena, Pará, 1°38’S 48°45’W, 4 Sep 2002, A. Sousa & V. Sena. MPEG 10407 (2, 14.7 and 18.3 mm SL), igarapé Santa Rosa, rio Pará, Melgaço, Pará, 22 Nov 2003, L. Montag. MPEG ex 10416 (30, 16.5-27.8 mm SL), stream, FLONA de Caxiuanã, rio Pará, Melgaço, Pará, 1°45.62’S 51°25.46’W, 30 Nov 2004, L. Montag. MPEG 10437 (42, 11.3-16.0 mm SL), stream, FLONA de Caxiuanã, rio Pará, Melgaço, Pará, 4 Nov 2004, L. Montag. MPEG 10477 (5, 11.6-16.9 mm SL), stream, FLONA de Caxiuanã, rio Pará, Melgaço, Pará, 1°45.62’S 51°25.46’W, 30 Nov 2004 L.F.A. MPEG 10487 (2, 14.8 and 16.4 mm SL), stream, FLONA de Caxiuanã, rio Pará, Melgaço, Pará, 29 Nov 2004, L. Montag. MPEG 10493 (27, 10.1-16.0 mm SL), stream, FLONA de Caxiuanã, rio Pará, Melgaço, Pará, 1 Dec 2004, L. Montag. MPEG 10497 (2, 11.4 and 16.9 mm SL), stream, FLONA de Caxiuanã, rio Pará, Melgaço, Pará, 1 Dec 2004, L. Montag. MPEG 10502 (1, 12.7 mm SL), stream, FLONA de Caxiuanã, rio Pará, Melgaço, Pará, 4 Dec 2004, L. Montag. MPEG 10903 (5, 19.0-30.9 mm SL), igarapé Vitória, Juruti, Pará, 2°10’47”S 56°4’38.5”W, 24 Aug 2006, A. Hercos. MPEG 10904 (78, 15.3-32.8 mm SL), igarapé Vitória, Juruti, Pará, 2°10’47”S 56°4’38.5”W, 25 Aug 2006, A. Hercos. MPEG 10908 (2, 19.5 and 26-5 mm SL), igarapé São Francisco, Juruti, Pará, 2°34’50.7”S 55°54’13.8”W, 19 Aug 2006, A. Hercos. MPEG 10914 (1, 19.4 mm SL), igarapé Vitória, Juruti, Pará, 2°10’47”S 56°4’38.5”W, 24 Aug 2006, A. Hercos. MPEG 10919 (60, 14.9-35.1 mm SL), igarapé Vitória, Juruti, Pará, 2°10’47”S 56°4’38.5”W, 24 Aug 2006, A. Hercos. MPEG 10933 (3, 18.2-29.6, igarapé Vitória, Juruti, Pará, 2°10’47”S 56°4’38.5”W, 24 Aug 2006, A. Hercos. MPEG 10934 (1, 31.6 mm SL), igarapé Socó, Juruti, Pará, 2°28’14.2”S 56°00’12.7”W, 18 Aug 2006, A. Hercos. MPEG 10935 (7, 20.8-31.2 mm SL), igarapé Vitória, Juruti, Pará, 2°10’47”S 56°4’38.5”W, 24 Aug 2006, A. Hercos. MPEG 10942 (8, 23.0-35.8 mm SL), igarapé Vitória, Juruti, Pará, 2°10’47”S 56°4’38.5”W, 24 Aug 2006, A. Hercos. MPEG 10949 (1, 16.3 mm SL), igarapé Mutum, Juruti, Pará, 2°36’44.5”S 56°11’35.5”W, 18 Aug 2006, A. Hercos. MPEG 11667 (1, 20.2 mm SL), Lago Cacuri, rio Pará, Melgaço, Pará, 21 Jan 2002, L. Montag. MPEG 12823 (1, 16.8 mm SL), igarapé São Francisco, Juruti, Pará, 15 Dec 2006, L. Montag. MPEG 12824 (7, 16.5-23.2 mm SL), igarapé São Francisco, Juruti, Pará, 13 Dec 2006, L. Montag. MPEG 12825 (1, 16.2 mm SL), igarapé São Francisco, Juruti, Pará, 14 Dec 2006, L. Montag. MPEG 12827 (1, 18.8 mm SL), igarapé São Francisco, Juruti, Pará, 15 Dec 2006, L. Montag. MPEG 12828 (2, 12.3 and 19.6 mm SL), igarapé Socó-Barroso, Juruti, Pará, 12 Dec 2006, L. Montag. MPEG 12829 (2, 13.3 and 14.9 mm SL), igarapé da Ponte, Juruti, Pará, 14 Dec 2006, L. Montag. MPEG 12830 (10, 13.6-27.7 mm SL), igarapé Socó-Barroso, Juruti, Pará, 13 Dec 2006, L. Montag. MPEG 12832 (75, 11.7-25.0 mm SL), igarapé da Ponte, Juruti, Pará, 13 Dec 2006, L. Montag. MPEG 12833 (2, 18.8 and 21.1 mm SL), igarapé Mutum, Juruti, Pará, 11 Dec 2006, L. Montag. MPEG 12834 (7, 15.8-21.6 mm SL), Igarapé Mutum, Juruti, Pará, 12 Dec 2006, L. Montag. MPEG 12835 (2, 15.6 and 18.0 mm SL), igarapé da Ponte, Juruti, Pará, 14 Dec 2006, L. Montag. MPEG 12836 (2, 12.5 and 14.4 mm SL), igarapé Socó-Barroso, Juruti, Pará, 12 Dec 2006, L. Montag. MPEG 12837 (167, 16.8-32.8 mm SL), igarapé da Ponte, Juruti, Pará, 30 Dec 2008, L. Montag. MPEG 12838 (2, 23.0 and 23.7 mm SL), igarapé da Ponte, Juruti, Pará, 14 Dec 2006, L. Montag. MPEG 12839 (4, 12.1-18.1 mm SL), igarapé Mutum, Juruti, Pará, 11 Dec 2006, L. Montag. MPEG 12840 (2, 16.9 and 23.3 mm SL), igarapé da Ponte, Juruti, Pará, 24 Aug 2006, L. Montag. MPEG 12954 (1, 23.2 mm SL), igarapé Mutum, Juruti, Pará, 14 Dec 2006, L. Montag. MPEG 13629 (1, 25.4 mm SL), igarapé Mutum, Juruti, Pará, 2°36’S 56°11.6’W, 12 May 2007, A. Hercos. MPEG 13645 (3, 22.0-23.3 mm SL), igarapé Socó-Barroso, Juruti, Pará, 2°28’12”S 56°00’36”W, 16 May 2007, A. Hercos. MPEG 13663 (66, 23.9-35.8 mm SL), igarapé da Ponte, Juruti, Pará, 2°10.8’S 56°4.7’W, 17 May 2007, A. Hercos. MPEG 13704 (1, 29.3 mm SL), igarapé Mutum, Juruti, Pará, 2°36.76’S 56°11.64’W, 11 May 2007, A. Hercos. MPEG 13708 (6, 17.6-27.1 mm SL), igarapé Socó-Barroso, Juruti, Pará, 2°28’12”S 56°0’36”W, 16 May 2007, A. Hercos. MPEG 13775 (7, 24.4-33.2 mm SL), igarapé da Ponte, Juruti, Pará, 2°10.8’S 56°4.67’W, A. Hercos. MPEG 14282 (3, 14.1-19.9) igarapé Mutum, Juruti, Pará, 2°36.7’S 56°11.6’W, 27 Nov 2007, A. Hercos. MPEG 14318 (1, 30.8 mm SL), rio Araticum, rio Trombetas, Oriximiná, Pará, 1°45’34.7” 56°22’35.5”W, 22 Oct 2007, W. Wosiacki. MPEG 14325 (3, 29.8-36.6 mm SL), rio Patauá, rio Trombetas, Oriximiná, Pará, 1°46’14.5”S 56°21’14.7”W, 7 Jun 2007, W. Wosiacki. MPEG 14332 (1, 27.5 mm SL), rio Araticum, rio Trombetas, Oriximiná, Pará, 1°45’34.7”S 56°22’35.5”W, 17 Oct 2007, W. Wosiacki. MPEG 14385 (11, 11.7-26.0 mm SL), igarapé Mutum, Juruti, Pará, 2°36.76’S 56°11.614’W, 27 Nov 2007, A. Hercos. MPEG 14399 (7, 24.1-36.2 mm SL), rio Patauá, rio Trombetas, Oriximiná, Pará, 1°46’14.5”S 56°21’12.7”W, 18 Oct 2007, W. Wosiacki. MPEG 14400 (8, 11.6-31.4 mm SL), rio Araticum, rio Trombetas, Oriximiná, Pará, 1°46’32.6”S 56°22’32.1”W, 18 Oct 2007, W. Wosiacki. MPEG 14406 (11, 11.6-26.2 mm SL), rio Patauá, rio Trombetas, Oriximiná, Pará, 1°46’14.5”S 56°21’12.7”W, 18 Oct 2007, W. Wosiacki. MPEG 14407 (1, 34.6 mm SL), rio Trombetas, Porto Trombetas, Oriximiná, Pará, 1°45’32.2”S 56°17’58.4”W, L. Montag. MPEG 14451 (3, 14.8-16.4 mm SL), igarapé da Ponte, Juruti, Pará, 2°10.8’S 56°4.667’W, 30 Nov 2007, A. Hercos. MPEG 14464 (13, 12.0-24.4 mm SL), igarapé Socó-Barroso, Juruti, Pará, 2°28’12”S, 56°4.12’W, 1 Dec 2007, A. Hercos. MPEG 14499 (6, 13.1-29.21 mm SL), igarapé da Ponte, Juruti, Pará, 2°10.9’S 56°4.67’W, 30 Nov 2007, A. Hercos. MPEG 14629 (17, 16.9-27.4 mm SL), igarapé da Ponte, Juruti, Pará, 2°10.8’S 56°4.667’W, 30 Nov 2007, A. Hercos. MPEG 14635 (4, 12.4-18.5 mm SL), igarapé Socó-Barroso, Juruti, Pará, 2°28’12”S 56°4.12’W, 1 Dec 2007, A. Hercos. MPEG 15094 (1, 18.9 mm SL), rio Branco, Juruti, Pará, 2°20’46.3”S 51°1’24.3”W, 18 Feb 2008, W. Wosiacki MPEG 15117 (3, 25.5-36.5 mm SL), Trilha T1, Nhamundá, Faro, 1°42’23.3”S 57°2’10.1”W, 25 Aug 2006, L. Montag. MPEG 15182 (39, 13.6-36.3 mm SL), igarapé I.M.T., rio Urucu, rio Solimões, Coari, Amazonas, 4°48’27.1”S 65°1’58.4”W, 6 Feb 2008, L. Montag. MPEG 10756 (1, 20.2 mm SL), igarapé Tamanduá, rio Urucu, rio Solimões, Coari, Amazonas, 4.87°S 65.25°W, 15 Aug 2006, W. Wosiacki. MPEG 10757 (3, 13.1-20.7 mm SL), igarapé Macaco, rio Urucu, rio Solimões, Coari, Amazonas, 4.86°S 65.8°W, 15 Aug 2006, W. Wosiacki. MPEG 10758 (2, 21.0 and 32.7 mm SL), igarapé Tamanduá, rio Urucu, rio Solimões, Coari, Amazonas, 4.87°S 65.25°W, 15 Aug 2006, W. Wosiacki. MPEG 10759 (15, 17.4-24.5 mm SL), igarapé Lontra, rio Urucu, rio Solimões, Coari, Amazonas, 4.88°S 65.15°W, 21 Aug 2006, W. Wosiacki. MPEG 10760 (1, 24.4 mm SL), rio Urucu, rio Solimões, Coari, Amazonas, 4.89°S 65.32°W, 19 Aug 2006, W. Wosiacki. MPEG 10761 (20, 11.0-30.7 mm SL), igarapé Tamanduá, rio Urucu, rio Solimões, Coari, Amazonas, 4.86°S 65.27°W, 15 Aug 2006, W. Wosiacki. MPEG 10762 (18, 10.2-30.6 mm SL), rio Urucu drainage, rio Solimões, Coari, Amazonas, 4.84°S 65.26°W, 18 Aug 2006, W. Wosiacki. MPEG 10763 (4, 13.1-19.3 mm SL), igarapé Lontra, rio Urucu, rio Solimões, Coari, Amazonas 4.87°S 65.2°W, 21 Aug 2006, W. Wosiacki. MPEG 10764 (2, 13.7 and 17.4 mm SL), rio Urucu drainage, rio Solimões, Coari, Amazonas, 4.84°S 65.26°W, 1 Aug 2006, W. Wosiacki. MPEG 10765 (2, 18.0 and 19.1 mm SL), igarapé Lontra, rio Urucu, rio Solimões, Coari, Amazonas, 4.86°S 65.27°W, 21 Aug 2006, W. Wosiacki. MPEG 11200 (1, 25.1 mm SL), rio Urucu drainage, rio Solimões, Coari, Amazonas, 8 Aug 2006, W. Wosiacki. MPEG 11201 (1, 21.6 mm SL), igarapé Tamanduá, rio Urucu, rio Solimões, Coari, Amazonas, 4.86°S 65.27°W, 2 Aug 2006, W. Wosiacki. MPEG 11202 (9, 11.5-19.1 mm SL), rio Urucu drainage, rio Solimões, Coari, Amazonas, 4.84°S 65.26°W, 18 Aug 2006, W. Wosiacki. MPEG 12258 (6, 15.1-27.2 mm SL), igarapé Lontra, rio Urucu, rio Solimões, Coari, Amazonas, 4.84°S 65.9°W, 18 Nov 2006, W. Wosiacki. MPEG 13266 (2, 27.4 and 29.0 mm SL), igarapé Lontra, rio Urucu, BOGPM, rio Solimões, Coari, Amazonas, 4°50’37”S 65°1’50.16”W, 6 Apr 2007, W. Wosiacki. MPEG 13266 (2, 27.4 and 29.0 mm SL), igarapé Lontra, rio Urucu, BOGPM, rio Solimões, Coari, Amazonas 4°50’37”S 65°1’50.16”W, 6 Apr 2007, W. Wosiacki. MPEG 15187 (1, 26.9 mm SL), igarapé Tamanduá, rio Urucu, rio Solimões, Coari, Amazonas, 4°49’43”S 65°1’45.4”W, 8 Feb 2008, L. Montag. MPEG 15203 (10, 13.1-37.0 mm SL), igarapé da Cobra, rio Urucu, rio Solimões, Coari, Amazonas, 4°53’32.7”S 65°11’8.2”W, 6 Feb 2008, L. Montag. MPEG 15227 (2, 7.8 and 11.5 mm SL), igarapé Tamanduá, rio Urucu, rio Solimões, Coari, Amazonas, 4°49’43”S 65°15’19.6”W, 7 Feb 2008, L. Montag. MPEG 15917 (24, 12.1-31.4 mm SL), igarapé Tamoatá, rio Paraconi drainage, FLONA do Pau Rosa, rio Madeira, Maués, Amazonas, 3°44’47.1”S 58°18’7.6”W, 14 Feb 2009, F. Silva. MPEG 16246 (19, 21.9-34.3 mm SL), igarapé Tamanduá, rio Urucu, rio Solimões, Coari, Amazonas, 4°53’18.3”S 65°9’25.5”W, 16 Dec 2008, L. Montag. MPEG 16247 (51, 15.4-33.4 mm SL), igarapé Tamanduá, rio Urucu, rio Solimões, Coari, Amazonas, 4°53’32.7”S 65°11’8.2”W, 28 Jul 2008, W. Wosiacki. MPEG 16248 (45, 11.4-30.5 mm SL), igarapé Tamanduá, rio Urucu, rio Solimões, Coari, Amazonas, 4°52’19.56”S 65°14’58.56”W, L. Montag. MPEG 16249 (33, 17.7-35.4 mm SL), igarapé Tamanduá, rio Urucu, rio Solimões, Coari, Amazonas, 4°53’32.7”S 65°11’8.2”W, 28 Jul 2008, W. Wosiacki. MPEG 16250 (2, 25.3 and 35.4 mm SL), igarapé Tamanduá, rio Urucu, rio Solimões, Coari, Amazonas, 4°51’43.9”S 65°15’54.4”W, 25 Jul 2008, W. Wosiacki. MPEG 16251 (5, 16.1-29.5 mm SL), igarapé Tartaruga, rio Urucu, rio Solimões, Coari, Amazonas, 4°56’13.2”S 65°18’22.5”W, 26 Jul 2008, W. Wosiacki. MPEG 16252 (12, 15.2-30.5 mm SL), rio Urucu, rio Solimões, Coari, Amazonas, 4°48’56”S 65°1’45.4”W, L. Montag. MPEG 16253 (16, 13.1-33.6 mm SL), igarapé Tamanduá, rio Urucu, rio Solimões, Coari, Amazonas, 4°53’32.7”S 65°11’8.2”W, 28 Jul 2008, W. Wosiacki. MPEG 16254 (12, 16.5-27.2 mm SL), igarapé Tartaruga, rio Urucu, rio Solimões, Coari, Amazonas, 4°56’13.2”S 65°18’22.5”W, 26 Jul 2008, W. Wosiacki. MPEG 16266 (2, 20.5 and 21.2 mm SL), igarapé Tamanduá, rio Urucu, rio Solimões, Coari, Amazonas, 4°53’4.9”S 65°13’34.5”W, 15 Jun 2009, L. Montag. MPEG 16267 (3, 16.8-21.5 mm SL), igarapé Tamanduá, rio Urucu, rio Solimões, Coari, Amazonas, 4°51’43.9”S 65°15’54.4”W, W. Wosiacki. MPEG 16268 (13, 13.0-30.1 mm SL), igarapé Tamanduá, rio Urucu, rio Solimões, Coari, Amazonas, 4°53’18.3”S 65°9’25.5”W, 28 Jul 2008, W. Wosiacki. MPEG 16269 (4, 11.7-17.5 mm SL), igarapé I.M.T., rio Urucu, rio Solimões, Coari, Amazonas, 4°49’28.92”S 65°1’50.16”W, 10 Apr 2007, W. Wosiacki. MPEG 16270 (7, 12.2-20.4 mm SL), igarapé Tamanduá, rio Urucu, rio Solimões, Coari, Amazonas, 4°51’43.9”S 65°15’54.4”W, 25 Jul 2008, W. Wosiacki. MPEG 16271 (9, 14.0-38.0 mm SL), igarapé Tamanduá, rio Urucu, rio Solimões, Coari, Amazonas, 4°50’18.96”S 65°1’50.1”W, 4 Apr 2008, W. Wosiacki. MPEG 16272 (74, 13.3-35.4 mm SL), igarapé Tamanduá, rio Urucu, rio Solimões, Coari, Amazonas, 4°53’4.9”S 63°13’34.5”W, W. Wosiacki. MPEG 16273 (2, 17.8 and 20.3 mm SL), igarapé Tartaruga, rio Urucu, rio Solimões, Coari, Amazonas, 4°53’4.3”S 65°20’6.5”W, 27 Jul 2009, W. Wosiacki. MPEG 16274 (6, 18.5-32.0 mm SL), igarapé I.M.T., rio Solimões, Coari, Amazonas, 4°48’55.9”S 65°1’45.5”W, 29 Jun 2009, W. Wosiacki. MPEG 16275 (1, 16.8 mm SL), igarapé Tamanduá, rio Urucu, rio Solimões, Coari, Amazonas, 4°52’20.3”S 65°15’0.7”W, 25 Jul 2008, W. Wosiacki. MPEG 16276 (3, 17.9-20.0 mm SL), igarapé Tartaruga, rio Urucu, rio Solimões, Coari, Amazonas, 4°53’4.3”S 65°13’6.5”W 27 Jul 2008 W. Wosiacki. MPEG 16277 (1, 18.1 mm SL), igarapé Marta, rio Urucu, rio Solimões, Coari, Amazonas, 4°51’39.2”S 65°4’40.4”W, 19 Jul 2008, W. Wosiacki. MPEG 16774 (69, 12.7-25.8 mm SL), rio Urucu drainage, rio Solimões, Coari, Amazonas, 4°S 65°W, 6 Aug 2009, B. Prudente. MPEG 16775 (138, 12.9-34.5 mm SL), rio Urucu drainage, rio Solimões, Coari, Amazonas, 4°53’2.6”S 65°13’33.8”W, 3 Aug 2009, B. Prudente. MPEG 16776 (9, 14.1-21.7 mm SL), igarapé Marta, rio Urucu, rio Solimões, Coari, Amazonas, 4°51’39.3”S 65°4’40.2”W, 3 Aug 2009, B. Prudente. MPEG 16777 (5, 18.7-28.9 mm SL), igarapé Tamanduá, rio Urucu, rio Solimões, Coari, Amazonas, 4°51’44”S 65°15’53.8”W, 2 Aug 2009, B. Prudente. MPEG 16778 (4, 15.1-19.9 mm SL), igarapé Tartaruga, rio Urucu, rio Solimões, Coari, Amazonas, 4°53’4.3”S 65°20’6.5”W, 6 Aug 2009, B. Prudente. MPEG 16779 (1, 12.6 mm SL), rio Urucu drainage, rio Solimões, Coari, Amazonas, 4°52’4.7”S 65°7’25.3”W, 5 Aug 2009, B. Prudente. MPEG 16780 (2, 12.6 and 17.9 mm SL), rio Urucu drainage, rio Solimões, Coari, Amazonas, 4°52’40.7”S 65°14’13.9”W, 7 Aug 2009, B. Prudente. MPEG 16783 (3, 7.9-27.1 mm SL), igarapé Tamanduá, rio Urucu, rio Solimões, Coari, Amazonas, 4°52’18.7”S 65°14’59.6”W, 3 Aug 2009, B. Prudente. MPEG 17408 (25, 20.5-35.7 mm SL), igarapé I.M.T., rio Urucu, rio Solimões, Coari, Amazonas, 4°48’55.2”S 65°1’46.1”W, 4 Aug 2009, B. Prudente. MPEG 17409 (235, 13.8-35.7 mm SL), rio Urucu drainage, rio Solimões, Coari, Amazonas, 4°52’40.7”S 65°14’13.9”W, 7 Aug 2009, B. Prudente. MZUSP 5915 (7, 14.2-20.3 mm SL), lago Jacaré, right margin of rio Solimões, upstream Manacapuru, Manacapuru, Amazonas, 3°17’S 60°38’W, 29-31 Mar 1967, Expedição Permanente da Amazônia. MZUSP 6052 (3, 18.7-21.5 mm SL), Lago Rei, ilha do Careiro, rio Negro drainage, Careiro da Várzea, Amazonas, 3°8’S 59°43’W, 12 Apr 1967, Expedição Permanente da Amazônia. MZUSP 6636 (55, 13.1-21.1 mm SL), stream of lago Manacapuru, rio Solimões, Manacapuru, Amazonas, 3°13’S 60°45’W, 13 Nov 1967, Expedição Permanente da Amazônia. MZUSP 9669 (24, 16.0-21.1 mm SL), lago Supiá, opposite to Codajás, rio Solimões, Codajás, Amazonas, 3°51’S 62°4’W, 24 Aug 1968, Expedição Permanente da Amazônia. MZUSP 9447 (87 of 111, 18.8-28.5 mm SL), igarapé Cajuassú, rio Cuminá, rio Trombetas, Pará, 1°19’S 55°45’W, 26 Jan 1968, Expedição Permanente da Amazônia. MZUSP 23232 (387, 13.3-23.4 mm SL, 10 c&s, 11.8-20.4 mm SL), lago Miuá, upstream Codajás, rio Solimões, Careiro, Amazonas, 3°51’S 60°4’W, 25 Aug 1968, Expedição Permanente da Amazônia. MZUSP 23253 (5, 12.5-15.7 mm SL), rio Solimões drainage, Coari, Amazonas, 4°5’S 63°9’W, 28 Aug 1968, Expedição Permanente da Amazônia. MZUSP 23419 (29, 17.5-25.0 mm SL), lago Buiuçu, Ati-Paraná, Northwest of Fonte Boa, rio Solimões, Fonte Boa, Amazonas, 2°31’S 66°6’W, 11-12 Aug 1968, Expedição Permanente da Amazônia. MZUSP 23489 (6, 24.8-29.6 mm SL), stream near Porto Antunes, rio Jutaí drainage, rio Solimões, Amazonas, 3°4’S 67°3’W, 22 Oct 1968, Expedição Permanente da Amazônia. MZUSP 23490 (8, 17.0-21.2 mm SL), stream near Porto Antunes, rio Jutaí drainage, rio Solimões, Amazonas, 3°4’S 67°3’W, 22 Oct 1968, Expedição Permanente da Amazônia. MZUSP 23495 (5, 19.3-32.3 mm SL), stream at rio Içapó, rio Jutaí mouth, rio Solimões, Amazonas, 2°56’S 66°48’W, 22 Oct 1968, Expedição Permanente da Amazônia. MZUSP 23510 (83 of 111, 22.9-32.0 mm SL, 4 c&s, 23.0-31.7 mm SL), igarapé number three, rio Solimões, Fonte Boa, Amazonas, 2°31’S 66°6’W, 25 Oct 1968, Expedição Permanente da Amazônia. MZUSP 29339 (6, 14.2-28.3 mm SL), Vista Escura, rio Tefé, Amazonas, 3°22’S 64°43’W, 4 Aug 1979, M. Golding. MZUSP 29340 (21, 19.5-23.1 mm SL), Supiã-Pucu, Capim beach, rio Tefé, Tefé, Amazonas, 3°22’S 64°43’W, 21 Jul 1979, M. Golding. MZUSP 31424 (1, 26.7 mm SL), Jurupari, Tefé, Amazonas, 3°22’S 64°43’W, 1 Aug 1979, M. Golding. MZUSP 52646 (3, 16.8-27.0 mm SL), igarapé Saracazinho, rio Trombetas, Porto Trombetas, Pará, 19 Mar 1997, E. Caramaschi & R. Reis. MZUSP 52667 (7, 13.6-34.8 mm SL), igarapé Periquito, upstream road, rio Trombetas, Porto Trombetas, Pará, 17 Mar 1997, E. Caramaschi & R. Reis. MZUSP 52678 (1, 21.7 mm SL), igarapé Saracá, downstream road, rio Trombetas, Porto Trombetas, Pará, 17 Mar 1997, E. Caramaschi & R. Reis. MZUSP 52691 (3, 12.0-23.0 mm SL), igarapé Saracá, upstream road, rio Trombetas, Porto Trombetas, Pará, 15 Mar 1997, E. Caramaschi & R. Reis. MZUSP 52705 (10, 15.8-22.3 mm SL), igarapé Saracá, upstream road, rio Trombetas, Porto Trombetas, Pará, 18 Mar 1997, E. Caramaschi & R. Reis. MZUSP 52739 (1, 17.0 mm), headwater of igarapé Papagaio, rio Trombetas, Porto Trombetas, Pará, 2 Aug 1997, E. Caramaschi & R. Reis. MZUSP 52747 (13, 17.3-28.0 mm SL), igarapé Saracá, upstream, rio Trombetas, Porto Trombetas, Pará, 30 Aug 1997, E. Caramaschi & R. Reis. MZUSP 52758 (2, 28.6 and 32.5 mm SL), igarapé Papagaio, a jusante, braço esquerdo, rio Trombetas, Porto Trombetas, Pará, 30 Aug 1997, E. Caramaschi & R. Reis. MZUSP 52793 (6, 14.2-22.0 mm SL), igarapé Periquito, upstream, rio Trombetas, Porto Trombetas, Pará, 2 Aug 1997, E. Caramaschi & R. Reis. MZUSP 101930 (4, 12.6-29.5 mm SL), igarapé Arapiranga, affluent of left margin of rio Jari, Porto do Figueira, downstream Cachoeira Santo Antônio, Laranjal do Jari, Amapá, 00°48’4”S 52°27’20”W, 25 Mar 2008, C. Moreira, T. Loboda & M. Soares. MZUSP 101931 (5, 16.3-20.5 mm SL), rio Caracuru and stream without name, affluent of rio Caruru, surroundings Vila Santa Maria, rio Jari drainage, Monte Dourado, Amapá, 00°54’55”S 52°34’39”W, 10 Aug 2007, M. Carvalho *et al.* MZUSP 101932 (1, 33.9 mm SL), igarapé Arapiranga, affluent of left margin of rio Jari, Sombra da Mata, downstream Cachoeira Santo Antônio, Laranjal do Jari, Amapá, 00°47’40”S 52°27’9”W, 25 Mar 2008, C. Moreira, T. Loboda & M. Soares. MZUSP 101933 (24, 13.2-34.9 mm SL, 2 c&s, 27.4 and 32.0 mm SL), igarapé Ting Ling, right margin of rio Jari, downstream Chachoeira Santo Antônio, Laranjal do Jari, Amapá, 00°49’54”S 52°40’25”W, 16 Oct 2007, M. Carvalho *et al.* MZUSP 102039 (2, 18.0 and 19.5 mm SL), igarapé Ting Ling, right margin of rio Jari, downstream Chachoeira Santo Antônio, Laranjal do Jari, Amapá, 00°49’54”S 52°40’25”W, 16 Aug 2007, M. Carvalho *et al.* MZUSP 103302 (12. 19.4-34.9 mm SL), igarapé Arapiranga at Balneário Sombra da Mata, left margin of rio Jari, downstream Cachoeira, Laranjal do Jari, Amapá, 00°47’41”S 52°27’10”W, 16 Feb 2009, J. Birindelli, L. Sousa & M. Soares. MZUSP 103273 (4, 23.5-32.8 mm SL), igarapé Arapiranga, affluent of left margin of rio Jari, Porto do Figueira, downstream Cachoeira Santo Antônio, Laranjal do Jari, Amapá, 00°48’5”S 52°27’20”W, 16 Feb 2009, J. Birindelli, L. Sousa & M. Soares. UFRO-I 2757 (2, 13.6 and 16.0 mm SL), igarapé Jatuarana, near confluence of rio Madeira, Porto Velho, Rondônia, 8°49’49.6”S 64°2’45.7”W. UFRO-I 6386 (22, 13.9-21.9 mm SL), stream at Estação Ecológica do Cuniã Madeira, rio Madeira, Porto Velho, Rondônia, 8°4’43.3”S 63°28’34.4”W. UFRO-I 6387 (2, 13.4 and 15.8 mm SL), igarapé Karipuna, upstream mouth of rio Madeira, Porto Velho, Rondônia, 9°12’19.7”S 64°37’57.3”W. USNM 229404 (3, 15.5-18.4 mm SL), Camaleão, ilha de Marchantaria, Amazonas, 3°11’10.9”S 60°7’51.4”W, 27 Sep 1977, P. Bayley. SMF 21321 (37, 17.0-26.6 mm SL), Manacapuru, Solimões.

*Peru.* ANSP 165007 (14, 18.0-35.2 mm SL), small stream about 70 km South of Iquitos, near Jenaro Herrera, rio Ucayali, Loreto, 5°0’0’’S 73°38’0’’ W, 23 Aug 1989, P. Fromm *et al.* ANSP 165043 (3, 20.5-25.6 mm SL), stream of rio Yanayacu, West of Miraflores, rio Maranon, rio Ucayali, Loreto, 4°42’0’’S 74°17’0’’W, 21 Aug 1989, P. Fromm *et al.* ANSP 167195 (11, 16.6-25.8 mm SL), small stream five minutes by foot from rigth margin of rio Nanay, about 1 km upstream Minchana, Loreto, 3°53’0’’S 73°27’0’’W, 11 Sep 1989, Dan & P. Fromm. ANSP 167196 (2, 20.5 and 22.1 mm SL), brook crossing left trail about 10 min from Minchana, rio Nanay, Loreto, 3°53’0’’S 73°27’0’’W, 11 Sep 1989, Dan & P. Fromm. ANSP 167197 (5, 10.9-21.2 mm SL), creek about 15 min paddle up rio Momon from Amazon camp, rio Ucayali, Loreto, 3°42’0’’S 73°13’0’’W, 3 Sep 1989, Dan & P. Fromm. ANSP 167198 (5, 22.5-32.4 mm SL), book crossing left trail about 20 min walk from Minchana, rio Nanay, Loreto, 3°53’0’’S 73°27’0’’W, 3 Sep 1990, Dan & P. Fromm. ANSP 167199 (23, 12.6-29.6 mm SL), stream upstream Nina Rumi, left margin of rio Nanay, Loreto, 3°44’0’’S 73°20’0’’W, 9 Sep 1990, Dan & P. Fromm. ANSP 167200 (56, 16.3-28.9 mm SL), stream almost dry, about six Miles up rio from confluence from rio Momon, left bank, Loreto, 3°46’0’’S 73°15’0’’ W, 23 Aug 1989, Dan & P. Fromm. ANSP 167201 (38, 11.4-27.5 mm SL), Laguna Rocafuerte, rigth margin of rio Nanay, about six miles uo from confluence with rio Momon, Loreto, 3°46’0’’S 73°15’0’’ W, 23 Aug 1990, Dan & P. Fromm. ANSP 167222 (1, 21.8 mm SL), stream almost dry at left margin of rio Nanay, about five miles downstream Santa Clara, Loreto, 3°45’0’’S 73°17’0’’ W, 4 Sep 1990, Dan & P. Fromm. ANSP 178193 (4, 14.5-19.1 mm SL), caño Santa Rita, rigth margin, trybutary of rio Nanay, 3.32 miles Northwest of Iquitos, near Pampa Chica community, Maynas, Loreto, 3°45’23’’S 73°17’28’’W, 2 Aug 2001, M. Sabaj *et al.* ANSP 180757 (12, 20.3-26.5 mm SL), mouth of stream feeding laguna connected to rio Nanay, about two hours upstream from Santa Clara by outboard motor boat, Maynas, Loreto, 3°52’21’’S 73°32’43’’ W, 16 Aug 2003, M. Sabaj *et al.* ANSP 190681 (1, 18.0 mm SL), rio Nanay, large Sandy beach on downstream end of island upstream from Santa Clara, Southwest of Iquitos, Loreto, 3°46’45’’S 73°22’6’’ W, 14 Aug 2003, M. Sabaj, N. Salcedo & B. Sidlauskas. MHNG 2200.024 (1, not measured), swamp at tributary of rio Corrientes. MZUSP 85597 (31, 11.0-33.1 mm SL), stream at Km 3 of Jenaro Herrera-Colonia Angamos road, upstream “aguajal”, Requena, Loreto, 4°55.889’S 73°38.756’W, 14 Jan 2004, H. Ortega *et al.* MZUSP 85603 (20, 17.5-34.0 mm SL, 2 c&s, 23.0-28.8 mm SL), stream about 0.3 km North of Km 3.9 of Jenaro Herrera-Colonia Angamos road, 4°53.91’S 73°38.366’W, 16 Jan 2004, H. Ortega, R. Reis & F. Lima. MZUSP 26697 (8, 16.3-31.1 mm SL), Jenaro Herrera, Arboreto, Requena, Loreto, 30 Jun 1979, P. de Rham. MZUSP 26698 (1, 21.9 mm SL), Jenaro Herrera, Loreto, 20 Aug 1979, H. Ortega. NRM 37465 (8, 17.8-24.8 mm SL), unnamed stream, about 200 m to the left of the road from Iquitos, Just before Santa Clara, rio Nanay drainage, Loreto, 7 Sep 1981, S. Kullander & A. Urtega. NRM 18655 (15, 12.4-27.5 mm SL), quebrada Sapuena at Km 10 on road Jenaro Herrera-Colonia Angamos, Loreto, 31 Aug 1983, S. Kullander *et al.* NRM 18662 (64, 14.4-31.3 mm SL), stream in arboretum at Km 3 on road Jenaro Herrera-Colonia Angamos, Loreto, 2 Sep 1983, S. Kullander, A. Urtega & T. Townshend. NRM 17038 (1, 27.1 mm SL), stream in civil village intended for fish pond close to source, rio Yavarí system, Colonia Angamos, Loreto, 1 Aug 1984 S. Kullander *et al.* NRM 49109 (3, 25.3-34.6 mm SL), Quebrada de las Granjas, rio Putumayo, Loreto, 21 Jul 1986, S. Kullander *et al.* NRM 49108 (27, 17.3-35.1 mm SL), fish pond in construction at Southwest village margin, rio Putumayo drainage, Loreto, 21 Jul 1986 S. Kullander *et al.* NRM 48119 (4, 14.1-25.6 mm SL), three small stream at Eastern margin of village, rio Putumayo, Loreto, 21 Jul 1986, S. Kullander *et al.*

***Copella compta***

*Type material.* CAS 60496 [ex IU 17693] (holotype of *Copeina compta*, 50.6 mm SL), CAS 60497 [ex IU 17694] (17 paratypes of *Copeina compta*, 18.3-37.6 mm SL), MCZ 31568 (3 paratypes of *Copeina compta*, 23.6-39.1 mm SL), MHNG 2200.38 (1 paratype of *Copeina compta*, 33.3 mm SL), SU 18070 (9 paratypes of *Copeina compta*, 21.4-55.7 mm SL, 1 c&s), creek above São Gabriel Rapids, rio Negro, Amazonas Brazil, C. Ternetz, 28-30 Jan 1925.

*Non-type material. Rio Amazonas basin, Brazil.* INPA 9162 (37, 16.9-68.9 mm SL, 4 c&s, 17.0-50.8 mm SL), stream at camp, rocks, Morro dos Seis Lagos, 23 Mar 1990, J. Zuanon, *et al*. LBP 7076 (34, 14.9-41.4 mm SL), affluent of igarapé Miuá, rio Negro, São Cabriel da Cachoeira, Amazonas, 0º6.2’S 66º53.7’W, Oliveira *et al.*, 14 Aug 2008. MZUSP 27457 (2, 28.4 and 29.4 mm SL), Morro dos Seis Lagos, São Gabriel da Cachoeira-Cucui, rio Negro, São Gabriel da Cachoeira, Amazonas, 0º7'S 67°5'W, 16 Nov 1982, L. Portugal. *Venezuela.* AMNH 231274 (20, 16.9-59.5 mm SL), tributary of rio Mawarinuma, at Serra da Neblina base camp, Amazonas, 0º55'N 66°10'W, 7-11 Feb 1984, C. Ferraris, W. Buck & R. Royero-I. AMNH 231273 (11, 12.0-28.0 mm SL), tributary of rio Mawarinuma, at Serra da Neblina base camp, Amazonas, 0º55'N 66°10'W, 5 Feb 1984, C. Ferraris, W. Buck & H. Olivarria. AMNH 231275 (9, 13.0-46.0 mm SL), tributary of rio Mawarinuma, at Serra da Neblina base camp, Amazonas, 0º55'N 66°10'W, 10 Feb 1984, C. Ferraris & R. Royero-I.

**Material examined of *Copella eigenmanni***

*Type material.* BMNH 1869.7.25.6 [ex 1869.7.25.6-7] (Lectotype of *Copeina eigenmanni*, 37.7 mm SL), Bogotá, Colombia, Cutter. BMNH 1869.7.25.7 (1 paralectotype of *Copeina eigenmanni*, 29.2 mm SL), Bogotá, Colombia, Cutter. CAS 60494 [ex IU 13251a] (holotype of *Copeina metae*, 28.8 mm SL), CAS 60495 [ex IU 13251] (26 paratypes of *Copeina metae*, 11.7-30.5 mm SL), SU 24656 (2 paratypes of *Copeina metae*, 24.5-28.8 mm SL), Barrigona [=Puerto Barrigón], rio Meta, Colombia, M. Gonzáles, 1914.

*Non-type material. Rio Orinoco basin*, *Colombia.* ANSP 84390 (1, 20.0 mm SL), Villavicencio, Meta, Oct 1940, C. Miles. ANSP 121628 (1, 30.4 mm SL), Lomalinda, near rio Ariari, Southeast of Villavicencio, Meta, 21 Aug 1969, E. Hugghins. ANSP 128621 (5, 22.2-38.4 mm SL), stream into Lake Mozambique (Mozambique ranch), North of ranch house, rio Meta drainage, Meta, 3°58’N 73°4’W, 25 May 1969, J. Böhlke & N. Foster. ANSP 128751 (21, 20.0-33.0 mm SL, 1 c&s, 28.4 mm SL), small stream at Hacienda Humacita, flowing generally South, presumably the general Lake Mozambique complex, 250 ft of stream collected, mostly above the bridge, Meta, 3°58’N 73°4’W, 21 Feb 1972, J. Böhlke & N. Foster. ANSP 128752 (10, 19.4-29.9 mm SL), laguna North of Lake Mozambique, Hacienda Mozambique, rio Meta drainage, Meta, 3°58’N 73°4’W, 23 Mar 1971, J. Böhlke *et al.* ANSP 128753 (50, 16.4-29.5 mm SL), Rancho El Viento, small embayment in long chain of continuous lagunas draining into rio Meta, 4°39’N 72°39’W, 30 May 1969, J. Böhlke *et al.* ANSP 128754 (1, 22.9 mm SL), small stream flowing generally South, entering general Lake Mozambique complex, Hacienda Humacita, rio Meta drainage, Meta, 3°58’N 73°4’W, 23 Mar 1971, J. Böhlke *et al.* ANSP 128755 (5, 21,1-25,2 mm SL), caño Angosturas at Hacienda Humacita and just below, rio Meta drainage, Meta, 3°58’N 73°4’W, 28 Mar 1971, J. Böhlke *et al.* ANSP 128756 (50, 15.3-24.1 mm SL), small stream flowing generally South, presumably general Lake Mozambique complex, Hacienda Humacita, Meta, 3°58’N 73°4’W, 24 Feb 1972, J. Böhlke *et al.* ANSP 128757 (26, 18.2-28.0 mm SL), caño Rico em La Defensa, Northwest of Laguna Mozambique, becomes Cano Buenaventura before entering rio Negro, Meta, 3°59’N 73°8’W, 6 Mar 1971, S. Roback. ANSP 128758 (50, 15.1-24.5 mm SL), laguna West of North shore of Lake Mozambique (second one to the Northwest) at Hacienda Mozambique, with outlet to the rio Metica, rio Meta drainage, Meta, 3°58’N 73°4’W, 6 Mar 1971, S. Roback. ANSP 128759 (87 de 111, 22.6-32.2 mm SL), small stream flowing generally South at Hacienda Humacita, entering general Lake Mozambique complex at, rio Meta drainage, Meta, 3°58’N 73°4’W, 6 Mar 1971, S. Roback. ANSP 128760 (4, 15.6-22.3 mm SL), caño El Viento, Finca El Viento South of Matazul, Meta, 4°8’N 72°39’W, 6 Mar 1971, S. Roback. ANSP 128761 (12, 13.4-23.9 mm SL), caño Emma, Finca El Viento, approximately 33.5 km Northwest of Puerto López, Meta, 4°8’N 72°39’W, 6 Mar 1971, S. Roback. ANSP 130085 (3, 15.5-20.9 mm SL), pond at Hacienda El Viento, 32 km Northeast of Puerto-López, Meta, 15 Feb 1973, J. Richardson Jr. ANSP 130088 (1, 15.7 mm SL), Mozambique Ranch pond, Meta, 6 Dec 1969, S. Roback. ANSP 130089 (7, 10.9-2.9 mm SL), pond at Rancho El Viento, Meta, 10 Dec 1969. ANSP 131689 (1, 17.7 mm SL), pool about 10 km East of Villavicencio, Meta, 6 Mar 1971, S. Roback. Foster. ANSP 131690 (7, 10.6-19.9 mm SL), Pond 1 in Palm Forest, East of Hacienda Mozambique, Meta, 7 Mar 1971, S. Roback. ANSP 131691 (1, 19.8 mm SL), Pond 1, 16 km Southwest of Puerto Lopes, third dam from La Balsa road, Meta, 2 Mar 1971, S. Roback. ANSP 131692 (1, 13.6 mm SL), Pond 2 in Palm Forest East of Hacienda Mozambique, 16 km Southwest of Puerto Lopez, Meta, 7 Mar 1971, S. Roback. ANSP 131693 (1, 15.7 mm SL), Pond 3, 16 km Southwest of Puerto López, first dam from La Balsa road, Meta, 1 Mar 1971, S. Roback. ANSP 133999 (1, 22.5 mm SL), Quebrada Venturosa, between La Balsa and Puerto López, rio Meta drainage, Meta, 4°5’N 72°58’W, 21 Mar 1975, J. Böhlke, W. Saul & L. Fuiman. ANSP 134454 (1, 21.7 mm SL), trybutary of caño La Raya (first caño North of La Siberia), rio Meta drainage, Meta, 4°5’N 73°5’W, 10 Mar 1975, J. Böhlke, W. Saul & L. Fuiman. ANSP 141556 (15, 13.1-23.6 mm SL), hallow seepage in low area West of tip of lake, before creek on East side Hacienda Mozambique, Meta, 3°58’N 73°4’W, 29 Feb 1972, D. & N. ANSP 178757 (214, 10.6-27.7 mm SL), hallow seepage in low area West of tip of lake, before creek on East side Hacienda Mozambique, Meta, 3°58’N 73°4’W, 27 Mar 1971, J. Böhlke *et al.* ANSP 191042 (22, 21.6-36.9 mm SL), seepage run-off into Lake Mozambique (North side), Mozambique ranch, Meta, 3°58’N 73°4’W, 25 May 1969, J. Böhlke & N. Foster. ANSP 191044 (2, 25.0 and 34.0 mm SL), seepage run-off into Lake Mozambique (North side), Mozambique ranch, Meta, 3°58’N 73°4’W, 25 May 1969, J. Böhlke & N. Foster. CZUT-IC 4084 (1, 20.0 mm SL), Canõ Guamirza, Guainia, 2°58’13”N 67º50’13”W, 15 Dec 2009-29 May 2010, F. Villa & A. Ortega. CZUT-IC 4089 (2, 19.5 and 23.7 mm SL), rio Guainia, San Jose-Playa community, Guainia, 4º41’44”N 68º2’7”W, 10 Dec 2009-29 May 2010, F. Villa & A. Ortega. CZUT-IC 4613 (4, 16.3-17.3 mm SL), caño Cubideño, Cubideño bridge, rio Meta drainage, Casanare, 24 Sep 2010, A. Ortega & A Urbano. CZUT-IC 4664 (4, 20.1-26.7 mm SL), Reserva Palmarito, rio Cravo Sur, rio Meta, Casanare, 25 Sep 2010, A. Ortega & A Urbano. CZUT-IC 5088 (1, 24.0 mm SL), Canõ Guamirza, Guainia, 2°58’13”N 67º50’13”W, 15 Dec 2009-29 May 2010, F. Villa & A. Ortega. FMNH 112220 (20 de 73, 21.5-25.6 mm SL), tributary of rio Manacacias, 1 km West of Puerto Guaitán, Meta, 3 Apr 1974, Thomerson *et al.* IAvH 876 (33, 16.0-24.7 mm SL), Puerto Gaitán, Meta, 3°13’49”N 73°52’39”W, 22 Feb 1984, M. Blanco *et al.* IAvH 885 (63, 11.8-25.1 mm SL), caño Muco at San Francisco farm, Puerto Gaitán-Gaviotas road, rio Vichada drainage, Meta, 3°13’49”N 73°52’39”W, 9 Dec 1983, M. Blanco *et al.* IAvH 1144 (250, 14.7-32.3 mm SL), caño Muco at Hacienda San Francisco, 22 km of Puerto Gaitán-Gaviotas road (deviation Km 17), Meta, 3°13’49”N 73°52’39”W, 22 Mar 1984, M. Blanco *et al.* IAvH 1205 (5, 14.4-21.0 mm SL), caño Muco at San Francisco farm, Puerto Gaitán-Gaviotas road, Meta, 3°13’49”N 73°52’39”W, 23 Feb 1984, M. Blanco *et al.* IAvH 1420 (11, 15.6-24.5 mm SL), Centro Gaviotas, caño Elvimida, Cumaribo, Vichada, 4°35’N 70°52’W, 23 Feb 1984, M. Blanco *et al.* IAvH 1575 (1, 22.4 mm SL), caño San Francisco, rio Muco drainage, Puerto Gaitán, Meta, 3°28’N 73°40’W, 23 Feb 1984, M. Blanco *et al.* IAvH 1590 (64, 12.0-32.4 mm SL), caño San Francisco, rio Muco drainage, Puerto Gaitán, Meta, 3°28’N 73°40’W, 23 Feb 1984, M. Blanco *et al.* IAvH 1591 (35, 11.7-21.0 mm SL), caño San Francisco, rio Muco drainage, Puerto Gaitán, Meta, 3°28’N 73°40’W, 23 Feb 1984, M. Blanco *et al.* IAvH 2284 (1, 23.0 mm SL), caño NN, affluent of caño Bocón, rio Inírida drainage, Puerto Inírida, Guainia, 3°39’N 68°1’W, 11 Mar 2007, H. Thorbjörn. IAvH 2431 (1, 14.6 mm SL), caño Muco at Hacienda San Francisco, 22 km of Puerto Gaitán-Gaviotas road (deviation Km 17), rio Vichada drainage, Meta, 3°13’49”N 73°52’39”W, 22 Mar 1984, Blanco *et al.* IAvH 2492 (20, 15.5-23.7 mm SL), Morichal near rio Yucao, rio Meta drainage, Puerto López, Meta, 4°5’N 72°58’W, 27 Nov 1978, Scully & Garzón. IAvH 3404 (20, 16.4-27.0 mm SL), caño Moriche, rio Meta drainage, Casanare, 11 Aug 1994, F. Rodriguez. IAvH 4179 (5, 14.8-18.2 mm SL), Madre Vieja, rio Tomo basin, rio Meta drainage, Cumaribo, Vichada, 5°21’55”N 67°51’33,3”W, 17 Feb 2004, Maldonado *et al.* IAvH 7906 (2, 26.0 and 28.1 mm SL), Quebrada La Palomera, affluent of rio Cravo Sur, rio Meta drainage, Yopal, Casanare, 5°22’9.5”N 72°16’17.3”W, 10 Apr 2006, Bogotá *et al.* IAvH 7907 (2, 27.8 and 28.5 mm SL), stream affluent of rio Caja vereda Jaguito, rio Meta drainage, Tauramena, Casanare, 5°3’6.1”N 72°45’22.6”W, 11 Apr 2006, Bogotá *et al.* IAvH 9938 (17, 13.8-26.4 mm SL), caño Fruta, 1 hour upstream Sarrapia, rio Meta drainage, Cumaribo, Vichada, 4°26’28,8”N 67°55’W, 11 Mar 2007, Bogotá & F. Villa. IAvH 10774 (1, 18.0 mm SL), stream of rio Tomo drainage, Cumaribo, Vichada, 5°21’32”N 67°51’46.2”W, 30 Jan 2004, Maldonado *et al.* IAvH 11223 (13, 19.7-50.4 mm SL), caño La Insula, Alto Neblinas way, Finca Unillanos, rio Meta drainage, Puerto Gaitán, Meta, 4º18’59.8”N 72º39’57.6W, 11 Mar 2007, Aya & Rincón. IAvH 11269 (4, 19.4-22.7 mm SL), laguna El Tinije, Aguazul, Casanare. IAvH ex 3405 (24, 12.4-25.0 mm SL), caño Agua Limón, Orinoco, Arauquita, Arauca, 6°55’N 70°58’W, 2 Nov 2003, G. Castaño. ICNMNH 942 (40, 20.6-36.4 mm SL), El Presentado, quebrada Tascona, affluent of rio Guacavia, rio Metica, rio Meta drainage, Cumaral, Meta, 30 Jan 1985, C. Román-Valencia. ICNMNH 1037 (28, 18.3-33.5 mm SL), caño Limón, caño Verde, rio Arauca drainage, 7 Mar 1977, P. Cala & P. Velásquez. ICNMNH 1382 (32, 16.9-24.3 mm SL), rio Tomo at Estación Tuparro, Vichada, 21 Feb 1972, P. Cala. ICNMNH 1383 (62, 12.7-27.8 mm SL), caño Baronas, Guaviare, Puerto Lleras, Meta, 2 Dec 1976, P. Cala. ICNMNH 1385 (4, 39.6-56.4 mm SL), quebrada Yaguaso, rio Meta drainage, Aguazul, Casanare, 8 Sep 1981, P. Cala. ICNMNH 1386 (17, 13.4-37.8 mm SL), caño Macapay, rio Meta drainage, Maya, Cundinamarca, 18 Jun 1976, P. Cala. ICNMNH 1389 (5, 16.3-42.9 mm SL), caño Carraño, rio Muco, rio Vichada drainage, Puerto Gaitán, Meta, 17 Jun 1974, P. Cala. ICNMNH 1395 (5, 31.2-50.1 mm SL), rio Muco, rio Vichada drainage, 14 Jul 1974, P. Cala. ICNMNH 1406 (3, 19.6-28.4 mm SL), caño Urimica, rio Tomo drainage, Gaviotas, Vichada, 15 Jul 1974, P. Cala. ICNMNH 1409 (22, 14.3-18.9 mm SL), rio Inírida, Puerto Inírida, Guainía, 8 Jan 1972, P. Cala. ICNMNH 1410 (58, 10.8-25.4 mm SL), caño Cajuy, Hda. Tanané, rio Meta drainage, Meta, 18 Jan 1967, C. Velásquez. ICNMNH 1428 (1, 16.5-26.9 mm SL), caño Zuria, rio Meta drainage, Villavicencio, Meta, 12 Nov 1987, P. Cala & P. Benavides. ICNMNH 1432 (9, 20.4-26.1 mm SL), caño Caimán affluent of rio Muco, rio Vichada drainage, Vichada, 14 Jul 1974, P. Cala. ICNMNH 1433 (1, 23.5 mm SL), rio Arauca, Saravena, Arauca, 5 Mar 1977, P. Cala. ICNMNH 1435 (51, 13.0-46.2 mm SL), caño Limón, caño Poporio, rio Ariari drainage, Guaviare, Meta, 29 Nov 1976, P. Cala. ICNMNH 1939 (1, 38.6 mm SL), rio Yucao, rio Meta drainage, Puerto Gaitán, Meta, 1 Nov 1992, W. Castro & R. Sánchez. ICNMNH 10485 (1, 32.1 mm SL), confluence of caño Chaparral and caño Grande, rio Tomo drainage, Vichada, 1 Jul 2004, S. Gavassa. ICNMNH 11177 (10, 11.4-24.6 mm SL), caño Pica Pico, La Hermosa, Finca Nicaragua, rio Meta drainage, Paz de Ariporo Casanare, 1 Oct 2004, M. Gutiérrez. ICNMNH 11219 (5, 12.2-14.8 mm SL), caño Moreno La Hermosa, Finca Nicaragua, rio Meta drainage, Paz de Ariporo, Casanare 5°38’15.4”N 70°12’41”W, 9 Nov 2004, M. Gutiérrez. ICNMNH 11251 (3, 17.6-19.6 mm SL), estero 1, La Hermosa, Finca Nicaragua, rio Meta drainage, Paz de Ariporo, Casanare, 5°37’31.61”N 70°15’16.8”W, 29 Oct 2004, M. Gutiérrez. ICNMNH 11268 (1, 22.8 mm SL), estero 2, La Hermosa, Finca Nicaragua, rio Meta drainage, Paz de Ariporo, Casanare, 31 Oct 2004, M. Gutiérrez. ICNMNH 11276 (10, 8.7-23.1 mm SL), estero 3, La Hermosa, Finca Nicaragua, rio Meta drainage, Paz de Ariporo, Casanare, 5°36’51.8”N 70°14’36.1”W, 8 Nov 2004, M. Gutiérrez. ICNMNH 11335 (18, 12.9-19.4 mm SL), laguna Rebalse, La Hermosa, Finca Nicaragua, rio Meta drainage, Paz de Ariporo, Casanare 5°36’11.1”N 70°16’45.1”W, 1 Nov 2004, M. Gutiérrez. ICNMNH 12189 (1, 22.6 mm SL), caño Porfía, affluent of rio Yucao, rio Meta drainage, Puerto Gaitán, Meta, 1 Nov 2004, Proyecto Ornamentales Orinoco. ICNMNH 12190 (1, 17.8 mm SL), caño La Berraquera, affluent of rio Yucao, rio Meta drainage, Puerto Gaitán, Meta, 1 Nov 2004, Proyecto Ornamentales Orinoco. ICNMNH 12192 (3, 25.5-39.4 mm SL), Centro de Acopio, rio Guainía, Puerto Inírida, 1 Feb 2005, Proyecto Ornamentales Orinoco. ICNMNH 12879 (10, 13.0-20.6 mm SL), caño Porfía, affluent of rio Yucao, rio Meta, Puerto Gaitán, Meta, 1 Nov 2004, Proyecto Ornamentales Orinoco. ICNMNH 12881 (2, 24.2 and 26.9 mm SL), Fazenda la Neblina, caño la Neblina affluent of rio Manacacías, rio Meta, Puerto Gaitán, Meta, 1 Nov 2004, Proyecto Ornamentales Orinoco. ICNMNH 12979 (6, 18.5-33.3 mm SL), caño la Pecuca affluent of rio Guacaula, rio Meta, Cumaral, Meta, 1 Oct 1990, G. Galvis. ICNMNH 12980 (5, 23.0-34.0 mm SL), caño Caibe, rio Meta drainage, Cumaral, Meta, 1 Apr 1995, G. Galvis. ICNMNH 13069 (1, 24.7 mm SL), caño Seco, affluent of rio Acacías, rio Meta, La Esmeralda, Meta, 1 Apr 1995, J. Mojica & Estudiantes Sistemática Animal. ICNMNH 15841 (1, 24.8 mm SL), mouth of rio Orotoy, affluent of rio Acacias, rio Meta drainage, Acacias, Meta, 1 Feb 2005, H. Mojica & H. Casas. MLS 937 (10, 25.3-29.8 mm SL), secondary branch of rio Cusiana, Yopal, Casanare, 4°54’8,5”N 72°30’8,8”W, Buitrago, Uriel. FMNH 55166 (4, 11.7-30.5 mm SL), rio Meta at Barrigona, Orinoco, M. Gonzales. MLS 939 (7, 17.0-30.2 mm SL), quebrada El Zorno, Yopal, Casanare, 5°10’12,6”N 72°29’59,8”W Buitrago, Uriel. MHNG 2200.29 (7, 21.2-26.6 mm SL), upper rio Meta, about 30 miles Southeast of Villavicencio, Orinoco, Dec 1963, R. Socolof. MHNLS 23579 (6, 17.2-20.9 mm SL), marginal branch of Macasabe lagoon, right bank of rio Guaviare, Vichada, 3°57’20.3”N 67°56’32.2”W, 5 Aug 2008, C. Lasso *et al.* MHNLS 23580 (2, 17.6 and 18.8 mm SL), marginal branch of Macasabe lagoon, right bank of rio Guaviare, Vichada, 3°57’20.3”N 67°56’32.2”W, 5 Aug 2008, C. Lasso *et al.* MHNLS 23755 (5, 26.9-38.7 mm SL), quebrada affluent of caño Tonina, tributary of left bank of caño Bocon, Los Pozos de Floro, Vichada, 3°39’38,3”N 68°3’53,7”W, 1 Aug 2008, C. Lasso *et al.* SIU 311376 (2, 24.0 and 31.6 mm SL), tributary of rio Manacacias, 1 km West of Puerto Gaitán, Meta, 3 Apr 1974, Thomerson *et al.* SU 50584 (1, 33.6 mm SL), floodplain stream at left bank of rio Oteguaza, 2.3 miles upstream Três Esquinas, 0°48’N 75°15’W, 8 Feb 1958, White *et al.* USNM 311008 (88, 13.7-32.6 mm SL), rio Uaupés, Mitu, 18 Aug 1989, H. Axelrod. USNM 323152 (6, 16.6-19.4 mm SL), caño Muco about 15 km toward Puerto Coreno from Puerto Gaitán, Orinoco, Vichada, 15 Jul 1992, Thomerson *et al.* MLS 313 (2, 29.0 and 36.0 mm SL), caño Carnicerías, Cumaral, Meta, 4°11’59”N 73°12’59,2”W. MLS 923 (17, 20.6-30.0 mm SL), quebrada El Moricho, Northest of rio Charte, Yopal, Casanare, 4°44’41.2”N 72°10’34.9”W, Buitrago, Uriel.

**Rio Orinoco basin, Venezuela.** AMNH 230891 (4, 24.0-31.6 mm SL), rio cora-cora, fourty minutes upstream Yutaje camp, rio Ventuari, Amazonas, 5°36’39”N 66°7’57”W, 27 Apr 1999, S. Schaefer, F. Provenzano & R. Rojas. AMNH 233001 (4, 17.6-22.8 mm SL), rio Cuao, Raudal Cielo, West side of Isla del Cielo, fifteen minutes by foot from SAS01-04 base camp, Amazonas, 7 Mar 2001, S. Schaefer, F. Provenzano & J. Baskin. ANSP 130036 (80, 14.1-19.6 mm SL), trybutary of rio Guariquito, 24 km Southeast of Calabozo on Estrada to Cazorla, Guarico, 8°38’N 67°20’W, 27 Nov 1966, N. Foster. ANSP 135721 (14, 16.2-29.0 mm SL), rio Tauca on Maripa-Ciudad Bolivar road, Bolivar, 7°28’N 64°52’W, 5 Feb 1977, J. Böhlke & W. Saul. ANSP 135737 (46, 19.2-33.1 mm SL), Morichal Zamorai (stream) between rios Tauca and Tiquire at Maripa-Cuidad Bolivar road, Bolivar, 3 Feb 1977, J. Böhlke, W. Saul, Ferrer-Veliz. ANSP 146228 (31, 19.0-30.0 mm SL), Morichal Zamorai (stream) ) between rios Tauca and Tiquire at Maripa-Cuidad Bolivar road, Bolivar, 7°28’N 64°54’W, 3 Feb 1972, J. Böhlke *et al.* ANSP 149409 (3, 23.4-29.1 mm SL), Puerto Ayacucho, Amazonas, 13 Mar 1950, J. Rivero. ANSP 159160 (1, 20.1 mm SL), shore and backwater of rio Sipapo at Raudal del Caldero, about 3 km above confluence with rio Orinoco, Amazonas, 5°4’N 67°46’W, 14 Nov 1985, B. Chernoff *et al.* ANSP 159162 (36, 16.0-25.8 mm SL), Morichal 26.9 km of Puerto Ayacucho, along Puerto Ayacucho-Caicara road, Orinoco, 15 Nov 1985, B. Chernoff *et al.* ANSP 159170 (8, 18.1-32.1 mm SL), Morichal Poso Vagabundo, Caicara-Ciudad Bolívar road, 3 km east of Maripa, Bolivar, 7°25’N 69°9’30”W, 19 Nov 1985, A. Machado. ANSP 159176 (34, 16.5-33.5 mm SL, 1 c&s 26.3 mm SL), caño crossing road to Las Trincheras, 2.7 km South of intersection Ciudad Bolivar-Caicara road, Bolivar, 4°39’N 72°39’W, 20 Nov 1985, W. Saul *et al.* ANSP 159182 (51, 15.2-35.3 mm SL), stream 21 km Southeast of El Burro at Caiacara-Puerto Ayacucho road, Amazonas, 26 Nov 1985, W. Saul *et al.* ANSP 159184 (100, 14.0-27.4 mm SL), caño and swamp 19 km Southwest of El Burro on Caiacara-Puerto Ayacucho road, Amazonas, Nov 1985, W. Saul *et al.* ANSP 159186 (1, 34.5 mm SL), caño 15.1 kmEast of ferry crossing on Caicara-Puerto Ayacucho highway, Bolivar, 28 Nov 1985, B. Chernoff *et al.* ANSP 159188 (12, 16.1-31.9 mm SL), swamp 22.1 km East of rio Parguaza, ferry crossing on Caicara-Puerto Ayacucho highway, Amazonas, 28 Nov 1985, B. Chernoff *et al.* ANSP 159192 (1, 19.9 mm SL), caño crossing dirt road near entrance to Hacienda Fundo Malama (Sr. Biales), about 15 km North Sipao, Bolivar, 7°35’N 65°22’W, 19 Nov 1985, W. Saul & R. Royero. ANSP 159193 (12, 18.5-22.4 mm SL), caño at concrete bridge near Hacienda Fundo Malama (Sr. Biales), about 11 km North of Sipao, Puerto Ayacucho, Amazonas, 6°8’N 67°22’W, 19 Nov 1985, W. Saul & R. Rovero. ANSP 165661 (5, 18.4-22.0 mm SL), caño Horeda, at border of Bolivar-Amazonas Terr., about 68 km Northeast of Puerto Ayacucho, on Puerto Ayacucho-Puerto Paez highway, Bolivar, 7°33’N 65°23’W, 10 Nov 1989, S. Schaefer *et al.* ANSP 165688 (5, 16.8-22.8 mm SL), caño Potrerito, affluent of rio Cinaruco, 24 km South of rio Cinaruco on San Fernando de Apure-Puerto Paez highway, Apure, 6°25’N 67°32’W, 11 Nov 1989, S. Schaefer *et al.* CAS 69237 (21, 22.6-40.3 mm SL), caño trybutary of rio Guarico at Calabozo, Guarico, 11 Feb 1938, F. Bond. CAS 69312 (6, 23.4-31.0 mm SL), rio Guaire at Caracas, Federal District, 22 Aug 1949, F. Bond. FMNH 85597 (4, 27.0-39.7 mm SL), pequeno park 800 m of Porto Ayacucho-San Mariapo road, Amazonas, 13 Jan 1975, J. Thomerson *et al.* FMNH 103819 (20 of 170, 19.9-31.0 mm SL), caño Provencial, about 10 km North of Puerto Ayacucho, Amazonas, 21 Jan 1991, B. Chernoff *et al.* MBUCV 328 (5, 28.3-35.3 mm SL), headwaters of Morichal de Yagrumito, South of Palenque, rio Orituco, rio Apure drainage, Guárico, 7 May 1960, F. Webezahn & G.Bergold. MBUCV 5208 (12, 20.4-26.8 mm SL), caño El Limón, Temblador-San Felix road (at Barrancas de Fajardo), Monagas, 3 May 1968, R. Ampueda & L. Duque. MBUCV 7270 (12, 19.9-27.1 mm SL), caño located at 20 km of San Fernando de Atabapo-Santa Barbara road, Amazonas, 13 Apr 1972, A. Cortéz. MBUCV 12536 (8, not measured), caño Carinagua, next pisciculture of M.A.C., rio Cataniapo, Amazonas, 5°38’11”N 67°35’33”W, 28 Feb 1981, G. Pereira. MBUCV 12563 (3, 23.3-33.3 mm SL), small stream crossing Puerto Ayacucho-Caicara road at 97 km of Puerto Ayacucho, Amazonas, 1 Mar 1981, G. Pereira. MBUCV 12585 (1, 19.4 mm SL), small stream crossing Puerto Ayacucho-Caicara Road, about 172 km of Puerto Ayacucho, Bolivar, 2 Mar 1981, G. Pereira. MBUCV 12736 (1, 37.8 mm SL), caño Las Pavas, affluent of rio Cataniapo, about 3 km of Puerto Ayacucho-Gavilan road, Amazonas, 5°34’42”N 67°32’16”W, 29 Jul 1981, R. Royero *et al.* MBUCV 12805 (9, 26.1-45.0 mm SL), caño Las Pavas, affluent of rio Cataniapo, about 3 km of Puerto Ayacucho-Gavilan road, Amazonas, 5°34’42”N 67°32’16”W, 29 Jul 1981, R. Royero *et al.* MBUCV 14069 (4, 19.9-28.8 mm SL), small stream affluent of rio Cataniapo, 12.5 km of Puerto Ayacucho-Gavilán road, Amazonas, 5°34’N 67°30’36”W, 6 May 1982, R. Royero *et al.* MBUCV 14337 (1, 32.9 mm SL), caño upstream Sarama Sota, upper rio Cataniapo, Amazonas, 5°38’N 67°7’40”W, 20 Aug 1984, R. Royero *et al.* MBUCV 14434 (35, 18.3-28.6 mm SL), caño Ucuoto, about 5 km of mouth, rio Cataniapo, Amazonas, 25 Dec 1983, R. Royero & L. López. MBUCV 14464 (2, 17.7 and 19.7 mm SL), mouth of caño Colorado, rio Cataniapo, Amazonas, 5°34’32”N 67°13’46”W, 23 Dec 1983, R. Royero & L. López. MBUCV 17478 (3, 29.9-39.6 mm SL), Pozo de Lucas, about 7 km South of San Fernando de Atabapo, Amazonas, 8 Apr 1982, R. Royero & G. Pereira. MBUCV 17725 (6, 15.7-42.1 mm SL), floodplain at 22.1 km east of ferry cross at Caicara-Puerto Ayacucho road, rio Parguaza, Bolívar, 28 Nov 1985, A. Machado & D. Ibarrara. MBUCV 17741 (2, 21.0 and 21.3 mm SL), caño Rodeo, 100 km east ofgas station of Maripa at Ciudad Bolívar-Caicara road, rio Caura, Bolívar, 21 Nov 1985, J. Lundberg, B. Saúl & R. Royero. MBUCV 17916 (5, 21.3-35.1 mm SL), caño crossing road to Las Trincheras, 2.7 km South Ciudad Bolívar-Caicara road, rio Caura, Bolívar, 7°22’N 64°56’W, 20 Nov 1985, J. Lundberg, B. Saúl & R. Royero. MBUCV 18249 (19, 13.0-22.8 mm SL), stream and floodplain at 19 km Southeast El Burro, Caicara-Puerto Ayacucho road, Amazonas, 26 Nov 1985, A. Machado *et al.* MBUCV 19717 (9, 19.9-41.2 mm SL), caño North of base camp, rio Mavaca, Amazonas, 20 Mar 1989, C. Ferraris *et al.* MBUCV 24402 (1, 25.3 mm SL), small stream at rigth bank (upstream) about 600 m upstream caño Gavilán mouth, rio Cataniapo, Amazonas, 5°32’13”N 67°23’18”W, 20 Dec 1984, R. Royero. MBUCV 24723 (1, 28.2 mm SL), caño La Piedrita at bridge, Temblador-Barrancas road, Monagas, 14 Feb 1978, Aguana & Padilla. MBUCV 27485 (34, 15.8-26.4 mm SL), rio Moquete, Morichal near Paso Bajito, rio Caris, Anzoátegui, 3 Oct 1984, A. Machado *et al.* MBUCV 27621 (1, 24.7 mm SL), caño Mapirito, rio Guanipa, Monagas, 19 Jan 1994, G. Pereira. MBUCV 28126 (3, 25.5-31.5 mm SL), rio Moquete, Morichal near Paso Bajito, rio Caris, Anzoátegui, 3 Dec 1984, A. Machado *et al.* MBUCV 28167 (6, 18.1-26.7 mm SL), Morichal, affluent of rio Moquete, near Paso Bajito, El Corozo road, Anzoátegui, 14 Feb 1984, A. Machado *et al.* MBUCV 28269 (3, 22.0-27.8 mm SL), rio Moquete, Morichal, near Paso Bajito, Anzoátegui, 2 Feb 1984, A. Machado *et al.* MBUCV 28341 (18 de 19, 20,1-25,9 mm SL), rio Moquete, Morichal, near Paso Bajito, Anzoátegui, 2 Feb 1984, A. Machado *et al.* MBUCV 28350 (44, 16.6-29.5 mm SL), Morichal affluent of rio Moquete, near Paso Bajito, rio Caris, Anzoátegui, 2 Feb 1984, A. Machado *et al.* MBUCV 28358 (54, 13.6-34.2 mm SL), Morichal, affluent of rio Moquete, about 10 km West of Paso Bajito, El Corozo road, rio Moquete, rio Caris, Anzoátegui, 2 Feb 1984, A. Machado *et al.* MBUCV 28428 (40, 14.2-21.3 mm SL), rio Caris, paso Los Cocos at tributary stream, Anzoátegui, 1 Feb 1984, A. Machado *et al.* MBUCV 28451 (10, 17.1-26.1 mm SL), fundo La Esperanza, rio Caris, Anzoátegui, 3 Oct 1984, A. Machado *et al.* MBUCV 28466 (21, 14.2-26.1 mm SL), paso Coera, rio Caris, Anzoátegui, 1 Feb 1984, A. Machado *et al.* MBUCV 28596 (1, 24.4 mm SL), paso Los Cocos, rio Caris, about 40 km South of El Tigre, Anzoátegui, 1 Feb 1984, A. Machado *et al.* MBUCV 28663 (27, 15.7-35.6 mm SL), rio Atapirire, bridge between Campo Aricuna and El Tigre road, Anzoátegui, 4 Oct 1984, A. Machado. MBUCV 29201 (5, 29.2-32.0 mm SL), rio Corocoro, 40 min upstream from Yutaje, Amazonas, 27 Apr 1999, S. Schaefer *et al.* MBUCV 29318 (3, 26.3-30.9 mm SL), rio Cuao, pools upstream raudal del Danto, rio Sipapo drainage, Amazonas, 5°12’39”N 67°33’35”W, 30 Apr 1999, S. Schaefer *et al.* MBUCV 29661 (2, 27.8 and 34.5 mm SL), caño Yurage, affluent of rio Cataniapo, between Merey community and Paso Las Pavas, Amazonas, 5°32’19”N 67°24’36”W, 15 Aug 2000, F. Provenzano *et al.* MBUCV 29696 (4, 10.8-30.1 mm SL), caño Peramán, affluent of rio Cataniapo at San Pedro community, Amazonas, 14 Oct 2000, J. Fernández & L. Marcano. MBUCV 29742 (1, 38.9 mm SL), caño Danto, affluent of rio Cataniapo, Amazonas, 16 Oct 2000, J. Fernández & L. Marcano. MBUCV 29759 (4, 11.2-23.3 mm SL), caño Las Pavas, affluent of rio Cataniapo, Amazonas, 16 Oct 2000, J. Fernández & L. Marcano. MBUCV 29767 (1, 37.2 mm SL), cañito upstream caño Gávilan, affluent of rio Cataniapo, Amazonas, 17 Oct 2000, J. Fernández & L. Marcano. MBUCV 30752 (15, 11.2-15.5 mm SL), caño Gallineta, affluent of rio Cataniapo, Amazonas, 30 Jan 2001, J. Fernández *et al.* MBUCV 30776 (4, 19.0-31.3 mm SL), caño Danto, affluent of rio Cataniapo, Amazonas, 31 Jan 2001, J. Fernández *et al.* MBUCV 31346 (24, 18.9-26.6 mm SL), rio Aro, Morichal at Road East of Esmeralda, Bolívar, 7°34’7”N 64°15’45”W, 25 Mar 2002, F. Provenzano & A. Rojas. MBUCV 31767 (8, 26.4-31.8 mm SL), caño Danto, affluent of rio Cataniapo, Amazonas, 23 Jan 2002, J. Fernández *et al.* MBUCV 33134 (58, 16.7-34.9 mm SL), Morichal Castillito, affluent of rio San Jose, Anzoátegui, 8°39’20.4”N 64°40’2.79”W, 28 May 2006, A. Marcano *et al.* MBUCV 33159 (22, 13.7-35.7 mm SL), Morichal Rabanal, Anzoátegui, 8°36'29.38"N 64°39'11.51W, 28 May 2006, A. Marcano *et al.* MBUCV 33335 (8, 19.4-35.0 mm SL), Morichal Cabeza de Coporo South of Santa Clara through Puerto Ayacucho, Anzoátegui, 8°27’12.54”N 64°38’35.80”W 30 May 2006, A. Marcano *et al.* MBUCV 33345 (16, 21.9-34.8 mm SL), quebrada San Antonio, rio Pao drainage, Anzoátegui, 30 May 2006, A. Marcano *et al.* MBUCV 33582 (19, 21.8-32.5 mm SL), Morichal Castillito, affluent of rio San Jose, rio Pao, Anzoátegui, 8°39’20.4”N 64°40’2.79”W, 2 Sep 2006, A. Marcano *et al.* MBUCV 33587 (40, 18.8-37.3 mm SL), Morichal Rabanal, rio Pao, Anzoátegui, 8°36'29.38"N 64°39'11.51W, 2 Sep 2006, A. Marcano *et al.* MBUCV 33591 (89, 11.6-35.5 mm SL), Morichal Cabeza de Coporo, Anzoátegui, 8°27’12.54”N 64°38’35.80”W, 3 Sep 2006, A. Marcano *et al.* MBUCV 33617 (3, 23.1-34.4 mm SL), quebrada San Antonio, South of Santa Clara, Anzoátegui, 8°27’12.54”N 64°38’35.80”W, 3 Sep 2006, A. Marcano *et al.* MBUCV 34191 (12, 15.0-29.4 mm SL), Morichal Veladero, under bridge at Zuata-Sitio Turístico Bayona road, near deviation to Fundo Trujillo, Anzoátegui, 7°59’45.79”N 65°8’21.37”W, 19 May 2007, A. Herrera *et al.* MBUCV 34249 (17, not measured), Morichal Veladero, behind camping of Sitio Turístico Bayona, Anzoátegui, 7°56’1.72”N 65°9’59.63”W, 20 May 2007, A. Herrera *et al.* MBUCV 34347 (65, 13.7-31.2 mm SL), Morichal Quebrada El Cebruno, rio San Bartolo, Anzoátegui, 7°56’38”N 64°59’38.63”W, 20 May 2007, A. Herrera *et al.* MBUCV 34359 (not measured), Morichal Cachicamo, after Hato Cachicamo, rio Maripe, Anzoátegui, 8°1’21.5”N 64°59’10.3”W, 20 May 2007, A. Herrera *et al.* MBUCV 34414 (1, 26.4 mm SL), Morichal Pesquero, at Finca El Saco, Anzoátegui, 8° 7’20.2”N 65°17’9.9”, 22 May 2007, A. Herrera *et al.* MBUCV 34576 (15, not measured), Morichal Agua Blanca, Anzoátegui, 8°7’0.7”N 65°23’36.10”W, 23 May 2007, A. Herrera *et al.* MBUCV 34648 (9, 18.9-24.9 mm SL), Morichal del Diablo, isolated lagoon at floodplain, Guárico, 8°20’7.9”N 65°34’29.0”W, 24 May 2007, A. Herrera *et al.* MBUCV 34654 (927, not measured), Morichal Guacharaca, isolated lagoon, morichal branch, Guárico, 8°8’56.34”N 65°32’44.08”W, 25 May 2007, A. Herrera *et al.* MBUCV 34756 (3, 15.3-22.9 mm SL), Morichal El Playazo, rio Zuata, Guárico, 8°32’39.20”N 65°34’5.43”W, 26 May 2007, A. Herrera *et al.* MBUCV 34838 (1, 14.0 mm SL), rio El Muerto and tributary at road to Santa María de Ipire, rio Zuata, Guárico, 0°51’59.6”N 65°20’10.3”W, 12 Aug 2007, F. Provenzano *et al.* MBUCV 34865 (5, not measured), Morichal rio Mapire, at canal with isolated pool, Anzoátegui, 8°15’20.63”N 64°56’43.03”W, 13 Aug 2007, F. Provenzano *et al.* MBUCV 34884 (303, not measured), Morichal El Merey, rio Mapire, Anzoátegui, 8°15’45.65”N 64°55’17.29”W, 13 Aug 2007, F. Provenzano *et al.* MBUCV 34888 (49, 15.5-34.4 mm SL), Morichal Casupo, balneário El Encanto, rio Mapire, Anzoátegui, 8°10’7.82”N 64°49'41.08”W, 13 Aug 2007, F. Provenzano *et al.* MBUCV 34998 (216, 13.4-37.4 mm SL), Morichal El Zamuro, rio Ature, Anzoátegui, 8°38’23.89”N 64°48’26.52”W, 7 Oct 2007, A. Herrera *et al.* MBUCV 35049 (8, 13.7-21.9 mm SL), Lavadero de Granza, affluent of rio San Pedro, perto de Mapire, Anzoátegui, 7°44’17.11”N 64°43’39.60”W, 8 Oct 2007, A. Herrera *et al.* MBUCV 35413 (50, 19.4-35.0 mm SL), Morichal Palenque, headwaters of rio Aguaro, North of Santa Rita, rio Guariquito, Guárico, 8°23’34.58”N 66°19’13.01”W, 24 Sep 2008, A. Marcano *et al.* MBUCV 35427 (14, 15.3-22.2 mm SL), Morichal Mariota, affluent of rio Aguaro, North of Santa Rita, rio Guariquito, Guárico, 8°17’0.63”N 66°19’38.94”W, 24 Sep 2008, A. Marcano *et al.* MBUCV 35444 (17, 14.5-30.8 mm SL), Morichal El Perro, affluent of rio Manapire, North of Santa Rita, rio Guariquito, Guárico, 8°16’53.49”N 66°17’36.60”W, 24 Sep 2008, A. Marcano *et al.* MBUCV 35467 (27, 16.7-31.1 mm SL), Morichal Chucuto, affluent of rio Manapire, North of Santa Rita, rio Guariquito, Guárico, 8°36’16.16”N 66°15’16.45”W, 24 Sep 2008, A. Marcano *et al.* MBUCV 35541 (1, 23.1 mm SL), Morichal El Perro, affluent of rio Manapire, North of Santa Rita, rio Guariquito, Guárico, 8°16’52.96”N 66°17’35.49”W, 25 Sep 2008, A. Marcano *et al.* MCNG 1757 (1, 24.6 mm SL), caño Macaurel at Hato Santiago, Apure, Guarico, 8°31’20”N 67°2’W, 28 Oct 1980, DTC80-118. MCNG 2245 (6, 15.1-18.5 mm SL), Hato El Encanto, rio Riecito, Apure, 30 Nov 1980, SR80-8. MCNG 2653 (2, 26.1 and 25.4 mm SL), caño Yatuje at the end of road, Amazonas, 5°32’50”N 66°6’57”W, 15 Mar 1981, SR81-20. MCNG 2653 (2, 26.1 and 25.4 mm SL), caño Yatuje at the end of road, Amazonas, 5°32’50”N 66°6’57”W, 15 Mar 1981, SR81-20. MCNG 3015 (1, 18.8 mm SL), rio Manapiare, near Yutaje, Amazonas, 5°35’N 66°17’50”W, 16 Mar 1981, SR81-23. MCNG 3321 (1, 21.9 mm SL), Hato San Jose del Aguaro near stream opposite tohouse, rio Aguaro, Guarico, 7°57’30”N 66°29’W, 29 Sep 1981, SR81-62. MCNG 6820 (1, 16.3 mm SL), caño La Pica, near rio Cinaruco, Apure, 6°48’N 67°49’20”W, 16 Jan 1982, DCT82-25. MCNG 11390 (6, 18.7-23.2 mm SL), affluent of rio Agua Blanca, Parque Aguaro-Guaritico about 39 km Southeast of Pelenque, rio Apure, Guárico, 8°43’30”N 66°53’50”W, 13 Apr 1984, DCT84-49. MCNG 11770 (6, 14.5-21.2 mm SL), caño affluent of rio Aguaro, Hato Los Pavones at 12 km West of Santa Rita, rio Apure, Guarico, 8°7’50”N 66°22’30”W, 22 Sep 1981, DCT81-125. MCNG 13216 (35, 17.9-36.4 mm SL), small stream east of San José, caño Agua Blanca, rio Apure, Guarico, 8°34’N 66°49’20”W, 14 Apr 1984, DCT84-51. MCNG 6685 (13.0, mm SL), caño Caripito at El Palomo at 150 km Southwest of Caicara del Bolivar, 6°33’N 67°0’30”W, 30 Mar 1982, DCT82-37. MCNG 14806 (7, 15.4-18.9 mm SL), rio San José, East of Road out of Palenque, 39 km Southeast of Palenque, rio Guariquito, Guárico, 8°40’N 66°54’W, 22 Jan 1986, DCT86-13. MCNG 14831 (1, 16.6 mm SL), Morichal near 17 km South of DCT 86-13, 6 km east way of grazón, rio San José, rio Guariquito drainage, Guárico, 8°34’40”N 66°52’25”W, 22 Jan 1986, DCT86-14. MCNG 14884 (1, 19.9 mm SL), rio San José, West side of Aguaro-Guariquito, 57 km South of Palenque, rio Guariquito, Guárico, 8°29’N 66°53’10”W, 22 Jan 1986, DCT86-15. MCNG 15187 (1, 22.1 mm SL), caño 29 km east of rio Cuchivero, road 19 at bridge (21 km east of Santa Rosalia), rio Suapire, Bolivar, 7°21’N 65°53’W, 25 Jan 1986, DCT86-25. MCNG 15223 (1, 17.2 mm SL), rio Urbani, affluent of rio Caura, 16 km South of road 19, on the way of las Tricheras, rio Caura, Bolivar, 7°19’N 64°58’W, 25 Jan 1986, DCT86-27. MCNG 15235 (1, not measured), caño El Rodeo, affluent of rio Pao, road 19, near El Vaquiro, Bolivar, 7°20’N 64°35’W, 25 Jan 1986, DCT86-28. MCNG 16288 (10, 21.5-35.8 mm SL), Morichal Tauca, Road to Caicara, about 20 km of Sipao, affluent of rio Tucuragua, Bolivar, 7°23’N 65°35’W, 20 May 1985, WC85-3. MCNG 16406 (2, 25.9 and 28.3 mm SL), Caicara del Orinoco-El Tigre road, about 1 km, left and rigth side of the road, Anzoateguí, 7°31’N 66°1’W, 24 Aug 1986, EC86-4. MCNG 16170 (1, 29.9-30.8 mm SL), rio Morichal Largo, at bridge near El Salto village, Monagas, 8°56’N 63°7’W, 17 Aug 1984, LN84-17. MCNG 17149 (9, 18.2-24.1 mm SL), caño Macanilla, upstream R. Chaviripa through Puerto Ayacucho, Bolivar, 7°12’N 66°22’W, 19 Dec 1986, EC86-6. MCNG 17442 (1, 17.1 mm SL), balneário Pozo Azul, at road El Burro-Puerto Ayacucho, Amazonas, 5°50’N 67°29’W, 20 Dec 1986, EC86-7. MCNG 17455 (108, 18.5-34.5 mm SL), Morichal at Puerto Ayacucho-Sabaneta road, Amazonas, 5°25’N 67°45’W, 23 Dec 1986, EC86-9. MCNG 17464 (4, 16.4-19.4 mm SL), river between Puerto Ayacucho and Galipero, Amazonas, 5°37’N 67°36’W, 25 Dec 1986, EC86-12. MCNG 17699 (3, 27.3-27.9 mm SL), caño about 31 km South of way to Puerto Ayacucho, Bolivar, 6°13’N 67°10’30”W, 18 Apr 1984, DCT84-63. MCNG 17736 (1, 26.1 mm SL), caño Chivaripa between Puerto Paez and Caicara, 79 km Southest Road to Puerto Ayacucho, Bolivar, 6°28’30”N 67°2’30”W, 18 Apr 1984, DCT84-65. MCNG 17743 (13, 18.9-30.2 mm SL), caño Agua Mena about 20 km Southeast Road to Puerto Ayacucho, Bolivar, 6°14’30”N 67°16’W, 18 Apr 1984, DCT84-62. MCNG 20099 (2, 18.3 and 24.4 mm SL), caño La Pica 5 km East of new embanckment, Capanaparo, Apure, 6°55’N 67°25’W, 1 May 2011, ABD89-21. MCNG 20263 (12, 13.7-18.9 mm SL), Laguna Brava at savanna, Finca Catalino, Capanaparo, Apure, 6°57’N 67°39’W, 28 Apr 1989, DCT89-127. MCNG 21144 (8, 17.9-27.9 mm SL), caño Urbana at bridge, 29 km North of Jabillal, rio Caura, Bolivar, 7°0’N 64°45’W, 4 Mar 2011, DCT89-59. MCNG 21643 (2, not measured), rio Guayapo at 149 km of confluence with rio Sipapo, Amazonas, 4°23’N 67°2’W, 20 May 1980, LN89-47. MCNG 21662 (3, 28.4-35.3 mm SL), cañito at 150 m Northwest of camping CVG-TECMIN at 149 km of confluence with rios Guayapo and Sipapo, Amazonas, 4°23’N 67°2’W, 22 May 1989, LN89-49. MCNG 22967 (1, 26.2 mm SL), caño Blanco affluent of rio Asisa, near Asisa community (Frente 02-Pica 2 of TECMIN), rio Ventuari, Amazonas, 4°30’N 65°46’30”W, 7 Oct 2011, LN89-121. MCNG 23361 (9, 15.5-33.6 mm SL), caño Bocachica North of airport of Maroa, via Yavita, rio Casiquiare, Amazonas, 2°47’N 67°40’W, 9 Aug 2011, DCT89-133. MCNG 23632 (22, 18.2-30.5 mm SL), caño Pozo Azul at balneário Pozo Azul, Puerto Ayacucho, Amazonas, 5°50’N 67°29’W, 13 May 1989, LN89-36. MCNG 23706 (3, 21.6-36.3 mm SL), Arekuna, Campam, EDELCA at Morichal, rio Caroni, Bolivar, 6°30’N 62°53’W, 3 Jul 1990, DCT90-5. MCNG 23897 (2, 15.5 and 16.2 mm SL), small pools at wood at 500m of rio Ventuari, Amazonas, 4°16’N 66°23’W, 28 Sep 1989, LN89-106. MCNG 23901 (13, 12.7-24.8 mm SL), small isolated and interconected pools at morichal at open savanah (CVG-Tecmin Frente 6 pica 2), West of rio Guapuchí, rio Ventuari, Amazonas, 4°14’N 66°44’W, 23 Sep 1989, LN89-96. MCNG 23908 (10, not measured), rio Morichal Largo, at bridge near El Salto village, Monagas, 8°56’N 63°7’W, 6 Nov 1989, LN84-17. MCNG 25668 (3, 19.9-22.1 mm SL), small stream at left side of rio Mavaca, Amazonas, 1°55’N 65°6’W, 3 Feb 1991, LN91-23. MCNG 26114 (4, not measured), small stream affluent of rio Siapa, 1°26’N 65°43’W, 20 Apr 1991, ABD91-28. MCNG 26419 (3, 13.8-16.3 mm SL), rio Cataniapo, near Las Pavas community, Amazonas, 5°32’25”N 67°29’30”W, 3 Sep 1991, OLM91-3. MCNG 26566 (3, not measured), caño San Miguel, West Galeras de Cinaruco, Apure, 6°34’24”N 67°17’32”W, 20 Jan 1992, LN92-7. MCNG 26596 (7, 11.9-22.7 mm SL), caño Pozo Azul at balneário, Amazonas, 5°45’49”N 67°29’21”W, 21 Jan 1992, LN92-8. MCNG 26606 (2, 18.6 and 23.4 mm SL), caño Agua Linda near Caicara-Puerto Ayacucho road, Bolivar, 5°50’24”N 67°27’10”W, 22 Jan 1992, LN92-9. MCNG 26874 (34, 15.0-38.1 mm SL), caño at jungle of rio Mavaca, Amazonas, 2°28’N 65°6’W, 25 Jan 1991, LN91-12. MCNG 26893 (1, 26.2 mm SL), Laguna West of Cinaruco, rio Apure, Apure, 6°33’6”N 67°30’43”W, 23 Jan 1992, LN92-12. MCNG 26993 (8, 24.2-30.8 mm SL), caño Guayabal, 44 km South of Puerto Ayacucho, road to Samariapo, Amazonas, 5°20’16”N 67°42’29”W, 20 Mar 1991, ABD91-12. MCNG 27006 (19, 15.7-30.7 mm SL), caño at 61 km east of Puerto Ayacucho-Caicara, rio Cataniapo, Amazonas, 5°35’47”N 67°12’38”W, 19 Mar 1991, ABD91-6. MCNG 27051 (9, 18.0-30.3 mm SL), east side of Isla Ratón, floodplain near Sabanita, Amazonas, 5°5’N 67°48’W, 14 Aug 1992, DCT92-8. MCNG 27144 (54, 16.1-29.4 mm SL), caño Morichal, near 1 km North of Esmeralda and rio Mavaca, Amazonas, 3°10’30”N 65°33’W, 22 Jan 1991, LN91-5. MCNG 26975 (1, 27.4 mm SL), caño Kaprina, 21 km West of rio Parguaza, Puerto Ayacucho-Caicara road, rio Cataniapo, Amazonas, 19 Mar 1991, ABD91-5. MCNG 27218 (8, 21.7-30.7 mm SL), isolated lagoon at meander of rio Mavaca, Amazonas, 2°22’30”N 65°5’30”W, 31 Jan 1991, LN91-19. MCNG 28769 (7, 27.5-35.9 mm SL), caño La Rueda, 24 km South of Puerto Ayacucho at road to Samariapo, Amazonas, ABD91-7. MCNG 30392 (14, 16.0-35.4 mm SL), caño about 20 min walking South of Macuruco, caño El Loro, Amazonas, 21 Aug 1994, ABD94-1. MCNG 30402 (7, 18.2-32.8 mm SL), Tobogan de la Selva about 3 km of La Coromoto community, Puerto Ayacucho-Samariapo road, Amazonas, 5°23’45”N 67°36’30”W, 29 Aug 1994, ABD94-7. MCNG 31506 (6, 14.8-22.2 mm SL), Parque Nacional Aguaro-Guariquito, Morichal Charcote (opened morichal), rio San Bartolo, rio Guariquito, Guarico, 8°24’16”N 66°34’23”W, 5 Jan 1995, DCT95-4. MCNG 31867 (10, 17.7-23.4 mm SL), Parque Nacional Aguaro-Guariquito, Morichal Simon (way to La Esperanza from El Mejo), tributary of rio San Bartolo, Guarico, 8°30’45”N 66°35’49”W, 9 Jan 1995, DCT95-12. MCNG 28781 (21, 23.2-32.9 mm SL), caño 28 km South of Puerto Ayacucho, road to Samariapo, Amazonas, ABD91-8. MCNG 30378 (1, 20.7 mm SL), base of Yapacana hill, near caño Cotua, Amazonas, 22 Aug 1994, ABD94-3. MCNG 33128 (21, 14.1-40.8 mm SL), rio Autana, Raudal Pereza, Amazonas, 24 Aug 1996, RNR92-1. MCNG 34545 (3, 25.2-30.0 mm SL), Galeras del Cinaruco, rio Apure, Apure, 6°34’N 67°15’W, 16 Jan 1996, DRO96-1. MCNG 36468 (32, 31.0-45.3 mm SL), quebrada entering morichal El Pozo, through CVG de caucho camping, Amazonas, 13 Jul 1994, DCT94-36. MCNG 36472 (1 of 5, 18.0 mm SL), caño Ucata, near Ucata community, Amazonas, 12 Jul 1994, DCT94-33. MCNG 36479 (7, 17.8-31.9 mm SL), Balneario Pozo Azul, Bolivar, 18 Aug 1992, PP92-1. MCNG 39995 (5, 15.6-21.8 mm SL), Laguna Oheros, rio Cinaruco, rio Apure, 6°32’48”N 67°25’54”W, 16 Feb 1999, DAA99-82. MCNG 40540 (3, not measured), rio Cinaruco, Espinar lagoon, Apure, 6°33’22”N 67°22’33”W, 17 Mar 1999, DAA99-127. MCNG 41789 (1, 23.3 mm SL), rio Matiyure at Mantecal and La Trinidad-Rincón Hondo road, rio Apure, Apure, 7°26’54”N 69°6’47”W, 4 Apr NL17-99. MCNG 42403 (29, 16.7-27.0 mm SL), small stream at 6 km South of rio Cinaruco, between El Burro and San Fernando de Apure, Apure, 6°31’22”N 67°33’4”W, 21 Jan 1999, LN99-37. MCNG 43444 (8, 25.6-36.3 mm SL), Pozo Azul near rio Cinaruco, Apure, 16 Mar 1999, DAA99-417. MCNG 43929 (3, 25.6-30.6 mm SL), caño Caicara at bridge near modules of UNELLEZ, Apure, 3 Jul 1999, OLM99-20. MCNG 45599 (29, 13.8-30.4 mm SL), rio Orinoco near Campamento Manaka, about 150 m of mouth of caño Carmen, Amazonas, 3°48’37”N 67°1’23”W, 10 Feb 2002, CGM02-04. MCNG 45979 (4, 17.9-23.1 mm SL), Maniapure, Bolivar, 15 Feb 2001, FA2001-01. MCNG 45984 (1, 19.7 mm SL), Maniapure, Bolivar, 15 Feb 2001, FA2001-02. MCNG 47333 (7, 20.1-23.4 mm SL), rio Ventuari at porto f Cucurital camping, Amazonas, 4°7’3”N 66°40’47”W, 26 Jun 2002, CGM02-54. MCNG 47342 (33, 15.8-41.6 mm SL), caño Carinagua near “Alto Carinagua” community, Amazonas, 5°40’42”N 67°32’7”W, 9 Oct 2002, CGM02-59. MCNG 48508 (10, 18.7-30.3 mm SL), caño at Tobogan de la selva, Amazonas, 16 Jun 1999, MFA99-1. MCNG 48711 (10, 13.0-22.3 mm SL), caño La Pica, Apure, 17 Feb 2001, FA2001-7. MCNG 49818 (47, not measured), caño Yakiguapo at rio Ventuari, Amazonas, 4°4’56”N 66°54’18”W, 6 Dec 2002, CGM02-74. MCNG 49905 (2, 23.1 and 23.9 mm SL), caño Moriche, 116 km North of Macuruco, 169 km Northwest of San Fernando de Atabapo, rio Ventuari, Amazonas, 4°45’5”N 66°21’32”W, 7 Apr 2004, VEN04-14. MCNG 50531 (2 of 4, 17.5-18.0 mm SL), Laguna de Macuruco, Amazonas, 7 Dec 2003, OLM03-22. MCNG 50573 (8, 19.2-29.5 mm SL), modules of UNELLEZ at gates, Apure, 30 Jun 2002, MCRA02-630. MCNG 51140 (7, 19.4-35.4 mm SL), at Tobogancito, about 1 km upstream Tobogan de la Selva, Amazonas, 4 Jun 2003, OLM03-2. MCNG 51147 (2, 20.2 and 32.5 mm SL), at Tobogancito, about 1 km of balneário of Tobogan de la Selva, Amazonas, 4 Jun 2003, OLM03-3a. MCNG 52513 (11, 18.4-24.8 mm SL), caño La Guardia at moriches near Isla Moriches, rio Capanaparo, Apure, 6°42’59”N 67°30’39”W, 16 Mar 2005, CGM05-39. MCNG 53133 (15, 15.6-18.7 mm SL), caño La Guardia, Apure, 6°50’40”N 67°19’50”W, 26 Feb 2005, CGM05-53. MCNG 53579 (36, 16.1-20.5 mm SL), caño La Guardia near Las Boconas lagoon, rio Capanaparo, Apure, 6°42’59”N 67°30’39”W, 10 Mar 2005, cgm05-44. MCNG 53617 (2, 14.6 and 15.9 mm SL), rio Pasiba at lagoon Pasibita, Amazonas, 16 Jan 2005, SCW05-24. MCNG 53953 (4, 28.6-49.7 mm SL), stream 30 km South of Puerto Ayacucho, Amazonas, 1 Apr 2004, VEN04-2. MCNG 54038 (49, 17.7-33.9 mm SL), Laguna La Rompía, rio Apure, Apure, 8 Jun 1982, OCVI82-5. MCNG 54039 (46, 11.4-37.1 mm SL), caño at 18 km from deviation of El Burro and Puerto Ayacucho, at Caicara road, Bolivar, 31 Mar 1983, OCIII83-9. MCNG 54040 (7, 31.7 mm SL), rio Apure 200 m of Ma Nieves bridge, Apure, 8 Jun 1982, OCVI82-4. MCNG 54359 (2, not measured), caño El Carmen, Amazonas, 2003, OR2.3. MHNG 2200.028 (10 of 26, 14.9-45.11 mm SL), stream affluent of rio Carrao, 5 km East of Canaima, Bolivar, 10 Feb 1968, E. Hoigné. MHNLS 912 (9, 25.3-34.6 mm SL), rio Temi, Guainia, Amazonas, 2°55’8”N 55°8’67”W, 30 Nov 1973, Arleo. MHNLS 2234 (4, 19.0-25.3 mm SL), rio Águaro, Guarico, 4 Apr 1974, Hoogesteijn, Reul & Rafael. MHNLS 5338 (7 of 8, 25.9-35.7 mm SL), caño Caripo, Pijiguaos, Bolívar, 4 Feb 1988, C. Lasso, G. Colonnello & C. Gauveca. MHNLS 5339 (1, 24.6 mm SL), small pool at quebrada La Tigra, Pijiguaos, Bolívar, 24 Apr 1988, C. Lasso, G. Colonnello& C. Gauveca. MHNLS 5340 (6, 22.6-44.6 mm SL), small pool at quebrada La Tigra, Pijiguaos, Bolívar, 27 Apr 1988, C. Lasso, G. Colonnello & C. Gauveca. MHNLS 5341 (1, 32.1 mm SL), caño Chorro de Água, las Bateas, Pijiguaos, Bolívar, 27 Apr 1988, C. Lasso, G. Colonnello & C. Gauveca. MHNLS 5342 (9, 21.4-35.6 mm SL), La Solanera, Pijiguaos, Bolívar, 26 Apr 1988, C. Lasso, G. Colonnello & C. Gauveca. MHNLS 5343 (3, 19.5-23.8 mm SL), quebrada Trapichote, crossing through El Jobal, Bolívar, 28 Apr 1988, C. Lasso, G. Colonnello & C. Gauveca. MHNLS 5577 (1, 27.3 mm SL), rio Suapure, crossing through El Jobal, Bolívar, 28 Apr 1988, C. Lasso, G. Colonnello & C. Gauveca. MHNLS 5736 (3, 22.7-31.6 mm SL), poço Suapi, mouth at rio Suapure, Pijiguaos, Bolívar, 29 Apr 1988, C. Lasso, G. Colonnello & C. Gauveca. MHNLS 9268 (17, 19.8-36.9 mm SL), rio Guarapiche, affluent of rio San Juan, South of San Antonio de Maturin, Monagas, 2 Apr 1985, H. Roman. MHNLS 9921 (12, 14.7-24.7 mm SL), caño Inajana, upstream through caño Jotajana, Delta Amacuro, 15 Feb 1993, V. Ponte & W. Wilbert. MHNLS 12465 (26, 14.8-27.8 mm SL), rio Cachipo, setor Cachipo, Reserva Florestal Guarapiche, Maturin, Monagas, 9°57’8”N 63°0’59”W, 16 May 1997, C. Lasso, V. Ponte & D. Figueira. MHNLS 13427 (27, 18.9-29.3 mm SL), Morichal Pozo Vagabundo, affluent of rio Caura, Maripa, Bolívar, 7°23’41”N 65°9’78”W, 15 May 2000, C. Lasso, C. Vispo & K. Nakamura. MHNLS 13428 (2, not measured), Morichal Pozo Vagabundo, affluent of rio Caura, Maripa, Bolívar, 7°23’41”N 65°9’78”W, 15 May 2000, C. Lasso, C. Vispo & K. Nakamura. MHNLS 13434 (14, 14.1-28.4 mm SL), Morichal Merecure, affluent of rio Caura, Maripa, Bolívar, 7°25’49”N 65°10’15”W, 15 May 2000, C. Lasso, C. Vispo & K. Nakamura. MHNLS 13458 (12, 15.2-26.0 mm SL), Morichal Curimar, estrada Maripa-Sipao, affluent of rio Terecay, Bolívar, 7°28’22”N 65°19’07”W, 15 May 2000, C. Lasso, C. Vispo & K. Nakamura. MHNLS 14502 (1, not measured), caño Culebra, affluent of rio Cataniapo, Atures, Amazonas, 5°32’50”N 67°26’53”W, 19 Jun 2001, C. Lasso *et al.* MHNLS 14503 (5, 14.4-48.6 mm SL), caño Las Pavas, affluent of rio Cataniapo, 12.9 km of bridge, Atures, Amazonas, 5°34’26”N 67°30’15”W, 17 Aug 2001, C. Lasso *et al.* MHNLS 14504 (1, not measured), caño Danta, affluent of rio Cataniapo, Danta comunity, Atures, Amazonas, 5°34’32”N 67°32’28”W, 19 Aug 2001, C. Lasso *et al.* MHNLS 14677 (4, not measured), caño Peraman, affluent of rio Cataniapo, at San Pedro community, Atures, Amazonas, 5°34’32”N 67°32’28”W, 23 Apr 2002, J. Fernandez & L. Marcano. MHNLS 14709 (1, not measured), caño Danto, affluent of rio Cataniapo, Atures, Amazonas, 5°24’32”N 67°32’28”W, 24 Apr 2002, J. Fernandez & L. Marcano. MHNLS 14715 (1, not measured), caño Danto, affluent of rio Cataniapo, Atures, Amazonas, 5°24’32”N 67°32’28”W, 24 Apr 2002, J. Fernandez & L. Marcano. MHNLS 14727 (1, not measured), caño Las Pavas, Atures, Amazonas, 5°34’26”N 67°30’15”W, 24 Apr 2002, J. Fernandez & L. Marcano. MHNLS 14791 (1, 25,6 mm SL), rigth bank of caño Culebra, affluent of lower rio Cataniapo, Atures, Amazonas, 5°32’50”N 67°26’53”W, 03 May 2001, J. Fernandez. MHNLS 14850 (1, not measured), Las Pavas, affluent of lower rio Cataniapo, Atures, Amazonas, 5°34’0”N 67°30’30”W, 27 Jun 2001, J. Fernandez. MHNLS 14984 (8, 17.2-28.8 mm SL), Morichal North of Cainama population, Parque Nacional Cainama, rio Caroni drainage, Bolívar, 6°12’35,9”N 62°51’8,7”W, 2 Sep 2002, C. Nascimento. MHNLS 15978 (1, 21.2 mm SL), canõ Lapa, rio Tuparro, Vichada, 16 Jun 2004, M. Lugo. MHNLS 16384 (15, not measured), canõ Culebra, affluent of rio Cataniapo, Puerto Ayacucho, Amazonas, 5°36’6”N 67°36’35”W, 17 Jan 2005, C. Lasso, A. Cervo & J. Fernandez. MHNLS 16487 (5, not measured), canõ Culebra, rigth bank of rio Cataniapo, Puerto Ayacucho, Amazonas, 5°32’50”N 67°26’53”W, 19 Jan 2005, J. Fernandez. MHNLS 16539 (8, 24.3-33.9 mm SL), canõ Danta rigth bank of rio Cataniapo, Ature, Amazonas, 5°34’32”N 67°32’28”W, 20 Jan 2005, J. Fernandez. MHNLS 16748 (1, not measured), canõ Sarrapia, affluent of rio Cataniapo, upstream rocks, Puerto Ayacucho, Amazonas, 27 Jan 2005, J. Fernandez. MHNLS 16779 (2, not measured), canõ Las Pavas, affluent of right margin of rio Cataniapo, Atures, Amazonas, 5°34’26”N 67°30’15”W, 28 Jan 2005, J. Fernandez. MHNLS 16791 (1, not measured), canõ Las Pavas, affluent of rigth bank of rio Cataniapo, Atures, Amazonas, 5°34’26”N 67°30’15”W, 31 Jan 2005, J. Fernandez. MHNLS 16878 (4, not measured), rio Cataniapo, bridge nacional road, Atures, Amazonas, 5°36’15”N 67°35’1”W, 1 Feb 2005, C. Lasso *et al.* MHNLS 16878 (4, not measured), rio Cataniapo, bridge nacional road, Atures, Amazonas, 5°36’15”N 67°35’1”W, 1 Feb 2005, C. Lasso *et al.* MHNLS 18056 (7, 15.1-23.5 mm SL), caño Guapuchi, affluent of rio Ventuari, Amazonas, 4°11’35.7”N 66°44’56.7”W, 25 Jul 2005, C. Lasso *et al.* MHNLS 18169 (2, 25.3 and 34.6 mm SL), caño El Carmen, Amazonas, 3°57’22,3”N 67°5’25”W, 27 Jul 2005, C. Lasso *et al.* MHNLS 18186 (12, 16.0-35.1 mm SL), morichal caño verde, close to Manaka camping, Amazonas, 27 Jul 2005, C. Lasso *et al.* MHNLS 18233 (5, not measured), caño La Guardia, affluent of rio Cataniapo, Mosquito Torto section (at morichal), Apure, 6°49’30.3”N 67°19’43”W, 12 Aug 2005, C. Montaña. MHNLS 18309 (4, 15.5-16.9 mm SL), caño La Guardia, affluent of rio Cataniapo, morichal 3, Apure, 6°49’22,3”N 67°19’6,6”W, 15 Aug 2005, C. Montaña. MHNLS 19041 (34, 17.3-27.3 mm SL), rio Cuao, Raudal, El Danto community, Amazonas, 5°2’39”N 67°33’36,6”W, 2 May 2006, C. Lasso & J. C. Rodriguez. MHNLS 19128 (6, not measured), Tobogan de La Selva, Puerto Ayacucho-Samariapo road, Puerto Ayacucho, Amazonas, 5°23’14,6”N 67°37’0”W, 15 May 2006, C. Lasso & J. C. Rodriguez. SU 59116 (2, 32.1 and 33.9 mm SL), quebrada San Jaime, tributary of rio Amana, 15 km South of Maturin, Monagas, 19 Mar 1939, F. Bond. SU 59325 (4, 22.3-29.1 mm SL), tributary of rio Guarico, Calabozo, Guarico, 14 May 1939, F. Bond. USNM 272404 (137, 13.3-29.5 mm SL), small stream at Road from Puerto Ayacucho to Samariapo, 2 km of Mirabel, Amazonas, 12 Dec 1984, R. Vari, C. Ferrari & O. Castillo. USNM 272403 (25, 12.2-31.1 mm SL), igarapé Guayabal Puerto Ayacucho-Samariapo road, Amazonas, 12 Dec 1984, R. Vari, C. Ferraris & O. Castillo. USNM 235895 (13, 18.8-31.6 mm SL), small stream and swamp on both sides of Rt 19 about 8 km east of rio Aro, 79 km West of Ciudad Bolivar, Bolivar, 4 Nov 1979.

*Rio Amazonas basin, Brazil.* LBP 6911 (7, 20.6-32.0 mm SL), igarapé Km 50, BR 307, rio Negro, São Gabriel da Cachoeira, Amazonas, 0º6.803’S 66º48.744’W, Oliveira *et al.*, 9 Aug 2008. LBP 7105 (1, 29.4 mm SL), igarapé Km 35, BR 307, rio Negro, São Gabriel da Cachoeira, Amazonas, 0º3.381’S 66º51.007’W, Oliveira *et al.*, 17 Aug 2008. MZUSP 74064 (24, 15.5-30.1 mm SL), highland stream at São João, near Santa Isabel do Rio Negro, rio Negro drainage, Amazonas, 0°24’S 65°2’W, 23 Oct 1972, Expedição Permanente da Amazônia. MZUSP 74065 (30, 18.9-29.2 mm SL), highland stream at São João, near Santa Isabel do Rio Negro, rio Negro drainage, Amazonas, 0°24’S 65°2’W, 23 Oct 1972, Expedição Permanente da Amazônia. MZUSP 74066 (2, 19.6 and 24.8 mm SL), highland stream at São João, near Santa Isabel do Rio Negro, rio Negro drainage, Amazonas, 0°24’S 65°2’W, 23 Oct 1972, Expedição Permanente da Amazônia. MZUSP 64575 (3, 47.6-56.1 mm SL), igarapé Yoariwasotoamakúya, affluent of rio Tiquié, Cachoeira Comprida community, rio Negro drainage, Amazonas, 0°15’44”N 70°1’5”W, 23 Oct 2000, Índios Tuyuka. MZUSP 64576 (2, 30.0 and 33.7 mm SL), igarapé do Buriti, Caruru community, rio Tiquié, rio Negro drainage, Amazonas, 0°16’27”N 69°54’56”W, 25 Oct 2000, F. Lima *et al.* MZUSP 64616 (7, 21,7-31,4 mm SL, 3 c&s, 35.0-36.4 mm SL), stream affluent of rio Tiquié, Fronteira community, Brazil-Colombia limit, rio Negro drainage, Amazonas, 31 Oct 2002, F. Lima. MZUSP 64687 (9, 11.7-18.9 mm SL), igarapé Umari at São Pedro community, rio Tiquié, rio Negro drainage, Amazonas, 0°15’41”N 69°57’23”W, Oct 2000, F. Lima *et al.* MZUSP 66658 (2, 15.2 and 17.0 mm SL), mouth of stream upstream Cachoeira do Caruru, Caruru community, rio Tiquié, rio Negro drainage, Amazonas, 0°16’27”N 69°54’56”W, 22 Oct 2000, F. Lima. MZUSP 81292 (3, 13.5-14.5 mm SL), rio Tiquié downstream Cachoeira Comprida community and lower portion of igarapé do Urumutum, rio Negro drainage, Amazonas, 0°15’N 70°1’W, 15 Nov 2002, F. Lima *et al.* MZUSP 81339 (6, 14.0-42.8 mm SL), igarapé Umari Norte, from Cachoeira do Caruru to Cachoeira da Abelha, São Pedro community, rio Tiquié, rio Negro drainage, Amazonas, 0°16’N 69°58’16”W, 2002, F. Lima *et al.* MZUSP 81403 (21, 15.1-21.0 mm SL), igarapé Açaí, near São Pedro community, rio Tiquié, rio Negro drainage, Amazonas, 0°16’N 69°58’W, Nov 2002, F. Lima *et al.* MZUSP 81437 (4, 14.5-20.9 mm SL), rapids upstream cachoeira do Caruru, Caruru community, rio Tiquié, rio Negro drainage, Amazonas, 0°16’29”N 69°54’54”W, 19 Jun 2002, F. Lima. MZUSP 81443 (69, 12.7-55.6 mm SL, 3 c&s, 26.0-44.3 mm SL), igarapé do Buriti, Caruru community, rio Tiquié, rio Negro drainage, Amazonas, 0°16’29”N 69°54’54”W, 20-25 Oct and 8 Nov 2002, F. Lima. MZUSP 81452 (10, 9.3-31.9 mm SL), marginal lagoons downstream São Pedro community, rio Tiquié, rio Negro drainage, Amazonas, 0°16’N 69°56’W, 24 Oct 2002, F. Lima *et al.* MZUSP 81478 (5, 17.7-42.4 mm SL), clear water stream opposite to old São Pedro community, rio Tiquié, rio Negro drainage, Amazonas, 23 Oct 2000, F. Lima *et al.* MZUSP 81494 (14, 11.5-26.5 mm SL), igarapé Mipiriyapotemakãya, affluent of igarapé Açaí, rio Tiquié, rio Negro drainage, Amazonas, 0°15’55”N 69°58’16”W, 29 Oct 2002, F. Lima *et al.* MZUSP 81551 (2, 17.8 and 18.9 mm SL), rio Tiquié, between São Pedro community and Cachoeira da Pedra Curta, rio Negro drainage, Amazonas, 0°16’N 69°58’W, 26 Oct 2002, F. Lima *et al.* MZUSP 84998 (11, 17.3-41.7 mm SL), stream at old São Pedro community, rio Tiquié, rio Negro drainage, Amazonas, 0°16’04,4”N 69°58’21,5”W, 22 Jun 2004, F. Lima. MZUSP 85034 (10, 13.3-41.4 mm SL), black water stram affluent of rio Tiquié, Fronteira community, rio Negro drainage, Amazonas, 0°15’35,2”N 70°2’42,6”W, 24 Jun 2004, F. Lima. MZUSP 85079 (1, 25.3 mm SL), Fronteira community, rio Tiquié, rio Negro drainage, Amazonas, 0°15’35,2”N 70°2’42,6”W, 24 Jun 2004, F. Lima. MZUSP 85149 (107, 12.8-48.3 mm SL, 8 c&s, 12.6-22.0 mm SL), igarapé do Buriti, Caruru community, rio Tiquié, rio Negro drainage, Amazonas, 0°16’N 69°54’W, 29 Jun to 1 Jul 2004, F. Lima. MZUSP 85192 (1, 23.0 mm SL), igarapé at São Tomé community, rio Negro drainage, Amazonas, 1 Jul 2004, F. Lima. MZUSP 92563 (11, 18.2-42.1 mm SL), stream affluent of rio Tiquié, Cunuri community, rio Negro drainage, Amazonas, 0°12’23”N 69°22’28”W, 27 Aug 2006, F. Lima. MZUSP 92573 (62, 8.1-16.8 mm SL, 35 c&s, 6.6-22.5 mm SL), igapó lake of igarapé Castanha affluent rio Tiquié, downstream Santa Rosa community, rio Negro drainage, Amazonas, 0°5’41”N 69°39’W, 3 Aug 2006, F. Lima *et al.* MZUSP 93345 (8, 14.7-41.1 mm SL), stream affluent of rio Tiquié, Serra do Mucura community, rio Negro drainage, Amazonas, 0°10’7”N 69°7’46”W, 10-11 Nov 2006, F. Lima *et al.*

*Colombia.* CZUT-IC 4542 (1, 13.7 mm SL), rio Cuduyarí, Piramiri, Vaupés, 1º22’0”N 70º33’6”W, 7 Nov 2009, F. Villa & A. Ortega. CZUT-IC 4854 (3, 15.9-25.5 mm SL), rio Guaduyuri, San Javier community, Vaupés, 7 Nov 2009, F. Villa & A. Ortega. CZUT-IC 4932 (4, 17.3-50.3 mm SL), caño el Sol, rio Vaupés, rio Negro drainage, Vaupés, 1°12’N 70°4’W, 10 Nov 2009, F. Villa & A. Ortega. CZUT-IC 7881 (1, 45.4 mm SL), rio Papuri, Piracuara community, rio Negro drainage, Yavarate, Vaupés, 21 Jul 2009, J. Suarez. IAvH 2331 (6, 28.3-32.9 mm SL), Quebrada Isue, rio Igará-Paraná, La Chorrera, Amazonas, 0°44’N 73°1’W, 5 Mar 1993, J. Perdomo. ICNMNH 14057 (1, 38.8 mm SL), caño Mituceño, affluent of rio Vaupés, Mitú, Amazonas, 1°13’59,3”N 70°12’34,16”W, 1 Aug 2005, Proyecto Ornamentales Amazonas. ICNMNH 14058 (1, 21.1 mm SL), caño Mituceño, affluent of rio Vaupés, Mitú, Amazonas, 1°13’59.3”N 70°12’34.16”W, 1 Aug 2005, Proyecto Ornamentales Amazonas. ICNMNH 14059 (2, 15.4 and 16.3 mm SL), caño Mituceño, affluent of rio Vaupés, Mitú, Amazonas, 1°13’59.3”N 70°12’34.16”W, 1 Aug 2005, Proyecto Ornamentales Amazonas. ICNMNH 14060 (10, 12.0-20.3 mm SL), caño Mituceño, affluent of rio Vaupés, Mitú, Amazonas, 1°13’59,3”N 70°12’34,16”W, 1 Aug 2005, Proyecto Ornamentales Amazonas. ICNMNH 17170 (3, 18.2-23.3 mm SL), upper rio Apaporis, upstream Jiri-Jirimo, Apaporis, Vaupés, 19 Mar 2009, F. Arbeláez. ICNMNH 11140 (77, 16.8-32.1 mm SL), rio Putumayo drainage, Orito, Putumayo, 1 Jul 204, C. A. Cipamocha. MLS 175 (6, 25.8-27.2 mm SL), Puerto Narino, May 1957, H. Niceforo. MZUSP 85022 (1, 30.6 mm SL), Puerto Colombia community (=Pupunha) and downstream, rio Tiquié, Vaupés, 0°13’54.6”N 70°4’47.7”W, 23 Jun 2004, F. Lima. ZMB 31746 (3, 34.8-36.0 mm SL), near locality of metae, H. Franke.

*Venezuela.* MBUCV 11336 (190, 15.1-36.3 mm SL), cañito at road to Solano, about 6.2 km northeast San Carlos de Rio Negro, rio Negro, 16 Nov 1977, K. Clark. MBUCV 15110 (5, 20.4-34.3 mm SL), caño Chola, at San Carlos de rio Negro-Solano road, rio Negro, Amazonas, 5 Dec 1984, R. Vari *et al.* MBUCV 15124 (5, 19.3-22.1 mm SL), caño Loro, at San Carlos de rio Negro-Solano road, rio Negro, rio Negro, Amazonas, 7 Dec 1984, A. Machado & D. Ibarrara. MCNG 53804 (62, 24.8-42.7 mm SL), rio Baria, 350 meters from camp base, rio Negro, Amazonas, 8 Feb 2005. USNM 272412 (7, 24.0-43.2 mm SL), igarapé Temblador at San Carlos de Rio Negro-Solano road, upstream portion, Amazonas, 5 Dec 1984, R. Vari *et al.* USNM 269899 (27, 19.4-39.8 mm SL), Caño Loro where crossed by road from San Carlos de Rio Negro to Solano, Amazonas, 7 Dec 1984, A. Machado & D. Ibarra. USNM 272409 (6, 19.7-31.1 mm SL), flooded grasslands to side of road from San Carlos de Rio Negro-Solano, about 2 km from San Carlos, Amazonas, 4 Dec 1984, J. Fernandez & O. Castillo. USNM 269898 (9, 16.4-28.3 mm SL), Caño Chola, where crossed by road San Carlos de Rio Negro to Solano, Amazonas, 5 Dec 1984, R. Vari *et al.* USNM 272413 (1, 29.2 mm SL), flooded grasslands to side of road from San Carlos de Rio Negro-Solano, about 2 km from San Carlos, Amazonas, 4 Dec 1984, J. Fernandez & O. Castillo.

**Material examined of *Copella nattereri***

*Type material. Rio Amazonas basin, Brazil.* NMW 56974 (5 paralectotypes of *Pyrrhulina nattereri*, 24.0-32.0 mm SL) and NMW 57148 (3 of 4 paralectotypes of *Pyrrhulina nattereri*, 16.4-31.6 mm SL), same data as the lectotype, see Remarks for additional information. NMW 95055 [ex NMW 57148] (lectotype of *Pyrrhulina nattereri*, 33.7 mm SL), Óbidos [=Pará], in a tributary of the rio Amazonas, 1865, Thayer Expedition. BMNH 909.4.2.28 (1 paralectotype of *Copeina callolepis*), Amazon, J. Arnoldi. MHNG 2577.032 (1 paratype of *Copella meinkeni*, 24.4 mm SL), rio Araçá, lake near Boa Vista, rio Negro, 0º11’S 63º11’W, 1-16 Jan 1987, A. Hanrieder. MNHG 2577.048 (19 paratypes of *Copella meinkeni*, 21.0-31.7 mm SL), rio Araçá, Negro, Amazonas, 0º1’S 63º11’W, 1-31 Jan 1987. MHNG 2205.096 (in part: 15 paratypes of *Copella meinkeni* of 41 non-types, 11.5-20.5 mm SL), rio Tarumã around Manaus, 3º0’34”S 60º4’58”W, 1919 Oct 1965, J. Géry. MTD F 30587 (holotype of *Copella meinkeni*, 40.6 mm SL), clear water stream on Southern Western bank of rio Negro about 5 km downstream Novo Airão, Amazonas, Brazil, 1986, W. Staeck. MTD F 30588-30592 (5 paratypes of *Copella meinkeni*, 37.7-45.0 mm SL), Brazil, Lamarque AM 10 km 21+2, near psciculture, 30 Oct 1978. NMW 56973 (7 paratypes of *Copella meinkeni*, 27.5-37.0 mm SL), Codajás and Tabatinga, Brazil, 1874 donation, Thayer Expedition.

*Non-type material. Rio Amazonas basin, Brazil.* MCZ 6259 (57, 18.3-30.7 mm SL), rio Amazonas at Óbidos [approximately 1º52’S 55º30”W], Nov-Dec 1865, Thayer Expedition. MHNG 2200.020 (9, 22.1-41.1 mm SL), stream at Rio Preto da Eva, at Manaus-Itacoatiara road, Amazonas, 21 Nov 1976, H. Axelrodi, M. Brittan & B. Frank. MHNG 2200.021 (2, 33.1 and 40.8 mm SL), Santa Isabel do Rio Negro, upper rio Negro, Amazonas, Oct 1962. MHNG 2205.095 (7 of 14, 17.0-23.4 mm SL), igarapé Aduja, rio Itu, rio Negro, Amazonas, 0º44'N 63°41'W, 10 Nov 1962. MHNG 2205.097 (1, 18.8 mm SL), “rio Negro?”, Amazonas, 1 Oct 1965. MHNG 2575.099 (3, 24.4-27.5 mm SL), rio Araça, rio Negro, Amazonas, 1 Jan 1983-31 Dec 1984. MHNG 2576.050 (5, 12.0-18.8 mm SL), igarapé Mamolé, tributary of rio Cuiuni, rio Negro, Amazonas, 0º45'S 63°9'W, 16-17 Nov 1984. MHNG 2576.069 (10 of 14, 8.1-20.6 mm SL), rigth bank of lower portion of stream tributary of rio Tapajós, surrounding the city upstream, Santarém, Pará, 27 Sep 1980, S. Kullander. MHNG 2711.041 (12, 27.1-29.2 mm SL), rio Araçá, Negro, Amazonas, 1’S 63º11’W, 1-16 Jan 1987. A. Hanrieder. MPEG 130 (1, 30.4 mm SL), Ilha de Tamaquaré, rio Negro, Santa Isabel do Rio Negro, Amazonas, 7 Feb 1980, M. Golding. MPEG 131 (8, 16.8-27.1 mm SL), rio Negro, Anavilhanas, Novo Airão, Amazonas, 1 Apr 1982, M. Golding. MPEG 132 (1, 24.0 mm SL), Ilha de Buiu-açú, near rio Urubaxi, rio Negro, Santa Isabel do Rio Negro, Amazonas, 6 Feb 1980, M. Golding. MPEG 9290 (1, 30.0 mm SL), headwater of igarapé Juruti Grande, Amazonas, Juruti, Pará, 2º34'29.7"S 56°24'12.2"W, 5 Aug 2004, W. Wosiacki. MPEG 9291 (2, 27.8 and 39.0 mm SL), headwater of igarapé Juruti Grande, Juruti, Pará, 2º34'36"S 56°24'3"W, 4 Aug 2004, W. Wosiacki. MPEG 9820 (7, 10.8-21.4 mm SL), igarapé Juruti Grande, rigth tributary, Juruti, Pará, 2º29'39.4"S 56°20'43.8"W, 6 Sep 2002, W. Wosiacki. MPEG 10936 (9, 15.2-25.2 mm SL), igarapé Itapiranga, Juruti, Pará, 2º28.2'13.7"S 56°11'40,4"W, 23 Aug 2006, A. Hercos. MPEG 10938 (2, 20.7 and 35.5 mm SL), igarapé Itapiranga, Juruti, Pará, 2º28.2'13.7"S 56°11'40.5"W, 22 Aug 2006, A. Hercos. MPEG 10943 (1, 16.4 mm SL), igarapé Itapiranga, Juruti, Pará, 2º28.2'13.7"S 56°11'40.4"W, 22 Aug 2006, A. Hercos. MPEG 10960 (9, 13.7-18.7 mm SL), igarapé Itapiranga, Juruti, Pará, 2º28.2'13.7"S 56°11'40.4"W, 22 Aug 2006, A. Hercos. MPEG 12913 (5, 15.5-21.2 mm SL), igarapé Itapiranga, Juruti, Pará, 16 Dec 2006, L. Montag. MPEG 12915 (1, 20.0 mm SL), igarapé Itapiranga, Juruti, Pará, 30 Dec 2006, L. Montag. MPEG 12946 (1, 21.5 mm SL), igarapé Itapiranga, Juruti, Pará, 16 Dec 2006, L. Montag. MPEG 12948 (2, 21.7 and 21.9 mm SL), igarapé Guaraná, Juruti, Pará, 16 Dec 2006, L. Montag. MPEG 13765 (6, 28.5-33.4 mm SL), igarapé Itapiranga, Juruti, Pará, 2º28.2'S 56°11.51'W, 13 May 2007, A. Hercos. MPEG 13788 (2, 18.2 and 27.0 mm SL), igarapé Itapiranga, Juruti, Pará, 2º28.2'S 56°11.513'W, 13 May 2007, A. Hercos. MPEG 14225 (5, 23.4-31.1 mm SL), igarapé Itapiranga, Juruti, Pará, 2º28.2'S 56°11.513'W, 29 Nov 2007, A. Hercos. MPEG 14227 (4, 19.9-27.5 mm SL), igarapé Itapiranga, Juruti, Pará, 2º28.2'S 56°11.513'W, 29 Nov 2007, A. Hercos. MPEG 14233 (2, 17.0 and 19.4 mm SL), igarapé Guaraná, Juruti, 2º29.77'S 56º13.84'W, 28 Nov 2007. MPEG 14347 (6, 20.5-32.8 mm SL), igarapé Mutum, Juruti, Pará, 2º36.8'S 56°11.6'W, 27 Nov 2007, A. Hercos. MPEG 14430 (2, 15.2 and 23.9 mm SL), rio Sacará, Trombetas, Oriximiná, Pará, 1º48'9.3”S 56°16'32.9”W, 20 Oct 2007, W. Wosiacki. MPEG 14596 (2, 21.2 and 22.1 mm SL), rio Sacará, Trombetas, Oriximiná, Pará, 1º48'9.3”S 56°16'32.9”W, 20 Oct 2007, W. Wosiacki. MPEG 15108 (2, 21.0 and 21.4 mm SL), igarapé do Piraquara, Nhamundá, Faro, Pará, 1º50'43.5”S 57°12'53.6”W, 23 Jan 2008, L. Montag. MPEG 15329 (4, 24.1-29.7 mm SL), Trilha T1, Nhamundá, Faro, Pará, 1º42'23.3”S 57°12'10.1”W, 26 Jan 2008, L. Montag. MPEG 15352 (1, 32.9 mm SL), Trilha T1, Nhamundá, Faro, Pará, 1º42'23.3”S 57°12'10.1”W, 26 Jan 2008, L. Montag. MPEG 15429 (5, 18.1-23.8 mm SL), Porto Cikel, Nhamundá, Faro, Pará, 1º42'48.4”S 57°12'48.4”W, 27 Jan 2008, L. Montag. MPEG 15905 (9, 9.9-33.9 mm SL), igarapé Itaubalzinho, affluent of rio Paraconi, FLONA do Pau Rosa, Maués, Amazonas, 3º50'18.3”S 58°13’0”W, 16 Feb 2009, F. Silva. MPEG 15906 (1, 28.8 mm SL), igarapé Itaubalzinho, affluent of rio Paraconi, FLONA do Pau Rosa, Maués, Amazonas, 3º49'50.2”S 58°17'36.5”W, 17 Feb 2009, F. Silva. MPEG 15907 (67, 15.9-34.5 mm SL), igarapé Itaubalzinho, affluent of rio Paraconi, FLONA do Pau Rosa, Maués, Amazonas, 3º46'38.4”S 58°15'12.9”W, 15 Feb 2009, F. Silva. MPEG 15908 (24, 23.8-33.9 mm SL), igarapé Cipoteua, rio Paraconi drainage, FLONA do Pau Rosa, Maués, Amazonas, 3º46'57.1”S 58°19'0.6”W, 14 Feb 2009, F. Silva. MPEG 15909 (9, 22.6-33.9 mm SL), igarapé Itaubalzinho, affluent of rio Paraconi, FLONA do Pau Rosa, Maués, Amazonas, 3º43'36.9”S 58°36.9'58”W, 13 Feb 2009, F. Silva. MPEG 15911 (20, 24.3-31.9 mm SL), igarapé Itaubalzinho, affluent of rio Paraconi, FLONA do Pau Rosa, Maués, Amazonas, 3º56'25.2”S 58°26'43.4”W, 25 Feb 2009, F. Silva. MPEG 15912 (5, 24.4-33.2 mm SL), igarapé Uixi, affluent of rio Paraconi, FLONA do Pau Rosa, Maués, Amazonas, 3º46'38.4”S 58°15'12.9”W, 15 Feb 2009, F. Silva. MPEG 15913 (41, 21.0-33.4 mm SL), affluent of igarapé Palhai, rio Paraconi drainage, FLONA do Pau Rosa, Maués, Amazonas, 4º13'6.6”S 58°16'54.9”W, 21 Feb 2000, F. Silva. MPEG 15914 (28, 13.7-30.6 mm SL), igarapé Itaubalzinho, affluent of rio Paraconi, FLONA do Pau Rosa, Maués, Amazonas, 3º56'23.2”S 58°22'4.5”W, 23 Feb 2009, F. Silva. MPEG 15915 (9, 21.8-24.7 mm SL), igarapé Itaubaizinho, affluent of rio Paraconi, FLONA do Pau Rosa, Maués, Amazonas, 4º48'56.8”S 58°17'42.5”W, 18 Feb 2009, F. Silva. MPEG 15919 (4, 16.5-30.1 mm SL), drainage of Lago do Elias, Paranã do Uraria, Maués, Amazonas, 3º43'29.8”S 58°16'18.3”W, 12 Feb 2009, F. Silva. MPEG 15974 (5, 10.4-14.5 mm SL), igarapé Azedal, rio Paraconi drainage, FLONA do Pau Rosa, Maués, Amazonas, 3º58'12.2”S 58°24'16.1”W, 24 Feb 2009, F. Silva. MPEG 15975 (1, 14.0 mm SL), igarapé Itaubaizinho, rio Paraconi drainge, FLONA do Pau Rosa, Maués, Amazonas, 3º48'56.8”S 58°17'42.5”W, 18 Feb 2009, F. Silva. MPEG 15976 (1, 22.8 mm SL), igarapé Palhalzinho, rio Paraconi drainage, FLONA do Pau Rosa, Maués, Amazonas, 3º49'50.2”S 58°17'36.5”W, 17 Feb 2009, F. Silva. MPEG 15977 (14, 14.9-27.1 mm SL), igarapé Itaubalzinho, affluent of rio Paraconi, FLONA do Pau Rosa, Maués, Amazonas, 4º6'6.1”S 58°18'58.4”W, 22 Feb 2009, F. Silva. MPEG 15978 (1, 26.0 mm SL), Lago do Elias, Paranã do Urariá, surroundings of FLONA do Pau Rosa, Maués, Amazonas, 3º43'29.8”S 58°16'18.3”W, 12 Feb 2009, F. Silva. MPEG 16019 (7, 8.8-25.0 mm SL), igarapé Itaubalzinho, affluent of rio Paraconi, FLONA do Pau Rosa, Maués, Amazonas, 3º38'12.2”S 58°24'16.1”W, 24 Feb 2009, F. Silva. MPEG 16021 (1, 25.9 mm SL), igarapé Itaubalzinho, affluent of rio Paraconi, FLONA do Pau Rosa, Maués, Amazonas, 3º56'25.2”S 58°26'43.4”W, 25 Feb 2009, F. Silva. MPEG 16039 (32, 10.5-17.7 mm SL), igarapé Itaubalzinho, affluent of rio Paraconi, FLONA do Pau Rosa, Maués, Amazonas, 3º48'56.8”S 58°17'42.5”W, 15 Mar 2009, F. Silva. MPEG ex 15917 (21, 11.7-32.2 mm SL), igarapé Tamoatá, rio Paraconi drainage, FLONA do Pau Rosa, Maués, Amazonas, 3º44'47.1”S 58°18'7.6”W, 14 Feb 2009, F. Silva. MZUSP 5576 (1, 17.5 mm SL), lagoa Parauacuí, Trombetas, Oriximiná, Pará, 1º46'S 55°52'W, 9 Feb 1967, Expedição Permanente da Amazônia. MZUSP 5810 (25, 14.4-21.1 mm SL), lago Saracá, Amazonas, Silves, Amazonas, 2º52'S 58°22'W, 17-18 Mar 1967, Expedição Permanente da Amazônia. MZUSP 6233 (7, 13.4-25.7 mm SL), lake at rio Negro margin upstream Manaus, rio Negro, Amazonas, 3º10'S 60°0'W, 28 Apr 1967, Expedição Permanente da Amazônia. MZUSP 6804 (8, 22.3-34.2 mm SL), igarapé do Rei, island close to the rio Cuieiras mouth, rio Negro, Amazonas, 2º42'S 60°20'W, 26 Jan 1977, Alpha Helix Amazon Expedition. MZUSP 6827 (81, 13.3-26.5 mm SL), igarapé Tarumãzinho and affluent number 1, North of Manaus, rio Negro, Amazonas, 3º2'S 60°9'W, Nov 1967, Expedição Permanente da Amazônia. MZUSP 6930 (32, 13.4-20.7 mm SL), stream of lago Puraquequara, Amazonas, 2º56'S 59°49'W, 23 Nov 1967, Expedição Permanente da Amazônia. MZUSP 7289 (91, 12.1-30.8 mm SL), stream of rio Maraú, Maués, Amazonas, 3º24'S 57°42'W, 3 Dec 1967, Expedição Permanente da Amazônia. MZUSP 7346 (379, 11.7-31.3 mm SL, 1 c&s, 31.6 mm SL), igarapé Limãozinho, Maués, Amazonas, 3º24'S 57°42'W, 4 Dec 1967, Expedição Permanente da Amazônia. MZUSP 7411 (25, 16.9-33.6 mm SL), stream of lago Saracá, Silves, Amazonas, 2º52'S 58°22'W, 6 Dec 1967, Expedição Permanente da Amazônia. MZUSP 7476 (110, 17.5-32.4 mm SL), rio Sanabani, Silves, Amazonas, 2º45'S 58°20'W, 7-8 Dec 1967, Expedição Permanente da Amazônia. MZUSP 7798 (2, 18.5 and 21.5 mm SL), stream of lago José-Açu, Parintins, Amazonas, 2º40'S 56°37'W, 11-12 Dec 1967, Expedição Permanente da Amazônia. MZUSP 8408 (118, 11.8-32.7 mm SL), igarapé Jacundá, Alter do Chão, Tapajós, Santarém, Pará, 2º30'S 54°57'W, 23 Dec 1967, Expedição Permanente da Amazônia. MZUSP 8448 (75, 11.9-23.5 mm SL), igarapé Jacundá, Alter do Chão, Tapajós, Santarém, Pará, 2º30'S 54°57'W, 23 Dec 1967, Expedição Permanente da Amazônia. MZUSP 8488 (110, 14.1-26.9 mm SL), igarapé affluent of left bank of rio Mapiri, Amazonas, Santarém, Pará, 2º26'S 54°44'W, 25 Dec 1967, Expedição Permanente da Amazônia. MZUSP 15550 (17, 14.5-32.3 mm SL, 2 c&s, 28.5-33.2 mm SL), lago Jacaré, headdwaters of serrinha, Reserva Biológica de Trombetas, Trombetas, Pará, 1º20'S 56°51'W, 25 Jul 1979, R. Castro. MZUSP 23575 (3, 13.2-14.0 mm SL), lagoon of Mr. Durval Magalhães, Tapequém-Roraima road, 47 km from Boa Vista, rio Branco, Boa Vista, Roraima, 2º50'N 60°40'W, 9 Feb 1969, T. Roberts. MZUSP 23673 (19, 10.1-24.7 mm SL), lago Jacaré, Trombetas, Pará, 1º20'S 56°51'W, 7-11 Oct 1969, Expedição Permanente da Amazônia. MZUSP 24246 (1, 18.0 mm SL), igarapé Jituarana, rigth bank of rio Tapajós near Boim, Tapajós, Pará, 3º0'S 55°15'W, 27 Oct 1970, Expedição Permanente da Amazônia. MZUSP 24964 (1, 18.3 mm SL), rio Cuieiras and affluents, rio Negro, Amazonas, 2º50'S 60°30'W, Jan 1977, Alpha Helix Amazon Expedition. MZUSP 27454 (1, 22.9 mm SL), Pedra do Gavião, rio Negro, Barcelos, Amazonas, 1º28'S 61°38'W, Nov 1983, L. Portugal. MZUSP 27456 (9, 17.9-23.2 mm SL), Pedra do Gavião, rio Negro, Barcelos, Amazonas, 1º28'S 61°38'W, 13-14 Nov 1982, L. Portugal. MZUSP 29342 (94, 12.6-27.2 mm SL), rio Negro downstream rio Daraá (central lake in island), rio Negro, Amazonas, 0º28'S 64°46'W, 17 Feb 1980, M. Golding. MZUSP 29343 (79, 20.5- 33.6 mm SL, 4 c&s, 23.9-32.3 mm SL), lake in island, rio Negro, Barcelos, Amazonas, 0º58'S 62°57'W, 29 Feb 1980, M. Golding. MZUSP 29344 (265, 20.5-33.6 mm SL), Anavilhanas, rio Negro, Amazonas, 2º42'S 60°45'W, Mar 1982, M. Golding. MZUSP 29345 (2, 18.1 and 24.0 mm SL), central lake of ilha de Buiu-Açu, near rio Urubaxi, rio Negro, Amazonas, 0º31'S 64°50'W, 6 Feb 1980, M. Golding. MZUSP 29346 (6, 14.3-16.4 mm SL), central lake of ilha de Buiu-Açu, near rio Urubaxi, rio Negro, Amazonas, 0º31'S 64°50'W, 6 Feb 1980, M. Golding. MZUSP 31334 (1, 30,7 mm SL), igapó at Anavilhanas, rio Negro, Amazonas, 2º42'S 60°45'W, Apr 1982, M. Golding. MZUSP 55135 (3, 18.7-26.4 mm SL), São João near Santa Isabel do Rio Negro, margins of lake in island, rio Negro, Amazonas, 21 Oct 1972, Expedição Permanente da Amazônia. MZUSP 58331 (5, 17.3-26.1 mm SL), chanel between lakes at São João near Santa Isabel do Rio Negro, rio Negro, Amazonas, 0º24'S 65°2'W, 24 Oct 1972, Expedição Permanente da Amazônia. MZUSP 58609 (37, 19.2-28.7 mm SL), lake of rio Aiuanã, rio Negro, Amazonas, 29 Oct 1972, Expedição Permanente da Amazônia. MZUSP 58611 (2, 18.5 and 21.1 mm SL), pool of rock at rio Negro, rio Negro, Barcelos, Amazonas, 0º12'S 64°4'W, 1 Nov 1972, Expedição Permanente da Amazônia. MZUSP 59166 (75, 17.5-31.6 mm SL), lake in lowland at Cantagalo, rio Negro, Amazonas, 28 Jan 1972, Expedição Permanente da Amazônia. MZUSP 59955 (7, 16.0-28.2 mm SL), highland stream at Cantagalo, rio Negro, Amazonas, 24 Jan 1972, Expedição Permanente da Amazônia. MZUSP 61926 (7, 19.2-25.5 mm SL), rio Aiuanã, near Santa Isabel do Rio Negro, rio Negro, Amazonas, 0º24'S 65°2'W, 22 Oct 1972, Expedição Permanente da Amazônia. MZUSP 62156 (3, 17.3-29.0 mm SL), lagoon in island of rio Negro, Paricatuba, rio Negro, Santa Isabel do Rio Negro, Amazonas, 0º31'S 65°1'W, 14 Nov 1972, Expedição Permanente da Amazônia. MZUSP 62167 (8, 12.5-19.3 mm SL), stream of lowland at rio Negro, Santa Isabel do Rio Negro, rio Negro, Amazonas, 0º24'S 65°2'W, 19 Oct 1972, Expedição Permanente da Amazônia. MZUSP 62202 (8, 12.5-27.3 mm SL), chanel between lakes at São João near Santa Isabel do Rio Negro, rio Negro, Amazonas, 0º24'S 65°2'W, 24 Oct 1972, Expedição Permanente da Amazônia. MZUSP 62225 (3, 17.9-19.6 mm SL), lagoon in island of rio Negro, Paricatuba, Santa Isabel do Rio Negro, Amazonas, 0º31'S 65°1'W, 14 Nov 1972, Expedição Permanente da Amazônia. MZUSP 63517 (12, 12.9-27.8 mm SL), stream of lowland at Santa Isabel do Rio Negro, rio Negro, Amazonas, 0º24'S 65°2'W, 19 Oct 1972, Expedição Permanente da Amazônia. MZUSP 63522 (4, 23.7-26.8 mm SL), rio Aiuanã near Santa Isabel do Rio Negro, lake and stream headwater of lake, rio Negro, 0º24'S 65°2'W, 22 Oct 1972, Expedição Permanente da Amazônia. MZUSP 63523 (6, 19.0-33.0 mm SL), rio Aiuanã, near Santa Isabel do Rio Negro, lake and stream headwater lake, rio Negro, Amazonas, 0º24'S 65°2'W, 22 Oct 1972, Expedição Permanente da Amazônia. MZUSP 63524 (2, 20.9 and 26.8 mm SL), rio Aiuanã, near Santa Isabel do Rio Negro, lake and stream headwater lake, rio Negro, Amazonas, 22 Oct 1972, Expedição Permanente da Amazônia. MZUSP 63525 (1, 16.5 mm SL), stream of lowland at Tapurucuara, Amazonas, 0º24'S 65°2'W, 19 Oct 1972, Expedição Permanente da Amazônia. MZUSP 63527 (2, 24.9 and 34.7 mm SL), São João near Santa Isabel do Rio Negro, rio Negro, Amazonas, 0º24'S 65°2'W, 24 Oct 1972, Expedição Permanente da Amazônia. MZUSP 63528 (1, 22.1 mm SL), São João, Santa Isabel do Rio Negro, rio Negro, Amazonas, 0º24'S 65°2'W, 27 Oct 1972, Expedição Permanente da Amazônia. MZUSP 63529 (2, 18.5 and 23.58 mm SL), São João, near Santa Isabel do Rio Negro, rio Negro, Amazonas, 0º24'S 65°2'W, 24 Oct 1972, Expedição Permanente da Amazônia. MZUSP 63530 (4, 18.1-24.9 mm SL), São João near Santa Isabel do Rio Negro, highland stream at rio Negro, Amazonas, 0º24'S 65°2'W, 23 Oct 1972, Expedição Permanente da Amazônia. MZUSP 66723 (7, 16.9-22.4 mm SL), São João, near Santa Isabel do Rio Negro, margins of lake in island, rio Negro, Amazonas, 21 Oct 1972, Expedição Permanente da Amazônia. MZUSP 66724 (1, 18.1 mm SL), São João, near Santa Isabel do Rio Negro, margins of lake in island, rio Negro, Amazonas, 21 Oct 1972, Expedição Permanente da Amazônia. MZUSP 66729 (21, 17.8-26.3 mm SL), stream of lowland at Santa Isabel do Rio Negro, rio Negro, Amazonas, 0º24'S 65°2'W, 19 Oct 1972, Expedição Permanente da Amazônia. MZUSP 66730 (8, 12.7-19.4 mm SL), stream of lowland at Santa Isabel do Rio Negro, rio Negro, Amazonas, 0º24'S 65°2'W, 19 Oct 1972, Expedição Permanente da Amazônia. MZUSP 66732 (4, 20.6-26.8 mm SL), São João near Santa Isabel do Rio Negro, margins of lake in island rio Negro, Amazonas, 21 Oct 1972, Expedição Permanente da Amazônia. MZUSP 66733 (10, 19,7-31,5 mm SL), São João near Santa Isabel do Rio Negro, margins o lake in island, rio Negro, Amazonas, 21 Oct 1972, Expedição Permanente da Amazônia. MZUSP 66745 (7, 15,3-28,7 mm SL), stream at São João near Santa Isabel do Rio Negro, rio Negro, Amazonas, 0º24'S 65°2'W, 27 Oct 1972, Expedição Permanente da Amazônia. MZUSP 66750 (1, 27.7 mm SL), stream at São João, near Santa Isabel do Rio Negro, rio Negro, Amazonas, 0º24'S 65°2'W, 27 Oct 1972, Expedição Permanente da Amazônia. MZUSP 66766 (20, 14.2-35.1 mm SL), São João near Santa Isabel do Rio Negro, chanel of lake, rio Negro, Amazonas, 0º24'S 65°2'W, 23 Oct 1972, Expedição Permanente da Amazônia. MZUSP 66767 (13, 12.3-20.5 mm SL), São João near Santa Isabel do Rio Negro, chanel of lake, rio Negro, Amazonas, 0º24'S 65°2'W, 23 Oct 1972, Expedição Permanente da Amazônia. MZUSP 74233 (19, 15.0-23.5 mm SL), stream of lowland at Santa Isabel do Rio Negro, rio Negro, Amazonas, 0º24'S 65°2'W, 19 Oct 1972, Expedição Permanente da Amazônia. MZUSP 74236 (5, 21.8-27.2 mm SL), lake of rio Aiuanã, rio Negro, Amazonas, 0º38'S 64°56'W, 29 Oct 1972, Expedição Permanente da Amazônia. MZUSP 74237 (13, 15.0-28.5 mm SL), lake of rio Aiuanã, rio Negro, Amazonas, 0º38'S 64°56'W, 29 Oct 1972, Expedição Permanente da Amazônia. MZUSP 74238 (21, 18.0-25.6 mm SL), lake of rio Aiuanã, rio Negro, Amazonas, 0º38'S 64°56'W, 29 Oct 1972, Expedição Permanente da Amazônia. MZUSP 74264 (39, 18.3-30.0 mm SL), igarapé Jaradá, affluent of rigth margin of rio Cuieiras, about 40 km of mouth, rio Negro, Manaus, Amazonas, 31 Jan 1977, Alpha Helix Amazon Expedition. MZUSP 74287 (5, 20.4-27.7 mm SL), igarapé Sirinau, rigth margin of rio Cuieiras, about 25 km of mouth, rio Negro, Manaus, Amazonas, 2º42'S 60°20'W, 30 Jan 1977, Alpha Helix Amazon Expedition. MZUSP 74519 (7, 20.8-24.1 mm SL), igarapé Arraia, rigth margin of rio Cuieiras, about 25 km of mouth, rio Negro, Amazonas, 2º42'S 60°20'W, 27 Jan 1977, Alpha Helix Amazon Expedition. MZUSP 74774 (3, 18.3-21.9 mm SL), igarapé Arraia, rigth margin of rio Cuieiras, about 25 km of mouth, rio Negro, Amazonas, 2º42'S 60°20'W, 27 Jan 1977, Alpha Helix Amazon Expedition. MZUSP 74781 (8, 21.9-32.2 mm SL), igarapé do Rei, island close to rio Cuieiras mouth, rio Negro, Amazonas, 2º42'S 60°20'W, 26 Jan 1977, Alpha Helix Amazon Expedition. MZUSP 85222 (16, 18.8-24.6 mm SL), stream headwater of lake at Cantagalo, rio Negro, Amazonas, 25 Jan 1972, Expedição Permanente da Amazônia. MZUSP 85223 (3, 15.3-17.1 mm SL), stream of lowland at Ilha Grande de Tapurucuara, rio Negro, Amazonas, 12 Nov 1972, Expedição Permanente da Amazônia. MZUSP 85224 (5, 13.5-28.3 mm SL), rocky pool communicating with rio Negro, Tapera, rio Negro, Amazonas, 0º12'S 64°4'W, 1 Nov 1972, Expedição Permanente da Amazônia. MZUSP 87424 (17, 13.0-23.2 mm SL), floodplain at pousada do Paraíso, near igarapé do Tauari, Rio Preto da Eva, Amazonas, 2º47'25.2"S 59°38'10.8"W, 5 Jul 2003, Exc. MZUSP/USP. MZUSP 87425 (1, 14.7 mm SL), igarapé Água Verde, at Road on the left margino f Rio Preto da Eva, downstream city, Rio Preto da Eva, Amazonas, 2º44'22,7"S 59°41'15,5"W, 5 Jul 2003, Exc. MZUSP/USP. MZUSP 87426 (24, 11.4-42.2 mm SL, 2 c&s, 26.5-38.7 mm SL), stream affluent of Rio Preto da Eva, Rio Preto da Eva, Amazonas, 2º44'35"S 59°40'7.8"W, 6 Jul 2003, Exc. MZUSP/USP. MZUSP 87427 (4, 13.5-26.7 mm SL), igarapé Agripino, affluent of Rio Preto da Eva, Rio Preto da Eva, Amazonas, 2º43'59.3"S 59°40'48"W, 6 Jul 2003, Exc. MZUSP/USP. MZUSP 87428 (1, 15.2 mm SL), rio Urubu, at Patauá farm, ramal ZF-9, Km 103-104 of road to Itacoatiara, Rio Preto da Eva, Amazonas, 2º39'37,8"S 59°22'21.7"W, 8 Jul 2004, Exc. MZUSP/USP. MZUSP 92144 (1, 17.4 mm SL), stream affluent of rio Tiquié, Serra do Mucura community, rio Negro, Amazonas, 0º10'7"N 69°7'46"W, 10 Aug 2006, F. Lima *et al.*. MZUSP 92838 (5, 14.0-19.3 mm SL), igarapé Juá, 7 km from Santarém by Road to the airport, Amazonas, Santarém, Pará, 2º26'S 54°46'52"W, 13 Nov 2006, L. Sousa & J. Birindelli. MZUSP 92855 (1, 18.4 mm SL), imprecise locality (aquarium store of Samuel at Santarém), 17 Oct 2006, L. Sousa & J. Birindelli. MZUSP 95276 (9, 17.1-28.1 mm SL), stream at left margin of rio Cuieiras, about 20 km of mouth, rio Negro, Manaus, Amazonas, 3º0'S 60°11'W, 27 Jan 1977, Alpha Helix Amazon Expedition. MZUSP 108818 (2, 21.0 and 25.5 mm SL), entrance of igarapé Demuriari, left margin of rio Negro, upstream São Gabriel da Cachoeira, leaves at floodplain area, rio Negro, São Gabriel da Cachoeira, Amazonas, 0º0'51"S 67°10'16"W, 12 Dec 2005, J. Muriel-Cunha, J. Zuanon. MZUSP 108819 (1, 25.8 mm SL), igarapé Barixia, rigth margino of rio Negro, rio Negro, São Gabriel da Cachoeira, Amazonas, 0º7'24"S 67°7'54"W, 14 Dec 2005, J.Muriel-Cunha, J. Zuanon & E. Trajano. NRM (3, 11.4-25.7 mm SL), stream at savanah at upper rio Araçá, rio Negro, Amazonas, 11 Nov 1984. MZUSP 24255 (52, 14.7-28.1 mm SL), igarapé-Açú, Tapajós, Aveiro, Pará, 30 Oct 1970, Expedição Permanente da Amazônia. MZUSP on loan Marilyn (3, 13.4-15.0 mm SL), Boa Vista, Roraima, 9-10 Feb 1969. MZUSP 112781 (5, 18.0-25.5 mm SL), igarapé Bandeira, affluent of rio Jufari, Caracaaí, Roraima, 1º2’56”S 62º8’43”W, 3 Sep 2011, Oyakawa *et al.* MZUSP 112541 (53, 23.5-32.29 mm SL), igarapé do Campo, affluent of rio Jufari, near Caicubi village, Caracaraí, Roraima, 1º4’1”S 62º7’40”W, 28 Aug 2011, Oyakawa *et al.* MZUSP 112789 (14, 11.5-24.0 mm SL), igarapé Pretinho, affluent of igarapé Caicubi, Caracaraí, Roraima, 0º56’46”S 62º6’26”W, 31 Aug 2011, Oyakawa *et al.* MZUSP 113116 (46, 10.0-32.0 mm SL), igarapé Santa Fé, affluent of rio Jufari, Caracaraí, Roraima, 1º0’34”S 62º13’4”W, 1 Sep 2011, Oyakawa *et al.* MZUSP 113139 (40, 9.5-25.0 mm SL), mouth of igarapé Pupunha at igarapé Caicubi, Caracaraí, Roraima, 0º59’25”S 62º5’56”W, 5 Sep 2011, Oyakawa *et al.* MZUSP 113194 (11, 14.1-30.4 mm SL), igarapé Tubana, affluent of rio Jurai, rio Negro, 1º5’25”S 62º7’35”W, 5 Sep 2011, Oyakawa *et al.* MZUSP 113276 (2, 31.1-31.8 mm SL), igarapé Pretinho, affluent of igarapé Caicubi, Caracaraí, Roraima, 1º1’46”S 62º6’60”W, 26 Aug 2011, Oyakawa *et al.* MZUSP 113354 (24, 9.9-30.2 mm SL), igarapé Pretinho, upstream mouth of igarapé Branquinho, rio Negro, Caracaí, Roraima, 0º55’50”S 62º6’30”W, 9 Set 2011, Oyakawa, 2012. MZUSP 113382 (3, 19.8-22.3 mm SL), temporary pool conecting with igarapé Pupunha, 1.9 km behind Vila de Caicubi, rio Negro, Roraima, 1º1’32”S 62º5’47”W, 11 Set 2011, O. Oyakawa *et al.* UFRO-I 8422 (2, 16.6 and 23.3 mm SL), lago Sampaio, rio Madeira mouth, Nova Olinda do Norte, Amazonas, 3º45'50.6"S 59°1.6'45.3”W, 25 Jan 2011, F. Vieira. USNM 300956 (17, 15.9-24.4 mm SL), rio Unini, rio Negro, Amazonas, 19 Nov 1988, H. Axelrod. USNM 317518 (2, 30.2 and 30.6 mm SL), rio Negro, downstream rio Daraá, central lake in sland, rio Negro, Amazonas, 11 Feb 1980, M. Golding.

*Colombia.* ICNMNH 6214 (4, 17.4-28.9 mm SL), quebrada La Arenosa, Km 9.5 at Leticia-Tarapacá road, 8 Nov 2001, C. Castellanos. ICNMNH 16375 (3, 22.3-28.4 mm SL), quebrada La Arenosa, Km 10.5 Letícia-Tarapaca road, Finca Agape, Leticia, 1 Oct 2005, Proyecto Ornamentales Amazonas. ICNMNH 13609 (1, 20.9 mm SL), quebrada La Arenosa, Km 11 Letícia-Tarapacá road, Letícia, Amazonas, 19 Nov 1997, J. Mojica & Estudiantes Biología. ICNMNH 16891 (10, 15.6-34.4 mm SL), quebrada La Arenosa, Km 10.5 Leticia-Tarapacá road, Leticia, 1 Apr 2007, J. I. Mojica & C. Castellanos. SMF 9925 (4, 27.1-32.1 mm SL), igarapé Preto, upper rio Solimões, Brazil, H. Schultz, 1961. SMF 9927 (2, 28.5-33.7 mm SL), igarapé Preto, upper rio Solimões, Brazil, H. Schultz, 1961. USNM 311005 (6, 19.8-31.9 mm SL), meadow stream near Leticia, 3 Dec 1974, D. Kramer.

*Rio Orinoco basin, Colombia.* CZUT-IC 4289 (1, 18.8 mm SL), rio Guainia, San Jose-Playa community, Guainia, 10 Dec 2009-29 May 2010, F. Villa & A. Ortega. CZUT-IC 4297 (10, 11.6-23.0 mm SL), caño Piedras, rio Guainia, Guainia, 2º6'17”N 67º6'40”W, 9 Dec 2009-29 May 2010, F. Villa & A. Ortega. CZUT-IC 4316 (1, 15.2 mm SL), caño Guamirza, rio Guainia, Guainia, 2º58'13”N 67º50'13”W, 10 Dec 2009, F. Villa & A. Ortega. IavH 2259 (4, 15.5-20.8 mm SL), laguna El Pavón of caño Bocón, rio Inírida, Puerto Inírida, Guainia, 3º40'N 67°57'W, M. Blanco *et al.* IavH 2289 (1, 25.1 mm SL), caño Bocón, rio Inírida, Puerto Inírida, Guainia, 3º40'N 67°57'W, 1 Jul 1976, H. Thorbjörn. IavH 2828 (2, 16.9 and 17.7 mm SL), caño Caranacoa, rio Inírida, Puerto Inírida, Guainia, 3º51'55"N 67°55'26"W, 29 Mar 1974, M. Blanco. IavH 9937 (2, 15.5 and 15.9 mm SL), mouth of caño Cajaro to Matavén, Cumaribo, Vichada, 4º30'36,2"N 68°3'27.5"W, 14 Mar 2007, Bogotá & F. Villa. ICNMNH 12191 (32, 12.8-26.0 mm SL), caño Bocón, laguna Tonina, rio Inírida, Puerto Inírida, Proyecto Ornamentales Orinoco. MNHLS 23690 (11, 17.0-19.1 mm SL), caño San Joaquin, affluent of left margin of rio Inírida, upstream serras Mavicure, opposite to Remanso community, Vichada, 3º26'47"N 68°0'10"W, 20 Feb 2008, C. Lasso *et al.* MNHLS 23760 (11, 12.3-22.1 mm SL), caño Tonina, affluent of left margin of caño Bocon, Vichada, 3º39'38,3"N 68°3'53.7"W, 20 Feb 2008, C. Lasso *et al.* MNHLS 23774 (6, 15.0-21.1 mm SL), laguna La Ceiba, floodplain of left margin of rio Inírida, between caño Bocon margin and Caranacoa community, Vichada, 3º41'43.3"N 67°57'14.3"W, 20 Feb 2008, C. Lasso *et al.* MNHLS 23818 (3, 16.6-18.6 mm SL), caño Vitina, affluent of rigth margin of rio Inírida, waters upstream Playa de Caranacoa, Vichada, 3º44'30.8"N 67°57'10.5"W, 20 Feb 2008, C. Lasso *et al.*

*Guyana.* ANSP 190682 (3, 18.8-22.0 mm SL), Manari River, rio Takutu, rio Branco, 10.2 km Northest Lethem, Rupununi, 3º26'35"N 59°44'34”W, 1 Nov 2003, M. Sabaj *et al.* SU 50442 (4, 14.5-20.3 mm SL), rio Nappi and others streams of rio Tacatu, rio Branco, Rupununi, 20 Sep 1957, McConnel & H. Rosemary.

*Venezuela.* MBUCV 11301 (4, not measured), caño Chola, Road to Solano, 17.7 km from San Carlos do Rio Negro, rio Negro, Amazonas, 22 Jan 1978, K. Clark. MBUCV 15105 (1, 22.7 mm SL), backwater on rocks at island in the middle of rio Negro, Amazonas, 4 Dec 1984, A. Machado *et al.* MBUCV 15115 (10, 15.9-23.7 mm SL), small stream at caño Urama, ustream Santa Lucia, rio Negro, Amazonas, 6 Dec 1984, R. Vari *et al.* MCNG 53773 (1, 25.1 mm SL), rio Yatua, 1º28’11”N 66º30’30”W, 5 Fev 2011.

*Rio Orinoco, Venezuela.* AMNH 230891 (4, 24.0-31.6 mm SL), rio cora-cora, fourty minutes upstream Yutaje camp, rio Ventuari, Amazonas, 5º36'39"N 66°7'57"W, 27 Apr 1999, S. Schaefer, F. Provenzano & R. Rojas. ANSP 161409 (3, 17.0-21.7 mm SL), caño east margin of rio Casiquiare about 7 km downstream mouth of rio Pamoni, Amazonas, 2º48'N 65°58'W, 20 Mar 1987, B. Chernoff *et al.* ANSP 190678 (71, 11.2-23.7 mm SL), caño Caripo, about 5 min of the confluence of rio Casiquiare with Orinoco, left side, about 3 km upstream mouth, Amazonas, 3º6'N 65°50'W, 16 Mar 1987, B. Chernoff, J. Fernandez & O. Castillo. ANSP 190680 (4, 20.9-25.1 mm SL), stream affluent of rio Casiquiare about 22 km downstream mouth of rio Pamoni, east side, Amazonas, 2º47'N 66°3'W, 20 Mar 1987, B. Chernoff *et al.* ANSP 191389 (1, 28.3 mm SL), caño Parhueña, upstream bridge of Road 12, about 35 km Northeast Puerto Ayacucho, Amazonas, 5º53'30,7"S 67°24'13.5”W, 21 Mar 2010, M. Sabaj Pérez *et al.* MBUCV 7705 (4, 19.1-21.2 mm SL), laguna de Titi, San Fernando de Atabapo, Amazonas, 5 Dec 1973, A. Cortéz. MBUCV 7828 (8, 13.3-19.0 mm SL), caño Guasuriapana, affluent of rio Atabapo, Amazonas, 24 Feb 1974, H. Lopez, O. Silva & A. Cortéz. MBUCV 7854 (18, 11.4-18.9 mm SL), caño Cascaradura, near San Fernando de Atabapo, Amazonas, 1 Dec 1973, A. Cortéz & R. Navarro. MBUCV 8144 (1, not measured), caño Cascaradura, affluent of rio Orinoco, about 7 km east San Fernando de Atabapo-Santa Barbara del Orinoco road, Amazonas, 13 Apr 1972, A. Cortéz. MBUCV 8644 (9, 14.6-23.4 mm SL), caño Moroto, near San Fernando de Atabapo, Orinoco, Amazonas, 13 Feb 1974, A. Cortéz. MBUCV 9462 (8, 15.2-22.2 mm SL), caño Cascaradura, affluent of rio Orinoco, about 7 km east San Fernando de Atabapo-Santa Barbara del Orinoco road, Amazonas, 16 Feb 1974, Moscó *et al.* MBUCV 14807 (2, 25.2 and 29.0 mm SL), rio Baria, about 3 km downstream Base La Neblina camp, Casiquiare, rio Negro, Amazonas, 25 Feb 1985, R. Royero & L. López. MBUCV 14821 (1, 43.2 mm SL), rio Baria, about 2 km downstream Base La Neblina camp, Casiquiare, rio Negro, Amazonas, 25 Feb 1985, R. Royero & L. López. MBUCV 14914 (1, 29.4 mm SL), rio Baria, aside Base La Neblina camp, Casiquiare, Amazonas, 27 Feb 1985, R. Royero *et al.* MBUCV 15120 (28, 14.0-22.1 mm SL), caño Manu, affluent of rio Casiquiare, about 250 m upstream Solano, Amazonas, 7 Dec 1984, R. Vari *et al.* MBUCV 17533 (7, 30.5 mm SL), caño Cascaradura, about 10 km San Fernando de Atabapo-Santa Barbara del Orinoco road, Orinoco, Amazonas, 19 Feb 1974, A. Cortéz & G. Colomine. MCNG 23578 (5, 15.7-19.6 mm SL), rio Atacavi at La Paloma community, 3º8’N 66º59’W, 7 Nov 2011. MCNG 28451 (1, 32.4 mm SL), Laguna José, about 4 km upstream sitio 93-4, Casiquiare, Amazonas, 1º45'50"N 66°50'20"W, 4 Feb 1993. MCNG 33901 (6, 16.-16.3 mm SL), about q hour before Boa Vista, Venezuela-Boa Vista road, Canaima, rio Branco drainage, 3º33’12”N 60º56’13”W, MCNG 35068 (4, 15.6-20.4 mm SL), caño Cachiapo, Casiquiare, Amazonas, 1º56'35"N 66°42'14"W, 19 Jan 1997. MCNG 35137 (2, 17.9 and 19.6 mm SL), rio Casiquiare, laguna Cadamuhedeyedi (diablo), Casiquiare, Amazonas, 2º22'25"N 66°31'5"W, 28 Jan 1997. MCNG 35287 (1, 11.6 mm SL), Laguna Curamoni, rio Casiquiare, Amazonas, 2º37'5"N 66°9'43"W, 1 Feb 1997. MCNG 35388 (2, 19.0 and 22.7 mm SL), rio Casiquiare, downstream caño Cachiapo, Amazonas, 1º56'35"N 66°42'14"W, 19 Jan 1997, KOW97-5. MCNG 35883 (2, 29.9 and 30.8 mm SL), 1.5 km from Rio Autana camp, near 'Los Raudales de Ceguera' community, rio Sipapo, Amazonas, 4º48'16"N 67°28'56"W, 11 Sep 2011, OLM97-7. MCNG 35954 (1, 17.5 mm SL), surroundings 'Puerto Esperanza', rio Sipapo, Amazonas, 4º42'37"N 67°44'58"W, 7 Sep 1997, OLM97-2. MCNG 37716 (204, 13.6-28.1 mm SL), Siapa, Amazonas, 2º5'47"N 66°11'18"W, 18 Jan 1998, LN98-31. MCNG 37948 (7, not measured), caño Iguarapo, tributary of rio Casiquiare, about 100 m of confluence, before Piedra Culimacare, Amazonas, 1º59'N 66°45'W, 8 Jan 1998, LN98-5. MCNG 38158 (13, 12.6-25.3 mm SL), caño Buridajow near confluence with rio Pasimoni, Amazonas, 21 Jan 1998, LN98-37. MCNG 38346 (228, 15.6-28.3 mm SL), drying pool at floodplain, downstream rigth margin of rio Siapa, near rio Emoni, Amazonas, 18 Jan 1998, LN98-30. MCNG 41969 (1, 27.7 mm SL), Laguna Arapacoa at Piedra Arapacoa, rio Pasimoni, Amazonas, 1º51'21"N 66°35'11"W, 7 Jan 1999, LN99-8. MCNG 42021 (9, 16.6-21.6 mm SL), caño Cachiapo, about 1 km upstream confluence of rio Casiquiare, Amazonas, 1º56'34"N 66°42'14"W, 6 Jan 1999, LN99-6. MCNG 42034 (6, 18.3-20.9 mm SL), caño Buridajow, about 1 km upstream confluence of rio Casiquiare Pasimoni, Amazonas, 1º48'27"N 66°33'7"W, 8 Jan 1999, LN99-9. MCNG 42052 (4, 15.4-19.0 mm SL), rio Pasimoni at beach Guisuri, Amazonas, 1º39'21"N 66°34'40"W, 8 Jan 1999, LN99-10. MCNG 42088 (4, 14.6-18.6 mm SL), Laguna de Candela, bank of rio Pasimoni, headwater of rio Yatua, Laguna de Candela, Amazonas, 1º31'42"N 66°33'52"W, 9 Jan 1999, LN99-12. MCNG 47657 (1, 18.8 mm SL), about 2 km upstream mouth of do rio Guapuchi toward "Arenas Blancas" community, rio Ventuari, Amazonas, 4º7'49"N 66°45'14"W, 17 Apr 2003, NKL03-05. MCNG 53716 (1, 28.0 mm SL), rio Yatua at flooded wood, about 3.17 km upstream camp base, Amazonas, 31 Jan 2005, OLM05-5. MNHLS 19046 (2, 23.4 and 30.1 mm SL), stream tributary of left margin of rio Autana, between Salto Pereza and Ceguera community, Amazonas, 4º46'50,6"N 67°37'3"W, 2 May 2006, C. Lasso & J. Rodriguez. MNHLS 22810 (127, 10.8-21.3 mm SL), Laguna Macuruco, Amazonas, 3º55'20"N 67°0'10"W, 25 Nov 2003, C. Lasso *et al.* USNM 269900 (254, 12.7-27.5 mm SL), igarapé Manu tributary of Casiquiare canal, about 250 miles upstream Solano, Amazonas, 7 Dec 1984, R. Vari *et al.* USNM 269901 (41, 7.2-28.5 mm SL), small caño of caño Urami, just upstream Santa Lucia, Amazonas, 1º17'N 66°51'W, 6 Dec 1984, R. Vari *et al.* USNM 272396 (65, 12.2-26.5 mm SL), lagoon Northeast of airport of São Carlos do Rio Negro, Amazonas, 4 Dec 1984. USNM 386132 (10, 16.2-23.8 mm SL), caño Cascadura, San Fernando de Atabapo, Amazonas, 10 Dec 1973.

**Material examined of *Copella vilmae***

*Type material.* SMF 5931 (holotype of *Copella vilmae*, 43.9 mm SL), MHNG 2200.018 (4, 22.1-45.3 mm SL), SMF 5967-77 (11, 18.6-31.8 mm SL), USNM 198135 [ex SU] (4, 33.0-46.4 mm SL), Igarapé Preto, affluent of upper rio Amazonas, near Belém, 60 Km downstream Letícia, Amazonas, Colombia, Dez 1960, H. Shultz.

*Non-type material. Rio Amazonas basin, Colombia.* AMNH 218052 (5, 25.9-34.0 mm SL), rio Amazonas at Letícia, Amazonas, 15-30 Ago 1965. AMNH 218053 (4, 36.5-46.6 mm SL, 1 c&s, 42.4 mm SL), rio Amazonas at Letícia, Amazonas, 15-30 Ago 1965. IavH 8385 (2, 29.6 and 31.8 mm SL), affluent of rio Purité, Letícia, Amazonas, 3º41'54”S 70º12'24.2”W, 27 Mar 2006, Arbeláez *et al.* IavH 11192 (11, 18.8-55.5 mm SL), headwaters of caño Salado, 15 minutes North of Estación Biológica El Zafire, Letícia, Amazonas, 4º0'S 69º53'W, 1 Dec 2008, Bogotá & Arcangel. IavH 11193 (10, 21,9-37,1 mm SL), caño Gravilla, Estación Biológica El Zafire, Letícia, Amazonas, 4º0'S 69º53'W, 1 Dec 2008, Bogotá & Arcangel. IavH 11194 (28, 17.2-39.4 mm SL), stream affluent of caño Salado, Estación Biológica El Zafire, Brazil-Colombia border, Letícia, Amazonas, 4º0'S 69º53'W, 1 Dec 2008, Bogotá & Arcangel. IavH ex 9410 (3, 22.9 mm SL), quebrada 2, affluent of rio Purité, three hours from Salados Varíos, PNN Amacayacu, Letícia, Amazonas, 3º41'37.5”S 70º12'26.5”W, 24 Mar 2006, Arbeláez *et al.* IavH ex 8380 (2, 17.0 and 24.3 mm SL), affluent of rio Purité, Letícia, Amazonas, 3º41'37.5”S 70º12'26.5”W, 24 Mar 2006, Arbeláez *et al.* IavH ex 8384 (2, 20.4 and 21.6 mm SL), affluent of rio Purité, Letícia, Amazonas, 3º41'54”S 70º12'24.2”W, 26 Mar 2006, Arbeláez *et al.* SMF 5966 (2, 13.3 and 23.5 mm SL), same data as type material.
